# A survey of linyphiid spiders from Xishuangbanna, Yunnan Province, China (Araneae, Linyphiidae)

**DOI:** 10.3897/zookeys.460.7799

**Published:** 2014-12-04

**Authors:** Qingyuan Zhao, Shuqiang Li

**Affiliations:** 1 Institute of Zoology, Chinese Academy of Sciences, Beijing 100101, China

**Keywords:** New species, new genus, biodiversity, Southeast Asia, Linyphiinae, Micronetinae, Erigoninae, taxonomy

## Abstract

Eight new genera and 30 new species are described: *Cirrosus* **gen. n.** (type species *Cirrosus atrocaudatus* **sp. n.** (♂♀)), *Conglin* **gen. n.** (type species *Conglin personatus* **sp. n.** (♀)), *Curtimeticus* **gen. n.** (type species *Curtimeticus nebulosus* **sp. n.** (♂)), *Gladiata* **gen. n.** (type species *Gladiata fengli* **sp. n.** (♂)), *Glebala* **gen. n.** (type species *Glebala aspera* **sp. n.** (♂)), *Glomerosus* **gen. n.** (type species *Glomerosus lateralis* **sp. n.** (♂)), *Smerasia* **gen. n.** (type species *Smerasia obscurus* **sp. n.** (♂♀)), *Vittatus* **gen. n.** (type species *Vittatus fencha* **sp. n.** (♂♀)); *Batueta cuspidata* **sp. n.** (♂♀), *Capsulia laciniosa* **sp. n.** (♂), *Dactylopisthes separatus* **sp. n.** (♀), *Gongylidiellum bracteatum* **sp. n.** (♀), *Houshenzinus xiaolongha* **sp. n.** (♂♀), *Laogone bai* **sp. n.** (♂), *Laogone lunata* **sp. n.** (♂♀), *Maro bulbosus* **sp. n.** (♀), *Nasoonaria circinata* **sp. n.** (♂♀), *Neriene circifolia* **sp. n.** (♂♀), *Oedothorax biantu* **sp. n.** (♀), *Oilinyphia hengji* **sp. n.** (♂♀), *Paikiniana furcata* **sp. n.** (♂♀), *Parameioneta bishou* **sp. n.** (♂♀), *Parameioneta multifida* **sp. n.** (♂♀), *Parameioneta tricolorata* **sp. n.** (♂♀), *Tapinopa undata* **sp. n.** (♂), *Theoa bidentata* **sp. n.** (♂♀), *Theoa vesica* **sp. n.** (♂♀), *Vittatus bian* **sp. n.** (♂♀), *Vittatus latus* **sp. n.** (♂♀), *Vittatus pan* **sp. n.** (♂♀). The male of *Kaestneria bicultrata* Chen & Yin, 2000 and the females of *Asiagone perforata* Tanasevitch, 2014 and *Batueta similis* Wunderlich & Song, 1995 are described for the first time; photos of *Bathyphantes paracymbialis* Tanasevitch, 2014 are provided.

## Introduction

The Linyphiidae is the second most diverse spider family in the world (Platnick 2014). Merrett (1963) studied the detailed structure of male palps of 124 British linyphiids, and grouped them into linyphiines and erigonines; Millidge made a great effort to identify the major lineages of the Linyphiidae based on male palpal morphology (1977), epigynal and tracheal system morphology (1984) respectively, and acknowledged seven subfamilies or groups (1993); Hormiga (2000) integrated a numerical cladistic method in his analysis of erigonine phylogenetic relationships, and found support for the monophyly of the subfamily Erigoninae. As suggested by Tanasevitch (2014a), the Linyphiidae should now be divided into the seven subfamilies: Dubiaraneinae Millidge, 1993, Erigoninae Emerton, 1882, Ipainae Saaristo, 2007, Linyphiinae Blackwall, 1859, Micronetinae Hull, 1920, Mynogleninae Lehtinen, 1967 and Stemonyphantinae Wunderlich, 1986.

It is commonly acknowledged that the linyphiid spiders are the dominant spider group of the temperate and cold regions of the northern hemisphere (Marusik and Koponen 2002; Paquin and Dupérré 2003; Scharff et al. 2003; Scharff and Gudik–Sørensen 2006). Although the study of its diversity is still immature, the family seems to be much less diverse in the subtropical and tropical regions (Scharff 1990; Sørensen et al. 2002; Floren and Deeleman–Reinhold 2005). Only a few works have focused upon the Linyphiidae in Southeast Asia (Thorell 1898; Locket 1982; Millidge and Russell–smith 1992; Millidge 1995; Tanasevitch 2010; Tanasevitch 2014b). Thorell described eight *Erigone* species and two *Linyphia* species from Myanmar (Thorell 1895, 1898) but the lack of illustrations in these two works made it difficult to use them for further identifications; Thorell’s work was later revised by van Helsdingen (1969) and Tanasevitch (2010) respectively, and useful figures were added; Locket (1982) investigated linyphiid species from western Malaysia, from where fourteen species were reported and five new genera were established; eleven new genera of Linyphiidae from rain forests of Southeast Asia were described by Millidge and Russell–Smith (1992); Tanasevitch (2014b) established two new genera from Laos: *Asiagone* Tanasevitch, 2014, and *Laogone* Tanasevitch, 2014, and in addition, he reported 6 new species.

Xishuangbanna of southern Yunnan belongs to the transitional zone from tropical southern Asia to subtropical East Asia (Zhu et al. 2006). Only a small number of linyphiid spiders have been reported from Xishuangbanna in the past studies, in contrast with the large number of spiders of less diverse groups found there (Tang and Li 2010; Gao and Li 2014). In 1995, one new genus *Nasoonaria* Wunderlich & Song, 1995 from Xishuangbanna, Yunnan was established and two new species were described: *Nasoonaria sinensis* Wunderlich & Song, 1995 and *Batueta similis* Wunderlich & Song, 1995, the latter is the first species of genus *Batueta* Locket, 1982 to be found outside of western Malaysia (Wunderlich and Song 1995), where this genus was originally described. In 2010, a new species *Neriene poculiforma* Liu & Chen, 2010 was reported from Xishuangbanna, Yunnan (Liu and Chen 2010), which is the twenty-ninth *Neriene* species found in China, making China the country harboring almost half of the total number of *Neriene* species.

Our research on the linyphiids in the tropical rain forest in Xishuangbanna of southern Yunnan has revealed 30 new species, together with 18 species already described, making a total of 48 from this tropical area.

## Material and methods

Specimens in this study were mainly collected by fogging, trapping, sieving and hand-collecting from tree canopy, tree trunks, and leaf-litter in tropical rain forest in Xishuangbanna, Yunnan (the four main collection localities are shown in Fig. 120). Collections were made throughout the year by Qingyuan Zhao, Guo Zheng, Zhiyuan Yao, Zhigang Chen and Guo Tang. Unless otherwise indicated all type specimens are deposited in the Institute of Zoology, Chinese Academy of Sciences in Beijing (IZCAS).

Specimens were examined using a LEICA M205 C stereomicroscope. Further details were studied under a BX51 compound microscope. Most illustrations were made using a camera lucida attached to an Olympus BX51 compound microscope, and then inked on ink jet plotter paper, and the rest were made from photographs. Male and female genitalia were examined and illustrated after being dissected from the spiders.

Left male palps are illustrated, except as otherwise indicated; photos and illustrations of right palps are flipped in figures to allow easy comparison with other species. Epigynes were removed and cleared in lactic acid or warm 10% potassium hydroxide (KOH) solution before illustration. All embolic divisions and vulvae were imaged after being embedded in Arabic gum. Photos were taken with an Olympus c7070 wide zoom digital camera (7.1 megapixels) mounted on an Olympus BX51 compound microscope. Images from multiple focal planes were combined using Helicon Focus (version 3.10) image stacking software. All measurements are given in millimeters. Eye diameters were measured at their widest extent. Leg measurements are shown as: total length (femur, patella, tibia, metatarsus, tarsus). The terminology of Erigoninae genitalic structure follows Hormiga (2000) and Tanasevitch (2014b); the nomenclature of Micronetinae genitalic structure is given after Saaristo and Tanasevitch (1996); the names of Linyphiinae copulatory organs follow van Helsdingen (1969).

Metatarsal trichobothrium (Tm) is given as the ratio of the distance between the proximal margin of the metatarsus and the root of the trichobothrium divided by the total length of the metatarsus (Denis 1949; Locket and Millidge 1953) and Tm value for the first and the fourth leg is given as TmI, TmIV respectively.

The tibial spine formula, which expresses the number of dorsal tibial spines on each of legs I to IV, is given for species in which it differs from the type species of the genus. The patellar spine formula is given only if it differs from the most common one (1-1-1-1).

For the known species only the references for their original description and the synonyms of their current valid names are given. All the other synonyms and references are listed in Platnick’s world spider catalog (2014).

Several species collected from canopy possessed a similar habitus. To verify the accuracy of pairing, the canopy linyphiids specimens were sequenced for DNA barcodes with the primers: 5’-GGTCAACAAATCATAAAGATATTGG-3’ and 5’-TAAACTTCAGGGTGACCAAAAAATCA-3’ (Folmer et al. 1994). This sequence data set, together with COI sequences of linyphiid spiders from BOLD (http://www.boldsystems.org) (see more information in Table 1), was analysed using MEGA 5 (Tamura et al. 2011) and a Neighbor-joining tree was constructed.

**Table 1.** DNA data information of species included in the phylogenetic analysis.

Genus	Species	COI	Genus	Species	COI
*Batueta*	*cuspidata* ♀	KP176798	*Theoa*	*vesica* ♀	KP176814
	*cuspidata* ♂	KP176799		*vesica* ♂	KP176815
	*similis* ♀	KP176800	*Vittatus*	*bian*	KP176816
	*similis* ♂	KP176801		*fencha* ♀	KP176817
*Cnephalocates*	*obscurus*	KC502219		*fencha* ♂	KP176818
*Dismodicus*	*decemoculatus*	KF368011		*latus*	KP176819
*Drapetisca*	*alteranda*	HQ979336		*pan*	KP176820
*Estrandia*	*grandaeva*	KF368087			
*Frontinella*	*communis*	HQ924611			
*Gladiata*	*fengli* ♀	KP176802			
	*fengli* ♂	KP176803			
*Houshenzinus*	*xiaolongha* ♀	KP176804			
	*xiaolongha* ♂	KP176805			
*Incestophantes*	*washingtoni*	KF368176			
*Islandiana*	*flaveola*	HQ924579			
*Kaestneria*	*pullata*	KF368179			
*Linyphantes*	*orcinus*	HQ580723			
*Linyphia*	*triangularis*	FR775771			
*Macrargus*	*multesimus*	HM434066			
*Mermessus*	*maculatus*	HQ979210			
*Microlinyphia*	*mandibulata*	GU682937			
*Nasoonaria*	*circinata* ♀	KP176806			
	*circinata* ♂	KP176807			
*Neriene*	*clathrata*	HQ924547			
*Oedothorax*	*trilobatus*	GU684170			
*Parameioneta*	*tricolorata* ♀	KP176808			
	*tricolorata* ♂	KP176809			
*Pityohyphantes*	*subarcticus*	KF368753			
*Poeciloneta*	*fructuosa*	HQ580516			
*Satilatlas*	*marxi*	KF368759			
*Scotinotylus*	*alpinus*	GU684443			
*Smerasia*	*obscurus* ♀	KP176810			
	*obscurus* ♂	KP176811			
*Souessa*	*spinifera*	KF368806			
*Stemonyphantes*	*blauveltae*	HM434067			
*Tachygyna*	*ursina*	HQ580722			
*Theoa*	*bidentata* ♀	KP176812			
	*bidentata* ♂	KP176813			

**Abbreviations and conventions.** Abbreviations used in the text are given in Table 2. References to figures in cited papers are listed in lowercase type (fig.); figures of this paper are noted with an initial capital (Fig.).

When extra materials are examined and recorded, and the paratype’s collecting information is the same as holotype’s, it will be implied in brackets as [same data as holotype].

**Table 2.** List of anatomical abbreviations used in the text and figures.

**Male palp**	
**AC**	apophysis of convector
**ALP**	anterior projection of lamella
**APC**	anterior process of convector
**APE**	anterior projection of embolic fig
**ARP**	anterior radical process
**ATA**	anterior part of terminal apophysis
**CV**	convector
**DC**	distal apophysis of convector
**DPE**	dorsal projection of embolic fig
**DSA**	distal suprategular apophysis
**DTA**	distal tibial apophysis
**E**	embolus
**EP**	embolus proper
**EPL**	embolic fig
**EBL**	embolic basal lobe
**F**	flag
**FG**	Fickert’s gland
**L**	lamella
**LC**	lamella characteristica
**LLP**	lateral projection of lamella
**MA**	median apophysis
**MM**	median membrane
**MS**	membrosclerum
**P**	parmula
**PC**	paracymbium
**PCA**	proximal cymbial apophysis
**PH**	pit hook
**PL**	pseudolamella
**PLP**	posterior projection of lamella
**PT**	protegulum
**PTA**	prolateral tibial apophysis
**R**	radix
**RA**	radical apophysis
**RBP**	retrobasal cymbial process
**RTA**	retrolateral tibial apophysis
**ST**	subtegulum
**T**	tegulum
**TA**	terminal apophysis of radix
**TH**	thumb of embolus
**TP**	tailpiece of radix
**Epigyne**	
**CD**	copulatory duct
**CO**	copulatory opening
**DP**	dorsal fig
**DPS**	distal part of scape
**FD**	fertilization ducts
**FO**	fertilization opening
**MPS**	median part of scape
**PI**	pit
**PMP**	posterior median fig
**PPS**	proximal part of scape
**PS**	proscape
**PW**	posterior wall of epigyne
**S**	spermatheca
**SC**	scape
**SCD**	scapoid
**St**	stretcher
**VP**	ventral fig
**Somatic morphology**	
**ALE**	anterior lateral eye
**AME**	anterior median eye
**AME-ALE**	distance between AME and ALE
**AME-AME**	distance between AME and AME
**PLE**	posterior lateral eye
**PME**	posterior median eye
**PME-PLE**	distance between PME and PLE
**PME-PME**	distance between PME and PME

## Taxonomy

### Family Linyphiidae Blackwall, 1859

#### Genus *Agyneta* Hull, 1911

*Agyneta*: Hull 1911: 583. Type species *Neriene decora* O. P.-Cambridge, 1871.

##### Agyneta nigra

Taxon classificationAnimaliaAraneaeLinyphiidae

(Oi, 1960)

Meioneta nigra : Oi 1960: 211, figs 318–321 (♂♀).
Agyneta nigra : Tanasevitch 2005: 170, figs 26–30 (♂).

###### Material examined.

1♂, CHINA, Yunnan: Menglun Town: Xishuangbanna Tropical Botanical Garden, 21°55.035'N, 101°16.500'E, elevation ca 588 m, 1.–15.07.2007, primary tropical seasonal rain forest, pitfall traps.

###### Distribution.

China, Korea, Japan, Mongolia, Russia.

###### Remarks.

Lamella characteristica of our specimen matches those illustrated by Saito (Saito 1983: 52, fig. 7) and differ slightly from Tanasevitch’s (Tanasevitch 2005: 170, figs 26–23).

#### Genus *Asiagone* Tanasevitch, 2014

*Asiagone*: Tanasevitch 2014b: 69. Type species *Asiagone signifera* Tanasevitch, 2014 from Laos.

##### Asiagone perforata

Taxon classificationAnimaliaAraneaeLinyphiidae

Tanasevitch, 2014

Figs 1 , 2 , 3 , 4

Asiagone perforata : Tanasevitch 2014b: 69, figs 7–14 (♂).

###### Material examined.

1♂, CHINA, Yunnan: Menglun Town: Xishuangbanna Nature Reserve, 21°57.669'N, 101°11.893'E, elevation ca 790 m, 16.–24.09.2006, primary tropical seasonal rain forest, pitfall traps; 1♂2♀, 21°57.445'N, 101°12.997'E, elevation ca 744 m, 10.–14.08.2006, primary tropical seasonal rain forest, pitfall traps; 1♂, 21°57.669'N, 101°11.893'E, elevation ca 790 m, 16.–24.08.2006, primary tropical seasonal rain forest, pitfall traps; 1♂, 21°54.984'N, 101°16.982'E, elevation ca 656 m, 1.–9.11.2006, secondary tropical seasonal moist forest, pitfall traps; 1♀, 21°54.718'N, 101°16.940'E, elevation ca 645 m, 19.–25.12.2006, secondary tropical seasonal moist forest, pitfall traps; 2♂, 21°57.669'N, 101°11.893'E, elevation ca 790 m, 16.–28.02.2007, primary tropical seasonal rain forest, pitfall traps; 1♂4♀, 21°54.607'N, 101°17.005'E, elevation ca 633 m, 1.–15.04.2007, secondary tropical seasonal moist forest, pitfall traps; 1♂1♀, 21°54.984'N, 101°16.982'E, elevation ca 656 m, 1.–15.04.2007, secondary tropical seasonal moist forest, pitfall traps; 3♂1♀, 21°54.767'N, 101°11.431'E, elevation ca 880 m, 16.–31.04.2007, secondary tropical montane evergreen broad-leaved forest, pitfall traps; 3♂2♀, 21°55.035'N, 101°16.500'E, elevation ca 558 m, 1.–15.07.2007, primary tropical seasonal rain forest, pitfall traps; 3♂2♀, 21°54.607'N, 101°17.005'E, elevation ca 633 m, 1.–15.07.2007, secondary tropical seasonal moist forest, pitfall traps.

###### Diagnosis.

**Figure 1.**
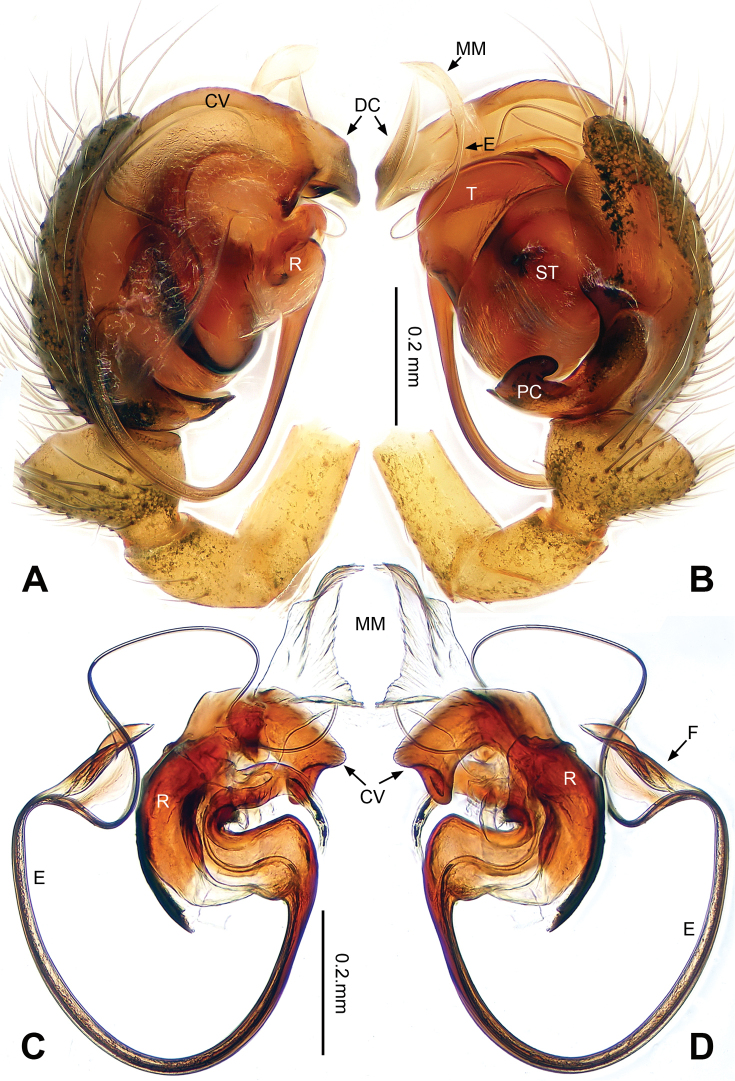
*Asiagone perforata*. **A** Palp, prolateral view **B** Palp, retrolateral view **C** Embolic division, ventral view **D** Embolic division, dorsal view. Scale bars: **A** as **B**, **D** as **C.**

**Figure 2.**
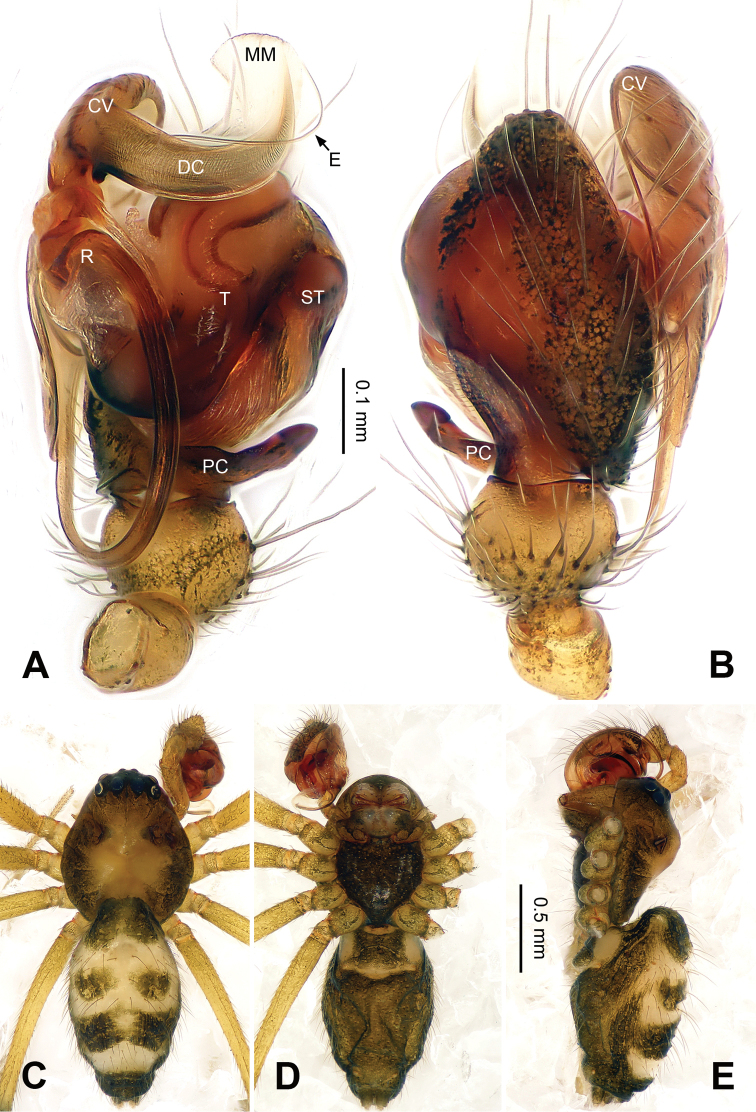
*Asiagone perforata.* **A** Palp, ventral view **B** Palp, dorsal view **C** Habitus, dorsal view **D** Habitus, ventral view **E** Habitus, lateral view. Scale bars: **B** as **A**, **C** and **D** as **E.**

**Figure 3.**
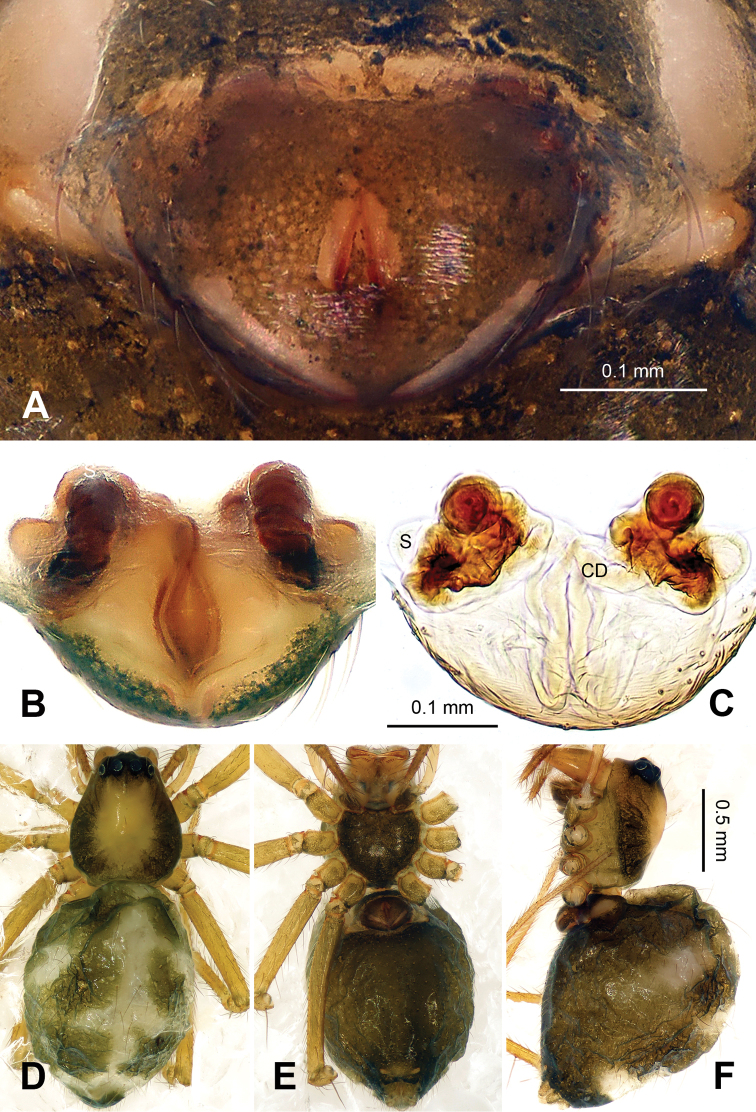
*Asiagone perforata.* **A** Epigyne, ventral view **B** Epigyne, dorsal view **C** Vulva, dorsal view **D** Habitus, dorsal view **E** Habitus, ventral view **F** Habitus lateral view. Scale bars: **B** as **C**, **D** and **E** as **F.**

**Figure 4.**
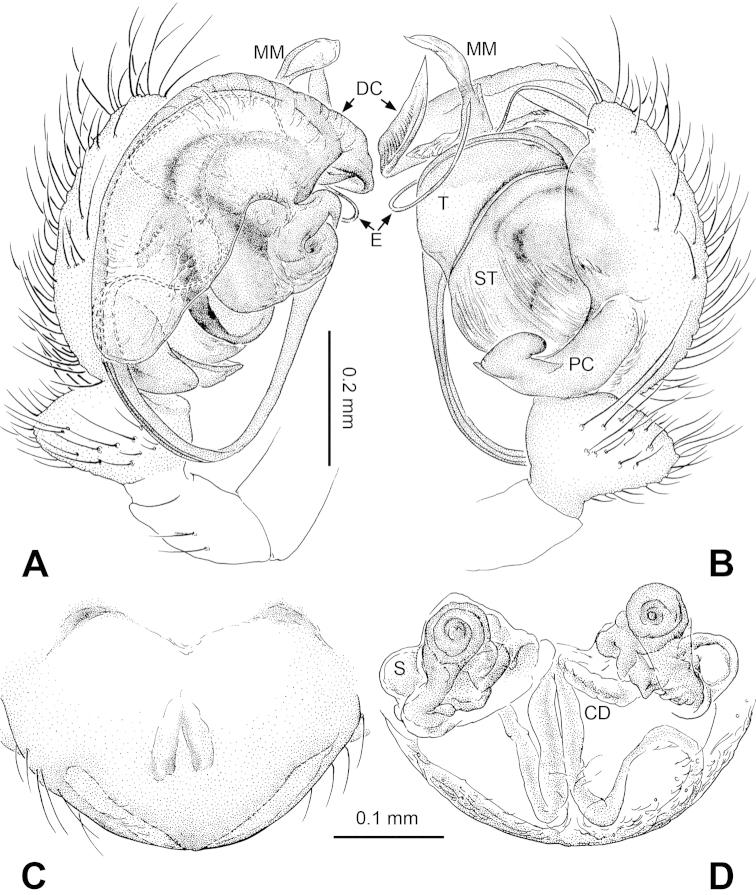
*Asiagone perforata.* **A** Palp, prolateral view **B** Palp, retrolateral view **C** Epigyne, ventral view **D** Vulva, dorsal view. Scale bars: **B** as **A**, **C** as **D.**

This genus was described from Laos by Tanasevitch (2014b), including two species: *Asiagone signifera* Tanasevitch, 2014 and *Asiagone perforata* Tanasevitch, 2014. The male is diagnosed as *Asiagone perforata* by the long, whip-like embolus equipped with a leaf-shaped process at the approximately midpoint (Fig. 1D), horn-shaped distal apophysis of convector (Fig. 2A), and convector’s membraniform extension that covers most part of bulb in prolateral view (Figs 1A, 4A). The female is described for the first time; it possesses a distinct lump-shaped epigyne (Fig. 3A), in which ventral and dorsal figs are fused and the margin of figs is indistinct (Fig. 3B–C).

###### Description.

Male. Well described, e. g. by Tanasevitch (2014b).

Female (one of females from Xishuangbanna). Total length: 2.13. Carapace 0.75 long, 0.63 wide, unmodified, brownish yellow with dark outer margin (Fig. 3D). Sternum 0.41 long, 0.50 wide. Clypeus 0.13 high. Chelicerae promargin with 6 teeth, retromargin with 5 teeth. Eye sizes and interdistances: AME 0.06, ALE 0.08, PME 0.08, PLE 0.08, AME-AME/AME 0.33, PME-PME/PME 0.63, AME-ALE/ALE 0.63, PME-PLE/PLE 0.63, coxae IV separated by 1.43 times their width. Length of legs: I 3.70 (1.04, 0.24, 0.96, 0.88, 0.58), II 3.85 (0.96, 0.25, 1.06, 0.92, 0.66), III 2.95 (0.83, 0.23, 0.70, 0.72, 0.47), IV 3.72 (1.00, 0.20, 1.00, 0.96, 0.56). Leg formula: II-IV-I-III. TmI 0.38, TmIV 0.31. Tibial spine formula: 2-2-1-1. Abdomen pale, with dark green patches. Epigyne: lump-shaped (Fig. 3A), copulatory openings ambiguous; copulatory ducts long and twirled (Figs 3C, 4D); spermathecae widely separated (Fig. 3C).

###### Distribution.

China, Laos.

###### Remarks.

Female of the species is reported for the first time.

#### Genus *Atypena* Simon, 1894

*Atypena*: Simon 1894: 668. Type species *Atypena superciliosa* Simon, 1894 from Southeast Asia.

##### Atypena cirrifrons

Taxon classificationAnimaliaAraneaeLinyphiidae

(Heimer, 1984)

Paranasoona cirrifrons : Heimer 1984: 87, figs 1–8 (♂♀).
Atypena cirrifrons : Tanasevitch 2014b: 72.

###### Material examined.

1♂, CHINA, Yunnan: Menglun Town: Xishuangbanna Nature Reserve, 21°54.738'N, 101°16.940'E, elevation ca 645 m, 19.–25.12.2006, secondary tropical seasonal rain forest, hand-collecting; 1♂, 21°54.607'N, 101°17.005'E, elevation ca 633 m, 19.–26.05.2007, secondary tropical seasonal rain forest, hand-collecting; 1♀, 21°54.772'N, 101°16.043'E, elevation ca 556 m, 18.07.2007, *Paramichelia baillonii* plantation, fogging; 1♂, 21°54.705'N, 101°16.898'E, elevation ca 664 m, 15.11.2009, Lvshilin tropical rain forest, fogging; 1♂, 21°54.609'N, 101°17.190'E, elevation ca 643 m, 17.11.2009, Lvshilin tropical rain forest, fogging; 1♀, 21°53.833'N, 101°17.001'E, elevation ca 618 m, 25.11.2009, teak plantation, fogging; 1♂, 21°53.992'N, 101°16.948'E, elevation ca 590 m, 2.12.2009, G213 Road, *Anogeissus acuminate* plantation, fogging.

###### Distribution.

South China, Vietnam and Laos.

#### Genus *Bathyphantes* Menge, 1866

*Bathyphantes*: Menge 1866: 116. Type species *Bathyphantes longipes* Menge, 1866 (= *Batueta gracilis* (Blackwall, 1841)).

##### Bathyphantes paracymbialis

Taxon classificationAnimaliaAraneaeLinyphiidae

Tanasevitch, 2014

Figs 5 , 6 , 7 , 8

Bathyphantes paracymbialis : Tanasevitch 2014b: 73, figs 15–23 (♂♀).

###### Material examined.

2♂2♀: CHINA, Yunnan: Menglun town: Xishuangbanna Nature Reserve, 21°57.445'N, 101°12.997'E, elevation ca 744 m, 1.–15.03.2007, primary tropical seasonal rain forest, pitfall traps; 1♀, 21°57.669'N, 101°12.893'E, elevation ca 790 m, 19.–25.10.2006, primary tropical seasonal rain forest, pitfall traps; 2♀, 21°57.445'N, 101°12.997'E, elevation ca 744 m, 16.–24.11.2006, primary tropical seasonal rain forest, pitfall traps; 1♂, 21°57.669'N, 101°12.893'E, elevation ca 790 m, 1.–9.12.2006, primary tropical seasonal rain forest, pitfall traps; 1♂, 21°57.445'N, 101°12.997'E, elevation ca 744 m, 5.–12.01.2007, primary tropical seasonal rain forest, hand-collecting; 3♂2♀, 21°57.445'N, 101°12.997'E, elevation ca 744 m, 16.–28.02.2007, primary tropical seasonal rain forest, pitfall traps.

###### Diagnosis.

**Figure 5.**
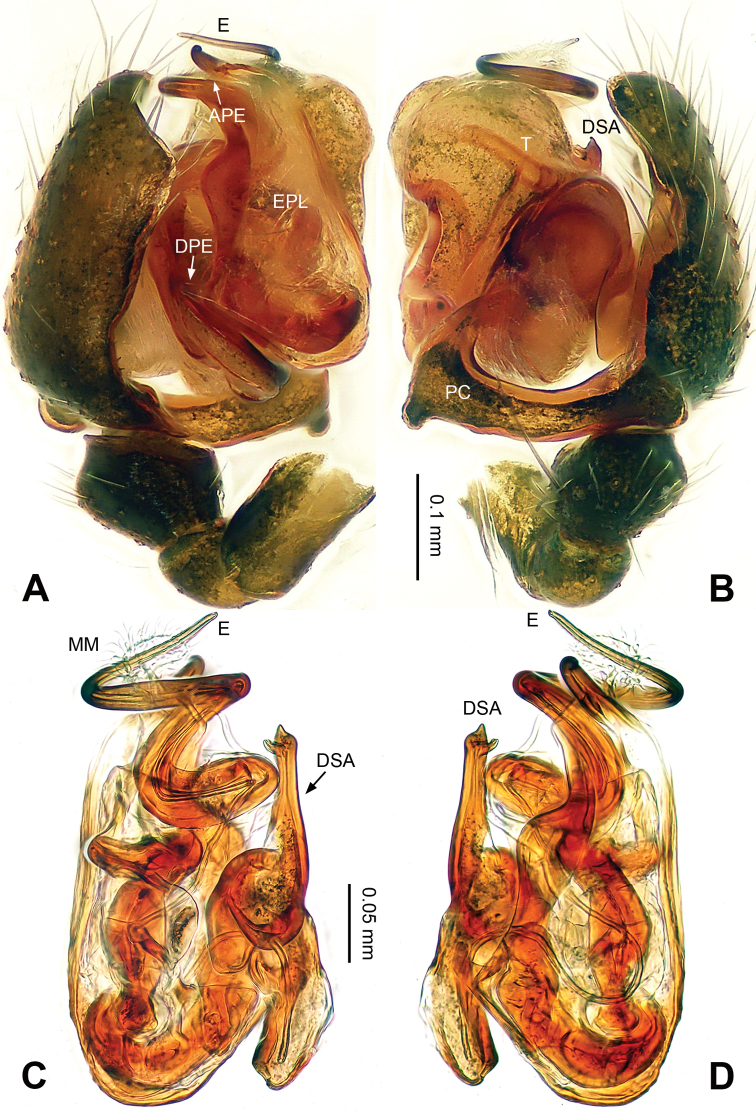
*Bathyphantes paracymbialis.* **A** Palp, prolateral view **B** Palp, retrolateral view **C** Embolic division, retrolateral view **D** Embolic division, ventral view. Scale bars: **A** as **B**, **D** as **C.**

**Figure 7.**
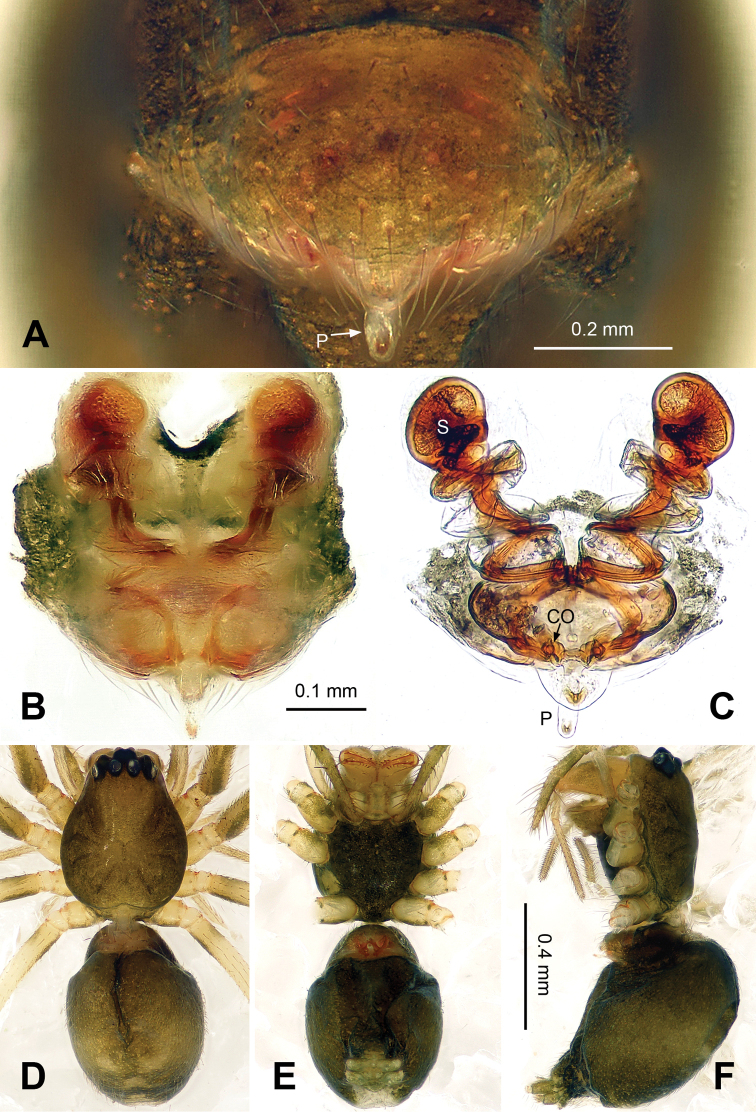
*Bathyphantes paracymbialis.* **A** Epigyne, ventral view **B** Epigyne, dorsal view **C** Vulva, dorsal view **D** Habitus, dorsal view **E** Habitus, ventral view **F** Habitus lateral view. Scale bars: **C** as **B**, **D** and **E** as **F.**

The male can be diagnosed by the small dorsal projection of embolic fig (Tanasevitch 2014b: fig. 16; Fig. 5A, D), two finger-like extensions of distal suprategular apophysis (Fig. 5C–D), and the slimmer embolus forming two coils (Fig. 5C). It differs from *Batueta floralis* Tu & Li, 2006 by the shape of paracymbium: ‘U’-shaped, with a blunt distal end in *Batueta floralis* (Tu and Li 2006: fig. 1B), whereas ‘J’-shaped, with a small projection at the paracymbium in *Batueta paracymbialis* (Tanasevitch 2014b: fig. 17; Fig. 5B). The female is recognized by the short parmula and copulatory ducts (Tanasevitch 2014b: figs 21–22; Fig. 7A–C).

###### Description.

Well described by Tanasevitch (2014b).

###### Distribution.

China, Laos.

#### Genus *Batueta* Locket, 1982

*Batueta*: Locket 1982: 372. Type species *Batueta voluta* Locket, 1982 from Malaysia.

##### Batueta cuspidata sp. n.

Taxon classificationAnimaliaAraneaeLinyphiidae

http://zoobank.org/585AF82A-32FE-40B7-A365-B2AF671FAE44

**Figure 9.**
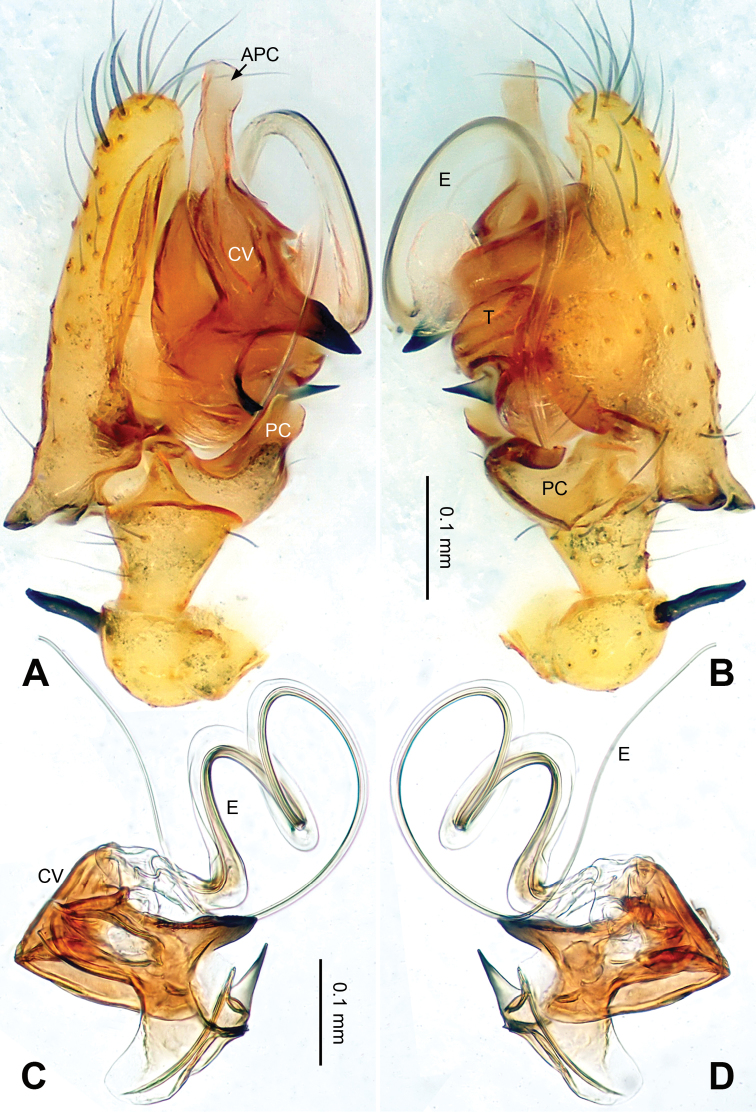
*Batueta cuspidata* sp. n., male holotype. **A** Palp, prolateral view **B** Palp, retrolateral view **C** Embolic division, prolateral view **D** Embolic division, retrolateral view. Scale bars: **A** as **B**, **D** as **C.**

**Figure 10.**
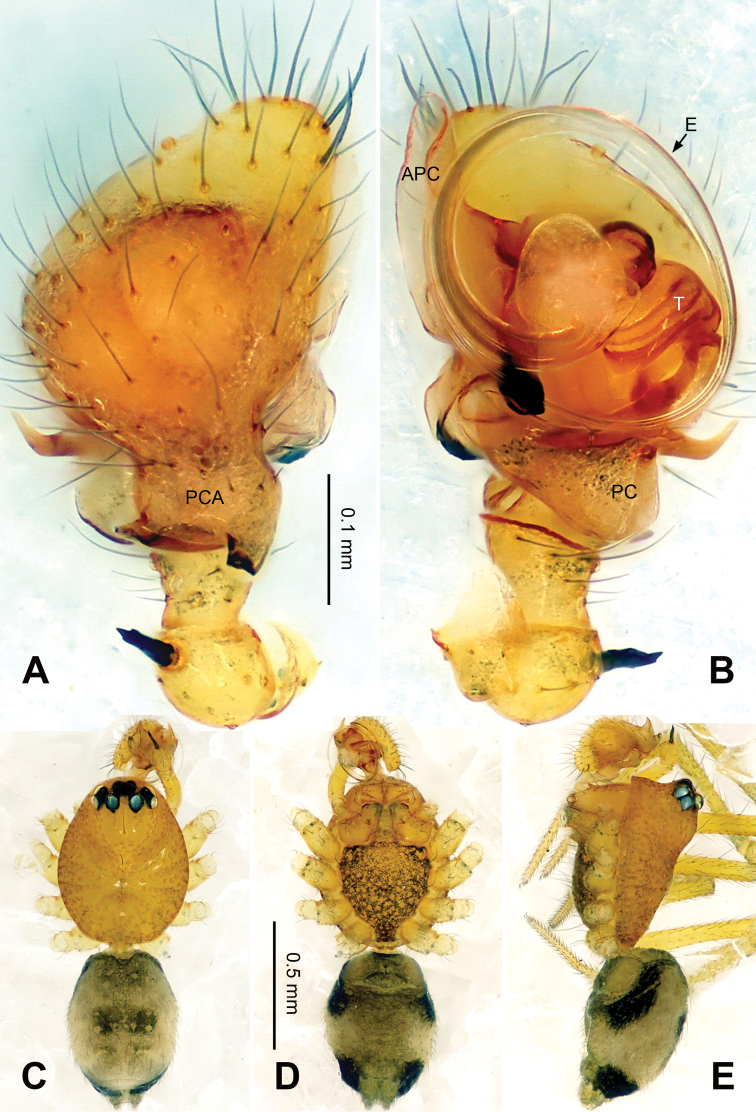
*Batueta cuspidata* sp. n., male holotype. **A** Palp, dorsal view **B** Palp, ventral view **C** Habitus, dorsal view **D** Habitus, ventral view **E** Habitus, lateral view. Scale bars: **B** as **A**, **C** and **E** as **D.**

**Figure 11.**
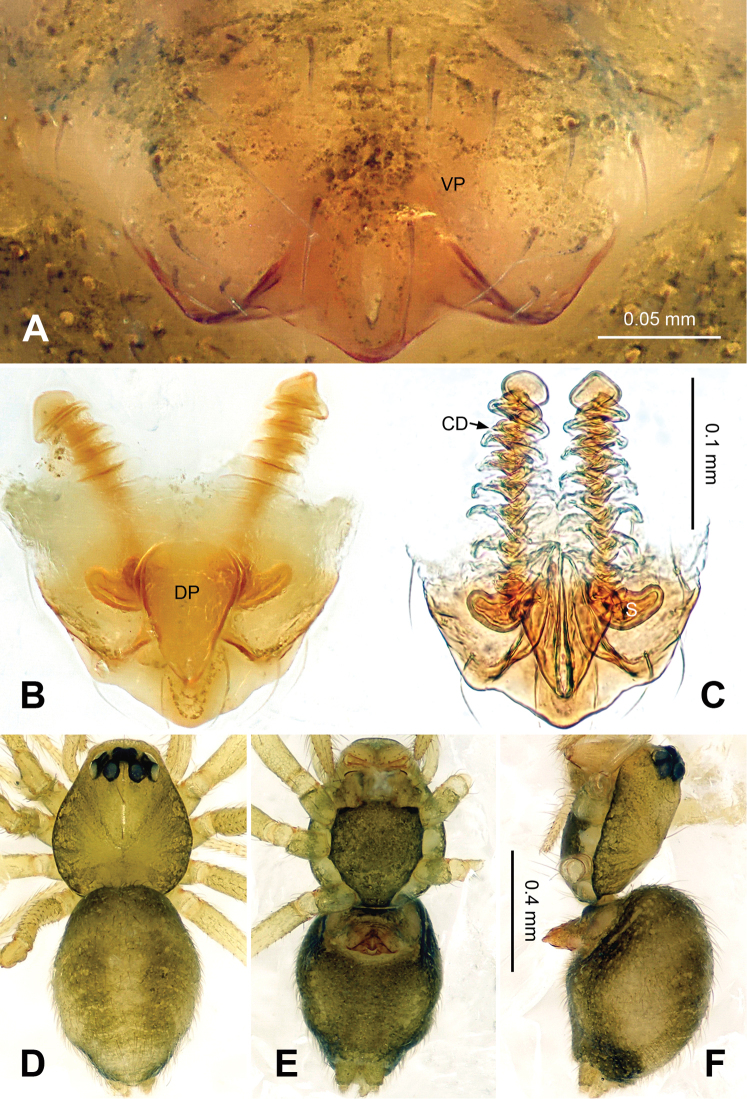
*Batueta cuspidata* sp. n., female paratype. **A** Epigyne, ventral view **B** Epigyne, dorsal view **C** Vulva, dorsal view **D** Habitus, dorsal view **E** Habitus, ventral view **F** Habitus lateral view. Scale bars: **B** as **C**, **D** and **E** as **F.**

**Figure 12.**
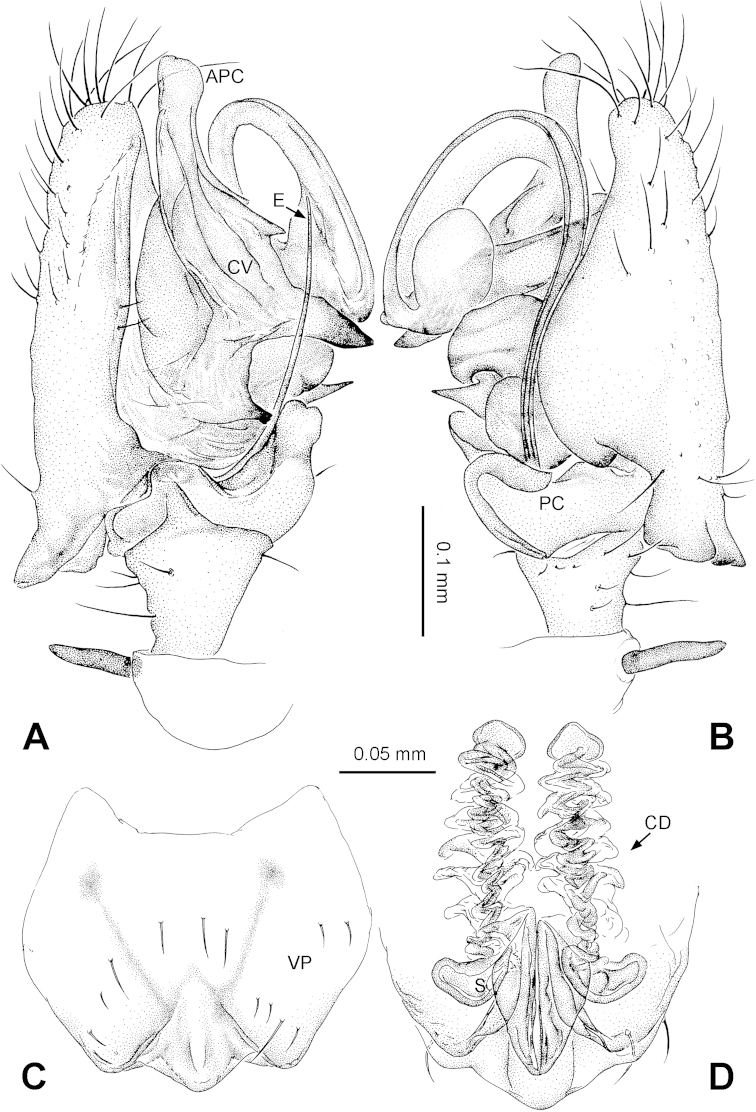
*Batueta cuspidata* sp. n., male holotype (**A–B**) and female paratype (**C–D**). **A** Palp, prolateral view **B** Palp, retrolateral view **C** Epigyne, ventral view **D** Vulva, dorsal view. Scale bars: **A** as **B**, **C** as **D.**

Figs 9 , 10 , 11 , 12

###### Types.

Holotype ♂: CHINA, Yunnan: Mengla County: Xishuangbanna Nature Reserve, Xiaolongha biodiversity preservation corridor, 21°24.161'N, 101°36.412'E, elevation ca 791 m, 16.06.2013, tropical seasonal rain forest, sieving. Paratypes 5♂, same data as holotype; 2♀, Xishuangbanna Tropical Botanical Garden, 21°55.551'N, 101°16.923'E, elevation ca 561 m, 5.–12.10.2007, rubber-tea plantation, hand-collecting; 1♀, 21°54.772'N, 101°16.043'E, elevation ca 556 m, 16.–31.06.2007, *Paramichelia baillonii* plantation, hand-collecting.

###### Etymology.

The name is derived from the Latin ‘cuspidatus’, which means ‘pointed end’, referring to the sharp tip of the lateral apophysis of the convector; adjective.

###### Diagnosis.

This new species is mostly related to *Batueta voluta* Locket, 1982 and *Batueta similis* Wunderlich & Song, 1995, and can be distinguished from them by the two basal outgrowths of cymbium: one is broad, the other is blunt (Fig. 10A), while *Batueta similis* has two slim and curved outgrowths. The convector in *Batueta cuspidata* is well-developed, anterior branch long and erect, slightly folded distally (Figs 9A, 10B), in contrast with the short and pointed ones in *Batueta voluta* (Locket 1982: fig. 58) and *Batueta similis* (Wunderlich and Song 1995: fig. 10). The female is diagnosed by its spiraling copulatory ducts in epigyne (Fig. 11C), which was not clearly noted in Locket’s work, but its ventral fig resembles that in *Batueta voluta* (Locket 1982: figs 62–63).

###### Description.

Male (holotype). Total length: 1.25. Carapace 0.63 long, 0.48 wide, orange, covered with deep impressions; ocular area elevated. Sternum 0.34 long, 0.34 wide. Clypeus 0.16 high. Chelicerae promargin with 3 teeth, retromargin with 3 teeth. Eye sizes and interdistances: AME 0.05, ALE 0.06, PME 0.06, PLE 0.06, AME-AME/AME 0.20, PME-PME/PME 0.67, AME-ALE/ALE 0.50, PME-PLE/PLE 0.33, coxae IV separated by 1.1 times their width. Length of legs: I 2.49 (0.63, 0.16, 0.69, 0.56, 0.45), II 2.14 (0.55, 0.16, 0.56, 0.47, 0.40), III 1.66 (0.41, 0.14, 0.41, 0.39, 0.31), IV 2.19 (0.55, 0.18, 0.56, 0.50, 0.40). Leg formula: I-IV-II-III. TmI 0.16, TmIV absent. Tibial spine formula: 1-1-1-1. Palp: patella with a thick dorsal spine, tibia without apophysis (Figs 9A–B, 10A–B). Cymbium with wide basal outgrowth, the tip of which with two small extensions turning clockwise in dorsal view (in left palp) (Fig. 10A); convector with three arms: anterior arm upright with a folded tip; ventral one with a black pointed tip; posterior one with hooked distal end (Fig. 9A, C); embolus whip-like, forming a coil (Fig. 10B).

Female (one of paratypes). Total length: 1.18. Carapace 0.55 long, 0.45 wide, yellow with green undertone. Sternum 0.32 long, 0.30 wide. Clypeus 0.11 high. Chelicerae promargin with 3 teeth, retromargin with 3 teeth. Eye sizes and interdistances: AME 0.03, ALE 0.05, PME 0.08, PLE 0.05, AME-AME/AME 0.33, PME-PME/PME 0.38, AME-ALE/ALE 0.60, PME-PLE/PLE 0.12, coxae IV separated by 2.9 times their width. Length of legs: I 2.08 (0.52, 0.16, 0.55, 0.41, 0.44), II 1.87 (0.48, 0.16, 0.47, 0.40, 0.36), III 1.56 (0.39, 0.16, 0.36, 0.33, 0.32), IV 1.99 (0.54, 0.18, 0.49, 0.40, 0.38). Leg formula: I-IV-II-III. TmI 0.20, TmIV absent. Tibial spine formula: 1-1-1-1. Abdomen greenish grey with irregular dark patches. Epigyne: ventral fig with three posterior projections, the middle one slightly bigger than the others (Fig. 11A); dorsal fig tongue-shaped (Fig. 11B); copulatory ducts following a double spiral pathway before joining spermathecae (Fig. 11C); spermathecae kidney-shaped, close to each other (Figs 11C, 12B).

###### Distribution.

Known only from type localities.

##### Batueta similis

Taxon classificationAnimaliaAraneaeLinyphiidae

Wunderlich & Song, 1995

**Figure 13.**
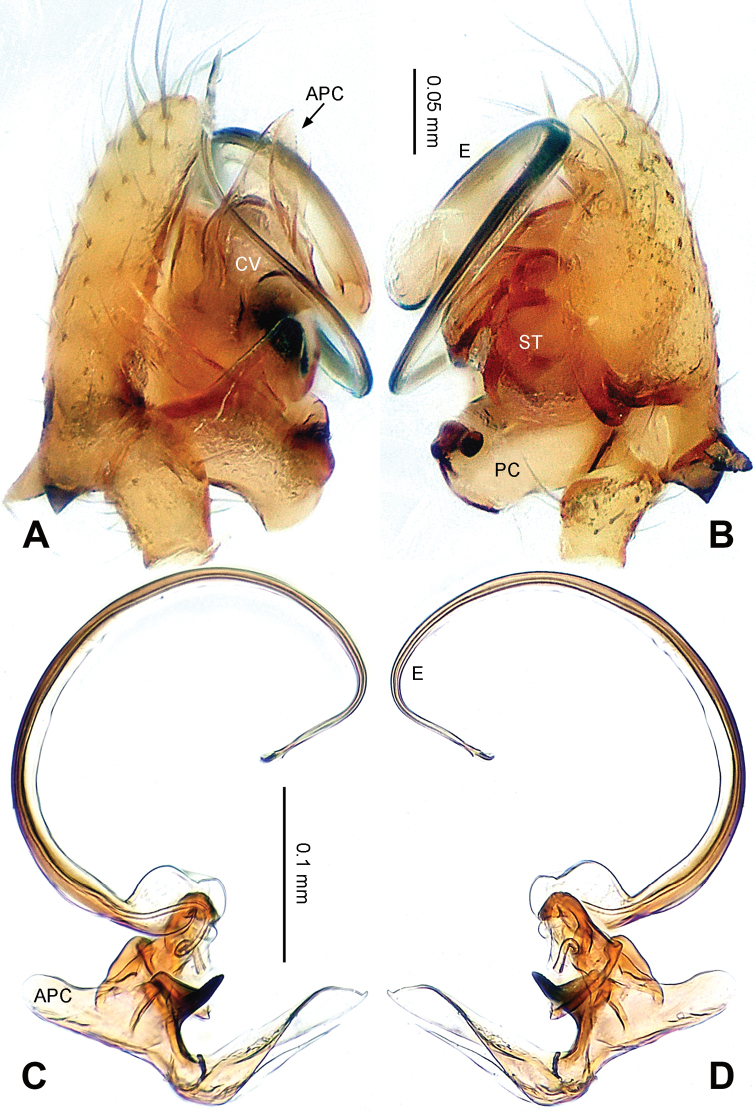
*Batueta similis.* **A** Palp, prolateral view **B** Palp, retrolateral view **C** Embolic division, prolateral view **D** Embolic division, retrolateral view. Scale bars: **A** as **B**, **D** as **C.**

**Figure 14.**
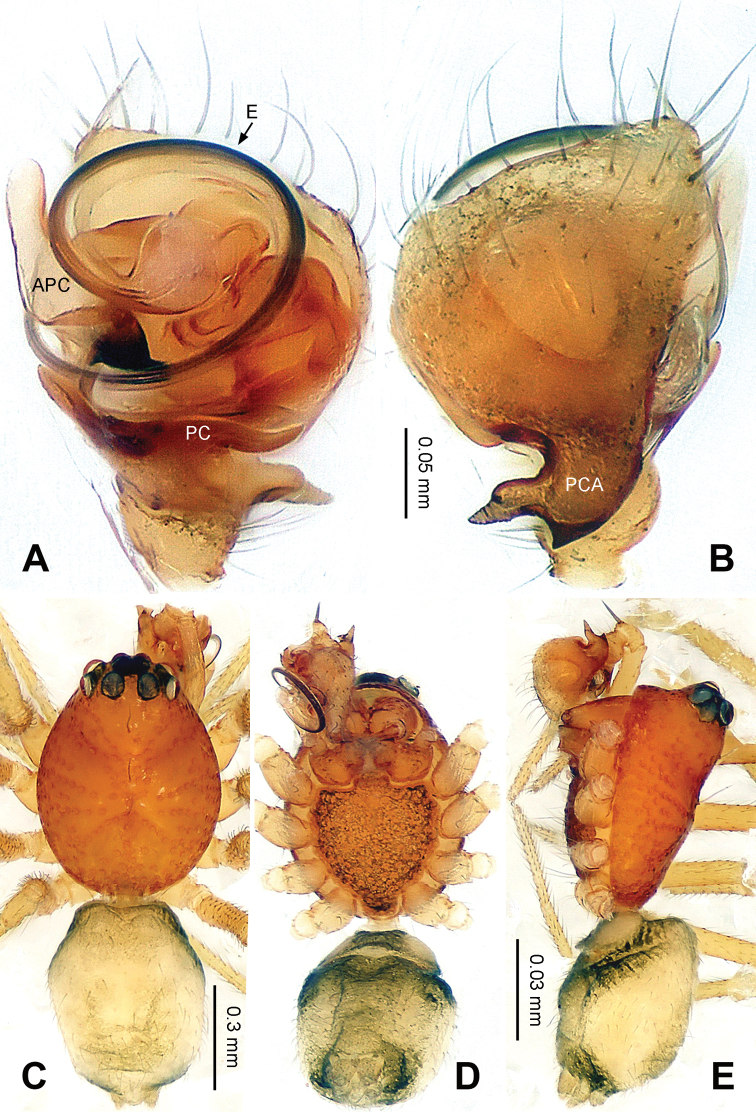
*Batueta similis.* **A** Palp, ventral view **B** Palp, dorsal view **C** Habitus, dorsal view **D** Habitus, ventral view **E** Habitus, lateral view. Scale bars: **A** as **B**, **D** as **C.**

**Figure 15.**
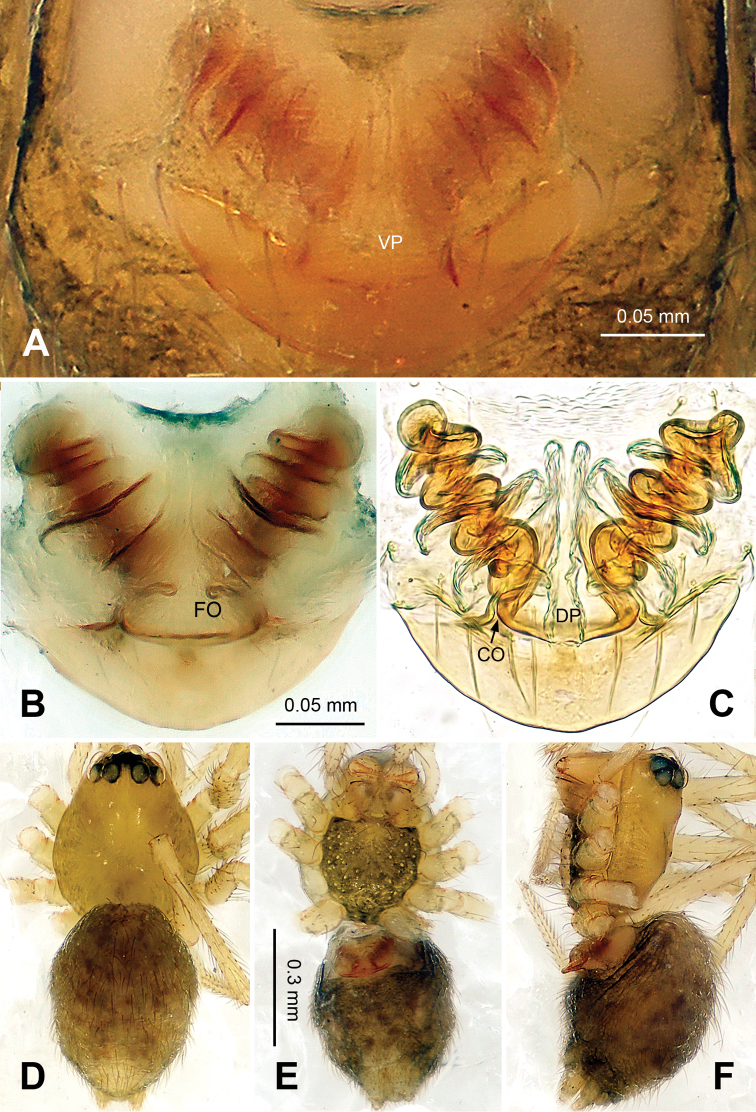
*Batueta similis.* **A** Epigyne, ventral view **B** Epigyne, dorsal view **C** Vulva, dorsal view **D** Habitus, dorsal view **E** Habitus, ventral view **F** Habitus lateral view. Scale bars: **C** as **B**, **D** and **F** as **E.**

**Figure 16.**
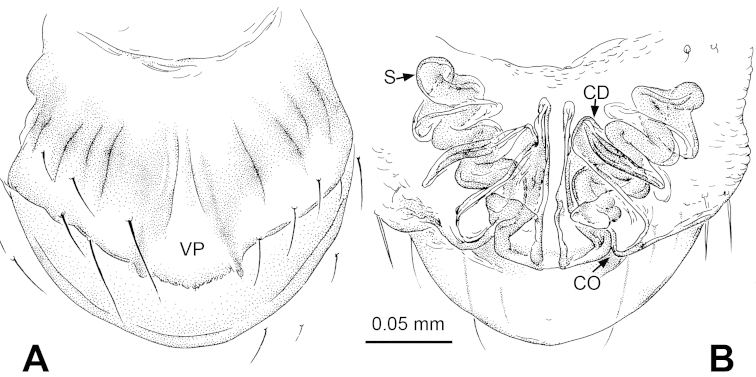
*Batueta similis.* **A** Epigyne, ventral view **B** Vulva, dorsal view Scale bar: **A** as **B.**

Figs 13 , 14 , 15 , 16

Batueta similis : Wunderlich and Song 1995: 345, figs 9–10 (♂).

###### Material examined.

2♂, CHINA, Yunnan: Menglun Town: Xishuangbanna, Tropical Botanical Garden 21°54.200'N, 101°16.923'E, elevation ca 608 m, 16.–24.11.2006, *Paramichelia baillonii* plantation, pitfall traps; 1♂, 21°53.823'N, 101°17.072'E, elevation ca 613m, 1.–9.12.2006, *Parameioneta baillonii* plantation, pitfall traps; 1♂, 21°54.772'N, 101°16.043'E, elevation ca 556 m, 16.–24.12.2006, *Parameioneta baillonii* plantation, pitfall traps; 1♀, 21°54.200'N, 101°16.923'E, elevation ca 608 m, 19.–25.01.2007, *Parameioneta baillonii* plantation, hand-collecting; 1♀, 21°53.823'N, 101°17.072'E, elevation ca 613 m, 19.–26.03.2007, *Parameioneta baillonii* plantation, pitfall traps; 2♀, 21°53.823'N, 101°17.072'E, elevation ca 613 m, 1.–15.04.2007, *Parameioneta baillonii* plantation, pitfall traps; 1♀, 21°54.772'N, 101°16.043'E, elevation ca 556 m, 10.–20.06.2007, *Parameioneta baillonii* plantation, hand-collecting; 1♀, 21°54.200'N, 101°16.923'E, elevation ca 608 m, 1.–15.07.2007, *Parameioneta baillonii* plantation, pitfall traps.

###### Diagnosis.

This species is distinguished from *Batueta cuspidata* sp. n. by a small dorsal fig of epigyne, shorter route and fewer turnings of copulatory ducts (Figs 15C, 16B). The spermathecae are not obviously present in this species (Fig. 15C).

###### Description.

Male. Well described, e. g. by Wunderlich and Song (1995).

Female (one of females from Xishuangbanna). Total length: 1.00. Carapace 0.50 long, 0.47 wide, earthy yellow. Sternum 0.28 long, 0.29 wide. Clypeus 0.08 high. Chelicerae promargin with 3 teeth, retromargin with 2 teeth. Eye sizes and interdistances: AME 0.05, ALE 0.05, PME 0.06, PLE 0.05, AME-AME/AME 0.40, PME-PME/PME 0.50, AME-ALE/ALE 0.20, PME-PLE/PLE 0.50, coxae IV separated by 1.22 times their width. Length of legs: I 1.85 (0.45, 0.15, 0.50, 0.40, 0.35), II 1.70 (0.44, 0.16, 0.43, 0.36, 0.31), III 1.23 (0.28, 0.09, 0.31, 0.30, 0.25), IV 1.80 (0.47, 0.16, 0.47, 0.39, 0.31). Leg formula: I-IV-II-III. TmI 0.24, TmIV absent. Tibial spine formula: 1-1-1-1. Abdomen greenish brown. Epigyne: ventral fig half-rounded, posterior margin smooth and hairless (Figs 15A, 16A); dorsal fig small, elliptical (Fig. 15C); copulatory ducts long, forming a helical structure (Figs 15C, 16B); fertilization openings conspicuous, close to copulatory openings (Fig. 15B).

###### Distribution.

China.

###### Remarks.

Female of the species is reported for the first time.

#### Genus *Capsulia* Saaristo, Tu & Li, 2006

*Capsulia*: Saaristo et al. 2006: 393. Type species *Centromerus tianmushanus* Chen & Song, 1987 from China.

##### Capsulia laciniosa sp. n.

Taxon classificationAnimaliaAraneaeLinyphiidae

http://zoobank.org/A5DC95E2-B3DA-42F9-A92C-F9110B06F90A

Figs 17 , 18 , 19

###### Types.

Holotype ♂: CHINA, Yunnan: Menglun Town: Xishuangbanna, Tropical Botanical Garden 21°53.833'N, 101°17.001'E, elevation ca 618 m, 25.11.2009, teak plantation, fogging.

###### Comparative material.

*Capsulia tianmushana* (Chen & Song, 1987), holotype ♂: CHINA, Zhejiang: Xinmaopeng Town: Mt. Tianmushan, 30°2.400'N, 119°3.000'E, 3.04.1983.

###### Etymology.

This name is derived from the Latin word ‘laciniosus’, which means ‘indented’, referring to the jagged fringe of the anterior terminal apophysis; adjective.

###### Diagnosis.

**Figure 17.**
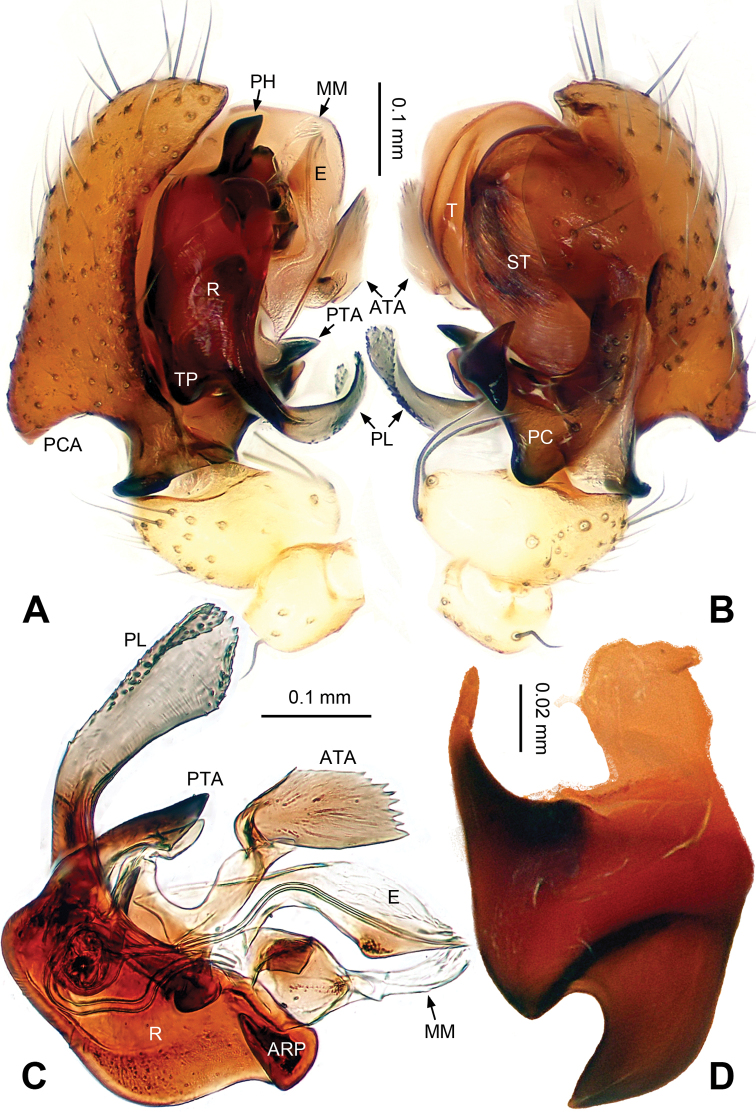
*Capsulia laciniosa* sp. n., male holotype. **A** Palp, prolateral view **B** Palp, retrolateral view **C** Embolic division, ventral view **D** distal part of suprategulum, ventral view. Scale bars: **A** as **B.**

**Figure 18.**
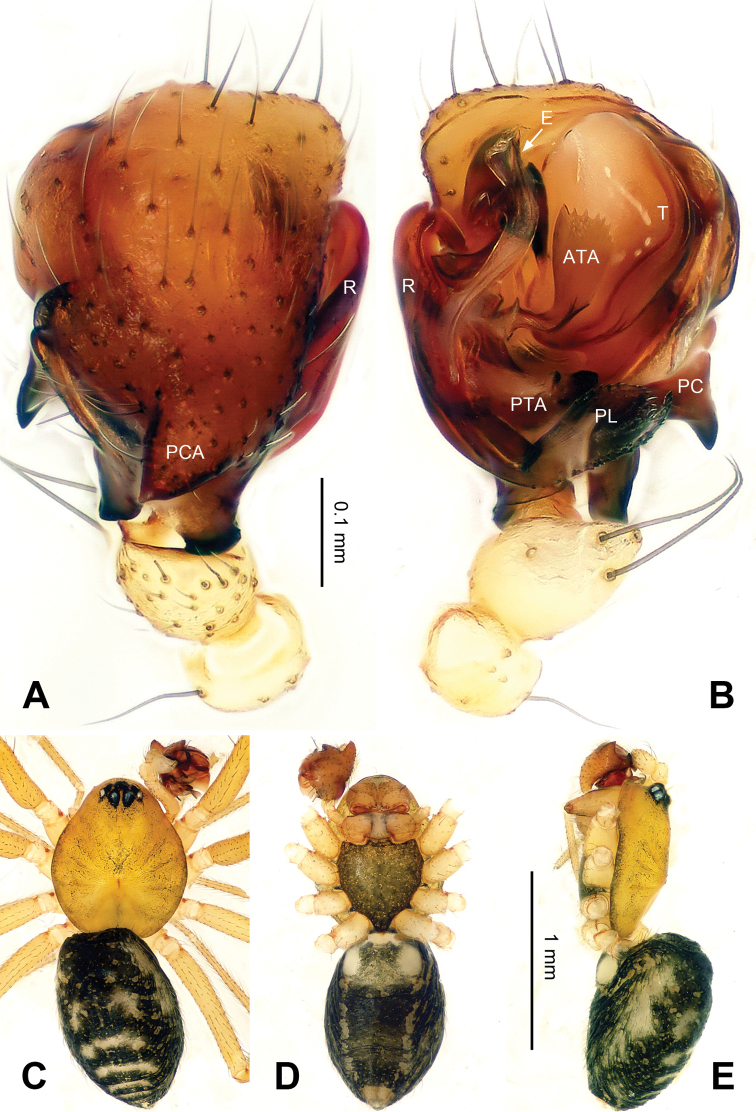
*Capsulia laciniosa* sp. n., male holotype. **A** Palp, dorsal view **B** Palp, ventral view **C** Habitus, dorsal view **D** Habitus, ventral view **E** Habitus, lateral view. Scale bars: **B** as **A**, **C** and **D** as **E.**

This species is similar to *Capsulia tianmushana* (Chen & Song, 1987) greatly, but differs by the following aspects: the edge of anterior terminal apophysis in *Capsulia laciniosa* sp. n. is dentate (Fig. 18C), but smooth in *Capsulia tianmushana*; the apex of pseudolamella in *Capsulia tianmushana* is broad and strongly papillate (Saaristo et al. 2006: figs 18, 19, 21), while narrower and slightly bifurcate in *Capsulia laciniosa* sp. n. (Fig. 17A–C); the thumb of embolus in *Capsulia laciniosa* sp. n. is wider, but less pointed (Fig. 17C).

###### Description.

**Figure 19.**
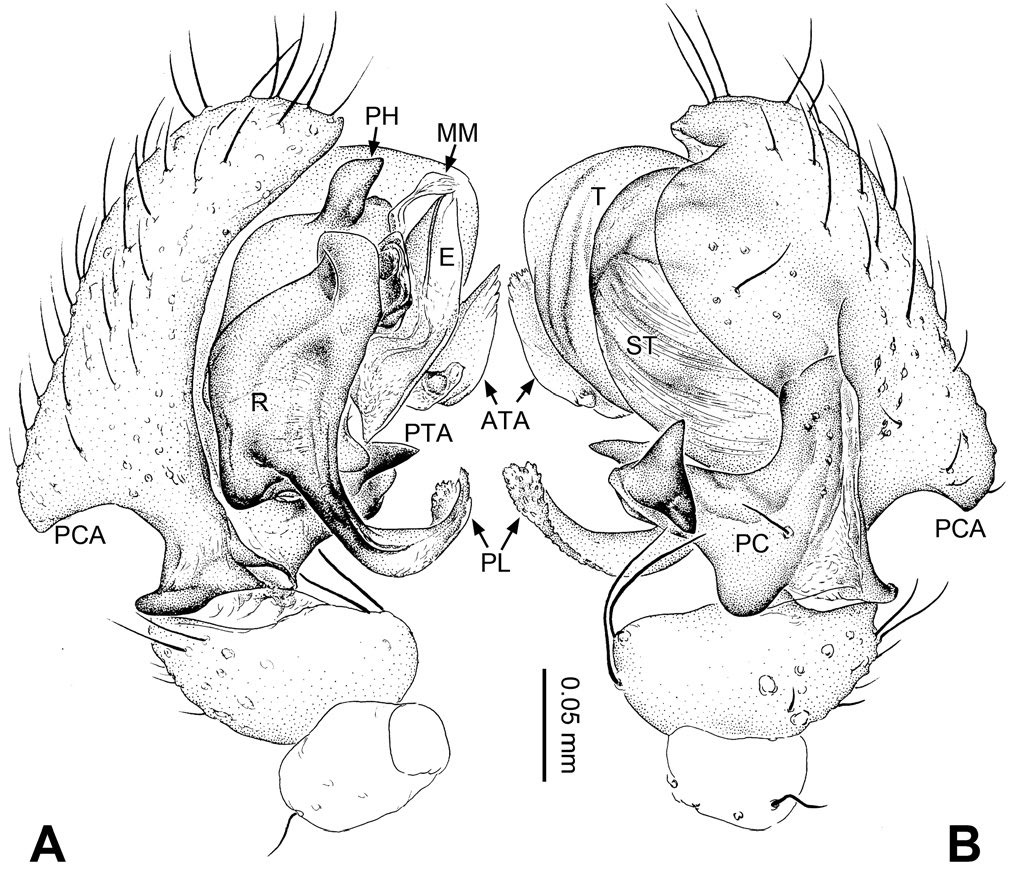
*Capsulia laciniosa* sp. n., male holotype. **A** Palp, prolateral view **B** Palp, retrolateral view. Scale bar: **A** as **B.**

Male (holotype). Total length: 1.85. Carapace 0.90 long, 0.75 wide, yellow. Sternum 0.50 long, 0.50 wide, dark green. Clypeus 0.13 high. Chelicerae promargin with 3 teeth, retromargin with no teeth. Eye sizes and interdistances: AME 0.05, ALE 0.06, PME 0.06, PLE 0.05, AME-AME/AME 0.32, PME-PME/PME 0.17, AME-ALE/ALE 0.33, PME-PLE/PLE 0.50, coxae IV separated by 1.14 times their width. Length of legs: I 3.06 (0.81, 0.25, 0.88, 0.64, 0.48), II 2.91 (0.78, 0.28, 0.76, 0.62, 0.47), III 2.51 (0.65, 0.26, 0.60, 0.60, 0.40), IV 3.33 (0.91, 0.23, 0.94, 0.79, 0.46). Leg formula: IV-I-II-III. TmI 0.25, TmIV absent. Tibial spine formula: 2-2-2-2. Abdomen dark green, with pale transversal stripes. Palp: tibia with two retrolateral trichobothria and one prominent ventral outgrowth equipped with three long setae at tip (Figs 17B, 18B); cymbium with a pointed outgrowth (Figs 17A–B, 19A–B); paracymbium broad at base, ‘V’-shaped, with one folded and slightly bifurcate tip (Figs 17B, 18B); protegulum somewhat triangular; pit hook short and pointed (Fig. 17D). Anterior terminal apophysis broad at tip, with jagged fringe (Figs 17C, 18B); posterior terminal apophysis stout, with sharp end (Fig. 17C); median membrane short, with fringed tip (Fig. 18C); pseudolamella long and curved, with papillate apex (Fig. 17A–B); embolus mesally broad with a pointed tip (Fig. 17C); radix narrow distally with a folded anterior radical process, tailpiece small (Fig. 17A).

Female. Unknown.

###### Distribution.

Known only from type locality.

##### Genus Cirrosus gen. n.

Taxon classificationAnimaliaAraneaeLinyphiidae

http://zoobank.org/4C3D37F2-F8FB-4BD9-BC35-E1CE80C2FDC4

###### Type species.

*Cirrosus atrocaudatus* sp. n.

###### Etymology.

The generic name is an arbitrary combination of letters. Gender is masculine.

###### Diagnosis.

**Figure 20.**
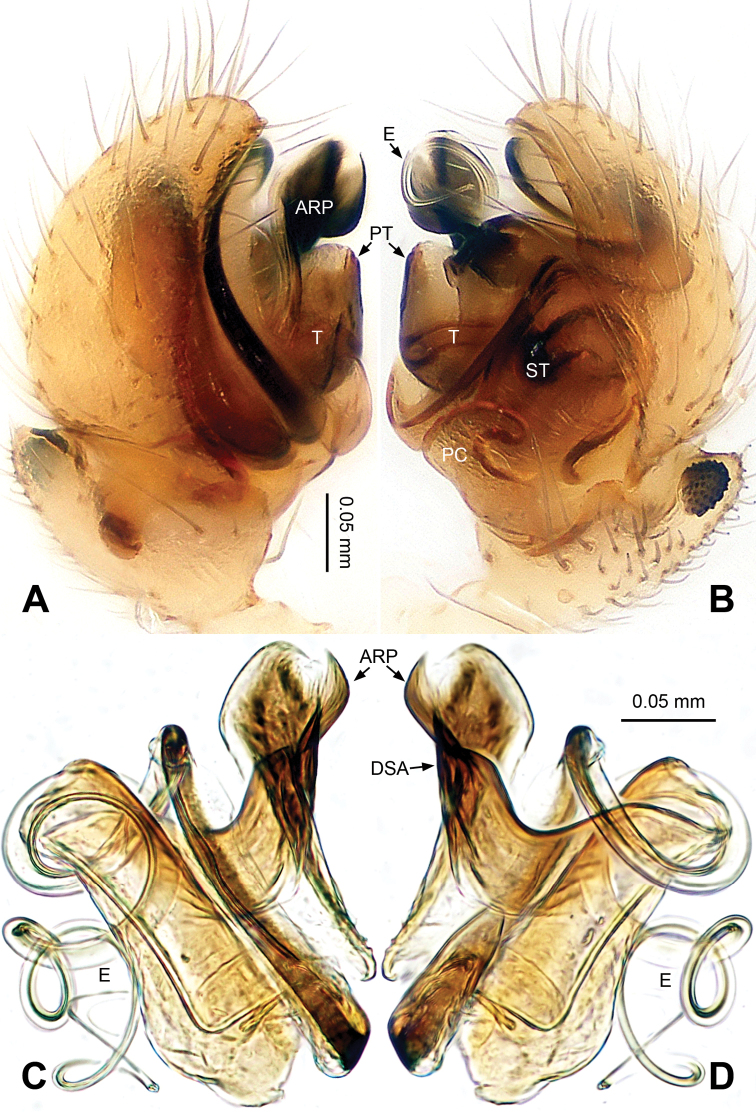
*Cirrosus atrocaudatus* sp. n., male holotype. **A** Palp, prolateral view **B** Palp, retrolateral view **C** Embolic division, prolateral view **D** Embolic division, retrolateral view. Scale bars: **B** as **A**, **C** as **D.**

This new genus is distinguished from all other linyphiids by its unique structure of embolic division, notably, by its hooked distal suprategular apophysis running along the tegulum (Fig. 20B), and long, filiform embolus, starting from the prolateral side of the embolic division, forming several coils (Fig. 20A, C–D), which is rarely seen in other genera.

###### Description.

Small sized Erigoninae. Carapace light yellow, unmodified in both sexes. Chelicerae with 4 promarginal and 3 retromarginal teeth. Chaetotaxy: tibial spine formula: 1-1-1-1. TmI ca 0.70, TmIV ca 0.70. Abdomen pale.

**Figure 21.**
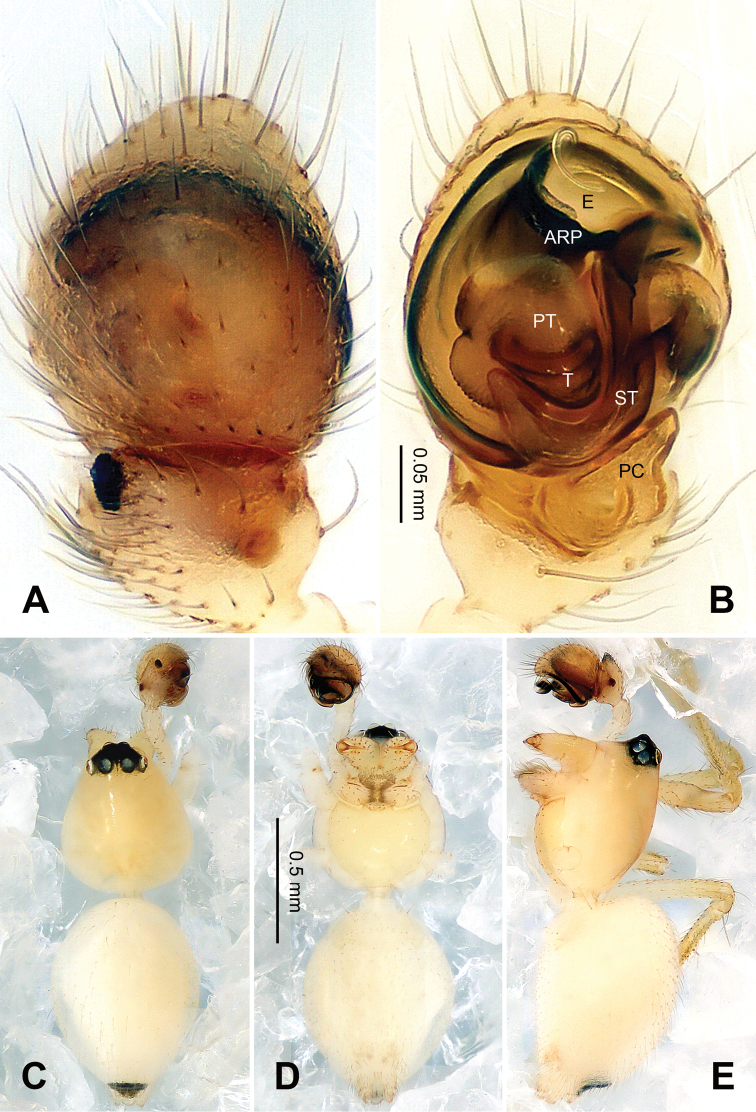
*Cirrosus atrocaudatus* sp. n., male holotype. **A** Palp, dorsal view **B** Palp, ventral view **C** Habitus, dorsal view **D** Habitus, ventral view **E** Habitus, lateral view. Scale bars: **A** as **B**, **C** and **E** as **D.**

Male palp: tibia short, conical, with one retrolateral trichobothrium; tibial dorsal apophysis small and with a small patch of dark papillae (Figs 20B, 21A). Paracymbium ‘J’-shaped, hooked at tip (Figs 20B, 23B), broad in ventral view (Fig. 21B). Distal suprategular apophysis wavy, with a sharp, erect tip in retrolateral view (Fig. 20D); anterior radical process flat, somewhat knob-shaped in lateral view (Fig. 20A–D); embolus long, filiform, and curled up (Fig. 20C–D).

**Figure 22.**
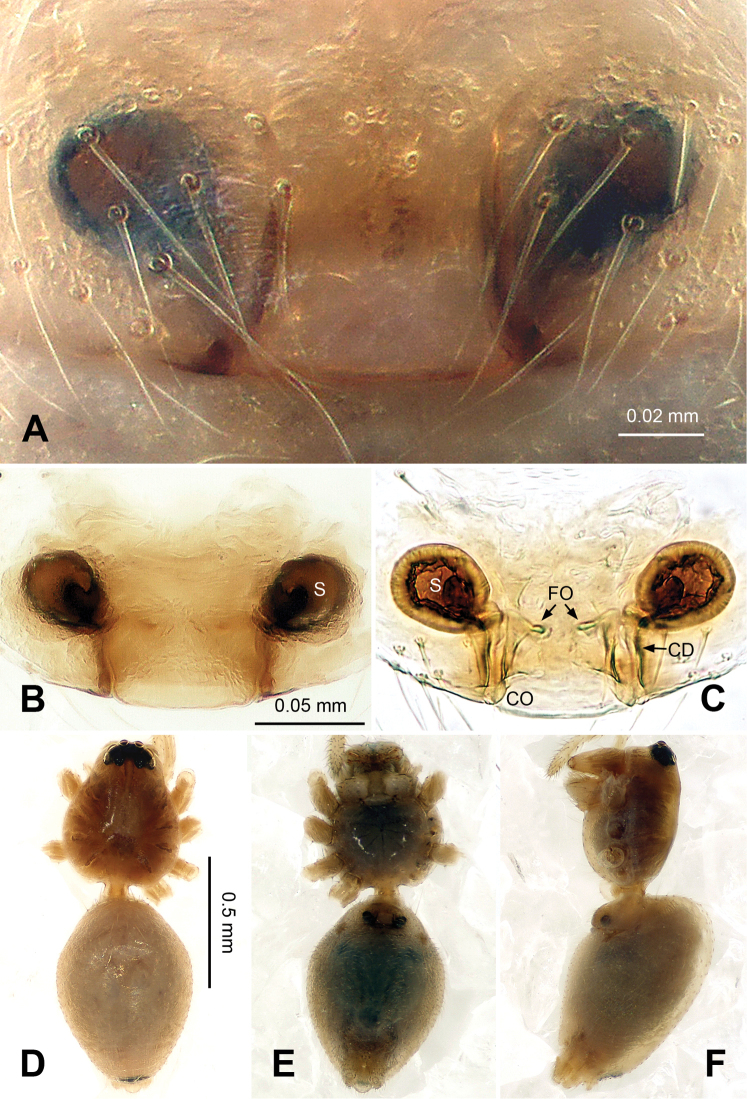
*Cirrosus atrocaudatus* sp. n., female paratype. **A** Epigyne, ventral view **B** Epigyne, dorsal view **C** Vulva, dorsal view **D** Habitus, dorsal view **E** Habitus, ventral view **F** Habitus lateral view. Scale bars: **C** as **B**, **E** and **F** as **D.**

**Figure 23.**
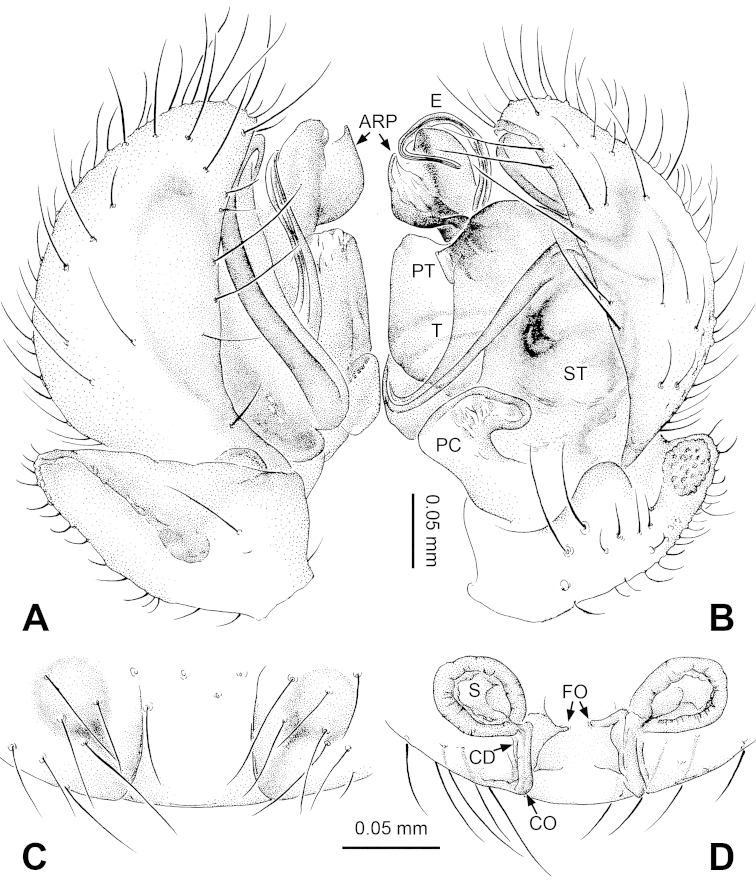
*Cirrosus atrocaudatus* sp. n., male holotype (**A–B**) and female paratype (**C–D**). **A** Palp, prolateral view **B** Palp, retrolateral view **C** Epigyne, ventral view **D** Vulva, dorsal view. Scale bars: **A** as **B**, **C** as **D.**

Epigyne: wide, with copulatory openings at the junction of two figs; copulatory ducts short, simple (Figs 22C, 23D); spermathecae somewhat rounded, separated by 1.5 diameter (Fig. 21B–C).

###### Species composition.

Type species only: *Cirrosus atrocaudatus* sp. n.

###### Distribution.

China.

###### Remarks.

The presence of long, coiled embolus and prominent anterior radical process indicate that it might be distantly related to Southeast Asian genus *Laogone* Tanasevitch, 2014, but close relatives are difficult to hypothesize.

##### Cirrosus atrocaudatus sp. n.

Taxon classificationAnimaliaAraneaeLinyphiidae

http://zoobank.org/CEE65301-FC9C-4195-9514-F169FA2E38FA

Figs 20 , 21 , 22 , 23

###### Types.

Holotype ♂: CHINA, Yunnan: Menglun Town: Xishuangbanna Tropical Botanical Garden, 21°54.600'N, 101°17.084'E, elevation ca 640 m, 17.11.2009, Lvshilin tropical rain forest, fogging. Paratypes 1♂, same data as holotype; 1♂, 21°55.035'N, 101°16.500'E, elevation ca 558 m, 22.07.2009, primary tropical seasonal rain forest, fogging; 3♂1♀, 21°53.646'N, 101°16.957'E, elevation ca 589 m, 26.11.2009, bamboo plantation, fogging.

###### Etymology.

The name for this species comes from the Latin word ‘ater’ and ‘cauda’. The former means ‘black’, and the latter means ‘tail’. The combination refers to the dark end of the male’s abdomen; adjective.

###### Diagnosis.

See diagnosis of the genus.

###### Description.

Male (holotype). Total length: 1.41. Carapace 0.63 long, 0.50 wide, unmodified, light yellow. Sternum 0.31 long, 0.38 wide. Clypeus 0.19 high. Chelicerae promargin with 4 teeth, retromargin with 3 teeth. Eye sizes and interdistances: AME 0.06, ALE 0.06, PME 0.07, PLE 0.06, AME-AME/AME 0.33, PME-PME/PME 0.57, AME-ALE/ALE 0.33, PME-PLE/PLE 0.13, coxae IV separated by 1.92 times their width. Length of legs: I 2.05 (0.56, 0.18, 0.50, 0.50, 0.31), II 2.19 (0.61, 0.17, 0.55, 0.55, 0.31), III 1.64 (0.45, 0.16, 0.34, 0.42, 0.27), IV 2.02 (0.55, 0.17, 0.47, 0.52, 0.31). Leg formula: II-I-IV-III. Abdomen pale, with black posterior tip (Fig. 21C). Palp: see description of the genus.

Female (one of paratypes). Total length: 1.30. Carapace 0.60 long, 0.46 wide, tanned. Sternum 0.36 long, 0.33 wide. Clypeus 0.14 high. Chelicerae promargin with 4 teeth, retromargin with 3 teeth. Eye sizes and interdistances: AME 0.03, ALE 0.04, PME 0.04, PLE 0.05, AME-AME/AME 0.10, PME-PME/PME 0.10, AME-ALE/ALE 0.20, PME-PLE/PLE 0.40, coxae IV separated by 1.60 times their width. Length of legs: I 1.76 (0.50, 0.19, 0.40, 0.38, 0.29), II 2.02 (0.53, 0.18, 0.46, 0.44, 0.31), III 1.46 (0.39, 0.16, 0.30, 0.34, 0.27), IV 1.86 (0.48, 0.16, 0.48, 0.45, 0.29). Leg formula: II- IV-I-III. Abdomen beige, with dark posterior tip. Epigyne: see description of the genus.

###### Distribution.

Known only from type localities.

##### Genus Conglin gen. n.

Taxon classificationAnimaliaAraneaeLinyphiidae

http://zoobank.org/D23AAAD0-13CF-4543-86B3-F037B2ED682E

###### Type species.

*Conglin personatus* sp. n.

###### Etymology.

The generic name is derived from the Chinese Pinyin ‘cóng lín’, meaning ‘forest’, in reference to habitat of the new genus. Gender is masculine.

###### Diagnosis.

**Figure 24.**
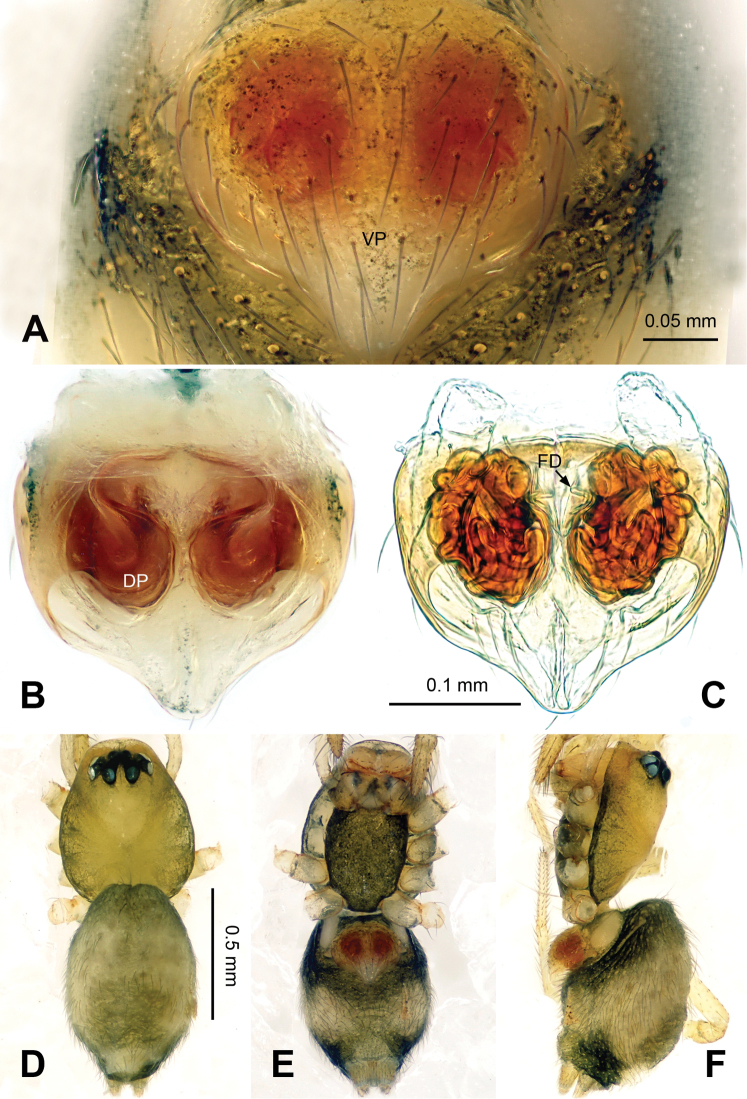
*Conglin personatus* sp. n., female holotype. **A** Epigyne, ventral view **B** Epigyne, dorsal view **C** Vulva, dorsal view **D** Habitus, dorsal view **E** Habitus, ventral view **F** Habitus lateral view. Scale bars: **B** as **C**, **E** and **F** as **E.**

This genus is diagnosed by its complexly routed copulatory ducts and the extended posterior end of the epigynal fig, not found in any other Southeast Asian erigonines (Fig. 24B–C).

###### Description.

Small sized Erigoninae. Carapace unmodified. Chelicerae with 5 promarginal teeth, and 4 retromarginal teeth. Chaetotaxy: tibial spine formula: 1-1-1-1. TmI unknown, TmIV unkown. Abdomen tanned, with dark green patches on the ventral side. Male unknown.

Epigyne: ventral fig round and hunched, with an extension at the posterior end (Fig. 24A); the dorsal fig much shorter than the ventral fig, with curved posterior rim (Fig. 24C). The copulatory ducts long and twisted, and the route difficult to discern (Fig. 24C); no apparent spermathecae present.

###### Species composition.

Type species only: *Conglin personatus* sp. n.

###### Distribution.

China.

##### Conglin personatus sp. n.

Taxon classificationAnimaliaAraneaeLinyphiidae

http://zoobank.org/F9275252-06DD-4B13-878E-B9378A0E0301

Figs 24 , 25

###### Types.

Holotype ♀: CHINA, Yunnan: Mengyang Town: Baihuashan Tunnel: Xishuangbanna nature reserve, 22°09.513'N, 100°53.220'E, elevation ca 894 m, 25.06.2013, tropical seasonal rain forest, sieving.

###### Etymology.

This name is derived from the Latin word ‘personatus’ which means ‘masked’, in reference to the mask-shaped epigynal fig; adjective.

###### Diagnosis.

See diagnosis of the genus.

###### Description.

Female (holotype). Total length: 1.30. Carapace 0.60 long, 0.50 wide, greenish yellow. Sternum 0.40 long, 0.30 wide. Clypeus 0.11 high. Eyes sizes and interdistances: AME 0.03, ALE 0.06, PME 0.06, PLE 0.04, AME-AME/AME 0.67, PME-PME/PME 0.67, AME-ALE/ALE 0.33, PME-PLE/PLE 1.17, Coxae IV separated by 1.17 times their width. Lengths of legs: I 2.03 (0.56, 0.15, 0.60, 0.48, 0.24), II 2.11 (0.55, 0.17, 0.54, 0.44, 0.41), III 1.63 (0.40, 0.15, 0.39, 0.35, 0.34), IV 2.22 (0.60, 0.16, 0.56, 0.48, 0.42). Leg formula: IV-II-I-III. Epigyne: see description of the genus.

Male. Unknown.

###### Distribution.

Only known from its type locality.

##### Genus Curtimeticus gen. n.

Taxon classificationAnimaliaAraneaeLinyphiidae

http://zoobank.org/3222D6B9-5916-4908-9E9C-5D4C2FEBCD40

###### Type species.

*Curtimeticus nebulosus* sp. n.

###### Etymology.

The generic name is an arbitrary combination of letters. Gender is masculine.

###### Diagnosis.

**Figure 26.**
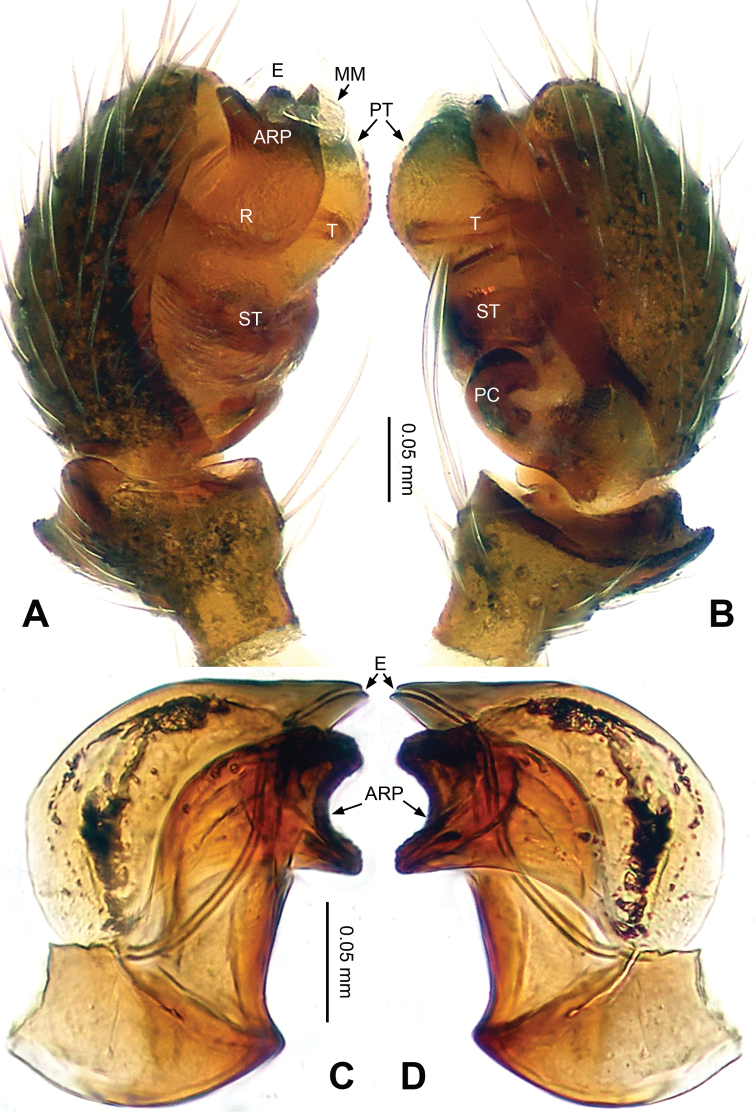
*Curtimeticus nebulosus* p. n., male holotype. **A** Palp, prolateral view **B** Palp, retrolateral view **C** Embolic division, retrolateral view **D** Embolic division, prolateral view. Scale bars: **A** as **B**, **D** as **C.**

**Figure 27.**
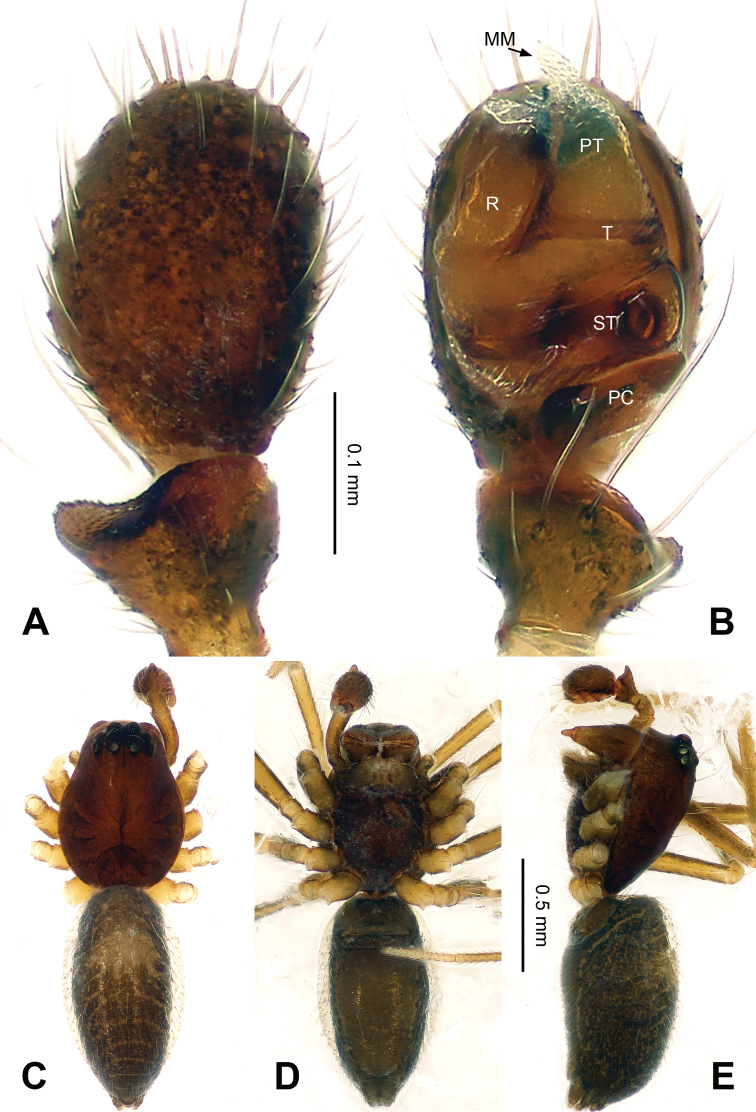
*Curtimeticus nebulosus* sp. n., male holotype. **A** Palp, dorsal view **B** Palp, ventral view **C** Habitus, dorsal view **D** Habitus, ventral view **E** Habitus, lateral view. Scale bars: **B** as **A**, **C** and **D** as **E.**

**Figure 28.**
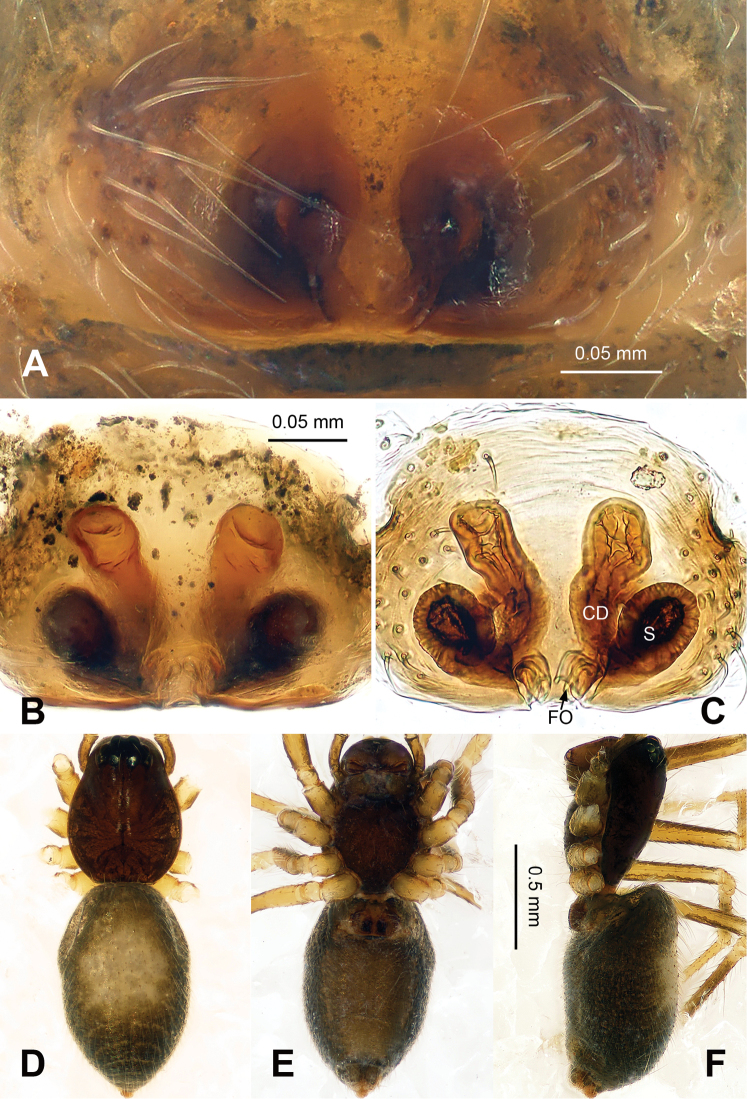
*Curtimeticus nebulosus* sp. n., female paratype. **A** Epigyne, ventral view **B** Epigyne, dorsal view **C** Vulva, dorsal view **D** Habitus, dorsal view **E** Habitus, ventral view **F** Habitus lateral view. Scale bars: **C** as **B**, **D** and **E** as **F.**

**Figure 29.**
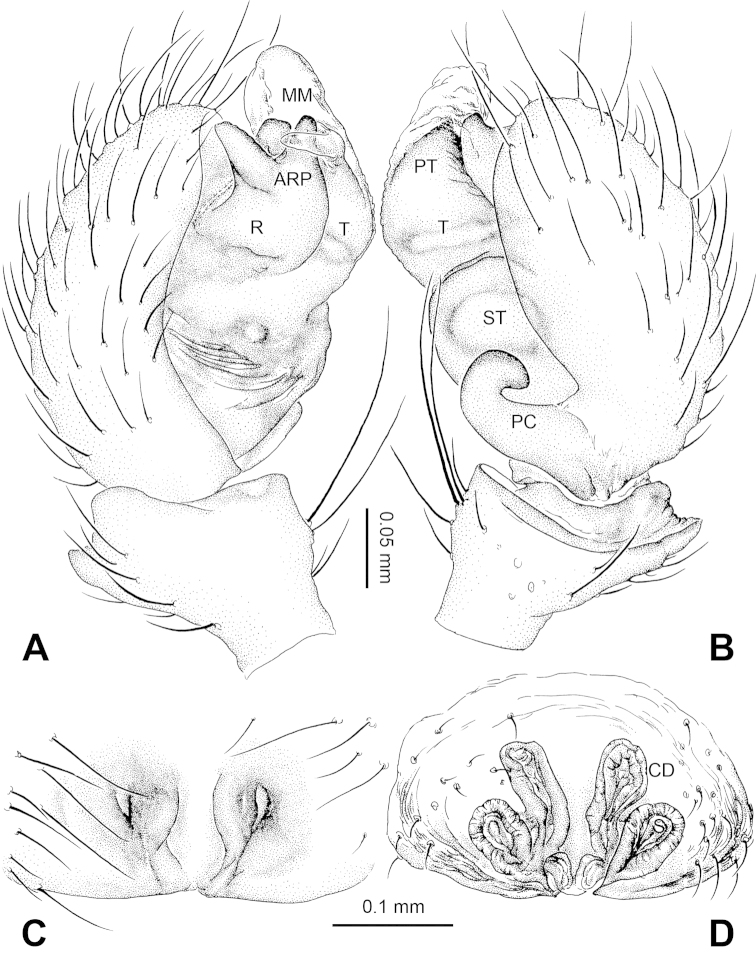
*Curtimeticus nebulosus* sp. n., male holotype (**A–B**) and female paratype (**C–D**). **A** Palp, prolateral view **B** Palp, retrolateral view **C** Epigyne, ventral view **D** Vulva, dorsal view. Scale bar: **B** as **A**, **C** as **D.**

This new genus is diagnosed by its prominent anterior radical process and the stout, short embolus. Its embolic division is similar to those in members of genus *Tmeticus* Menge, 1868, *Donacochara* Simon, 1884. All of them have a simple, straight embolic division with an embolus proper (Millidge 1977: fig. 41), but it differs from the other two by having a bifurcate anterior radical process (Figs 26A, 29A), each branch with a blunt tip (Fig. 26C–D) and an inconspicuous tailpiece. It is also clearly distinguished by the short palpal tibia with broad distal end and the short palpal patella without ventral teeth (Fig. 27C–E). The epigyne in female resembles that in *Oedothorax* Bertkau, 1883 (Roberts 1987: figs 59b–o), but has longer copulatory ducts; The epigyne of female paratype is also quite similar to those in *Paratmeticus bipunctis* (Bösenberg & Strand, 1906) and *Tmeticus nigriceps* (Kulczyński, 1916) in ventral view (Marusik and Koponen 2010: figs 14, 18), but differs by the route of copulatory ducts (Figs 28C, 29D).

###### Description.

Small sized Erigoninae. Carapace unmodified, reddish brown, with dark radial stripes in both sexes. Chelicerae with 5 promarginal teeth, and 4 retromarginal teeth in both sexes. Chaetotaxy: tibial spine formula: 2-2-1-1. TmI ca 0.50 in male, ca 0.60 in female, TmIV ca 0.70 in male, ca 0.50 in female. Abdomen greenish grey with a pale central patch.

Male palp: tibia with two retrolateral trichobothria and several ventral long setae; tibia with two apophyses, the retrolateral one petal-like (Fig. 26A–B), the inner surface of which covered with inconspicuous papillae (Fig. 27A); protegular process prominent, with pointed tip (Fig. 26B). Radix small slender, with two anterior branches (Fig. 26A, C–D); embolus stout, situated between radical process and protegulum (Fig. 26B); in ventral view the tegular sac partially covering the ventral tip of anterior radical process (Fig. 27B).

Epigyne: ventral fig wide, with copulatory openings at the junction of dorsal fig and ventral fig (Fig. 28C); copulatory ducts straight and long, in the shape of cylinder (Fig. 28C); spermathecae elliptical (Fig. 28C); fertilization ducts short, following an arc route (Figs 28C, 29D).

###### Species composition.

Type species only: *Curtimeticus nebulosus* sp. n.

###### Distribution.

China.

##### Curtimeticus nebulosus sp. n.

Taxon classificationAnimaliaAraneaeLinyphiidae

http://zoobank.org/1C18C765-9804-467E-ADE5-EEDF97B54235

Figs 26 , 27 , 28 , 29

###### Types.

Holotype ♂: CHINA, Yunnan: Menglun Town: Xishuangbanna Tropical Botanical Garden, 21°55.035'N, 101°16.500'E, elevation ca 558 m, 22.07.2007, primary tropical seasonal rain forest, fogging. Paratypes 1♀, same data as holotype; 1♀, 21°55.035'N, 101°16.500'E, elevation ca 558 m, 5.–12.01.2007, primary tropical seasonal rain forest, hand-collecting; 1♀, 21°55.035'N, 101°16.500'E, elevation ca 558 m, 19.–25.02.2007, primary tropical seasonal rain forest, hand-collecting; 1♀, 21°55.035'N, 101°16.500'E, elevation ca 558 m, 4.–11.04.2007, primary tropical seasonal rain forest, hand-collecting; 1♂, 21°54.646'N, 101°16.257'E, elevation ca 572 m, 4.–11.05.2007, rubber-tea plantation, hand-collecting; 1♂, 21°55.035'N, 101°16.500'E, elevation ca 558 m, 10.–20.07.2007, primary tropical seasonal rain forest, fogging.

###### Etymology.

The name for this species comes from the Latin word ‘nebulosus’, which means ‘cloudy, foggy’, in reference to the tegular sac covering the tip of the ventral radical process; adjective.

###### Diagnosis.

See diagnosis of the genus.

###### Description.

Male (holotype). Total length: 1.70. Carapace 0.76 long, 0.53 wide, dark red. Sternum 0.40 long, 0.40 wide. Clypeus 0.13 high. Eye sizes and interdistances: AME 0.06, ALE 0.06, PME 0.06, PLE 0.05, AME-AME/AME 0.33, PME-PME/PME 0.66, AME-ALE/ALE 0.50, PME-PLE/PLE 1.00, coxae IV separated by 0.75 time their width. Length of legs: I 2.73 (0.76, 0.20, 0.75, 0.63, 0.39), II 2.51 (0.72, 0.20, 0.65, 0.57, 0.37), III 2.07 (0.56, 0.20, 0.48, 0.48, 0.35), IV 2.69 (0.74, 0.20, 0.70, 0.69, 0.36). Leg formula: I-IV-II-III. Abdomen greenish grey. Palp: see description of the genus.

Female (one of paratypes). Total length: 1.69. Carapace 0.73 long, 0.55 wide, dark red. Sternum 0.70 long, 0.55 wide. Clypeus 0.09 high. Eyes sizes and interdistances: AME 0.07, ALE 0.05, PME 0.05, PLE 0.06, AME-AME/AME 0.29, PME-PME/PME 1.00, AME-ALE/ALE 0.20, PME-PLE/PLE 0.50, coxae IV separated by 0.93 time their width. Length of legs: I 2.65 (0.76, 0.20, 0.70, 0.60, 0.39), II 2.51 (0.72, 0.20, 0.65, 0.57, 0.37), III 2.07 (0.56, 0.20, 0.48, 0.48, 0.35), IV 2.69 (0.74, 0.20, 0.70, 0.69, 0.36). Leg formula: IV-I-II-III. Abdomen greenish grey, with a pale patch in the middle of dorsum. Epigyne: see description of the genus.

###### Distribution.

Known only from type localities.

#### Genus *Dactylopisthes* Simon, 1884

*Dactylopisthes*: Simon 1884: 594. Type species: *Erigone digiticeps* Simon, 1884.

##### Dactylopisthes separatus sp. n.

Taxon classificationAnimaliaAraneaeLinyphiidae

http://zoobank.org/669B3DD3-5B63-4F95-863D-8FBC59F7EE6C

Figs 30 , 31

###### Types.

Holotype ♀: CHINA, Yunnan: Menglun Town: Xishuangbanna Tropical Botanical Garden, 21°55.551'N, 101°16.923'E, elevation ca 561 m, 16.–28.02.2007, rubber-tea plantation, trunk traps. Paratypes 1♀, 21°54.213'N, 101°16.927'E, elevation ca 590 m, 24.11.2006, G 213 road, arbor plantation, fogging; 1♀, 21°55.551'N, 101°16.923'E, elevation ca 585 m, 19.–25.01.2007, rubber tree plantation, hand-collecting; 1♀, 21°54.463'N, 101°15.978'E, elevation ca 569 m, 1.–15.02.2007, rubber-tea plantation, trunk traps; 1♀, 21°54.646'N, 101°16.257'E, elevation ca 572 m, 1.–15.02.2007, rubber-tea plantation, trunk traps; 1♀, 21°54.684'N, 101°16.319'E, elevation ca 585 m, 16.–28.02.2007, rubber tree plantation, trunk traps; 1♀, 21°54.498'N, 101°16.326'E, elevation ca 586 m, 4.–11.05.2007, rubber tree plantation, hand-collecting; 1♀, 21°54.710'N, 101°16.941'E, elevation ca 652 m, 15.11.2009, Lvshilin, tropical seasonal rain forest; 1♀, 21°56.206'N, 101°16.204'E, elevation ca 558 m, 1.12.2009, tropical evergreen rain forest.

###### Etymology.

The name for this species is derived from the Latin word ‘separatus’, which means ‘unconnected’, referring to the separated ventral fig of the epigyne; adjective.

###### Diagnosis.

**Figure 30.**
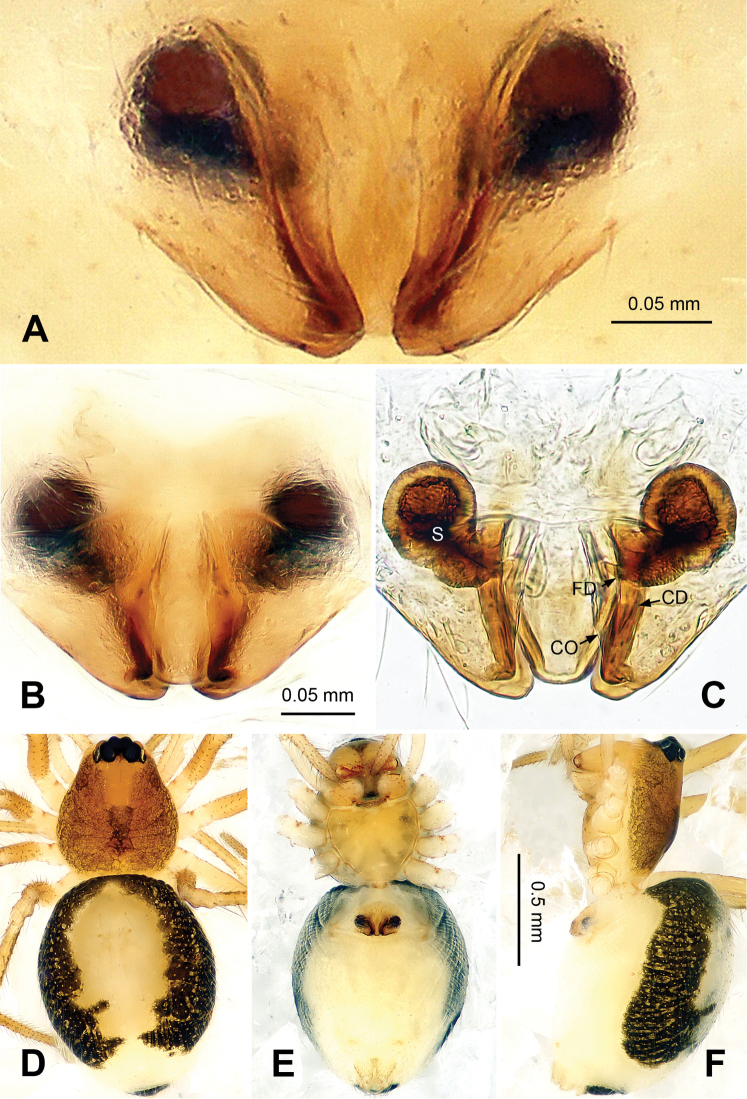
*Dactylopisthes separatus* sp. n., female holotype. **A** Epigyne, ventral view **B** Epigyne, dorsal view **C** Vulva, dorsal view **D** Habitus, dorsal view **E** Habitus, ventral view **F** Habitus lateral view. Scale bars: **C** as **B**, **D** and **E** as **F.**

This species is similar to *Dactylopisthes locketi* (Tanasevitch, 1983) in the general shape of epigyne (Tanasevitch 1989: fig. 127), but different in the detailed structure: the spermathecae in *Dactylopisthes separatus* sp. n. are more separated than those in *Dactylopisthes locketi* (Fig. 30B), and situated closer to the posterior rim of the epigyne; the two pieces of separated ventral fig are narrower and more pointed (Fig. 30A).

###### Description.

**Figure 31.**
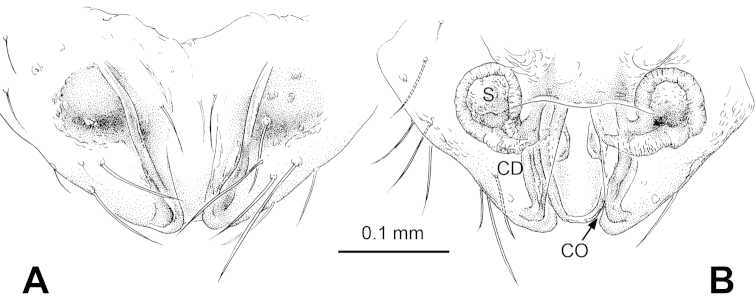
*Dactylopisthes separatus* sp. n., female holotype. **A** Epigyne, ventral view **B** Vulva, dorsal view. Scale bars: **A** as **B.**

Female (holotype). Total length: 1.60. Carapace 0.69 long, 0.55 wide, yellow, with dark margin. Sternum 0.36 long, 0.41 wide. Clypeus 0.14 high. Chelicerae promargin with 4 teeth, retromargin with 3 teeth. Eye sizes and interdistances: AME 0.05, ALE 0.06, PME 0.06, PLE 0.06, AME-AME/AME 0.20, PME-PME/PME 0.67, AME-ALE/ALE 0.13, PME-PLE/PLE 0.17, coxae IV separated by 1.92 times their width. Length of legs: I 2.11 (0.61, 0.20, 0.50, 0.45, 0.35), II 2.22 (0.62, 0.19, 0.55, 0.53, 0.33), III 1.76 (0.48, 0.18, 0.40, 0.42, 0.28), IV 2.24 (0.63, 0.19, 0.50, 0.55, 0.37). Leg formula: IV-II-I-III. TmI 0.69, TmIV 0.65. Tibial spine formula: 2-2-1-1. Abdomen pale, with a greenish grey horseshoe band on dorsum, and a small dark patch at the posterior end. Epigyne: ventral fig subdivided, with two rounded ends (Figs 30A, 31A); dorsal fig tongue-shaped (Fig. 30B); copulatory openings near the posterior margin of ventral fig (Fig. 30C); copulatory ducts short and straight; spermathecae somewhat elliptical, separated by two diameters (Fig. 30C).

Male. Unknown.

###### Remarks.

The diagnosis of this species is solely based on the female specimens and the genera within or related to the *Savignia* group share very similar epigynal structure, which makes it rather difficult to place species in correct genus by comparing epigynes only. The value of TmI of this species and the pattern on its abdomen’s dorsum seem to be different from other existing congeners in *Dactylopisthes*, but it could be tentatively placed in this genus because of its resemblance to *Dactylopisthes locketi*. A more reasonable diagnosis will be made when the male specimens are collected and studied.

#### Genus *Erigone* Audouin, 1826

*Erigone*: Audouin 1826: 320. Type species *Linyphia longipalpis* Sundevall, 1830.

##### Erigone grandidens

Taxon classificationAnimaliaAraneaeLinyphiidae

Tu & Li, 2004

Erigone grandidens : Tu and Li 2004: 420, fig. 2A–J (♂♀).

###### Material examined.

1♂, CHINA, Yunnan: Menglun Town: Xishuangbanna Tropical Botanical Garden, 21°54.459'N, 101°16.755'E, elevation ca 644 m, 20.11.2009, secondary forest; 1♂, 21°53.794'N, 101°17.152'E, elevation ca 594 m, 27.11.2009, low evergreen forest.

###### Distribution.

China, Vietnam.

##### Genus Gladiata gen. n.

Taxon classificationAnimaliaAraneaeLinyphiidae

http://zoobank.org/3C41D5A8-393F-4417-BB08-2A4E13D6B7D2

###### Type species.

*Gladiata fengli* sp. n.

###### Etymology.

The generic name is an arbitrary combination of letters. Gender is feminine.

###### Diagnosis.

The number of spines on tibia IV and the conformation of male palp implies that this genus should belong to subfamily Erigoninae, and the conspicuously large proximal cymbial projection resembles that in erigonine genera *Minicia* Thorell, 1875, *Eskovia* Marusik & Saaristo, 1999, *Sintula* Simon, 1884. The embolus in this new genus takes an anti-clockwise route to form a loop, in contrast with the distal suprategular apophysis turning clockwise (Fig. 33B), which is rare in other erigonines. The atrium in female’s epigyne resembles that in the genus *Ketambea* Millidge & Russell-Smith, 1992, *Tapinocyba* Simon, 1884, but the copulatory ducts follow a different route.

###### Description.

Median sized Erigoninae. Male with post-ocular area slightly elevated, with a row of setae along the axis from clypeus to the fovea (Fig. 33E). Chelicerae with 5 promarginal teeth, and 4 retromarginal teeth. Abdomen with distinct pattern. Chaetotaxy: tibial spine formula: 2-2-1-1. TmI ca 0.37, TmIV ca 0.65.

**Figure 32.**
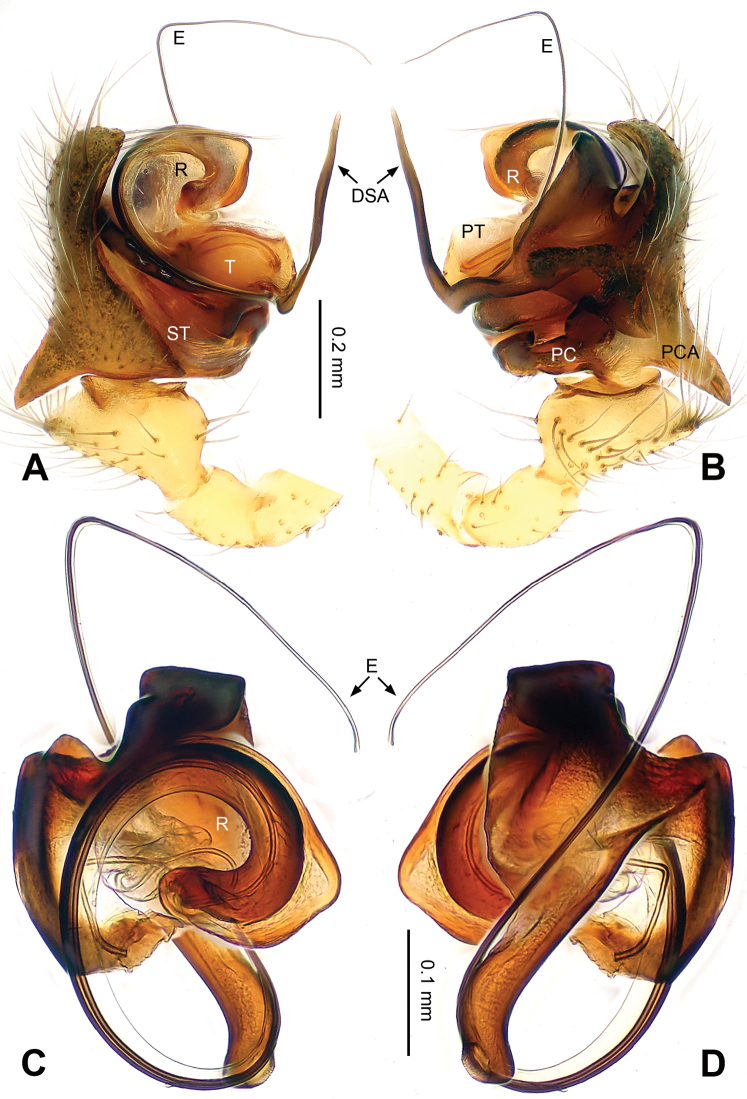
*Gladiata fengli* sp. n., male holotype. **A** Palp, prolateral view **B** Palp, retrolateral view **C** Embolic division with broken DSA, prolateral view **D** Embolic division with broken DSA, retrolateral view. Scale bars: **B** as **A**, **C** as **D.**

Male palp: tibia with 4 trichobothria, 2 retrolateral and two dorsal, tibia dorsally humped, equipped by a row of long setae (Fig. 32A–B); cymbium with a prominent, pointed projection (Fig. 32A–B); paracymbium ‘J’-shaped, basally broad, with an extended distal end (Fig. 32B); distal suprategular apophysis basally broad, stretching along the tegulum then turning up to form a spine (Fig. 32B); radix straight, reduced (Fig. 32A); no obvious tailpiece present; embolus long, thread-like, starting from the prolateral side of the radix (Fig. 32A).

Epigyne: ventral fig bulge, dorsal fig tongue-shaped, with an obvious atrium lying in between (Fig. 34A); copulatory ducts long, twisted (Fig. 34C); spermathecae small (Fig. 34C).

###### Species composition.

Type species only: *Gladiata fengli* sp. n.

###### Distribution.

China.

##### Gladiata fengli sp. n.

Taxon classificationAnimaliaAraneaeLinyphiidae

http://zoobank.org/BDA90FB1-5390-4C60-8391-B1A1527BA4AA

**Figure 33.**
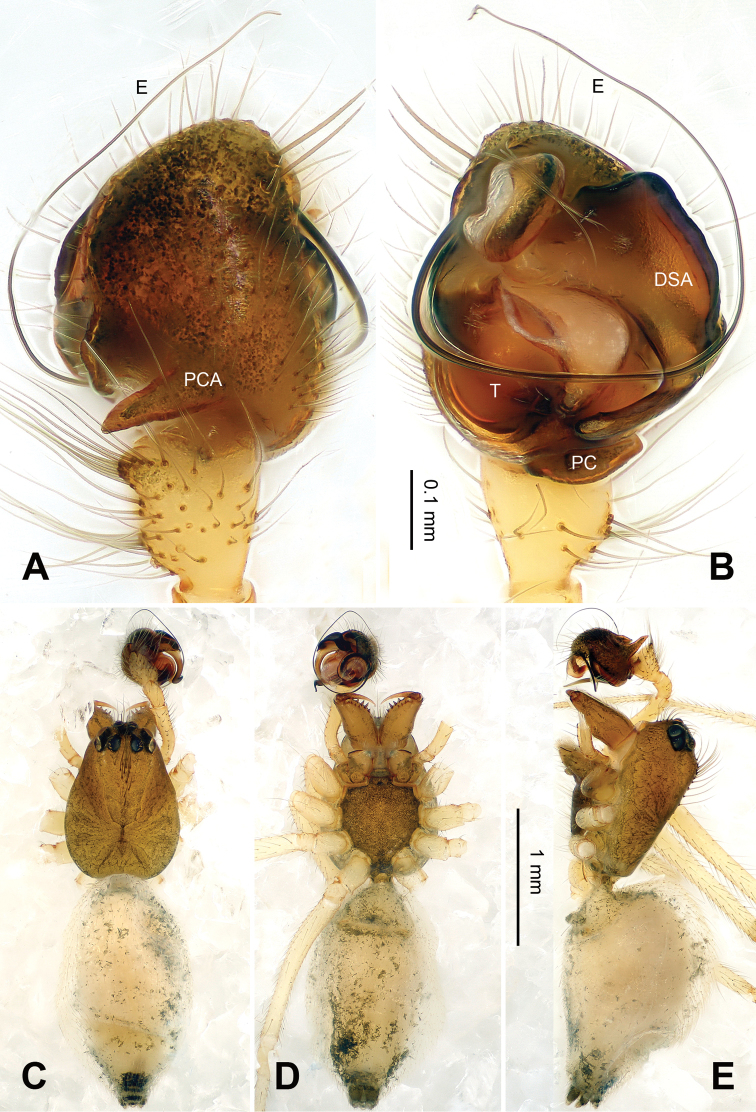
*Gladiata fengli* sp. n., male holotype. **A** Palp, dorsal view **B** Palp with broken DSA, ventral view **C** Habitus, dorsal view **D** Habitus, ventral view **E** Habitus, lateral view. Scale bars: **A** as **B**, **C** and **D** as **E.**

**Figure 35.**
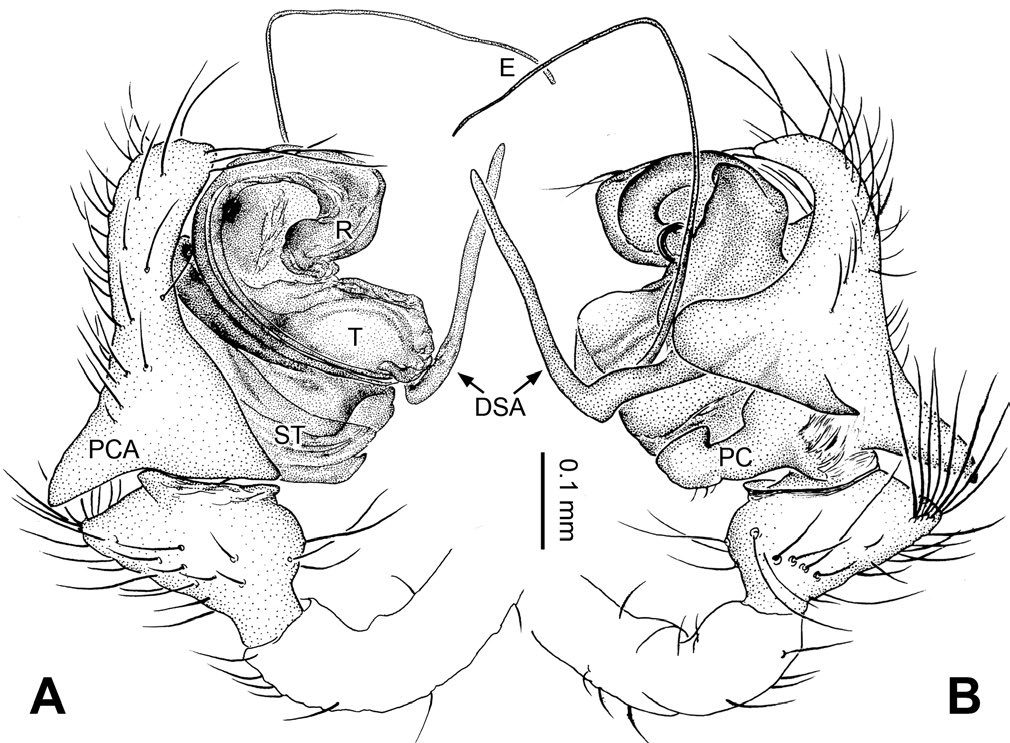
*Gladiata fengli* sp. n., male holotype. **A** Palp, prolateral view **B** Palp, retrolateral view. Scale bar: **A** as **B.**

Figs 32 , 33 , 34 , 35

###### Types.

Holotype ♂: CHINA, Yunnan: Menglun Town: Xishuangbanna Nature Reserve, 21°57.445'N, 101°12.997'E, elevation ca 744 m, 1.–15.05.2007, primary tropical seasonal rain forest, trunk traps. Paratypes 1♀, 21°57.883'N, 101°12.147'E, elevation ca 839 m, 15.08.2011, primary tropical seasonal rain forest, fogging; 1♂, 21°57.445'N, 101°12.997'E, elevation ca 744 m, 16.–31.03.2007, primary tropical seasonal rain forest, trunk traps; 1♂, 21°57.445'N, 101°12.997'E, elevation ca 744 m, 16.–30.04.2007, primary tropical seasonal rain forest, trunk traps; 1♂, Xishuangbanna Tropical Botanical Garden, 21°53.993'N, 101°16.810'E, elevation ca 611 m, 19. 08.2007, *Anogeissus acuminate* plantation, fogging.

###### Etymology.

This specific name is derived from the Chinese Pinyin ‘fēng lì’, meaning ‘pointed, sharp’, in reference to the pointed proximal cymbial outgrowth of the male palp; term in apposition.

###### Diagnosis.

See diagnosis of the genus.

###### Description.

Male (holotype). Total length: 2.78. Carapace 1.16 long, 0.78 wide, post-ocular area slightly elevated, with a row of setae growing along the axis from clypeus to the fovea. Sternum 0.63 long, 0.58 wide. Clypeus 0.19 high. Eye sizes and interdistances: AME 0.07, ALE 0.11, PME 0.10, PLE 0.10, AME-AME/AME 0.43, PME-PME/PME 0.80, AME-ALE/ALE 0.36, PME-PLE/PLE 0.40, coxae IV separated by 0.95 time their width. Length of legs: I 3.71 (1.15, 0.25, 0.94, 0.78, 0.59), II 4.97 (1.40, 0.29, 1.25, 1.30, 0.73), III 3.95 (1.13, 0.28, 0.98, 1.00, 0.56), IV 4.99 (1.40, 0.28, 1.33, 1.33, 0.65). Leg formula: IV-II-III-I. TmI 0.37, TmIV 0.65. Abdomen pale, with irregular dark dots. Palp: see description of the genus.

Female (one of paratypes). Total length: 2.56. Carapace 1.00 long, 0.75 wide, dark brown. Sternum 0.60 long, 0.54 wide. Clypeus 0.20 high. Eyes sizes and interdistances: AME 0.08, ALE 0.10, PME 0.09, PLE 0.10, AME-AME/AME 0.38, PME-PME/PME 0.53, AME-ALE/ALE 0.16, PME-PLE/PLE 0.40, coxae IV separated by 1.13 times their width. Lengths of legs: I 4.98 (1.40, 0.31, 1.38, 1.18, 0.71), II 4.60 (1.30, 0.32, 1.24, 1.10, 0.64), III 3.54 (1.04, 0.20, 0.90, 0.90, 0.50), IV 4.60 (1.31, 0.31, 1.22, 1.13, 0.63). Leg formula: I-IV-II-III. TmI 0.35, TmIV 0.60. Abdomen beige, with dark green venter and symmertric patches on the dorsum. Epigyne: see description of the genus.

###### Distribution.

Known only from type localities.

##### Genus Glebala gen. n.

Taxon classificationAnimaliaAraneaeLinyphiidae

http://zoobank.org/74F7D9AA-7F2A-44F4-8FF8-4D3413B352FD

###### Type species.

*Glebala aspera* sp. n.

###### Etymology.

The generic name is an arbitrary combination of letters. Gender is feminine.

###### Diagnosis.

**Figure 36.**
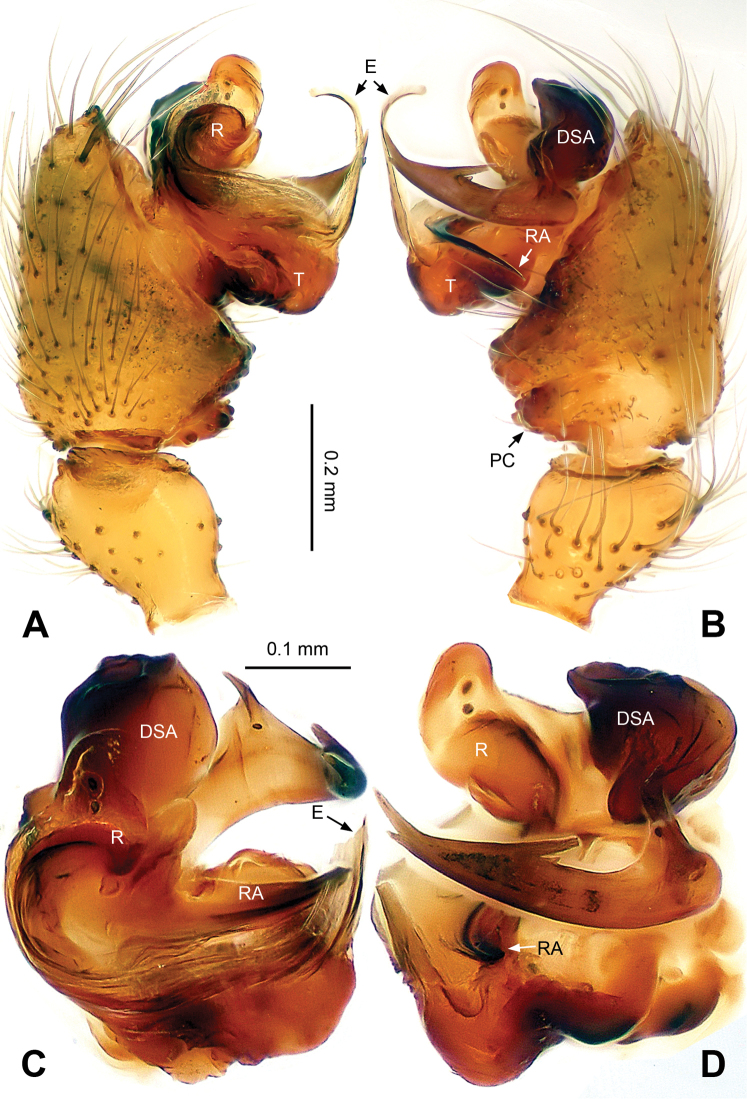
*Glebala aspera* sp. n., male holotype. **A** Right palp, prolateral view **B** Right palp, retrolateral view **C** Embolic division of right palp, prolateral view **D** Embolic division of right palp, retrolateral view. Scale bars: **A** as **B**, **C** as **D.**

This genus is unique for its knobble paracymbium (Fig. 36B), which is not known in any other linyphiid genera. The bulb is distinguished by the ambiguous delimitation of the tegulum and subtegulum (Fig. 36A).

###### Description.

Median sized Erigoninae. Male carapace slightly elevated, with a row of long hair growing along the axis. Chelicerae with 6 promarginal teeth, and 5 retromarginal teeth. Chaetotaxy: tibial spine formula: 2-2-1-1. TmI ca 0.32, TmIV ca 0.72. Abdomen with distinct pattern. Female unknown.

Male palp: tibia with two retrolateral trichobothria, with a row of setae dorsally. Cymbium hoof-like (Fig. 36A–B). Paracymbium with rough surface, strongly reduced (Fig. 36B). The bulb simple. Distal suprategular apophysis with two branches, one broad at tip, another long and curved, with bifid tip and two small processes at the mid-part (Fig. 36A–D). Radical apophysis spear-like (Fig. 36B). The embolus long, membraneous, stretching distally, with a slightly-bent, blunt-ended tip (Fig. 36A–B).

###### Species composition.

Only the type species *Glebala aspera* sp. n.

###### Distribution.

China.

##### Glebala aspera sp. n.

Taxon classificationAnimaliaAraneaeLinyphiidae

http://zoobank.org/396C198F-10CC-468D-96E1-C695A3A73C68

Figs 36 , 37 , 38

###### Types.

Holotype ♂: CHINA, Yunnan: Menglun Town: Xishuangbanna Nature Reserve, 21°57.669'N, 101°11.893'E, elevation ca 790 m, 16.–30.04.2007, primary tropical seasonal rain forest, hand-collecting.

###### Etymology.

The specific name is derived from the Latin word ‘asper’, meaning ‘rough’, in reference to the rough surface of the paracymbium; adjective.

###### Diagnosis.

See diagnosis of the genus. Abdominal pattern of this species is notably similar to that in *Gladiata fengli* sp. n.

###### Description.

**Figure 37.**
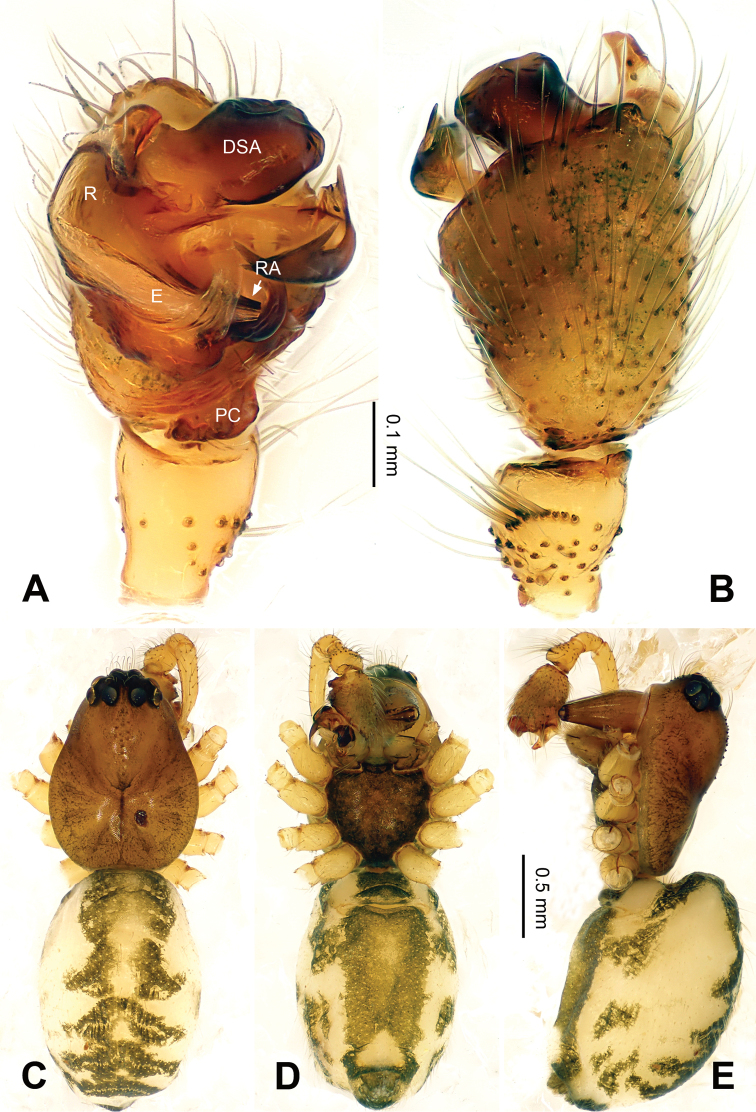
*Glebala aspera* sp. n., male holotype. **A** Right palp, ventral view **B** Right palp, dorsal view **C** Habitus, dorsal view **D** Habitus, ventral view **E** Habitus, lateral view. Scale bars: **B** as **A**, **C** and **D** as **E.**

Male (holotype). Total length: 2.64. Carapace 1.38 long, 0.90 wide, brown, with long setae growing from ocular area to the fovea (Fig. 37E). Sternum 0.55 long, 0.65 wide. Clypeus 0.24 high. Eye sizes and interdistances: AME 0.08, ALE 0.11, PME 0.10, PLE 0.12, AME-AME/AME 0.38, PME-PME/PME 0.80, AME-ALE/ALE 0.36, PME-PLE/PLE 0.42, Coxae IV separated by 1.41 times their width. Lengths of legs: I 5.62 (1.56, 0.31, 1.50, 1.41, 0.84), II 5.31 (1.48, 0.35, 1.38, 1.35, 0.75), III 4.18 (1.25, 0.28, 1.00, 1.10, 0.55), IV 5.19 (1.44, 0.28, 1.40, 1.38, 0.69). Leg formula: I-II-IV-III. Abdomen, pale, with dark ventrum, three pairs of lateral stripes and dorsal dark green folium. Palp: see description of the genus.

Female. Unknown.

###### Distribution.

Known only from type locality.

##### Genus Glomerosus gen. n.

Taxon classificationAnimaliaAraneaeLinyphiidae

http://zoobank.org/DC3B6190-92E9-400B-AF3B-C6889C084A7C

###### Type species.

*Glomerosus lateralis* sp. n.

###### Etymology.

The generic name is an arbitrary combination of letters. Gender is masculine.

###### Diagnosis.

**Figure 39.**
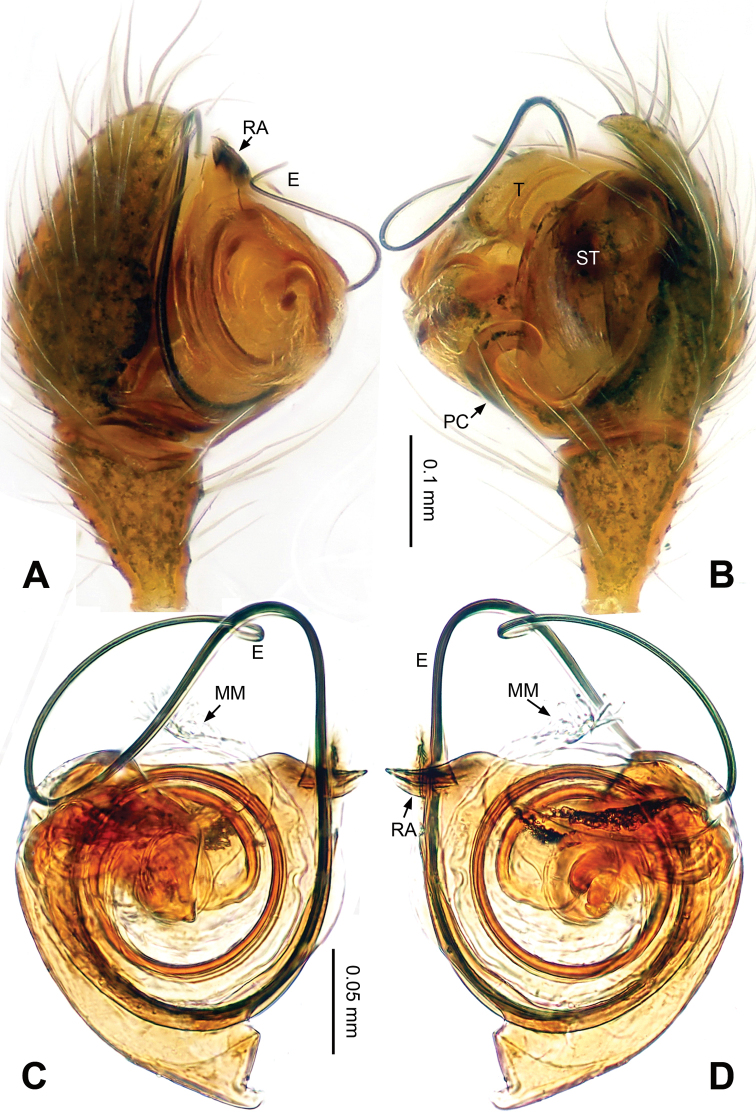
*Glomerosus lateralis* sp. n., male holotype. **A** Palp, prolateral view **B** Palp, retrolateral view **C** Embolic division, dorsal view **D** Embolic division, ventral view. Scale bars: **A** as **B**, **D** as **C.**

**Figure 40.**
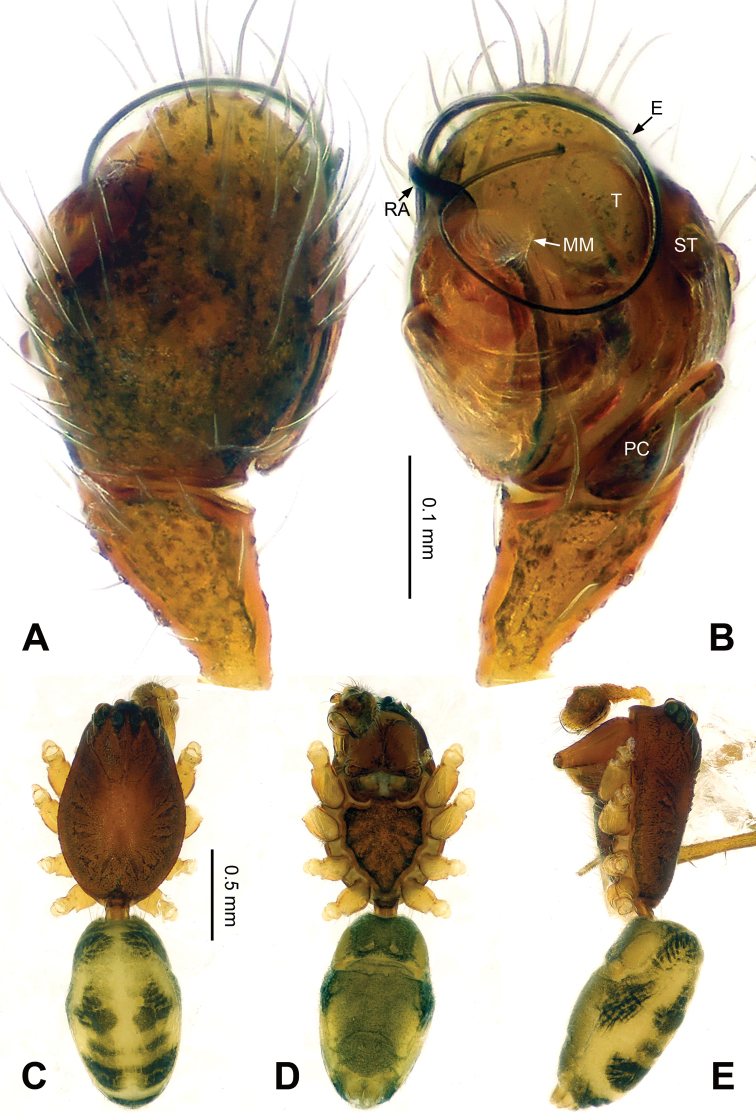
*Glomerosus lateralis* sp. n., male holotype. **A** Palp, dorsal view **B** Palp, ventral view **C** Habitus, dorsal view **D** Habitus, ventral view **E** Habitus, lateral view. Scale bars: **A** as **B**, **D** and **E** as **C.**

The genus is diagnosed by the unique palp: tibia without any apophysis (Figs 39A–B, 40A–B); bulb is globe-like (Fig. 39B); paracymbium slender, ‘J’-shaped, the base of which is closely attached to cymbium (Fig. 39B); the embolus is similar to that in genus *Plectembolus* Millidge & Russell-Smith, 1992 and *Plicatiductus* Millidge & Russell-Smith 1992 known from Southeast Asia, but *Glomerosus* gen. n. has fewer coils and a whip-like, loose tip (Fig. 39D). The embolic division has a less sclerotised terminal apophysis, which is small with an attenuated tip (Fig. 39A).

###### Description.

Body elongate. Male carapace long and narrow (Fig. 40C–E), unmodified. Chelicerae with 2 promarginal teeth, and 1 retromarginal teeth. Legs long and slender. Chaetotaxy: tibial spine formula: 2-2-2-2. TmI ca 0.17, TmIV absent. Female unknown. Abdomen with dark green markings against yellow background on dorsum, venter grayish green.

**Figure 41.**
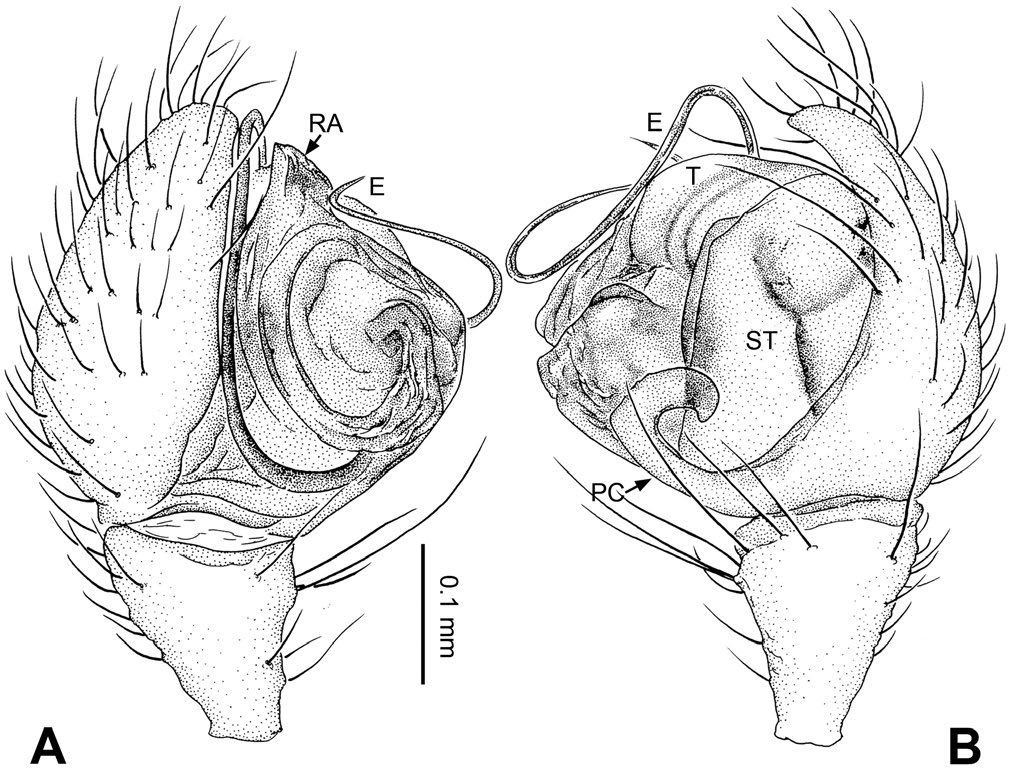
*Glomerosus lateralis* sp. n., male holotype. **A** Palp, prolateral view **B** Palp, retrolateral view. Scale bar: **B** as **A.**

Male palp: tibia unmodified, with one retrolateral trichobothrium and one dorsal trichobothrium; paracymbium ‘J’-shaped, with a curved apex (Fig. 39B); median membrane small piece, with fringed rim (Figs 39D, 40B); radical apophysis short, erect (Fig. 40B); radix slender and curved (Fig. 41C); embolus long, whip-like, coiled at the prolateral side of bulb (Fig. 39A, C).

###### Species composition.

Only the type species *Glomerosus lateralis* sp. n.

###### Distribution.

China.

##### Glomerosus lateralis sp. n.

Taxon classificationAnimaliaAraneaeLinyphiidae

http://zoobank.org/6DD3655D-4D69-4E32-99E4-6F394816705C

Figs 39 , 40 , 41

###### Types.

Holotype ♂: CHINA, Yunnan: Menglun Town: Xishuangbanna Tropical Botanical Garden, 21°55.035'N, 101°16.500'E, elevation ca 558 m, 10.–20.07.2007, primary tropical seasonal rain forest, hand-collecting.

###### Etymology.

The species name is taken from the Latin word ‘lateralis’, meaning ‘of the side, lateral’, in reference to the position of the coiled embolus; adjective.

###### Diagnosis.

See diagnosis of the genus.

###### Description.

Male (holotype). Total length: 2.23. Carapace 1.13 long, 0.67 wide, reddish brown. Sternum 0.47 long, 0.39 wide. Clypeus 0.20 high. Eye sizes and interdistances: AME 0.06, ALE 0.09, PME 0.08, PLE 0.08, AME-AME/AME 0.17, PME-PME/PME 0.38, AME-ALE/ALE 0.11, PME-PLE/PLE 0.50, coxae IV separated by 0.86 time their width. Length of legs: I 4.97 (1.30, 0.25, 1.36, 1.28, 0.78), II 4.45 (1.17, 0.25, 1.16, 1.09, 0.70), III 2.63 (0.75, 0.20, 0.65, 0.60, 0.45), IV 3.35 (0.88, 0.19, 0.88, 0.85, 0.55). Leg formula: I-II-IV-III. Abdomen yellow, with about four pairs of dark green markings of different sizes on the dorsum, a small patch of the same color at the posterior tip of abdomen. Palp: see description of the genus.

Female. Unknown.

###### Distribution.

Known only from type locality.

#### Genus *Gongylidiellum* Simon, 1884

*Gongylidiellum*: Simon 1884: 605. Type species: *Neriene latebricola* O. P.-Cambridge, 1871.

##### Gongylidiellum bracteatum sp. n.

Taxon classificationAnimaliaAraneaeLinyphiidae

http://zoobank.org/7B82A84F-50A2-44D8-87DB-3DC4ACBEBF45

Figs 42 , 43

###### Types.

Holotype ♀: CHINA, Yunnan: Menghai County: Manguan Village: Xishuangbanna nature reserve, 22°01.772'N, 100°23.711'E, elevation ca 1187 m, 1.07.2013, secondary forest, hand-collecting.

###### Etymology.

This name comes from the Latin word ‘bracteatus’, which means ‘covered by scale, small fig’, in reference to the scale-shaped structure on the ventral fig; adjective.

###### Diagnosis.

**Figure 42.**
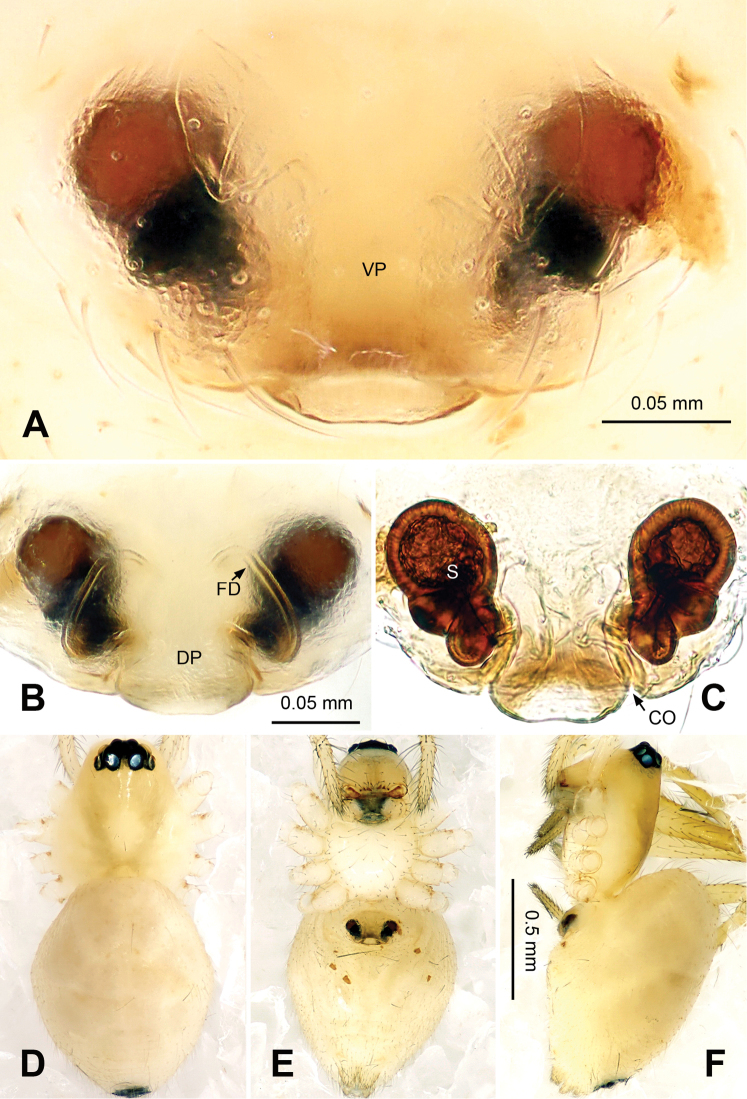
*Gongylidiellum bracteatum* sp. n., female holotype. **A** Epigyne, ventral view **B** Epigyne, dorsal view **C** Vulva, dorsal view **D** Habitus, dorsal view **E** Habitus, ventral view **F** Habitus lateral view. Scale bars: **C** as **B**, **D** and **E** as **F.**

It is similar to *Gongylidiellum latebricola* (O. P.-Cambridge, 1871) by the shape of epigyne and the conformation of vulva (Roberts 1987: 80, fig. 34c; Tanasevitch 1990: 111, fig. 24.40); its ventral fig is equipped with a pair of small membraneous extensions (Fig. 42A), which couldn’t be seen in *Gongylidiellum latebricola*.

###### Description.

**Figure 43.**
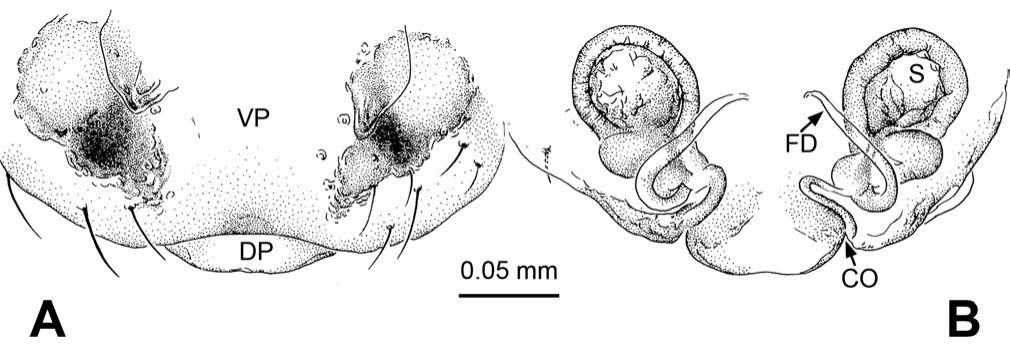
*Gongylidiellum bracteatum* sp. n., female holotype. **A** Epigyne, ventral view **B** Vulva, dorsal view Scale bar: **B** as **A.**

Female (holotype). Total length: 1.48. Carapace 0.70 long, 0.56 wide, light yellow. Sternum 0.31 long, 0.39 wide. Clypeus 0.13 high. Chelicerae promargin with 3 teeth, retromargin with 2 teeth. Eye sizes and interdistances: AME 0.04, ALE 0.06, PME 0.05, PLE 0.06, AME-AME/AME 0.75, PME-PME/PME 1.00, AME-ALE/ALE 0.17, PME-PLE/PLE 0.33, coxae IV separated by 1.64 times their width. Length of legs: I 2.13 (0.61, 0.20, 0.53, 0.48, 0.31), II 2.26 (0.64, 0.19, 0.56, 0.53, 0.34), III 1.65 (0.45, 0.17, 0.36, 0.41, 0.26), IV 2.16 (0.64, 0.17, 0.50, 0.54, 0.31). Leg formula: II-IV-I-III. TmI 0.65, TmIV 0.63. Tibial spine formula: 1-1-1-1. Abdomen pale, with a black posterior tip. Epigyne: ventral fig half rounded (Figs 42A, 43A); dorsal fig with a curved posterior margin; copulatory ducts make two turnings before joining the spermathecae (Fig. 42C); spermathecae rounded, separated by 1.5 their diameter (Fig. 42C); fertilization ducts long, following a ‘C’-shaped route (Fig. 43B).

Male. Unknown.

###### Distribution.

Known only from type locality.

#### Genus *Houshenzinus* Tanasevitch, 2006

*Houshenzinus*: Tanasevitch 2006b: 292. Type species *Houshenzinus rimosus* Tanasevitch, 2006 from China.

##### Houshenzinus xiaolongha sp. n.

Taxon classificationAnimaliaAraneaeLinyphiidae

http://zoobank.org/F6411604-37DC-417D-86C5-C5F9CE2550CD

Figs 44 , 45 , 46

###### Types.

Holotype ♂, CHINA, Yunnan: Mengla County: Xishuangbanna Nature Reserve, Xiaolongha biodiversity preservation corridor, 21°24.265'N, 101°37.296'E, elevation ca 653 m, 27.06.2012, valley evergreen rain forest, sieving. Paratypes 1♂2♀, same data as holotype.

###### Etymology.

This species’s name is derived from the Chinese Pinyin ‘xiǎo lóng hā’, which is the type locality of this species; term in apposition.

###### Diagnosis.

**Figure 44.**
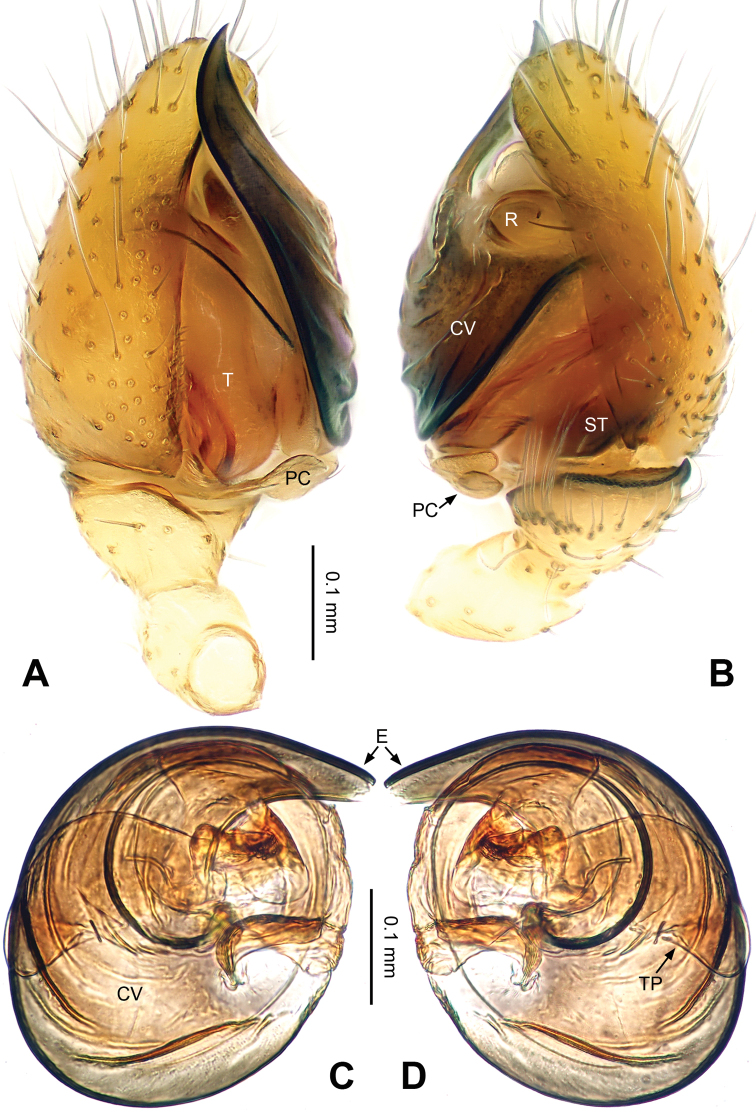
*Houshenzinus xiaolongha* sp. n., male holotype. **A** Palp, prolateral view **B** Palp, retrolateral view **C** Embolic division, ventral view **D** Embolic division, dorsal view. Scale bar: **B** as **A**, **C** as **D.**

**Figure 45.**
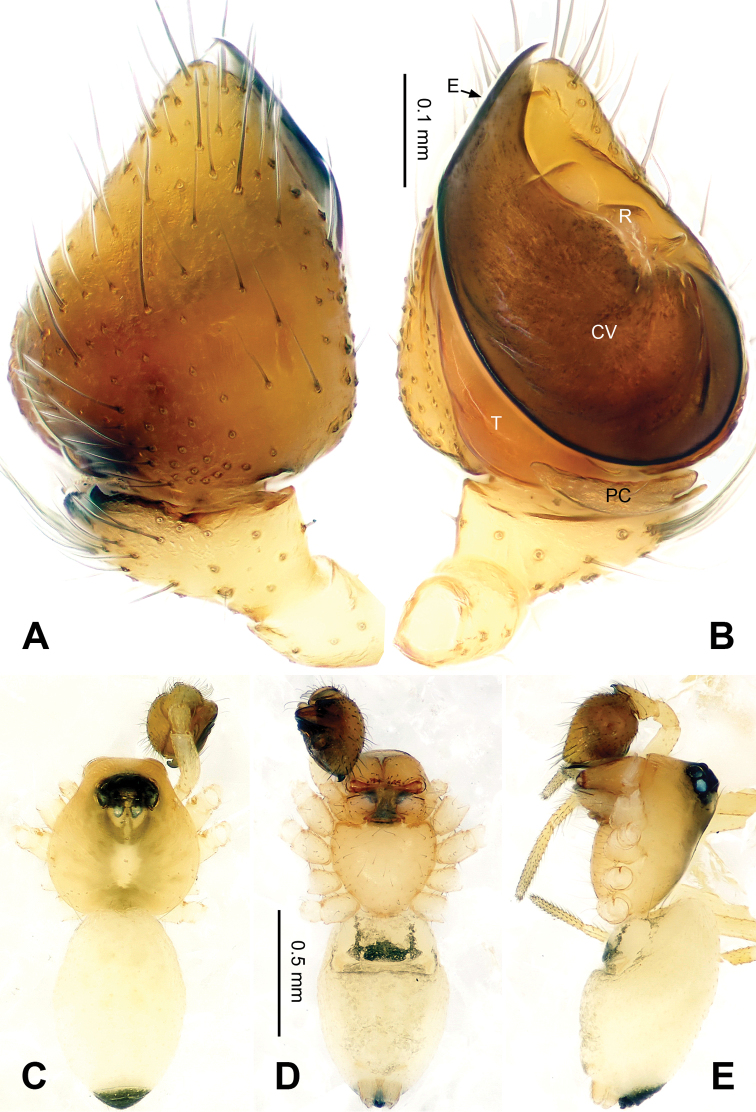
*Houshenzinus xiaolongha* sp. n., male holotype. **A** Palp, doral view **B** Palp, ventral view **C** Habitus, dorsal view **D** Habitus, ventral view **E** Habitus, lateral view. Scale bars: **A** as **B**, **C** and **E** as **D.**

**Figure 46.**
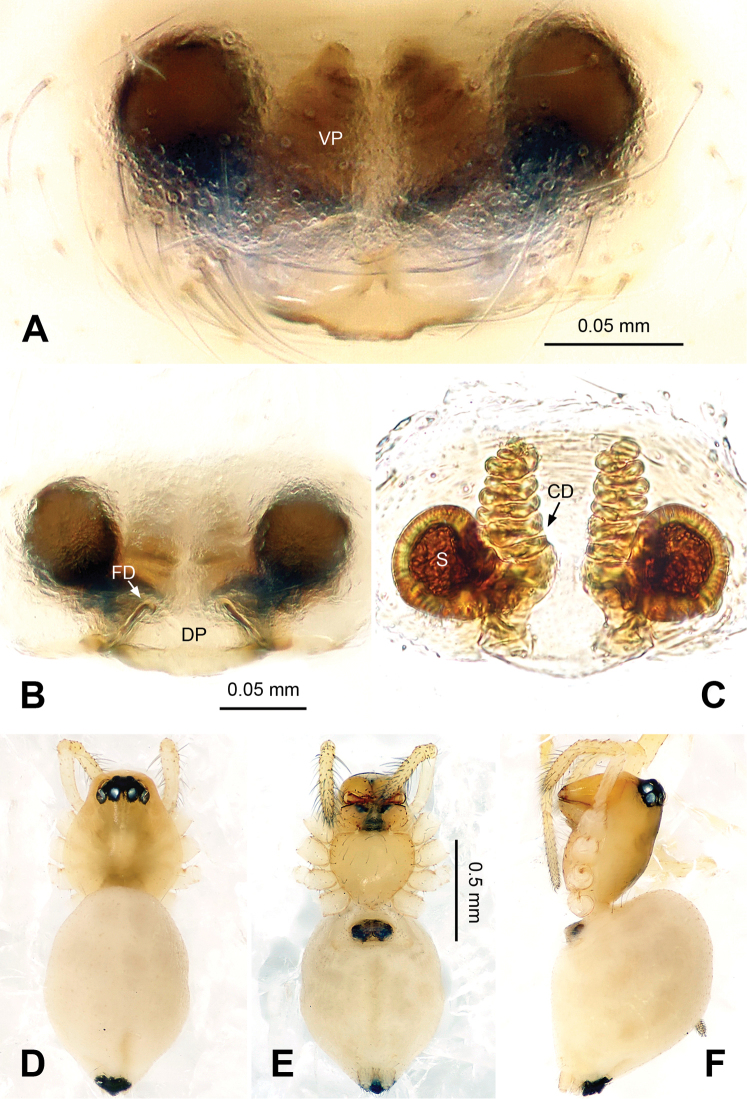
*Houshenzinus xiaolongha* sp. n., female paratype. **A** Epigyne, ventral view **B** Epigyne, dorsal view **C** Vulva, dorsal view **D** Habitus, dorsal view **E** Habitus, ventral view **F** Habitus lateral view. Scale bars: **C** as **B**, **D** and **F** as **E.**

This species is distinguished by the shape of convector in male (Fig. 45B; Tanasevitch 2006b: figs 45–46) and the loops of copulatory ducts in female’s epigyne (Fig. 46C; Song and Li 2008: fig. 287), but differs from the type species by the inconspicuous dorsal tibial apophysis (Fig. 45A) and ridges on convector in male’s palp (Fig. 44A–B), and three more loops than the copulatory ducts make in female’s epigyne.

###### Description.

Male (holotype). Total length: 1.40. Carapace 0.63 long, 0.58 wide, yellow. Sternum 0.39 long, 0.56 wide. Clypeus 0.17 high. Chelicerae promargin with 3 teeth, retromargin with 3 teeth. Eye sizes and interdistances: AME 0.05, ALE 0.06, PME 0.08, PLE 0.08, AME-AME/AME 0.56, PME-PME/PME 0.60, AME-ALE/ALE 0.33, PME-PLE/PLE 0.50, coxae IV separated by 2.00 times their width. Length of legs: I 2.34 (0.63, 0.22, 0.58, 0.50, 0.41), II 2.33 (0.63, 0.20, 0.58, 0.53, 0.39), III 1.81 (0.50, 0.19, 0.39, 0.42, 0.31), IV 2.69 (0.63, 0.19, 0.53, 0.54, 0.35). Leg formula: I-II-IV-III. Tm I 0.63, Tm IV 0.59. Tibial spine formula: 2-2-1-1. Abdomen pale, with a black posterior end. Palp: tibia equipped with two retrolateral trichobothria, one dorsal trichobothrium, and a small dorsal apophysis (Fig. 45A). Paracymbium ‘J’-shaped, with a bifurcate tip (Fig. 44B). Tailpiece tongue-shaped when viewed dorsally (Fig. 44D). Convector prominent, comma-shaped, covering most part of the embolic division (Fig. 45B). Embolus thread-like, coiled, stretching along the outer margin of distal suprategular apophysis (Fig. 44B).

Female (one of paratypes). Total length: 1.60. Carapace 0.60 long, 0.56 wide, unmodified, same as male in coloration. Sternum 0.32 long, 0.40 wide. Clypeus 0.16 high. Chelicerae promargin with 6 teeth, retromargin with 3 teeth. Eye sizes and interdistances: AME 0.05, ALE 0.08, PME 0.06, PLE 0.07, AME-AME/AME 0.63, PME-PME/PME 0.52, AME-ALE/ALE 0.23, PME-PLE/PLE 0.38, coxae IV separated by 2.00 times their width. Length of legs: I 2.32 (0.63, 0.20, 0.58, 0.50, 0.41), II 2.34 (0.64, 0.20, 0.57, 0.55, 0.38), III 1.80 (0.50, 0.18, 0.38, 0.43, 0.31), IV 2.36 (0.63, 0.23, 0.61, 0.55, 0.33). Leg formula: IV-II-I-III. Tm I 0.71, Tm IV 0.68. Tibial spine formula: 2-2-1-1. Abdomen light pink, with a black tip. Epigyne: ventral fig semi-transparent, mesally concave at the posterior rim (Fig. 46A); copulatory ducts long, forming about five loops before joining spermathecae (Fig. 46C); spermathecae oval, separated by 1.5 times their diameter (Fig. 46B).

###### Distribution.

Known only from type locality.

#### Genus *Hylyphantes* Simon, 1884

*Hylyphantes*: Simon 1884: 464. Type species *Erigone nigrita* Simon, 1881.

##### Hylyphantes graminicola

Taxon classificationAnimaliaAraneaeLinyphiidae

(Sundevall, 1830)

Linyphia graminicola : Sundevall 1830: 26 (♂♀).
Hylyphantes graminicola : Roberts 1987: 42, fig. 12b (♂♀).

###### Material examined.

1♂, CHINA, Yunnan: Mengla County: Menglun Town: Xishuangbanna Tropical Botanical Garden, 21°54.565'N, 101°16.021'E, elevation ca 584 m, 21.VIII.2011, fogging.

###### Distribution.

Palearctic and SE Asia.

#### Genus *Kaestneria* Wiehle, 1956

*Kaestneria*: Wiehle 1956: 272. Type species *Linyphia dorsalis* Wider, 1834.

##### Kaestneria bicultrata

Taxon classificationAnimaliaAraneaeLinyphiidae

Chen & Yin, 2000

Figs 47 , 48 , 49

Kaestneria bicultrata : Chen and Yin 2000: 88, figs 17–20 (♀).

###### Material examined.

1♂, CHINA, Yunnan: Menglun Town: Xishuangbanna Nature Reserve, 21°54.718'N, 101°16.940'E, elevation ca 645 m, 5.–12.01.2007, secondary tropical seasonal rain forest, hand-collecting; 1♂, 21°54.646'N, 101°16.257'E, elevation ca 572 m, 5.–12.02.2007, rubber-tea plantation, hand-collecting; 1♂, 21°54.463'N, 101°15.978'E, elevation ca 569 m, 19.–25.02.2007, rubber-tea plantation, hand-collecting; 1♂1♀, 21°54.646'N, 101°16.257'E, elevation ca 572 m, 19.–26.05.2007, rubber-tea plantation, hand-collecting.

###### Diagnosis.

**Figure 47.**
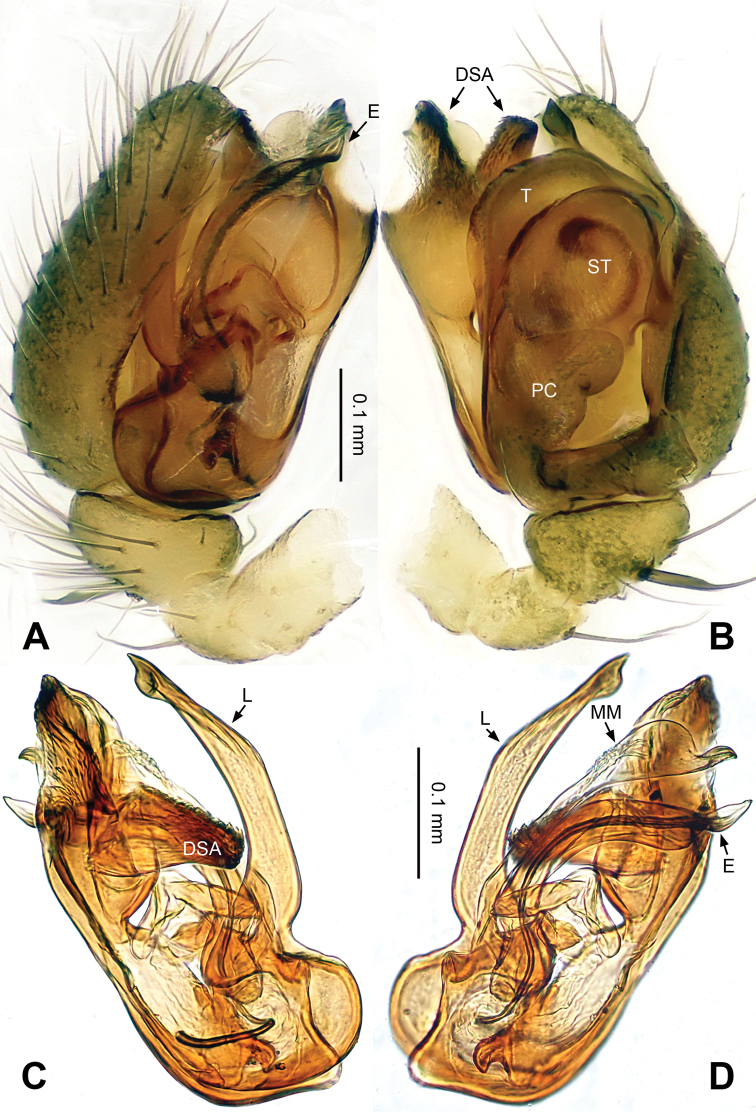
*Kaestneria bicultrata.* **A** Palp, prolateral view **B** Palp, retrolateral view **C** Embolic division, retrolateral view **D** Embolic division, prolateral view. Scale bars: **B** as **A**, **C** as **D.**

The female is easily recognized by the copulatory duct route and the finger-like spines on each side of the ventral fig (Chen and Yin 2000: figs 18–19). The male is similar to *Kaestneria pullata* O. P.-Cambridge, 1863 (Roberts 1987: fig. 71b) and *Kaestneria minima* Locket, 1982, but differs by the shape of paracymbium and embolic division (Fig. 47B, D). Both male and female differ from other congeners by having pattern on carapace and abdomen.

###### Description.

Female. Well described, e.g. by Chen and Yin (2000).

**Figure 48.**
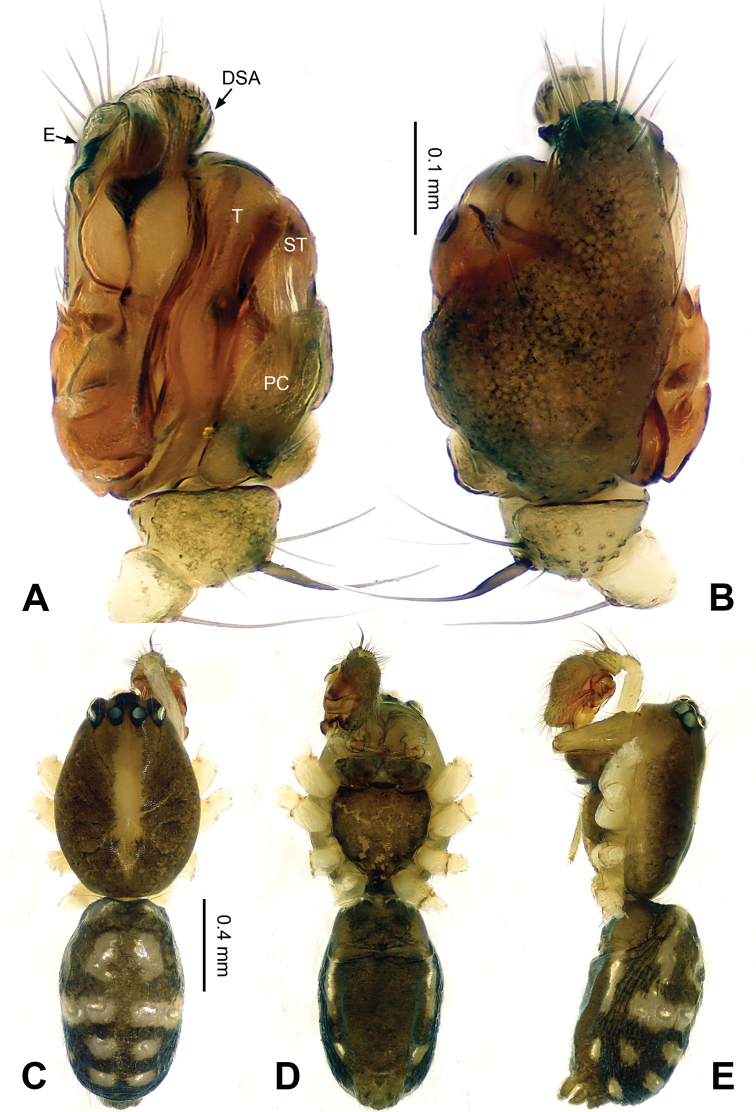
*Kaestneria bicultrata*. **A** Palp, ventral view **B** Palp, dorsal view **C** Habitus, dorsal view **D** Habitus, ventral view **E** Habitus, lateral view. Scale bars: **A** as **B**, **D** and **E** as **C.**

**Figure 49.**
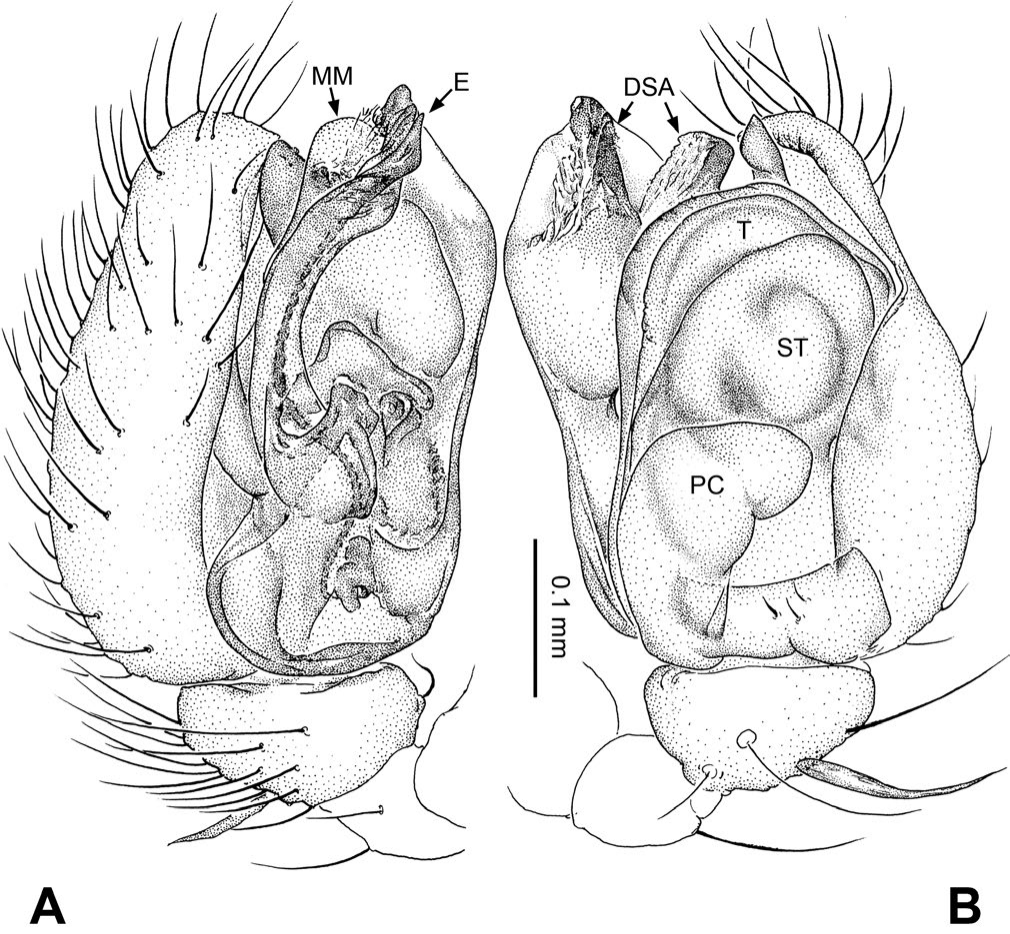
*Kaestneria bicultrata*. **A** Palp, prolateral view **B** Palp, retrolateral view. Scale bar: **A** as **B.**

Male (one of males from Xishuangbanna). Total length: 1.78. Carapace 0.94 long, 0.64 wide, brown, with a pale stripe along the axis. Sternum 0.50 long, 0.45 wide. Clypeus 0.15 high. Chelicerae promargin with no teeth, retromargin with 1 tooth. Eye sizes and interdistances: AME 0.05, ALE 0.08, PME 0.07, PLE 0.06, AME-AME/AME 0.60, PME-PME/PME 0.43, AME-ALE/ALE 0.63, PME-PLE/PLE 0.67, coxae IV separated by 0.60 time their width. Lengths of legs: I 5.00 (1.30, 0.24, 1.28, 1.38, 0.80), II 3.14 (1.13, 0.20, 1.04, 1.13, 0.64), III 2.63 (0.76, 0.18, 0.56, 0.70, 0.43), IV 3.73 (1.10, 0.19, 0.88, 1.03, 0.53). Leg formula: I-IV-II-III. TmI 0.15, TmIV absent. Tibial spine formula: 2-2-2-2. Abdomen greenish brown, with white patches, same as the pattern in female holotype (Chen and Yin 2000: fig. 17). Palp: tibia small, with one long, thick dorsal setae (Figs 47B, 48A–B); paracymbium ‘V’-shaped, basally somewhat rectangular, with a broad semi-circular tip (Fig. 47B); distal suprategular apophysis long, with a bifid apex, each embranchment blunt-ended, with rough surface (Figs 47B, 49B); lamella long and erect, terminating in a finger-like end (Fig. 47C–D); median membrane long, broad, abruptly produced into a hooked tip (Fig. 47D); embolus simple, ribbon-like with a pointed tip (Fig. 47D).

###### Distribution.

China.

###### Remarks.

Male of the species is reported for the first time.

#### Genus *Laogone* Tanasevitch, 2014

*Laogone*: Tanasevitch 2014b: 76. Type species *Laogone cephala* Tanasevitch, 2014 from Laos.

##### Laogone bai sp. n.

Taxon classificationAnimaliaAraneaeLinyphiidae

http://zoobank.org/188350EA-3A2B-44E6-A0DC-C6B0C6C3ACA5

Figs 50 , 51

###### Types.

Holotype ♂, CHINA, Yunnan: Meng’a Town: Wengnan village: Xishuangbanna Nature Reserve, 22°05.002'N, 100°22.009'E, elevation ca 1118 m, 30.07.2007, secondary seasonal rain forest, fogging.

###### Etymology.

This species’s name is derived from the Chinese Pinyin ‘bái’, meaning ‘white’, which refers to the color of the holotype’s body; term in apposition.

###### Diagnosis.

This species is similar to the type species *Laogone cephala*, but differs by the long and pointed dorsal tibial process, and the slimmer membrosclerum.

###### Description.

**Figure 50.**
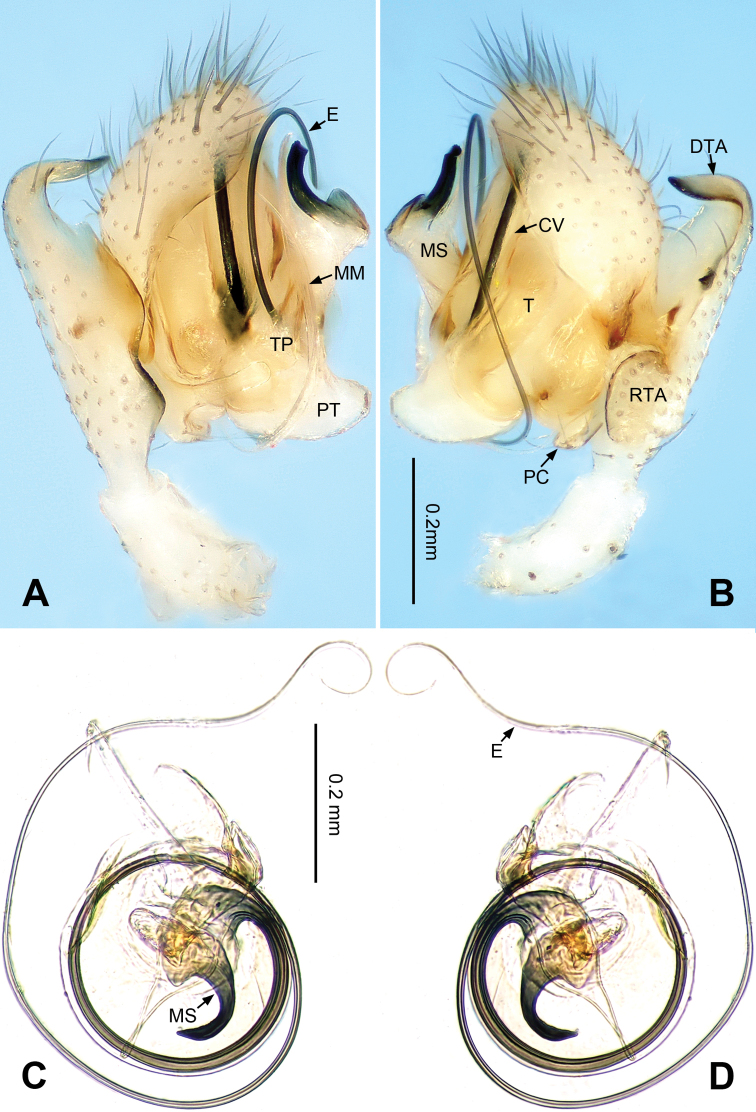
*Laogone bai* sp. n., male holotype. **A** Palp, prolateral view **B** Palp, retrolateral view **C** Embolic division, dorsal view **D** Embolic division, ventral view. Scale bars: **A** as **B**, **D** as **C.**

**Figure 51.**
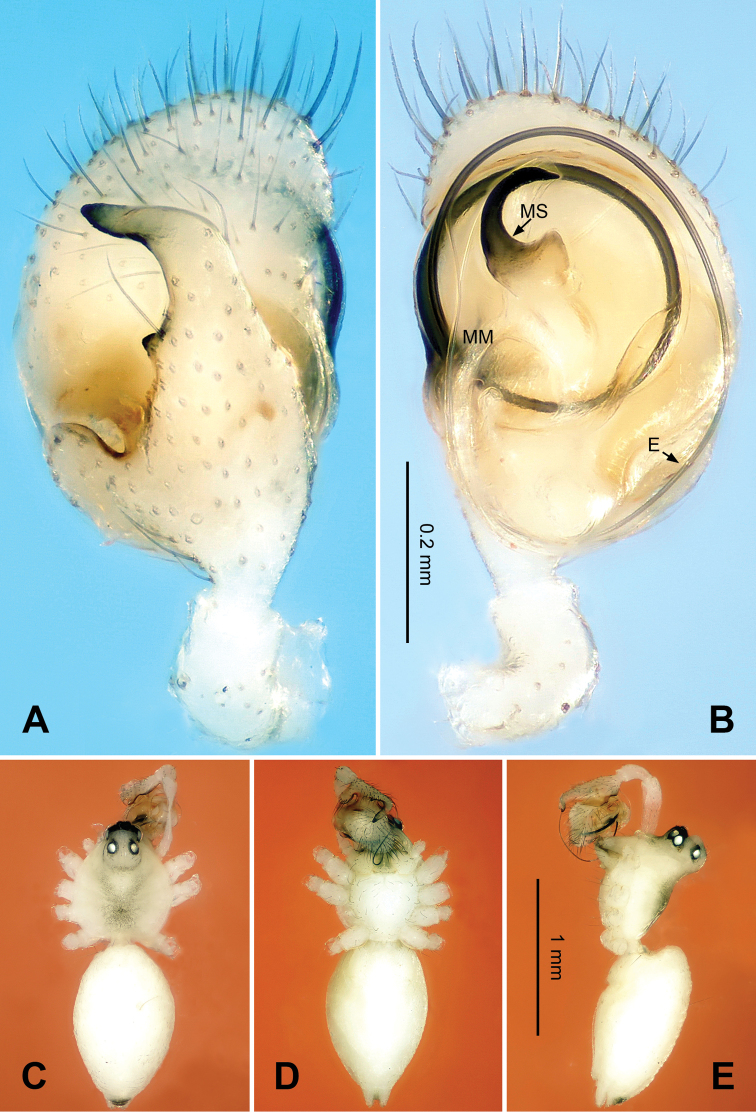
*Laogone bai* sp. n., male holotype. **A** Palp, dorsal view **B** Palp, ventral view **C** Habitus, dorsal view **D** Habitus, ventral view **E** Habitus, lateral view. Scale bars: **A** as **B**, **C** and **D** as **E.**

Male (holotype). Total length: 1.75. Carapace 0.75 long, 0.63 wide, pale, elevated into a lobe carrying posterior eyes. Sternum 0.44 long, 0.44 wide. Clypeus 0.16 high. Chelicerae promargin with 4 teeth, retromargin with 3 teeth. Eye sizes and interdistances: AME 0.04, ALE 0.06, PME 0.05, PLE 0.06, AME-AME/AME 0.40, PME-PME/PME 1.23, AME-ALE/ALE 0.38, PME-PLE/PLE 1.02, coxae IV separated by 1.58 times their width. Length of legs: I 3.34 (0.81, 0.25, 0.94, 0.86, 0.48), II 3.40 (0.88, 0.20, 0.90, 0.86, 0.56), III 2.50 (0.70, 0.25, 0.50, 0.66, 0.39), IV 3.05 (0.80, 0.20, 0.80, 0.80, 0.45). Leg formula: II-I-IV-III. Tm I 0.80, Tm IV 0.79. Tibial spine formula: 2-2-1-1. Abdomen pale, with a black tip near spinnerets. Palp: retrolateral tibial apophysis lobe-like, with a row of spines along the margin (Fig. 50B,), dorsal tibial apophysis long, ‘7’-shaped, with a pointed tip (Fig. 51A). Paracymbium with a process (Fig. 50A). Convector present. Membrosclerum ‘C’ -shaped in ventral view (Fig. 51B), slim and attenuated at distal end; median membrane long and narrow, stretching along the proximal part of embolic division (Fig. 50A–B). Embolus whip-like, forming two loops (Fig. 51B).

Female. Unknown.

###### Distribution.

Known only from type locality.

##### Laogone lunata sp. n.

Taxon classificationAnimaliaAraneaeLinyphiidae

http://zoobank.org/2364FCE7-0ED6-416E-81E3-6E59CF3ABFEC

**Figure 52.**
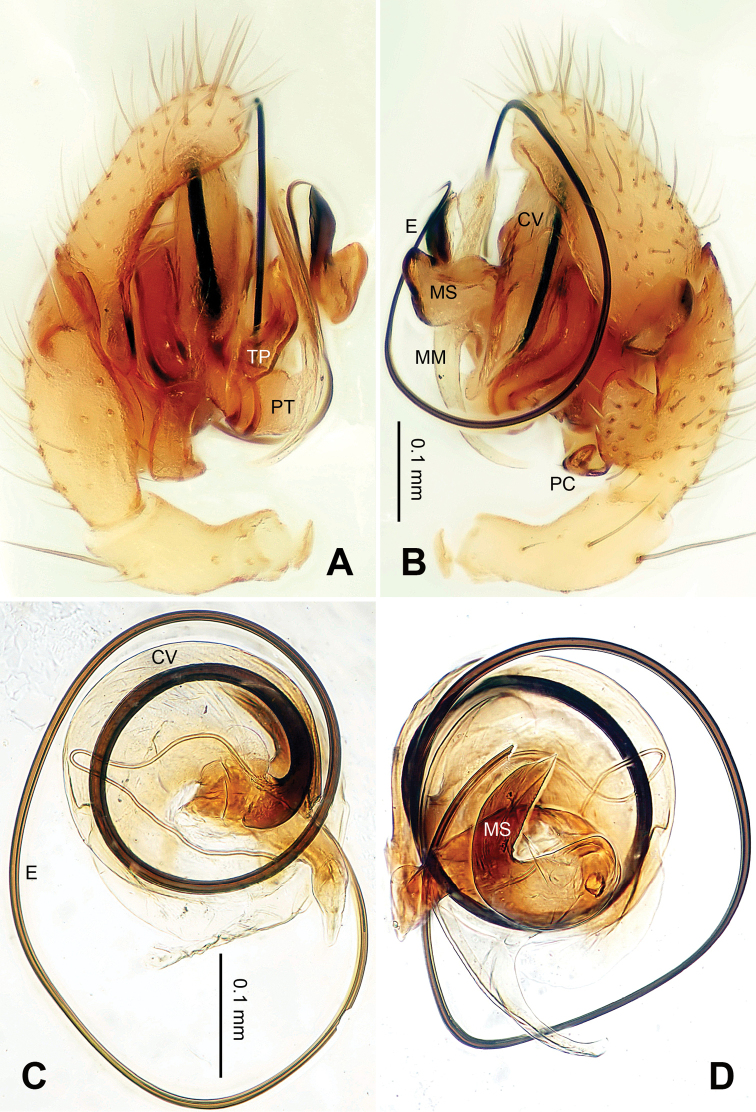
*Laogone lunata* sp. n., male holotype. **A** Palp, prolateral view **B** Palp, retrolateral view **C** Embolic division, dorsal view **D** Embolic division, ventral view. Scale bars: **A** as **B**, **D** as **C.**

**Figure 53.**
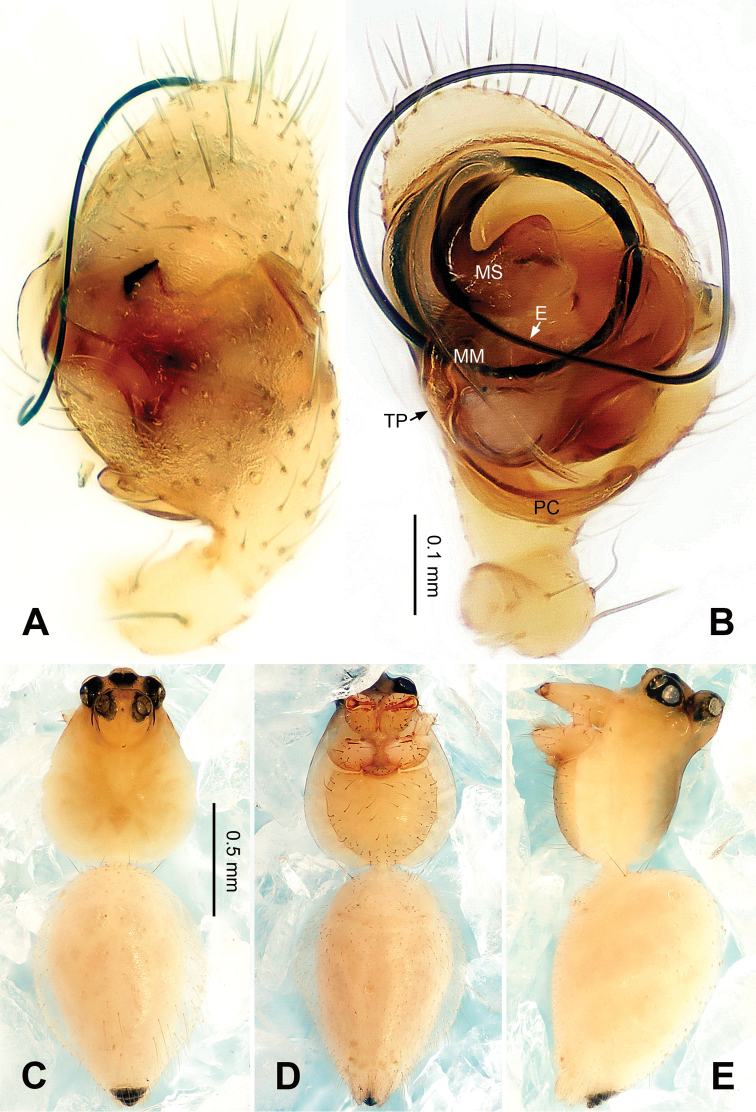
*Laogone lunata* sp. n., male holotype. **A** Palp, dorsal view **B** Palp, ventral view **C** Habitus, dorsal view **D** Habitus, ventral view **E** Habitus, lateral view. Scale bars: **A** as **B**, **D** and **E** as **C.**

**Figure 54.**
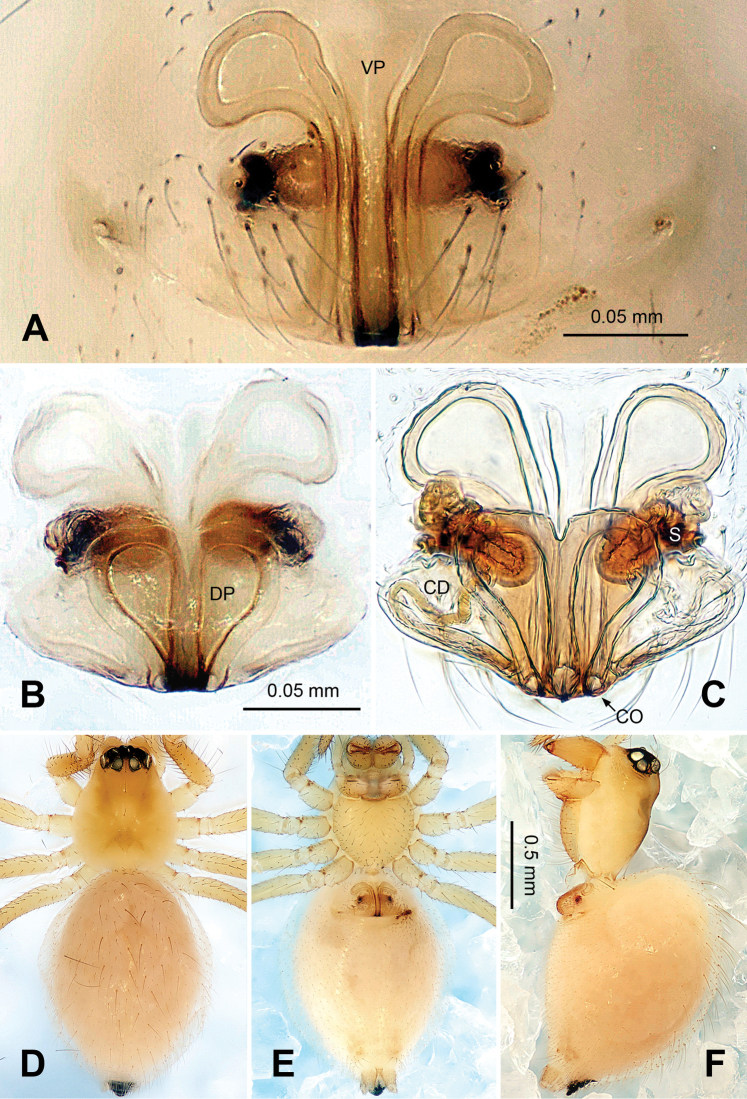
*Laogone lunata* sp. n., female paratype. **A** Epigyne, ventral view **B** Epigyne, dorsal view **C** Vulva, dorsal view **D** Habitus, dorsal view **E** Habitus, ventral view **F** Habitus lateral view. Scale bars: **C** as **B**, **D** and **E** as **F.**

**Figure 55.**
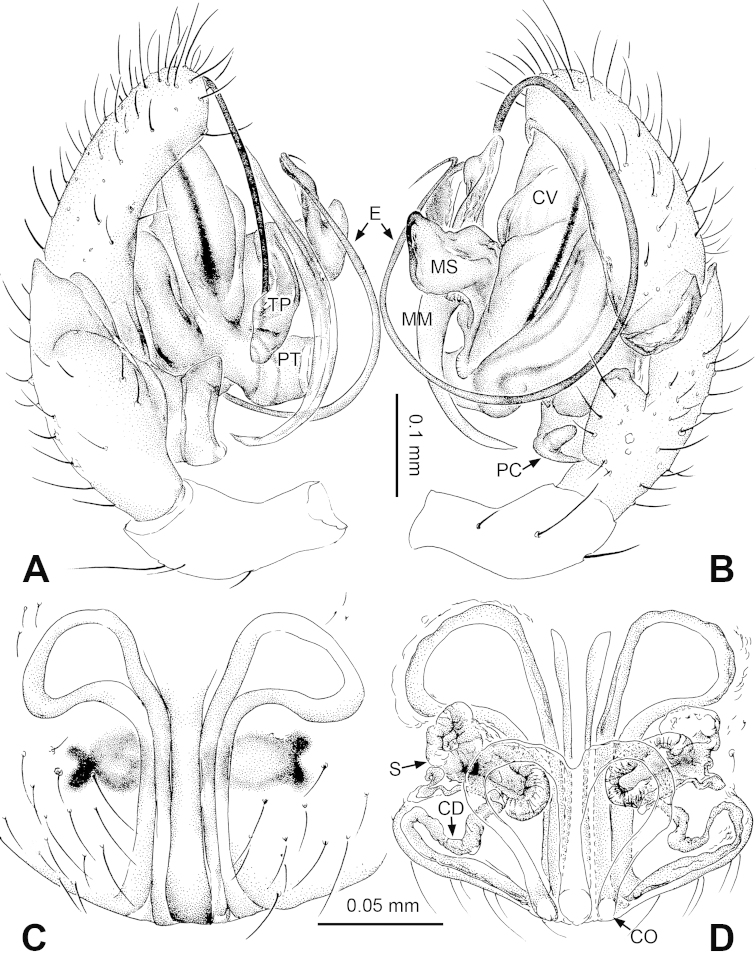
*Laogone lunata* sp. n., male holotype (**A–B**) and female paratype (**C–D**). **A** Palp, prolateral view **B** Palp, retrolateral view **C** Epigyne, ventral view **D** Vulva, dorsal view. Scale bars: **A** as **B**, **C** as **D.**

Figs 52 , 53 , 54 , 55

###### Types.

Holotype ♂, CHINA, Yunnan: Menglun Town: Xishuangbanna Nature Reserve, 21°57.445'N, 101°12.997'E, elevation ca 744 m, 30.07.2007, primary tropical seasonal rain forest, fogging. Paratypes 8♂18♀, same data as holotype; 1♂3♀, 21°54.607'N, 101°17.005'E, elevation ca 633 m, 5.–12.03.2007, secondary tropical seasonal moist forest, hand-collecting; 6♂4♀, 21°54.607'N, 101°17.005'E, elevation ca 633 m, 28.07.2007, secondary tropical seasonal moist forest, fogging; 23♂24♀, 21°57.669'N, 101°11.893'E, elevation ca 790 m, 7.08.2007, primary tropical seasonal rain forest, fogging; 3♂1♀, 21°54.984'N, 101°16.982'E, elevation ca 656 m, 10.08.2007, secondary tropical seasonal moist forest, fogging; 1♂8♀, 21°54.705'N, 101°16.898'E, elevation ca 664 m, 15.11.2009, Lvshilin tropical rain forest, fogging; 31♂29♀, 21°54.609'N, 101°17.090'E, elevation ca 643 m, 17.11.2009, Lvshilin tropical rain forest, fogging; 10♂35♀, 21°54.600'N, 101°17.084'E, elevation ca 640 m, 17.11.2009, Lvshilin tropical rain forest, fogging.

###### Etymology.

The name comes from the Latin word ‘lunatus’, meaning ‘shaped like a crescent moon’, which refers to the shape of the cymbium in lateral view; adjective.

###### Diagnosis.

This species is similar to the type species *Laogone cephala* (Tanasevitch 2014b: figs 28, 30–32; Fig. 52B), but differs by the short dorsal tibial apophysis, and the shape of membrosclerum.

###### Description.

Male (holotype). Total length: 1.90. Carapace 0.77 long, 0.56 wide, yellow, faded at the outer margin. Sternum 0.38 long, 0.44 wide. Clypeus 0.08 high. Chelicerae promargin with 4 teeth, retromargin with 3 teeth. Eye sizes and interdistances: AME 0.04, ALE 0.10, PME 0.09, PLE 0.08, AME-AME/AME 0.39, PME-PME/PME 1.25, AME-ALE/ALE 0.40, PME-PLE/PLE 1.04, coxae IV separated by 1.67 times their width. Length of legs: I 2.76 (0.67, 0.23, 0.70, 0.69, 0.47), II 2.79 (0.70, 0.23, 0.70, 0.69, 0.47), III 2.13 (0.55, 0.22, 0.47, 0.55, 0.34), IV 2.69 (0.78, 0.20, 0.63, 0.69, 0.39). Leg formula: II-I-IV-III. Tm I 0.79, Tm IV 0.73. Patellar spine formula: 2-2-2-2. Abdomen pale, with a black tip near spinnerets. Palp: patella elongate; tibia with two retrolateral and one dorsal trichobothria. Retroventral and retrodorsal apophyses both short and broad (Figs 52B, 53A). Cymbium crescent-moon shaped in lateral view (Fig. 52A–B). Paracymbium mostly concealed under bulb (Figs 52B, 53B), with one small pointed tip (Figs 52B, 53B). Convector flat, round in ventral view. Membrosclerum comma-shaped from ventral view (Fig. 53B); tailpiece small, tongue-shaped when viewed prolaterally (Fig. 52B); median membrane ribbon-like, with a tapering tip (Figs 52B, 53B). Loose part of embolus thread-like, forming one big loop, the enveloped part of embolus in convector forming small loop (Fig. 53B).

Female (one of paratypes). Total length: 1.97. Carapace 0.78 long, 0.59 wide, unmodified, same as male in coloration. Sternum 0.41 long, 0.44 wide. Clypeus 0.14 high. Chelicerae promargin with 5 teeth, retromargin with 5 teeth. Eye sizes and interdistances: AME 0.04, ALE 0.09, PME 0.08, PLE 0.08, AME-AME/AME 0.25, PME-PME/PME 0.50, AME-ALE/ALE 0.22, PME-PLE/PLE 0.38, coxae IV separated by 1.46 times their width. Length of legs: I 2.54 (0.70, 0.23, 0.63, 0.59, 0.39), II 2.54 (0.70, 0.23, 0.63, 0.59, 0.39), III 2.02 (0.55, 0.22, 0.47, 0.47, 0.31), IV 2.42 (0.63, 0.23, 0.61, 0.56, 0.39). Leg formula: II-I-IV-III. Tm I 0.76, Tm IV 0.89. Patellar spine formula: 2-2-2-2. Abdomen light pink, with a black tip. Epigyne: ventral fig semi-transparent, equipped with long veins forming two loops on it (Figs 54A, 55C); dorsal fig large, heart-shaped (Figs 54B–C, 55D); copulatory ducts heading towards laterally then turning up and joining the spermathecae (Figs 54C, 55D); spermathecae somewhat oval (Fig. 54B).

###### Distribution.

Known only from type locality.

#### Genus *Maro* O. P.-Cambridge, 1906

*Maro*: O. P.-Cambridge 1906: 77. Type species *Maro minutus* O. P.-Cambridge, 1906.

##### Maro bulbosus sp. n.

Taxon classificationAnimaliaAraneaeLinyphiidae

http://zoobank.org/F475F573-ECFF-4092-9CEF-1A040EEE6803

Figs 56 , 57

###### Types.

Holotype ♀: CHINA, Yunnan: Menglun Town: Xishuangbanna Tropical Botanical Garden, 21°55.035'N, 101°16.500'E, elevation ca 558 m, 1.–15.06.2007, primary tropical seasonal rain forest. Paratypes 2♀, 21°57.445'N, 101°12.997'E, elevation ca 744 m, 1.–15.06.2007 primary tropical seasonal rain forest; 21°55.428'N, 101°16.441'E, elevation ca 598 m, 16.–31.07.2007, secondary tropical seasonal rain forest; 1♀, 21°54.646'N, 101°16.257'E, ca 572 m, 1.–9.12.2006, rubber-tea plantation.

###### Etymology.

The species’ name is derived from the Latin word ‘bulbosus’, meaning ‘bulb-like’, referring to the hemisphere-shaped epigyne; adjective.

###### Diagnosis.

**Figure 56.**
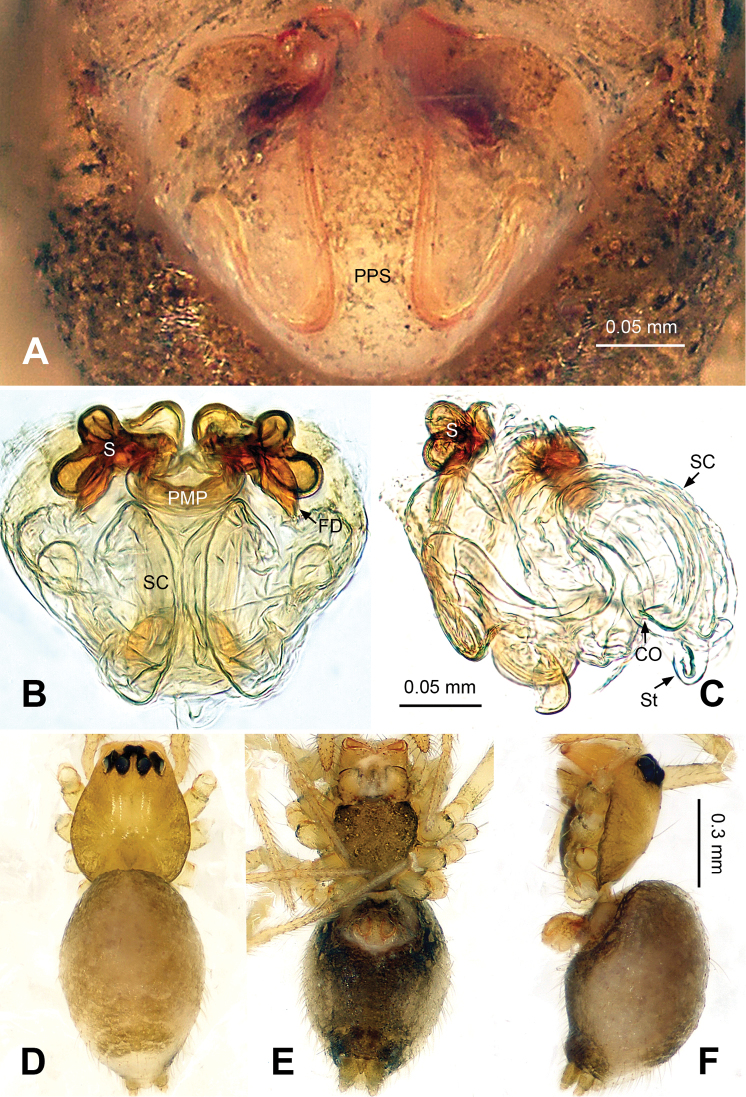
*Maro bulbosus* sp. n., female holotype. **A** Epigyne, ventral view **B** Epigyne, dorsal view **C** Vulva, lateral view **D** Habitus, dorsal view **E** Habitus, ventral view **F** Habitus lateral view. Scale bars: **B** as **C**, **D** and **E** as **F.**

**Figure 57.**
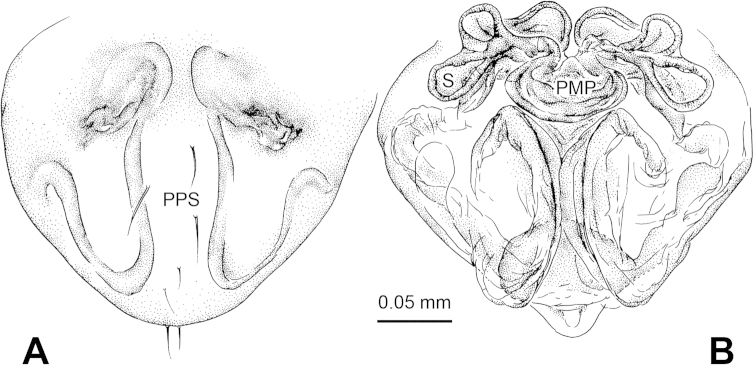
*Maro bulbosus* sp. n., female holotype. **A** Epigyne, ventral view **B** Vulva, dorsal view Scale bar: **A** as **B.**

This species resembles *Maro flavescens* (O. P.-Cambridge, 1873) by the conformation of ventral fig, but differs in these aspects: the posterior median fig is elliptical in *Maro bulbosus* sp. n. (Fig. 56B), whereas it is tongue-shaped in *Maro flavescens* (Tanasevitch 2006a: figs 35–36); spermathecae of *Maro bulbosus* sp. n. are close to each other and have multi-chambered spermathecae (Figs 56B, 57B), while big, round ones in *Maro flavescens*.

###### Description.

Female (holotype). Total length: 1.17. Carapace 0.50 long, 0.43 wide, yellow. Sternum 0.30 long, 0.30 wide. Clypeus 0.10 high. Chelicerae promargin with 3 teeth, retromargin with 2 teeth. Eye sizes and interdistances: AME 0.03, ALE 0.06, PME 0.05, PLE 0.05, AME-AME/AME 0.33, PME-PME/PME 0.60, AME-ALE/ALE 0.50, PME-PLE/PLE 0.80, coxae IV separated by 0.92 time their width. Length of legs: I 1.72 (0.44, 0.15, 0.45, 0.38, 0.30), II 1.57 (0.34, 0.16, 0.41, 0.37, 0.29), III 1.41 (0.36, 0.16, 0.32, 0.31, 0.26), IV 1.78 (0.48, 0.14, 0.46, 0.40, 0.30). Leg formula: IV-I-II-III. TmI 0.34, TmIV absent. Tibial spine formula: 2-2-1-1. Abdomen with yellow dorsum and brown venter. Epigyne: semi-transparent, with part of spermathecae and copulatory ducts visible, posterior margin convax with a long, folded stretcher (Fig. 56B); posterior median fig, small, oval (Fig. 56B); copulatory ducts long, forming a semi-loop structure before connecting with spermathecae.

Male. Unknown.

###### Distribution.

Known only from type localities.

#### Genus *Nasoona* Locket, 1982

*Nasoona*: Locket 1982: 366. Type species *Nasoona prominula* Locket, 1982 from Malaysia.

##### Nasoona asocialis

Taxon classificationAnimaliaAraneaeLinyphiidae

(Wunderlich, 1974)

Oedothorax asocialis : Wunderlich 1974: 172, figs 6–7 (♀).
Nasoona asocialis : Tanasevitch 2014b: 78, figs 33–38, 44–50 (♂♀).

###### Material examined.

2♂4♀, CHINA, Yunnan: Menglun Town: Xishuangbanna Tropical Botanical Garden, 21°55.035'N, 101°16.500'E, elevation ca 558 m, 22.07.2007, primary tropical seasonal rain forest, fogging; 3♀, 21°55.035'N, 101°16.500'E, elevation ca 558 m, 19.–25.02.2007, Primary tropical seasonal rain forest, hand-collecting; 1♀, 21°54.200'N, 101°16.923'E, elevation ca 608 m, 10.–20.06.2007, *Paramichelia baillonii* Plantation, hand-collecting; 3♂8♀, 21°55.139'N, 101°16.295'E, elevation ca 523 m, 30.11.2009, tropical evergreen rain forest, fogging.

###### Distribution.

China, Laos, Nepal.

##### Nasoona crucifera

Taxon classificationAnimaliaAraneaeLinyphiidae

(Thorell, 1895)

Erigone crucifera : Thorell 1895: 110 (♀).
Nasoona crucifera : Tanasevitch 2010: 104, figs 39–43 (♂♀).

###### Material examined.

1♀, CHINA, Yunnan: Menglun Town: Xishuangbanna Botanical Garden, 21°57.445'N, 101°12.997'E, elevation ca 744 m, 5.–12.01.2007, primary tropical seasonal rain forest, fogging; 1♂, 21°55.035'N, 101°16.560'E, elevation ca 558 m, 12.05.2007, primary tropical seasonal rain forest, fogging; 1♂, 21°53.823'N, 101°17.072'E, elevation ca 613 m, 6.–12.03.2007, *Paramichelia baillonii* plantation, fogging; 1♀, 21°54.674'N, 101°16.207'E, elevation ca 583 m, 11.11.2009, rubber tree plantation, fogging; 1♀, 21°54.555'N, 101°16.860'E, elevation ca 610 m, 29.11.2009, evergreen forest, fogging; 1♂, G213 Road, 21°53.992'N, 101°16.948'E, elevation ca 596 m, 2.12.2009, *Anogeissus acuminate* plantation, fogging.

###### Distribution.

China, Myanmar, Vietnam.

#### Genus *Nasoonaria* Wunderlich & Song, 1995

*Nasoonaria*: Wunderlich and Song 1995: 346. Type species *Nasoonaria sinensis* Wunderlich & Song, 1995 from China.

##### Nasoonaria circinata sp. n.

Taxon classificationAnimaliaAraneaeLinyphiidae

http://zoobank.org/B3E2B97F-3B5E-4269-9A64-7BD8A8F3699C

**Figure 58.**
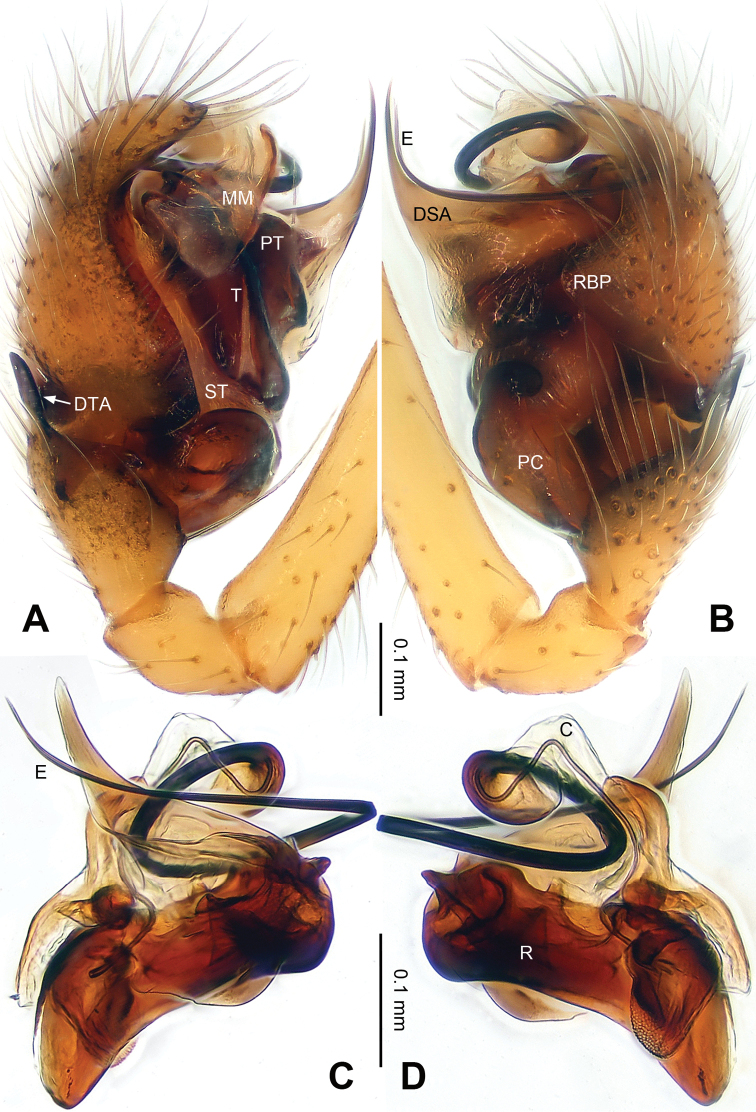
*Nasoonaria circinata* sp. n., male holotype. **A** Palp, prolateral view **B** Palp, retrolateral view **C** Embolic division, ventral view **D** Embolic division, dorsal view. Scale bars: **A** as **B**, **C** as **D.**

**Figure 59.**
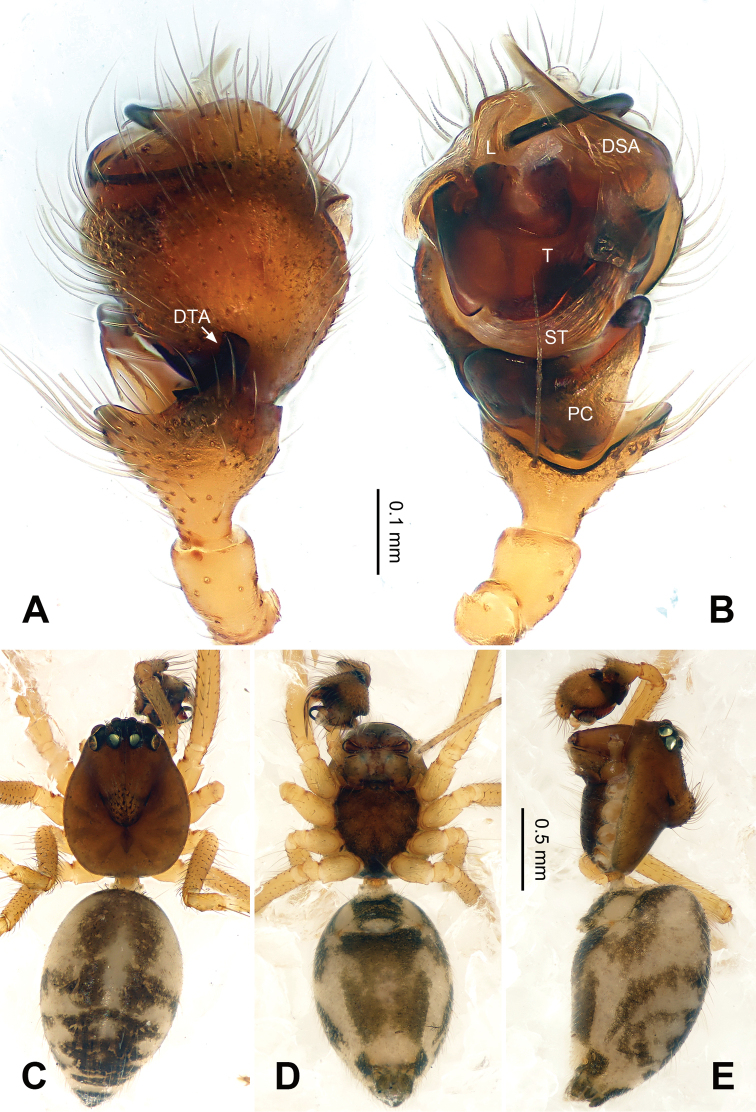
*Nasoonaria circinata* sp. n., male holotype. **A** Palp, dorsal view **B** Palp, ventral view **C** Habitus, dorsal view **D** Habitus, ventral view **E** Habitus, lateral view. Scale bars: **A** as **B**, **C** and **D** as **E.**

**Figure 60.**
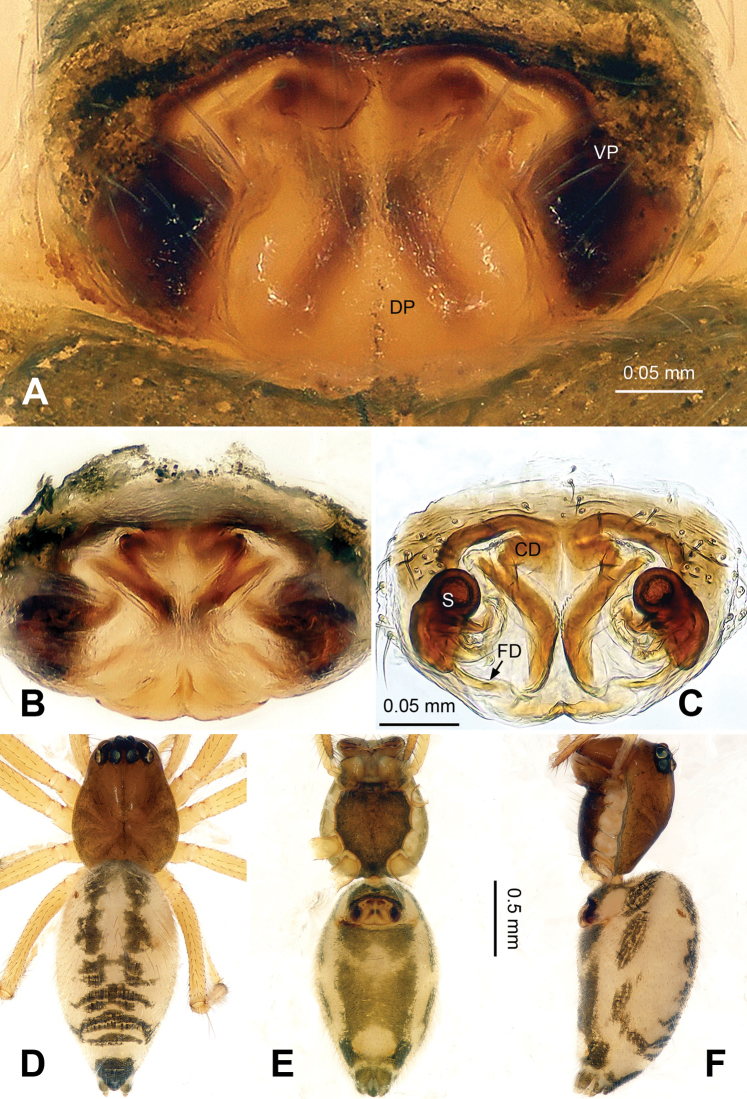
*Nasoonaria circinata* sp. n., female paratype. **A** Epigyne, ventral view **B** Epigyne, dorsal view **C** Vulva, dorsal view **D** Habitus, dorsal view **E** Habitus, ventral view **F** Habitus lateral view. Scale bars: **B** as **C**, **D** and **E** as **F.**

**Figure 61.**
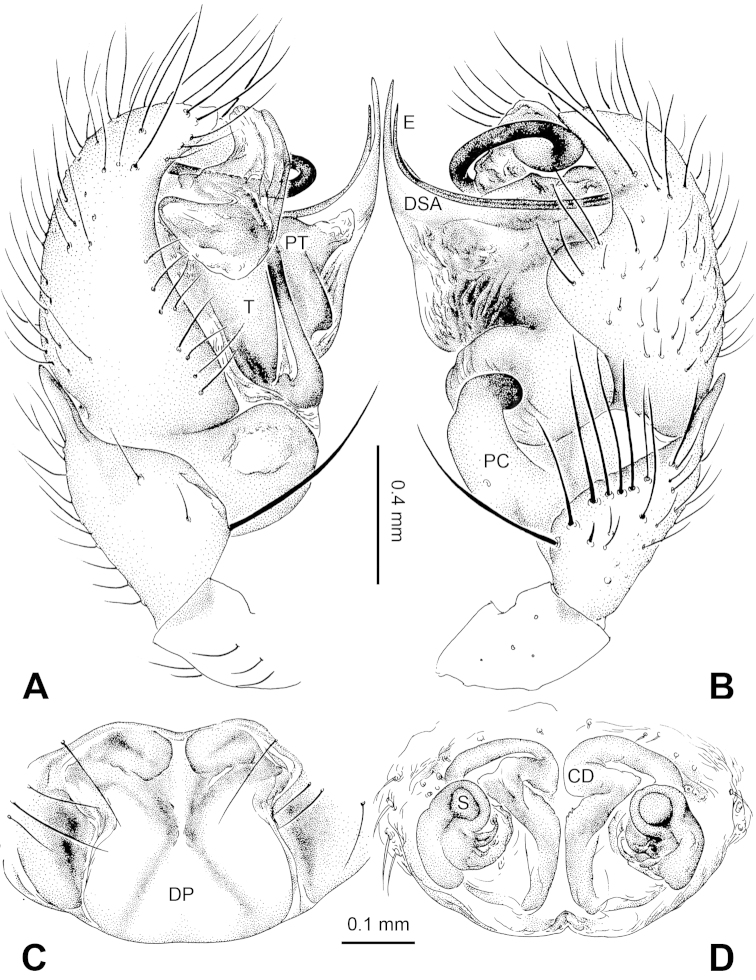
*Nasoonaria circinata* sp. n., male holotype (**A**–**B**) and female paratype (**C**–**D**). **A** Palp, prolateral view **B** Palp, retrolateral view **C** Epigyne, ventral view **D** Vulva, dorsal view. Scale bars: **A** as **B**, **C** as **D.**

Figs 58 , 59 , 60 , 61

###### Types.

Holotype ♂: CHINA, Yunnan: Menglun Town: Xishuangbanna Tropical Botanical Garden, 21°55.035'N, 101°16.500'E, elevation ca 558 m, 22.07.2007, primary tropical seasonal rain forest, fogging. Paratypes 1♂1♀, same data as holotype; 1♀, 21°54.718'N, 101°16.940'E, elevation ca 645 m, 5.–12.02.2007, primary tropical seasonal moist forest, Menglun Nature Reserve, hand-collecting; 2♂, 21°55.035'N, 101°16.500'E, elevation ca 558 m, 1.–15.04.2007, primary tropical seasonal rain forest, Menglun Nature Reserve, trunk traps; 2♂2♀, 21°55.035'N, 101°16.500'E, elevation ca 558 m, 16.–31.05.2007, primary tropical seasonal rain forest, pitfall traps; 2♂5♀, 21°54.380'N, 101°16.815'E, elevation ca 620 m, 21.11.2009, G213 Road, bamboo plantation, fogging; 10♂14♀, 21°54.614'N, 101°16.890'E, elevation ca 640 m, 14.11.2009, Lvshilin tropical rain forest, fogging; 4♀, 21°54.714'N, 101°16.935'E, elevation ca 660 m, 16.11.2009, Lvshilin tropical rain forest, fogging; 1♂13♀, 21°54.705'N, 101°16.898'E, elevation ca 664 m, 15.11.2009, Lvshilin tropical rain forest, fogging; 15♀, 21°53.646'N, 101°16.975'E, elevation ca 589 m, 26.11.2009, G213 Road, bamboo plantation, fogging; 3♀, 21°53.622'N, 101°16.955'E, elevation ca 581 m, 26.11.2009, G213 Road, bamboo plantation, fogging; 5♂7♀, 21°53.794'N, 101°17.152'E, elevation ca 594 m, 27.11.2009, G213 road, evergreen forest, fogging; 2♀, 21°54.089'N, 101°17.024'E, elevation ca 570 m, 28.11.2009, G213 road, fogging.

###### Etymology.

This specific name originates from the Latin word ‘circinatus, meaning ‘coiled’, for the long embolus exhibiting coil-like loops; adjective.

###### Diagnosis.

Male of *Nasoonaria circinata* sp. n. can be distinguished from the type species by the modified carapace (Fig. 59E), prominent distal suprategular apophysis and the long, coiled embolus in palp (Fig. 58B). The female is distinguished from *Nasoonaria sinensis* Wunderlich & Song, 1995 by the different pathway taken by the copulatory ducts and by the position of spermathecae (Fig. 60C).

###### Description.

Male (holotype). Total length: 2.35. Carapace 0.95 long, 0.75 wide, reddish brown, raised into a hump in the middle of the thoracic part, with a row of long setae growing along the axis from the ocular area to the hump. Sternum 0.47 long, 0.47 wide. Clypeus 0.16 high. Chelicerae promargin with 5 teeth, retromargin with 4 teeth. Eye sizes and interdistances: AME 0.06, ALE 0.10, PME 0.08, PLE 0.10, AME-AME/AME 0.50, PME-PME/PME 0.50, AME-ALE/ALE 0.50, PME-PLE/PLE 0.75, coxae IV separated by 1.38 their width. Length of legs: I 4.27 (1.13, 0.30, 0.97, 1.09, 0.78), II 4.15 (1.09, 0.30, 1.13, 1.00, 0.63), III 3.13 (0.76, 0.28, 0.78, 0.78, 0.53), IV 4.01 (1.13, 0.25, 1.00, 1.00, 0.63). Leg formula: I-II-IV-III. TmI 0.61, TmIV 0.69. Tibial spine formula: 2-2-1-1. Palp: tibia cone-shaped, with slightly protruding dorsal apophysis (Fig. 59A) and 2 retrolateral trichobothria. Paracymbium ‘U’-shaped, with wide base and attenuated, round black apex (Fig. 58B); Embolus long, slim, forming two coils (Fig. 58A–D). Distal suprategular apophysis broad at base, distally taper (Fig. 58B).

Female (one of paratypes). Total length: 2.50. Carapace 0.90 long, 0.75 wide, similar to that of male in coloration, but slightly lighter, unmodified. Sternum 0.53 long, 0.53 wide. Clypeus 0.15 high. Chelicerae promargin with 6 teeth, retromargin with 4 teeth. Eyes diameter and interdistances: AME 0.08, ALE 0.10, PME 0.08, PLE 0.08, AME-AME/AME 0.25, PME-PME/PME 0.25, AME-ALE/ALE 0.30, PME-PLE/PLE 0.25, Coxae IV separated by 1.36 times their width. Lengths of legs: I 3.96 (1.10, 0.30, 1.06, 0.90, 0.60), II 3.71 (1.00, 0.30, 0.90, 0.88, 0.63), III 3.00 (0.81, 0.28, 0.75, 0.69, 0.47), IV 3.94 (1.06, 0.25, 1.00, 1.00, 0.63). Leg formula: I-IV-III-II. TmI 0.60, TmIV 0.66. Epigyne: ventral fig slightly narrowed mesally (Fig. 60A). Copulatory ducts long and sinuous, extending anteriorly then turning back to connect with spermathecae (Fig. 60C). Spermathecae globular, separated from each other by about 1.5 their diameters (Fig. 60C). Fertilization ducts directed mesally (Fig. 60C).

###### Distribution.

Known only from type localities.

##### Nasoonaria sinensis

Taxon classificationAnimaliaAraneaeLinyphiidae

Wunderlich & Song, 1995

Nasoonaria sinensis : Wunderlich and Song 1995: 347, figs 11–18 (♂♀).

###### Material examined.

1♂, CHINA, Yunnan: Menglun Town: Xishuangbanna Nature Reserve, 21°54.984'N, 101°16.982'E, elevation ca 656 m, 19.–26.05.2006, secondary tropical seasonal rain forest, hand-collecting; 1♂1♀, 21°54.747'N, 101°11.431'E, elevation ca 880 m, 19.–27.08.2006, secondary tropical montane evergreen broad-leaved forest, hand-collecting; 1♂, 21°54.984'N, 101°16.982'E, elevation ca 656 m, 5.–12.09.2006, secondary tropical seasonal rain forest, hand-collecting; 1♀, 21°54.772'N, 101°16.043'E, elevation ca 556 m, 19.–25.01.2007, *Paramichelia baillonii* plantation, hand-collecting; 1♀, 21°54.772'N, 101°16.043'E, elevation ca 556 m, 19.–25.01.2007, secondary tropical seasonal rain forest, hand-collecting.

###### Distribution.

China.

#### Genus *Nematogmus* Simon, 1884

*Nematogmus*: Simon 1884: 615. Type species *Theridion sanguinolentum* Walckenaer, 1841.

##### Nematogmus sanguinolentus

Taxon classificationAnimaliaAraneaeLinyphiidae

(Walckenaer, 1841)

Theridion sanguinolentum : Walckenaer 1841: 326 (♀).
Nematogmus sanguinolentus : Simon 1884: 615, figs 431–432 (♂♀).

###### Material examined.

3♀, CHINA, Yunnan: Mengla County: Xishuangbanna Nature Reserve, Xiaolongha biodiversity preservation corridor, 21°24.832'N, 101°37.906'E, elevation ca 721 m, 18.11.2013, valley evergreen rain forest, sieving; 1♂9♀, Xishuangbanna Nature Reserve, Xiaolongha biodiversity preservation corridor, 21°24.832'N, 101°37.906'E, elevation ca 721 m, 19.06.2013, valley evergreen rain forest, sieving; 3♀, Mengyang Town: Baihuashan Tunnel: Xishuangbanna nature reserve, 22°09.513'N, 100°53.220'E, elevation ca 894 m, 25.06.2013, tropical seasonal rain forest, sieving; 2♀, 22°09.481'N, 100°53.358'E, elevation ca 872 m, 29.06.2013, tropical seasonal rain forest, sieving.

###### Distribution.

Palearctic.

#### Genus *Neriene* Blackwall, 1833

*Neriene*: Blackwall 1833: 188. Type species *Neriene marginata* Blackwall, 1833 (= *Nematogmus clathrata* (Sundevall, 1830)).

##### Neriene circifolia sp. n.

Taxon classificationAnimaliaAraneaeLinyphiidae

http://zoobank.org/EE96A747-DA48-45D9-9EB9-3E55CAA73507

**Figure 62.**
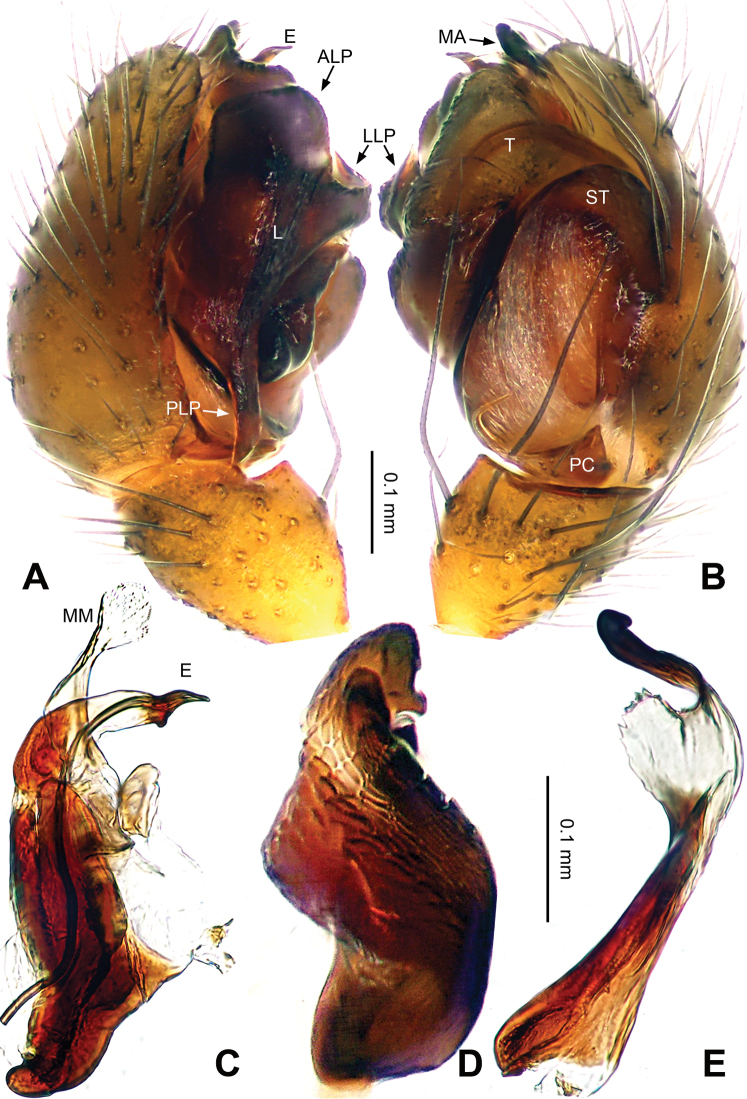
*Neriene circifolia* sp. n., male holotype. **A** Palp, prolateral view **B** Palp, retrolateral view **C** Embolus, prolateral view **D** Terminal apophysis, prolateral view **E** Median apophysis, ventral view. Scale bars: **B** as **A**, **C** and **D** as **E.**

**Figure 63.**
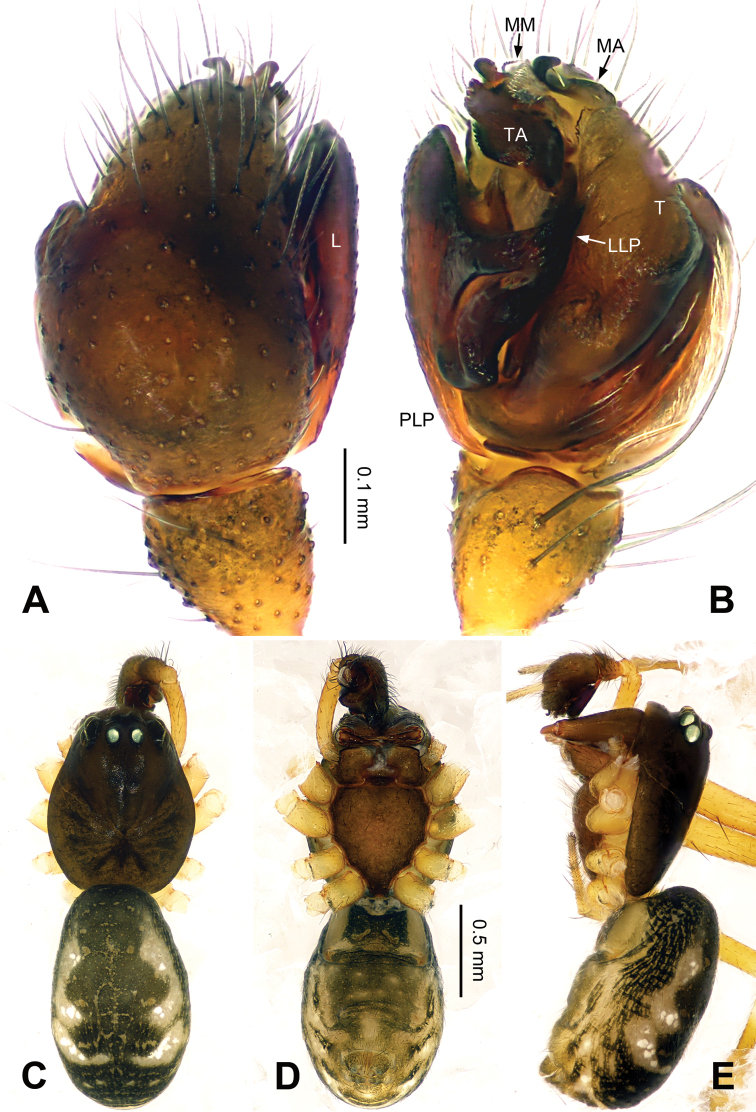
*Neriene circifolia* sp. n., male holotype. **A** Palp, dorsal view **B** Palp, ventral view **C** Habitus, dorsal view **D** Habitus, ventral view **E** Habitus, lateral view. Scale bars: **B** as **A**, **C** and **E** as **D.**

**Figure 64.**
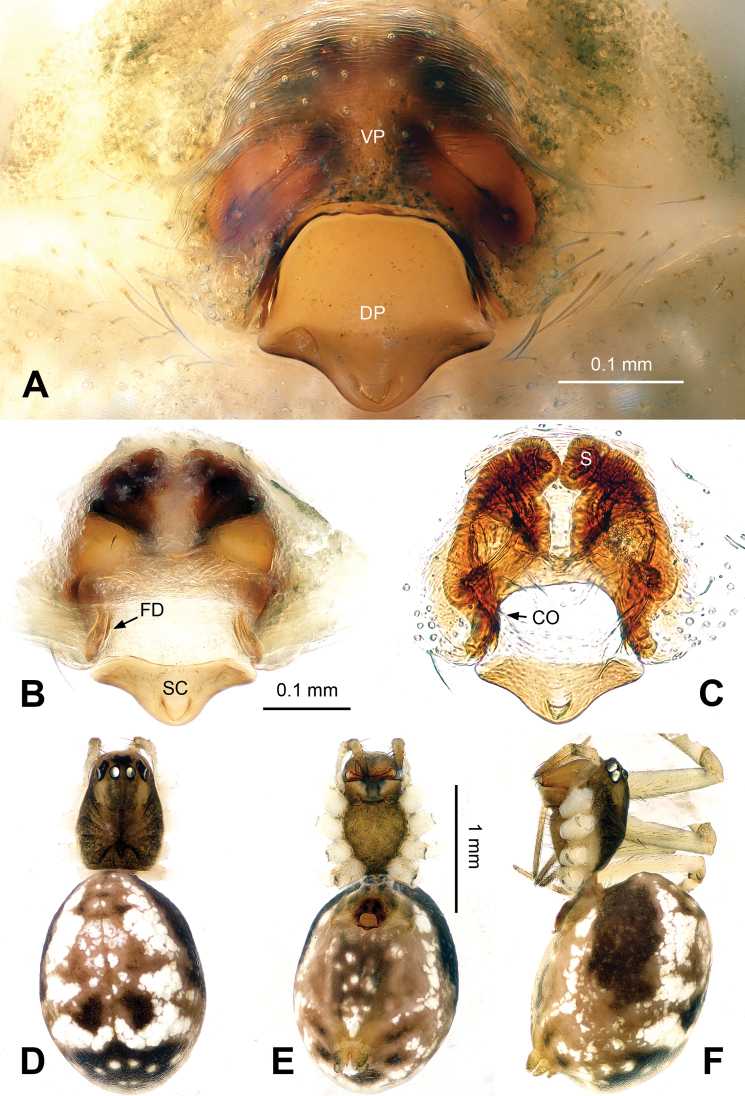
*Neriene circifolia* sp. n., female paratype. **A** Epigyne, ventral view **B** Epigyne, dorsal view **C** Vulva, dorsal view **D** Habitus, dorsal view **E** Habitus, ventral view **F** Habitus lateral view. Scale bars: **C** as **B, D** and **F** as **E.**

**Figure 65.**
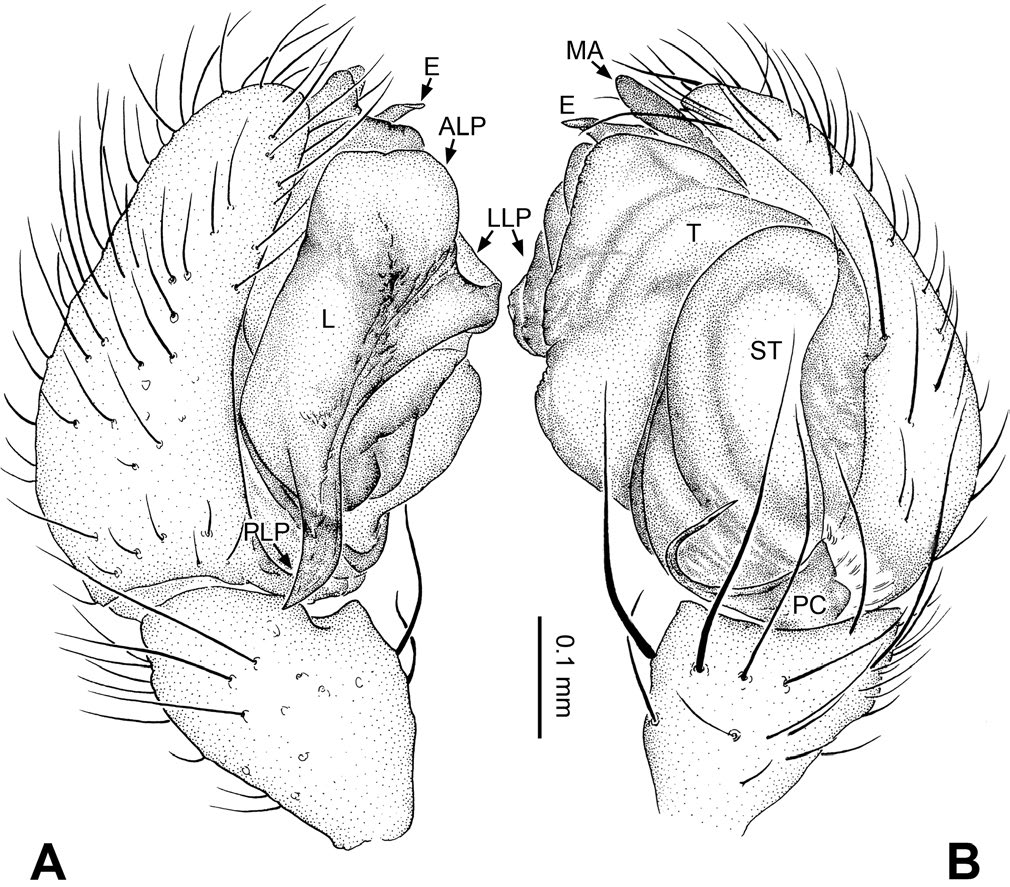
*Neriene circifolia* sp. n., male holotype. **A** Palp, prolateral view **B** Palp, retrolateral view. Scale bar: **A** as **B.**

Figs 62 , 63 , 64 , 65

###### Types.

Holotype ♂: CHINA, Yunnan: Mengla County: Xishuangbanna Nature Reserve, Xiaolongha biodiversity preservation corridor, 21°24.236'N, 101°36.268'E, elevation ca 711 m, 17.06.2013, tropical seasonal rain forest, hand-collecting. Paratype 1♀, 21°24.159'N, 101°37.178'E, elevation ca 635 m, 27.06.2012, tropical seasonal rain forest, fogging.

###### Etymology.

This specific name originates from the Latin words ‘circum’ meaning ‘in a circle’ and ‘folius’ meaning ‘leaf’, referring to the shape of the median membrane, the apex of which looks like a small, round leaf; term in apposition.

###### Diagnosis.

This species is similar to *Neriene birmanica* (Thorell, 1887) by having small paracymbium with a curved, filiform tip (Fig. 62B) and the stout, wide terminal apophysis forming about one coil (Fig. 62D). It could be distinguished from *Neriene birmanica* by the shape of embolus and lamella. *Neriene birmanica* has sword-like embolus (Xu et al. 2010: fig. 6), a slim, spear-like lateral projection of the lamella (Xu et al. 2010: fig. 3), while *Neriene circifolia* sp. n. has a rostriform embolus, slightly curved at tip (Fig. 62C), and a broader and more sclerotized lateral projection of lamella (Fig. 62A). In female’s epigyne, the spiral grooves forming one more coil than that in *Neriene birmanica* (Fig. 64C).

###### Description.

Male (holotype). Total length: 2.19. Carapace 1.00 long, 0.86 wide, greenish brown in coloration. Sternum 0.59 long, 0.56 wide. Clypeus 0.19 high. Chelicerae promargin with 6 teeth, retromargin with 6 teeth. Eye sizes and interdistances: AME 0.08, ALE 0.09, PME 0.10, PLE 0.10, AME-AME/AME 0.38, PME-PME/PME 0.60, AME-ALE/ALE 0.78, PME-PLE/PLE 1.00, coxae IV separated by 0.78 time their width. Length of legs: I 4.45 (1.19, 0.31, 1.06, 1.17, 0.72), II 3.68 (1.00, 0.31, 0.94, 1.05, 0.38), III 2.52 (0.69, 0.23, 0.53, 0.63, 0.44), IV 3.35 (0.83, 0.25, 0.75, 0.97, 0.55). Leg formula: I-II-IV-III. TmI 0.25. Patellar spine formula 2-2-2-2. Abdomen dark green with three irregular white patches at each lateral side. Palp: patella short, with one long dorsal seta; tibia with two retrolateral trichobothria, and long setae (Fig. 62A–B); paracymbium small, ‘J’-shaped, with a tapering tip (Figs 62B, 65B); lamella with three projections: anterior projection wide and blunt, the posterior one long, straight with slightly curved tip, the lateral one with sharp tip (Figs 62A, 63B). Terminal apophysis stout, broad, forming about one coil (Fig. 62D); median membrane with leaf-like tip (Fig. 62C); embolus simple, bending forward at half length, with beak-like tip (Fig. 62C).

Female (paratype). Total length: 2.68. Carapace 1.00 long, 0.64 wide, same coloration and pattern as male. Sternum 0.60 long, 0.50 wide. Clypeus 0.13 high. Chelicerae like in male. Eye sizes and interdistances: AME 0.08, ALE 0.10, PME 0.08, PLE 0.08, AME-AME/AME 0.25, PME-PME/PME 0.25, AME-ALE/ALE 0.30, PME-PLE/PLE 0.25, coxae IV separated by 1.36 times their width. Length of legs: I 4.13 (1.10, 0.34, 1.00, 1.04, 0.65), II 3.90 (1.02, 0.31, 0.94, 1.02, 0.61), III 2.52 (0.70, 0.24, 0.52, 0.66, 0.40), IV 3.52 (0.94, 0.28, 0.75, 1.00, 0.55). Leg formula: I-II-IV-III. TmI 0.60. Spination of patella like in male. Epigyne: atrium broad (Fig. 64A), scape of dorsal fig with a slightly pointed end (Fig. 64C). Spiral grooves with about three coils (Fig. 64B–C). Spermathecae situated mesally (Fig. 64C).

###### Distribution.

Known only from type localities.

###### Remarks.

**Figure 66.**
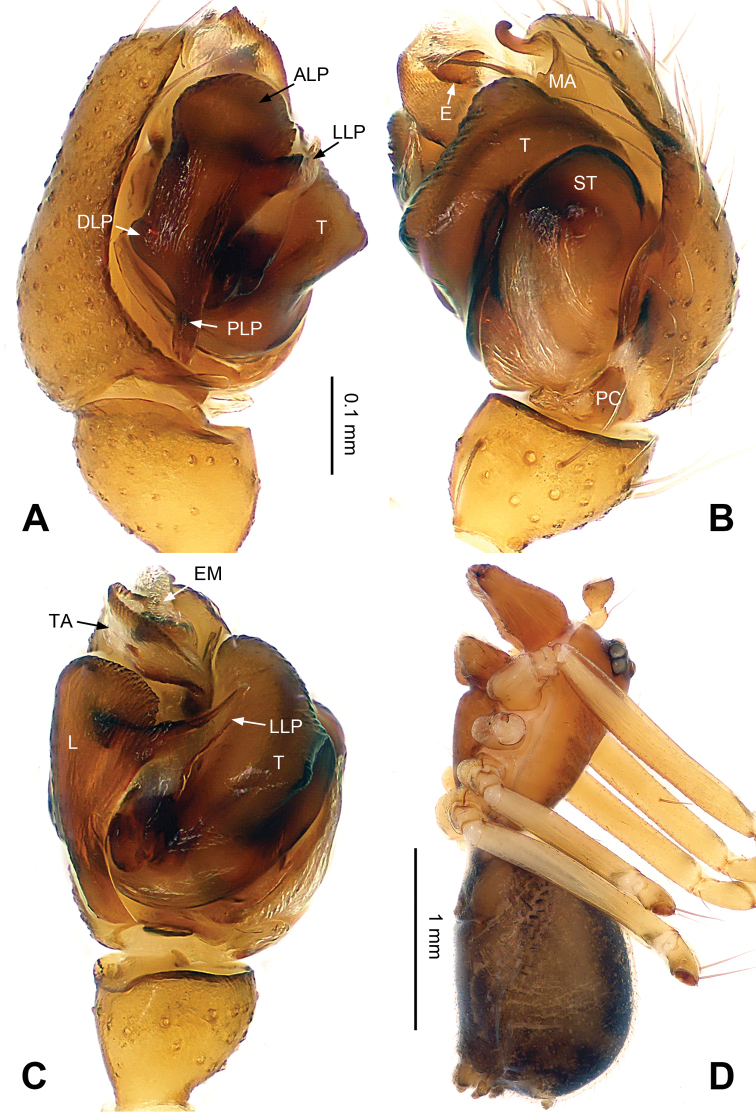
*Ambengana complexipalpis*, male holotype. **A** Palp, prolateral view **B** Palp, retrolateral view **C** Palp, ventral view **D** Habitus, lateral view. Scale bar: **A** and **C** as **B.**

We have closely examined and taken photos of the holotype of *Ambengana complexipalpis* Millidge & Russell-Smith, 1992 (Museum of Natural History, Geneva, Dr Peter J. Schwendinger), both the left palp and the habitus (Fig. 66). By comparing it with the pictures from Xu’s paper (Xu et al. 2010: figs 1–7), a few differences were found in the detailed structure of palp between two species. Whether or not the new synonymy proposed by Xu et al. (2010) is valid is uncertain, and a further study is required.

##### Neriene macella

Taxon classificationAnimaliaAraneaeLinyphiidae

(Thorell, 1898)

Linyphia macella : Thorell 1898: 319 (♂).
Neriene macella : van Helsdingen 1969: 186, figs 257–262 (♂♀).

###### Material examined.

1♂, CHINA, Yunnan: Menglun Town: Xishuangbanna Botanical Garden, 21°54.463'N, 101°15.978'E, elevation ca 569 m, 5.–12.02.2007, rubber tree plantation, hand-collecting; 1♂, 21°54.463'N, 101°15.978'E, elevation ca 569 m, 19.–26.03.2007, rubber-tea plantation, hand-collecting; 1♂, 21°53.823'N, 101°17.072'E, elevation ca 613 m, 19.–26.04.2007, *Paramichelia baillonii* plantation, hand-collecting.

###### Distribution.

China, Laos, Malaysia, Myanmar, Thailand.

##### Neriene nitens

Taxon classificationAnimaliaAraneaeLinyphiidae

Zhu & Chen, 1991

Neriene nitens : Chen and Zhang 1991: 167, figs 166. 1–9 (♂♀).

###### Material examined.

1♂, CHINA, Yunnan: Menglun Town: Xishuangbanna Nature Reserve, 21°57.445'N, 101°12.997'E, elevation ca 744 m, 30.07.2007, primary tropical seasonal rain forest, fogging.

###### Distribution.

China.

##### Neriene strandia

Taxon classificationAnimaliaAraneaeLinyphiidae

(Blauvelt, 1936)

Linyphia strandia : Blauvelt 1936: 116, pl. 5, figs 32–35, 37–38 (♂♀).

###### Material examined.

1♂, CHINA, Yunnan: Menglun Town: Xishuangbanna Botanical Garden, 21°54.646'N, 101°16.257'E, elevation ca 572 m, 1.–9.10.2006, rubber-tea plantation, fogging.

###### Distribution.

Borneo, China.

####### Genus *Oedothorax* Bertkau, 1883

*Oedothorax* Bertkau, in Förster and Bertkau 1883: 235. Type species *Neriene gibbosa* Blackwall, 1841.

##### Oedothorax biantu sp. n.

Taxon classificationAnimaliaAraneaeLinyphiidae

http://zoobank.org/75C1B392-8785-4AD8-BC82-D58EBBD7A8C4

Figs 67 , 68

###### Types.

Holotype ♀: CHINA, Yunnan: Menglun Town: Xishuangbanna Tropical Botanical Garden, 21°54.380'N, 101°16.518'E, elevation ca 627 m, 22.11.2009, bamboo plantation, fogging.

###### Etymology.

This name comes from the Chinese Pinyin ‘biān tū’, meaning ‘knobs on each side’, referring to the prominence on each side of the ventral fig of epigyne; noun in apposition.

###### Diagnosis.

**Figure 67.**
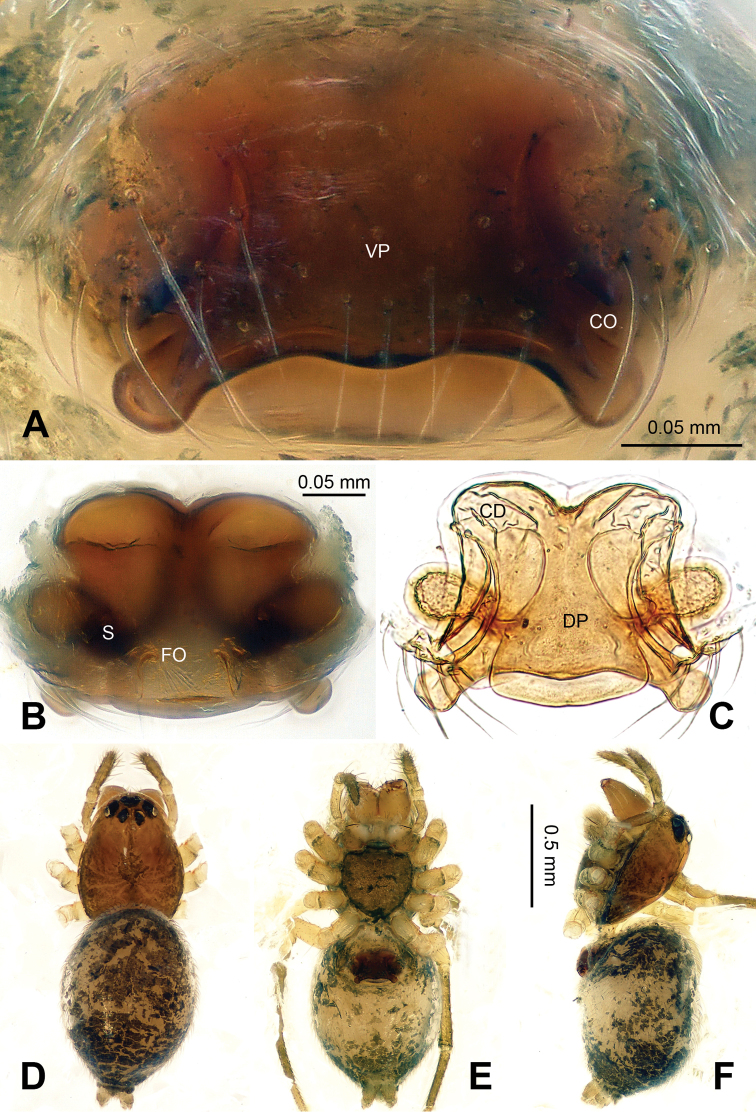
*Oedothorax biantu* sp. n., female holotype. **A** Epigyne, ventral view **B** Epigyne, dorsal view **C** Vulva, dorsal view **D** Habitus, dorsal view **E** Habitus, ventral view **F** Habitus lateral view. Scale bars: **C** as **B**, **D** and **E** as **F.**

This species is diagnosed by short copulatory ducts (Fig. 67A), position of spermathecae (Fig. 67C); it differs from congeners by tibial spination (1-1-1-1 in new species and 2-2-1-1 in other), the knob on each side of ventral fig and the long slits on the ventral fig (Fig. 67A).

###### Description.

**Figure 68.**
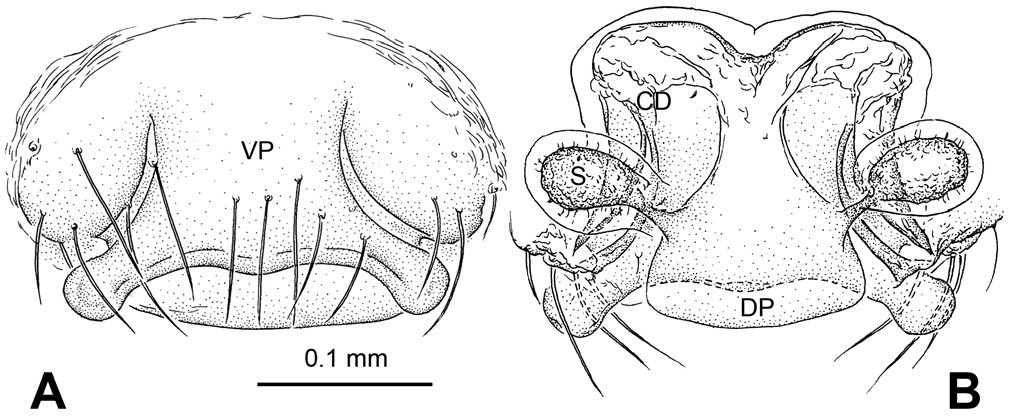
*Oedothorax biantu* sp. n., female holotype. **A** Epigyne, ventral view **B** Vulva, dorsal view Scale bar: **B** as **A.**

Female (holotype). Total length: 1.45. Carapace 0.68 long, 0.56 wide, dark brown. Sternum 0.31 long, 0.35 wide. Clypeus 0.10 high. Chelicerae promargin with 3 teeth, retromargin with 3 teeth. Eye sizes and interdistances: AME 0.04, ALE 0.06, PME 0.05, PLE 0.06, AME-AME/AME 0.75, PME-PME/PME 0.60, AME-ALE/ALE 0.33, PME-PLE/PLE 0.67, coxae IV separated by 1.36 times their width. Length of legs: I 1.57 (0.44, 0.19, 0.38, 0.31, 0.25), II 1.50 (0.43, 0.19, 0.35, 0.28, 0.25), III 1.32 (0.34, 0.16, 0.34, 0.25, 0.23), IV 1.63 (0.47, 0.16, 0.41, 0.34, 0.25). Leg formula: IV-I-II-III. TmI 0.59, TmIV 0.59. Tibial spine formula 1-1-1-1 Abdomen greenish grey. Epigyne: ventral fig broad and slightly swollen, with a wavy posterior rim and one small knob on each side (Figs 67A, 68A); copulatory ducts complex (Figs 67C, 68B); spermathecae oval, separated by 2.00 times their diameter; fertilization ducts short (Fig. 67B).

Male. Unknown.

###### Distribution.

Known only from type locality.

####### Genus *Oilinyphia* Ono & Saito, 1989

*Oilinyphia*: Ono and Saito 1989: 232. Type species *Oilinyphia peculiaris* Ono & Saito, 1989 from Japan.

##### Oilinyphia hengji sp. n.

Taxon classificationAnimaliaAraneaeLinyphiidae

http://zoobank.org/25D0F06E-5735-4DC8-8C43-6232EBF98076

**Figure 69.**
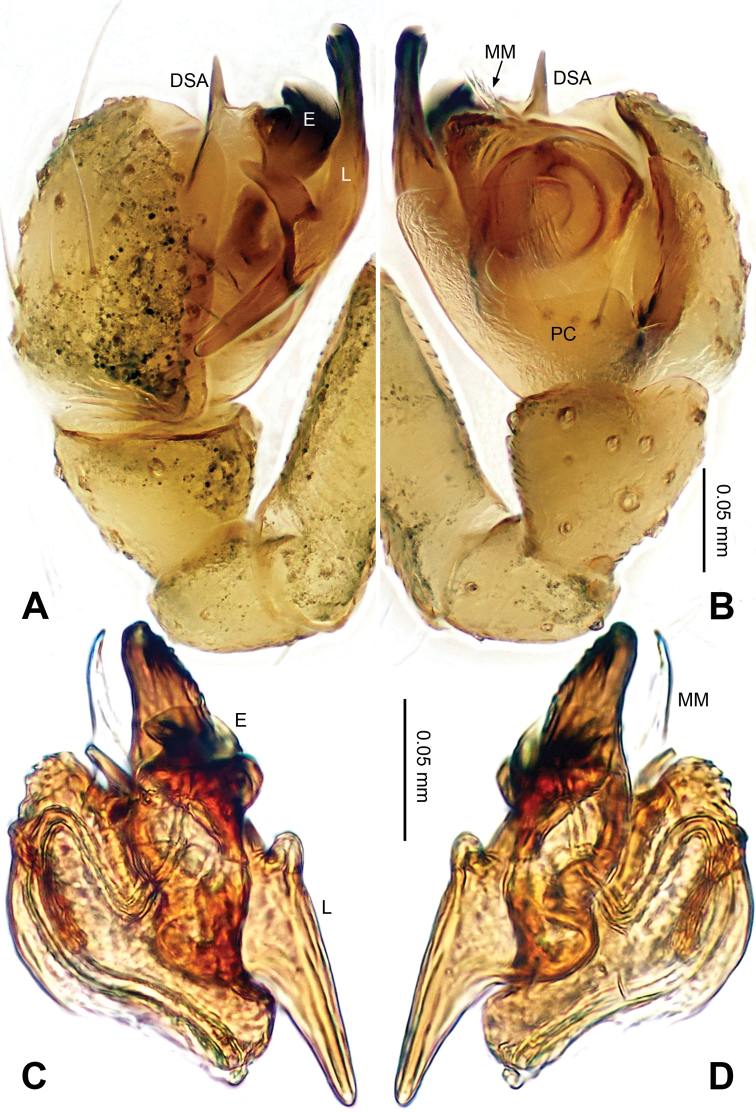
*Oilinyphia hengji* sp. n., male holotype. **A** Palp, prolateral view **B** Palp, retrolateral view **C** Embolic division, dorsal view **D** Embolic division, ventral view. Scale bars: **A** as **B**, **C** as **D.**

**Figure 70.**
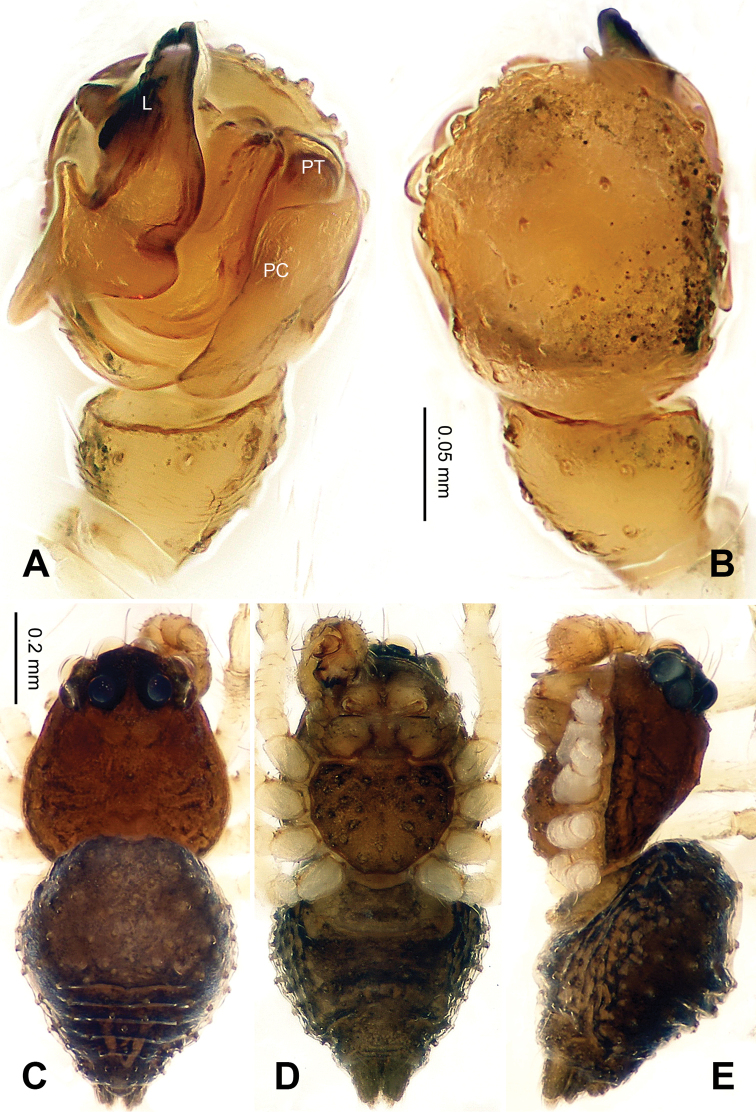
*Oilinyphia hengji* sp. n., male holotype. **A** Palp, ventral view **B** Palp, dorsal view **C** Habitus, dorsal view **D** Habitus, ventral view **E** Habitus, lateral view. Scale bars: **A** as **B**, **D** and **E** as **C.**

**Figure 71.**
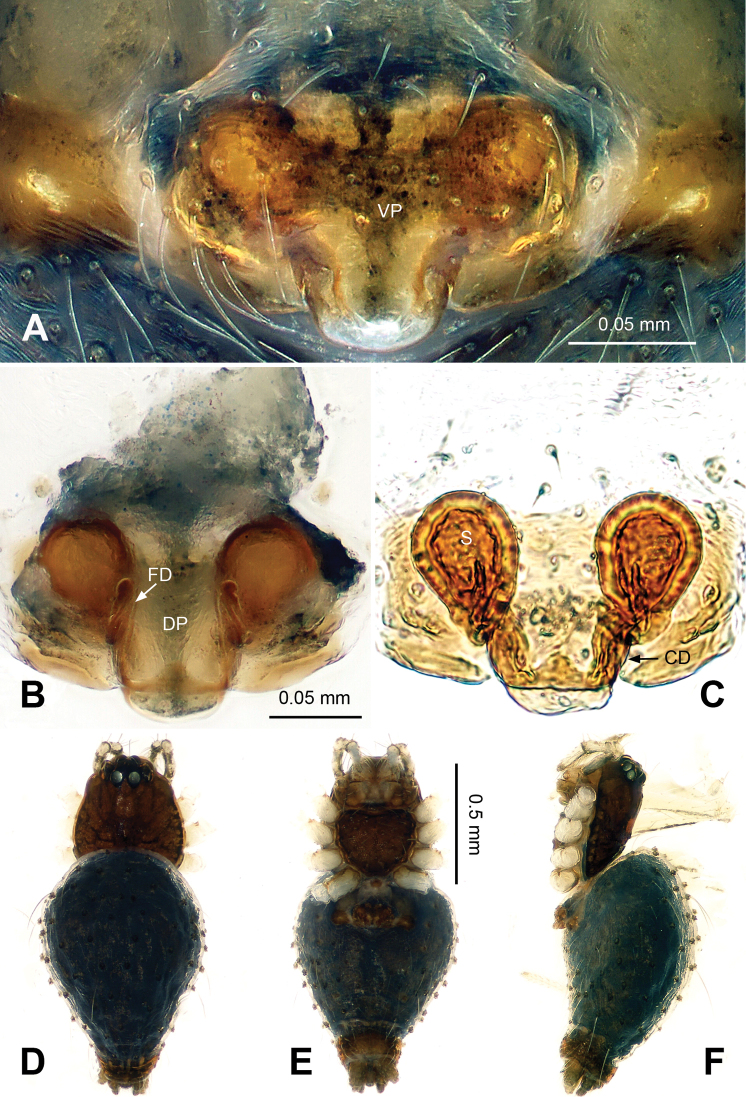
*Oilinyphia hengji* sp. n., female paratype. **A** Epigyne, ventral view **B** Epigyne, dorsal view **C** Vulva, dorsal view **D** Habitus, dorsal view **E** Habitus, ventral view **F** Habitus lateral view. Scale bars: **C** as **B**, **D** and **F** as **E.**

**Figure 72.**
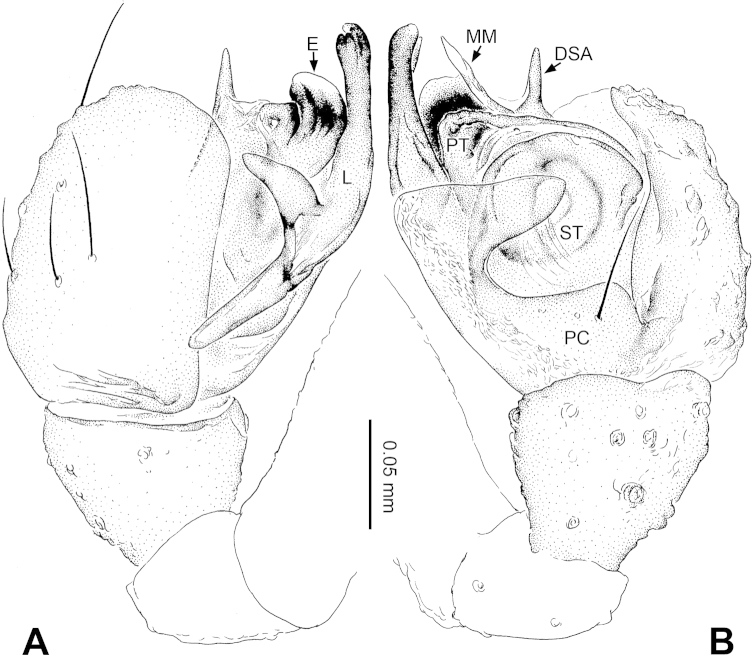
*Oilinyphia hengji* sp. n., male holotype. **A** Palp, prolateral view **B** Palp, retrolateral view. Scale bar: **A** as **B.**

Figs 69 , 70 , 71 , 72

###### Types.

Holotype ♂: CHINA, Yunnan: Menglun Town: Xishuangbanna Nature Reserve, 21°54.725'N, 101°13.261'E, elevation ca 734 m, 8.08.2007, primary tropical seasonal rain forest, fogging. Paratype 1♀, Lvshilin, 21°54.617'N, 101°16.843'E, elevation ca 738 m, 8.08.2011, primary tropical seasonal rain forest, fogging.

###### Etymology.

The name comes from the Chinese Pinyin ‘héng jĭ’, which means ‘ridge’, referring to the transversal ridges on the dorsum of its abdomen; noun in apposition.

###### Diagnosis.

This species is similar to *O. jadbounorum Ponksee* & Tanikawa, 2010 from Thailand by the body shape, conformation of palp and epigyne, but differs from sibling species by wide tip of the embolus (Fig. 69A) (pointed in *Oilinyphia jadbounorum* (Ponksee and Tanikawa 2010: fig. 3)). Females of two species differ by the shape of the median extension of the ventral fig tapering in *Oilinyphia jadbounorum* (Ponksee and Tanikawa 2010: fig. 2) and almost rectangular in the new species (Fig. 71A). The new species clearly differs from the generotype by having TmIV, 2 cheliceral teeth instead of 3, small papillae on the abdomen and by the shape of the copulatory organs.

###### Description.

Male (holotype). Total length: 1.00. Carapace 0.53 long, 0.48 wide, reddish brown. Sternum 0.28 long, 0.35 wide, heart-shaped, sparsely clothed by small tubercles. Clypeus 0.08 high. Chelicerae promargin with 2 teeth, retromargin with 0 teeth. Eye sizes and interdistances: AME 0.08, ALE 0.10, PME 0.08, PLE 0.07, AME-AME/AME 0.06, PME-PME/PME 0.75, AME-ALE/ALE 0.25, PME-PLE/PLE 0.29, coxae IV separated by 1.41 their diameters. Length of legs: I 1.72 (0.44, 0.16, 0.40, 0.41, 0.31), II 1.87 (0.48, 0.18, 0.45, 0.42, 0.34), III 1.31 (0.22, 0.12, 0.34, 0.33, 0.30), IV 1.72 (0.45, 0.15, 0.44, 0.38, 0.30). Leg formula: I-II-IV-III. TmI 0.32, TmIV 0.24. Abdomen dark green, cone-like with attenuated posterior tip, adorned with ridges and papillae. Palp: tibia with one retrolateral trichobothrium and without apophyses; paracymbium broad at base, ‘C’-shaped (Figs 69B, 72B); protegulum somewhat triangular (Fig. 77B); distal suprategular apophysis thorn-like (Fig. 69A–B); median membrane slender and short, with attenuated tip (Fig. 69C–D); lamella with three apophyses, the distal one long, with a blunt end; the mesal one short, with pointed apex; the proximal one long and straight; embolus short, broad, slightly curved (Figs 69A, 72A).

Female (paratype). Total length: 1.38. Carapace 0.56 long, 0.39 wide, same coloration as in male. Sternum 0.31 long, 0.35 wide. Clypeus 0.09 high. Chelicerae promargin with 3 teeth, retromargin with 3 teeth. Eye sizes and interdistances: AME 0.05, ALE 0.05, PME 0.05, PLE 0.05, AME-AME/AME 0.20, PME-PME/PME 0.50, AME-ALE/ALE 0.60, PME-PLE/PLE 0.56, coxae IV separated by 1.36 times their width. Length of legs: I 1.90 (0.50, 0.15, 0.45, 0.45, 0.35), II 2.05 (0.50, 0.19, 0.50, 0.48, 0.35), III 1.67 (0.47, 0.16, 0.34, 0.38, 0.32), IV 1.63 (0.47, 0.16, 0.41, 0.34, 0.25). Leg formula: IV-I-II-III. TmI 0.28, TmIV 0.59. Abdomen same coloration and pattern as male. Epigyne: ventral fig wide, extended mesally with a truncated end (Fig. 71A); copulatory ducts short and simple; spermathecae oval, separated by less than their diameter (Fig. 71C); fertilization ducts short, turning upwards (Fig. 71B).

###### Distribution.

Known only from type localities.

####### Genus *Paikiniana* Eskov, 1992

*Paikiniana*: Eskov 1992: 164. Type species *Cornicularia bella* Paik, 1978 from Korea.

##### Paikiniana furcata sp. n.

Taxon classificationAnimaliaAraneaeLinyphiidae

http://zoobank.org/59D05E5C-FB7B-472E-8EF1-CCB351C49463

**Figure 73.**
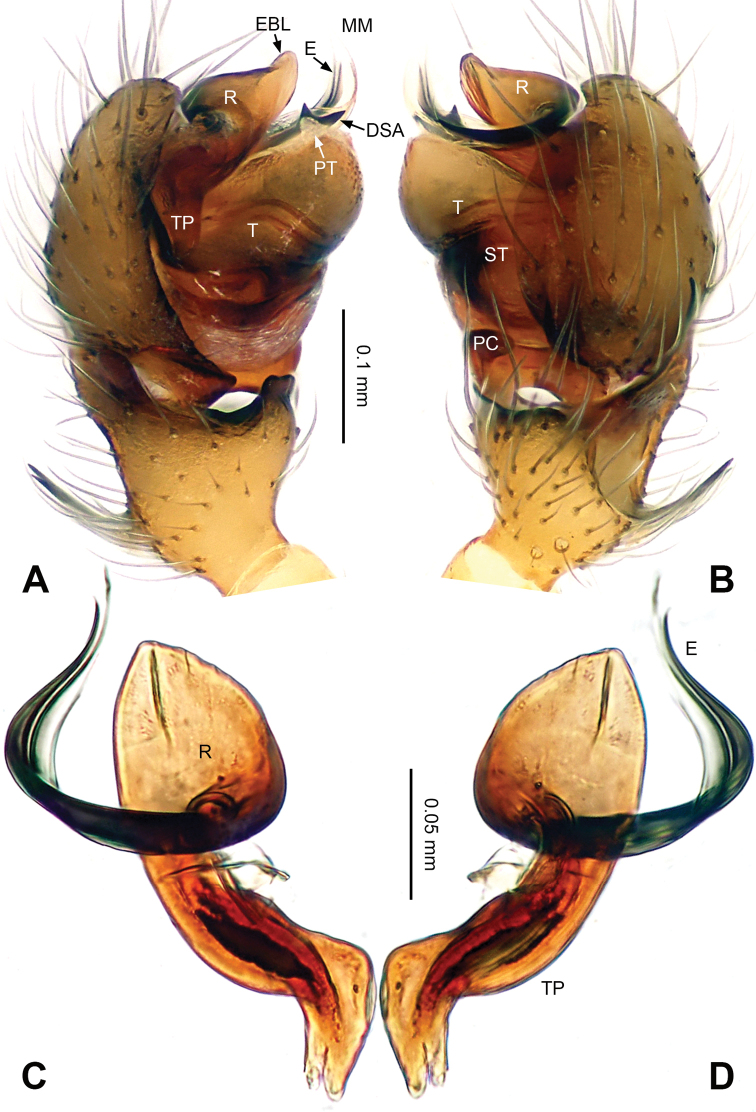
*Paikiniana furcata* sp. n., male holotype. **A** Palp, prolateral view **B** Palp, retrolateral view **C** Embolic division, retrolateral view **D** Embolic division, prolateral view. Scale bars: **B** as **A**, **C** as **D.**

**Figure 74.**
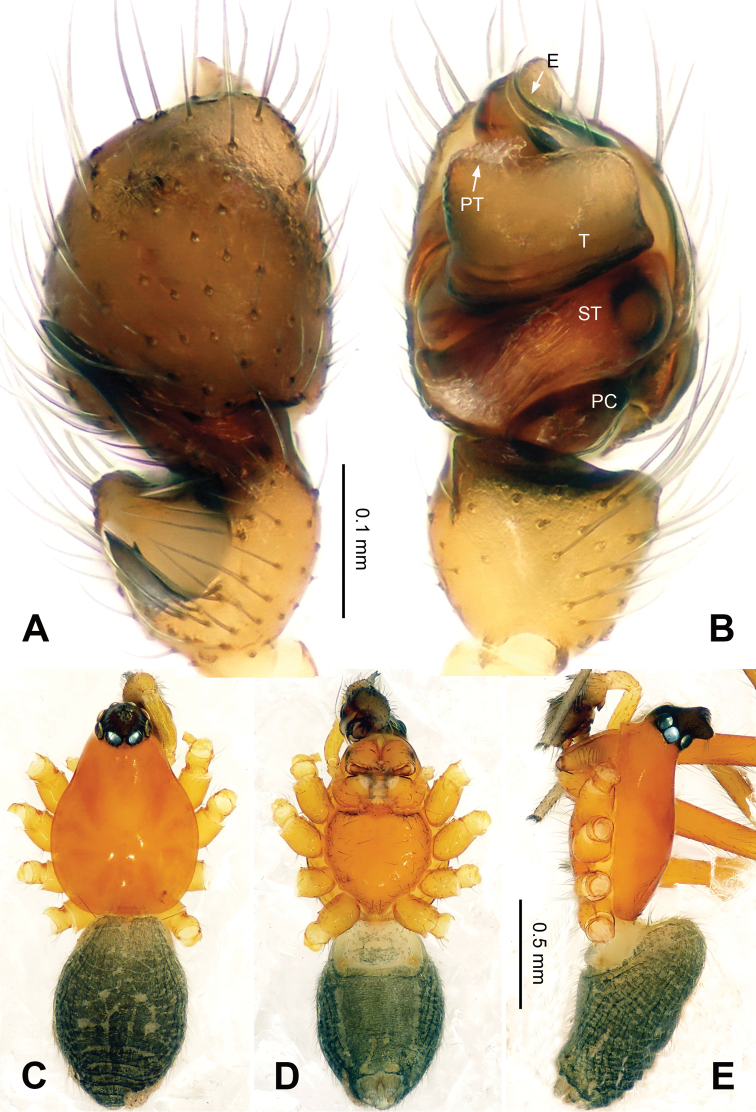
*Paikiniana furcata* sp. n., male holotype. **A** Palp, dorsal view **B** Palp, ventral view **C** Habitus, dorsal view **D** Habitus, ventral view **E** Habitus, lateral view. Scale bars: **B** as **A**, **C** and **D** as **E.**

**Figure 75.**
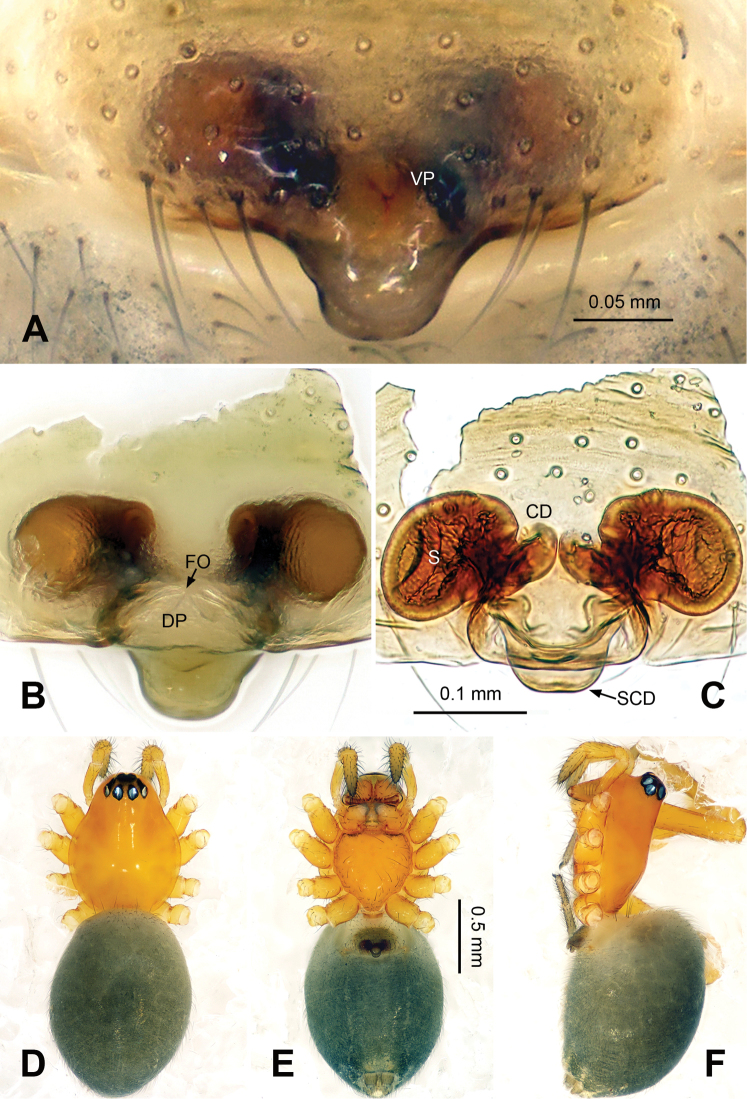
*Paikiniana furcata* sp. n., female paratype. **A** Epigyne, ventral view **B** Epigyne, dorsal view **C** Vulva, dorsal view **D** Habitus, dorsal view **E** Habitus, ventral view **F** Habitus lateral view. Scale bars: **B** as **C**, **D** and **F** as **E.**

**Figure 76.**
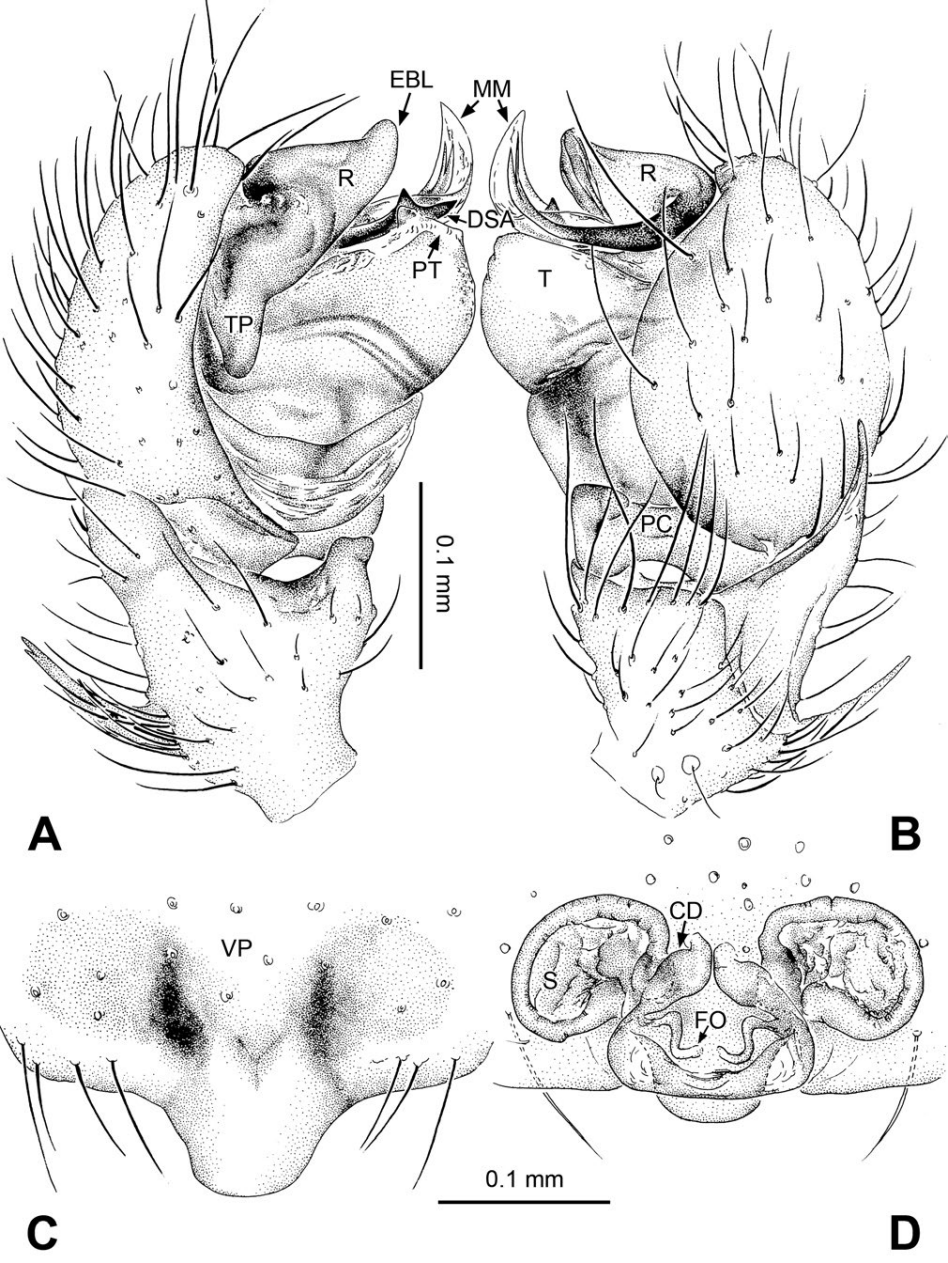
*Paikiniana furcata* sp. n., male holotype (**A–B**) and female paratype (**C–D**). **A** Palp, prolateral view **B** Palp, retrolateral view **C** Epigyne, ventral view **D** Vulva, dorsal view. Scale bar: **B** as **A**, **C** as **D.**

Figs 73 , 74 , 75 , 76

###### Types.

Holotype ♂: CHINA, Yunnan: Mengyang Town: Xishuangbanna nature reserve, 22°09.513'N, 100°53.220'E, elevation ca 894 m, 25.06.2013, tropical seasonal rain forest, sieving. Paratypes 4♂9♀, same data as holotype; 1♂10♀, 22°09.776'N, 100°52.565'E, elevation ca 921 m, 26.06.2013, tropical seasonal rain forest, sieving; 4♂1♀, Guanping Town: Xishuangbanna nature reserve, 22°13.646'N, 100°53.348'E, elevation ca 939 m, 27.06.2013, tropical seasonal rain forest, sieving; 1♀, 22°19.461'N, 100°53.429'E, elevation ca 851 m, 28.06.2013, tropical seasonal rain forest, sieving; 1♂, 22°19.463'N, 100°53.429'E, elevation ca 851 m, 29.06.2013, tropical seasonal rain forest, sieving; 3♂, Menghai Town: Mangunzi Village: Xishuangbanna nature reserve, 22°01.772'N, 100°23.711'E, elevation ca 1187 m, 25.06.2013, secondary tropical seasonal rain forest, sieving.

###### Etymology.

This specific name was taken from the Latin word ‘furcatus’, which means ‘forked’, referring to the bifurcate tip of the distal suprategular process; adjective.

###### Diagnosis.

The male is easily distinguished from other *Paikiniana* species by the forked end of distal suprategular apophysis and the slightly acute angle formed by the two retrolateral tibial apophyses (Fig. 73A–B). The shape of carapace lobe in *Paikiniana furcillata* sp. n. resembles that in *Paikiniana vulgaris* (Oi, 1960) (Oi 1960: fig. 15), but differs from it by the detailed structure of distal suprategular apophysis. The embolic division resembles that in *Paikiniana lurida* (Seo, 1991) (Song and Li 2008: fig. 53), but distinguished from it by the shape of embolic basal lobe and tailpiece (Fig. 73C). The female of the new species has a short projection from the dorsal fig (Fig. 75A), short copulatory ducts and large spermathecae (Fig. 75C), and is similar to *Paikiniana biceps* Song & Li, 2008 (Song and Li 2008: figs 42–45).

###### Description.

Male (holotype). Total length: 1.85. Carapace 0.94 long, 0.69 wide, bright orange, with horn-shaped cephalic lobe. Sternum 0.47 long, 0.53 wide. Clypeus 0.16 high. Chelicerae promargin with 4 teeth, retromargin with 4 teeth. Eye sizes and interdistances: AME 0.06, ALE 0.06, PME 0.06, PLE 0.08, AME-AME/AME 0.33, PME-PME/PME 0.33, AME-ALE/ALE 0.10, PME-PLE/PLE 0.13, coxae IV separated by 1.14 times their width. Length of legs: I 2.88 (0.81, 0.25, 0.78, 0.63, 0.41), II 2.78 (0.78, 0.25, 0.75, 0.60, 0.40), III 2.17 (0.62, 0.20, 0.56, 0.49, 0.30), IV 2.85 (0.80, 0.25, 0.79, 0.65, 0.36). Leg formula: I-IV-II-III. TmI 0.46, TmIV 0.63. Tibial and patellar spine formula: 1-1-1-1. Palp: tibia distally broadened, cone-shaped, with 2 retrolateral trichobothria and 2 tibial apophyses forming an acute angle, the distal one flat, triangular, close to the cymbium, the proximal shorter, directed upward (Fig. 73B); paracymbium hook-shaped (Figs 73B, 76B); protegulum membranous (Fig. 73A); distal suprategular apophysis bifurcated at tip (Fig. 73A); embolic basal lobe small, pointed (Fig. 73C–D); tailpiece of radix leaf-like from the prolateral view (Figs 73A, 76A).

Female (one of paratypes). Total length: 2.22. Carapace 0.97 long, 0.80 wide, same as male in coloration, unmodified. Sternum 0.55 long, 0.58 wide. Clypeus 0.16 high. Chelicerae like in male. Eye sizes and interdistances: AME 0.07, ALE 0.09, PME 0.08, PLE 0.08, AME-AME/AME 0.13, PME-PME/PME 0.38, AME-ALE/ALE 0.11, PME-PLE/PLE 0.25, coxae IV separated by 1.18 times their width. Length of legs: I 3.06 (0.88, 0.28, 0.82, 0.64, 0.44), II 2.92 (0.84, 0.28, 0.78, 0.63, 0.39), III 2.37 (0.66, 0.23, 0.58, 0.56, 0.34), IV 3.01 (0.88, 0.25, 0.81, 0.69, 0.38). Leg formula: I-IV- II-III. TmI 0.48, TmIV 0.36. Spination as in male. Epigyne: ventral fig basally wide, posterior rim narrowed (Fig. 75A); scapoid tongue-shaped; copulatory ducts short, mesally oriented, spermathecae somewhat elliptical, separated by their minimal diameter (Fig. 75B–C).

###### Distribution.

Known only from type localities.

####### Genus *Parameioneta* Locket, 1982

*Parameioneta*: Locket 1982: 375. Type species *Parameioneta spicata* Locket, 1982 from Malaysia.

##### Parameioneta bishou sp. n.

Taxon classificationAnimaliaAraneaeLinyphiidae

http://zoobank.org/4EC61749-6E28-42E7-9199-F4CDE9F83D5C

**Figure 77.**
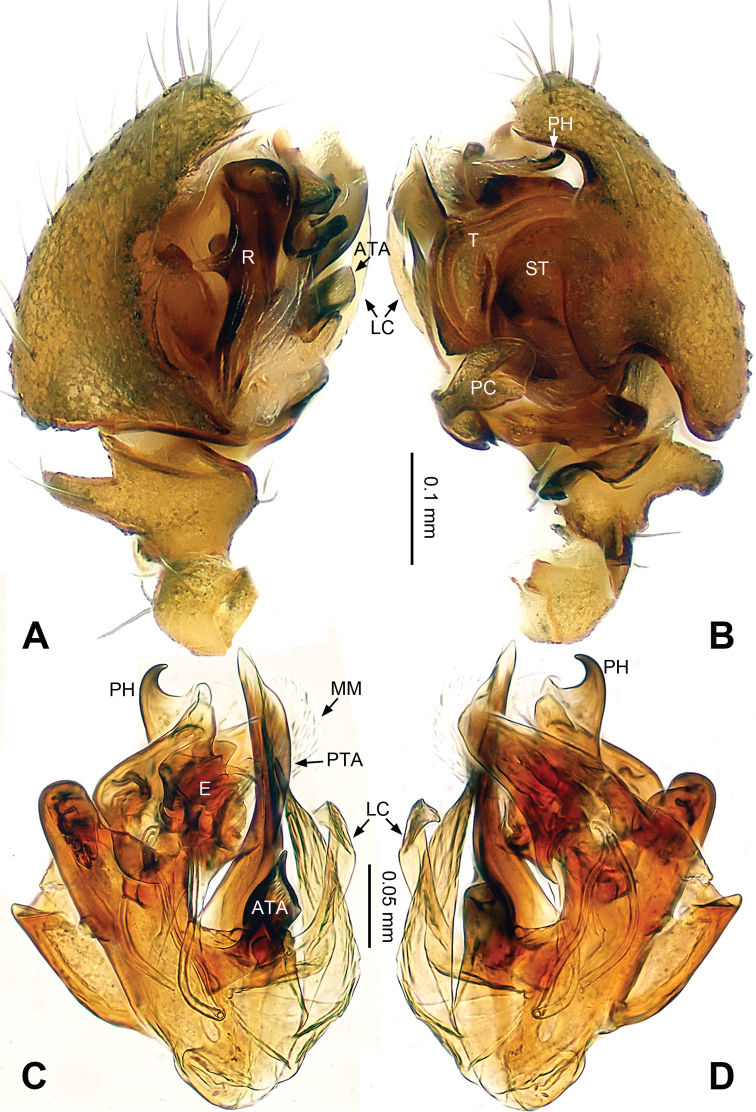
*Parameioneta bishou* sp. n., male holotype. **A** Palp, prolateral view **B** Palp, retrolateral view **C** Embolic division, prolateral view **D** Embolic division, retrolateral view. Scale bars: **A** as **B**, **C** as **D.**

**Figure 78.**
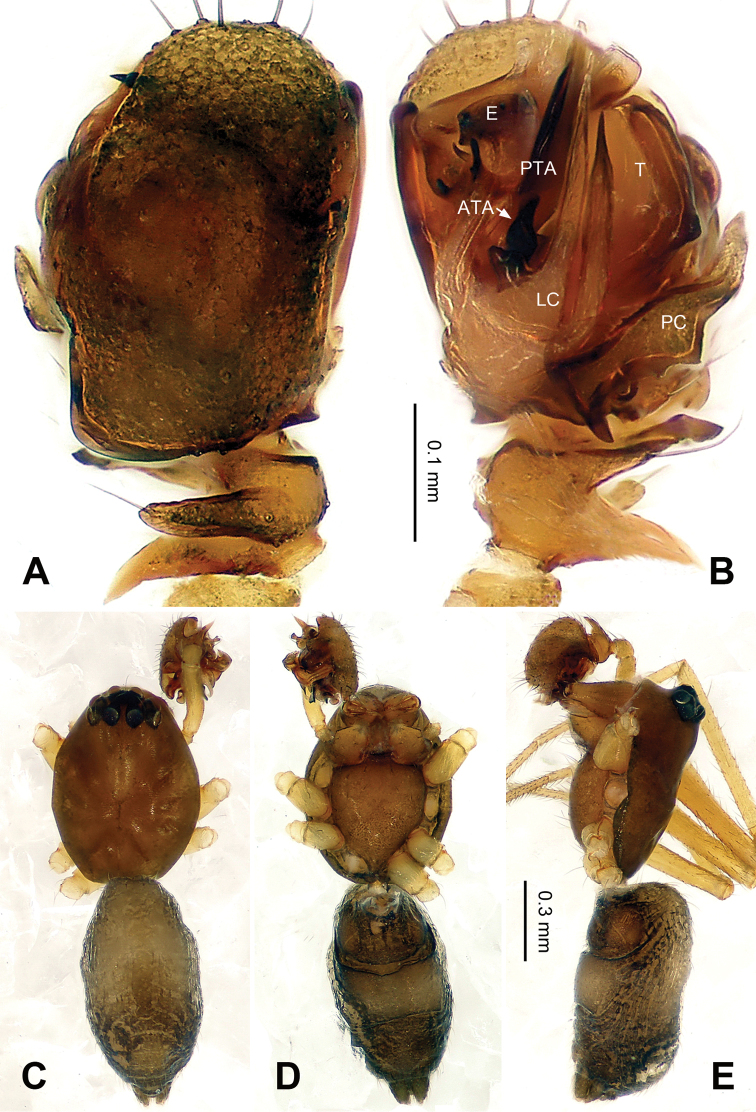
*Parameioneta bishou* sp. n., male holotype. **A** Palp, dorsal view **B** Palp, ventral view **C** Habitus, dorsal view **D** Habitus, ventral view **E** Habitus, lateral view. Scale bars: **A** as **B**, **C** and **D** as **E.**

**Figure 79.**
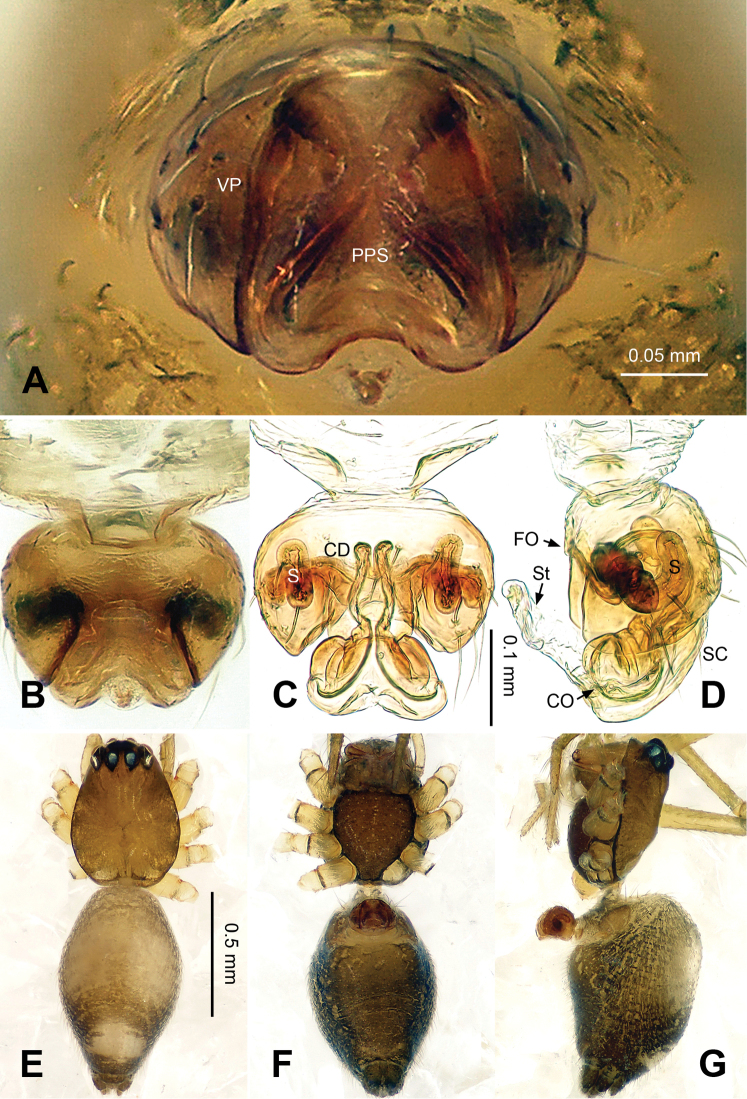
*Parameioneta bishou* sp. n., female paratype. **A** Epigyne, ventral view **B** Epigyne, dorsal view **C** Vulva, dorsal view **D** Vulva, lateral view **E** Habitus, dorsal view **F** Habitus, ventral view **G** Habitus lateral view. Scale bars: **B** and **D** as **C, F** and **G** as **E.**

**Figure 80.**
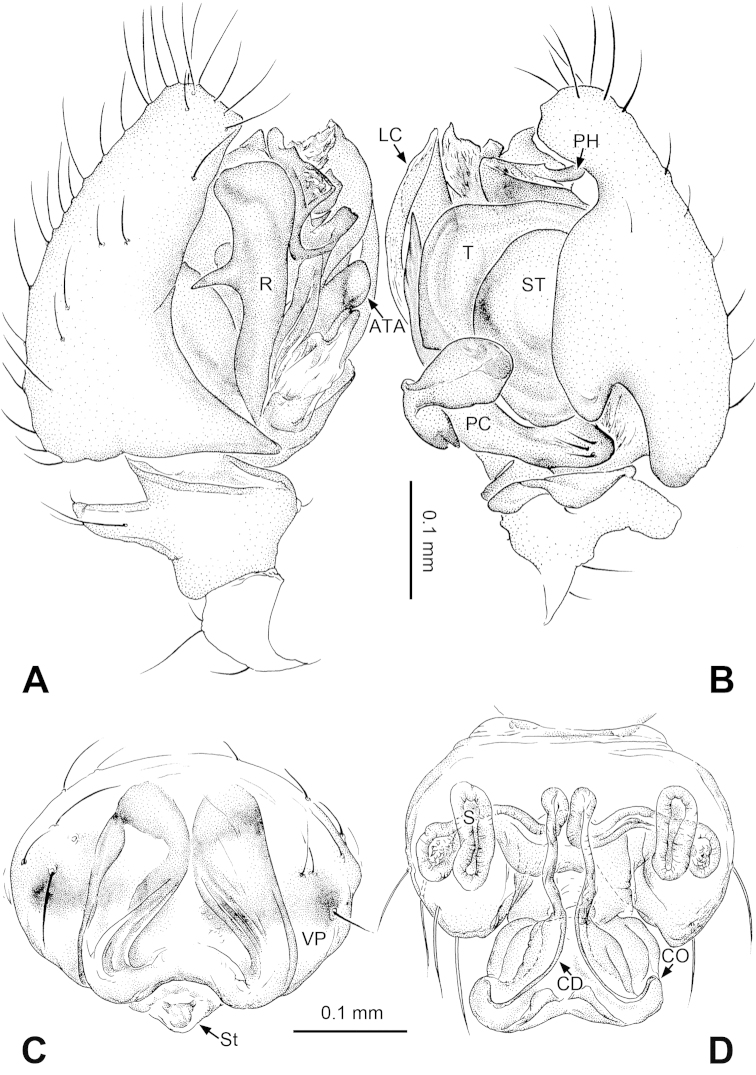
*Parameioneta bishou* sp. n., male holotype (**A–B**) and female paratype (**C–D**). **A** Palp, prolateral view **B** Palp, retrolateral view. **C** Epigyne, ventral view **D** Vulva, dorsal view. Scale bars: **A** as **B**, **D** as **C.**

Figs 77 , 78 , 79 , 80

###### Types.

Holotype ♂: CHINA, Yunnan: Menglun Town: Xishuangbanna Botanical Garden, 5.–12.07.2007, fogging. Paratype 1♂, 21°54.555'N, 101°16.860'E, elevation ca 610 m, 29.11.2009, evergreen forest, fogging. Paratypes 1♀, 21°53.823'N, 101°17.072'E, elevation ca 613 m, 5.–12.03.2007, *Paramichelia baillonii* plantation, hand-collecting; 1♀, 21°54.459'N, 101°16.750'E, elevation ca 640 m, 20.XI.2009, G213 Road, secondary forest, fogging.

###### Etymology.

This name is from the Chinese Pinyin ‘bì shǒu’, meaning ‘short/small sword’, which refers to the sword-shaped tibial dorsal apophysis; noun in apposition.

###### Diagnosis.

Male of new species is similar to *Parameioneta biobata* Tu & Li, 2006, but differs in the following aspects: *Parameioneta biobata* has one curved tibial apophysis (Tu and Li 2006: fig. 7A), while *Parameioneta bishou* sp. n. has three (Figs 77A–B, 78A–B); paracymbium’s apex in *Parameioneta bishou* sp. n. is broad (Fig. 77B), not as slim as in *Parameioneta biobata* (Tu and Li 2006: fig. 7A); lamella characteristica in *Parameioneta bishou* sp. n. has two branches, both of which are long and pointed (Fig. 78B), while in *Parameioneta biobata* one is pointed, the other is broad at tip (Tu and Li 2006: fig. 7C). The female is distinguished from other congeners by the conformation of epigyne.

###### Description.

Male (holotype). Total length: 1.52. Carapace 0.75 long, 0.54 wide, reddish brown. Sternum 0.40 long, 0.33 wide. Clypeus 0.10 high. Chelicerae promargin with 4 teeth, retromargin with 4 teeth. Eye sizes and interdistances: AME 0.05, ALE 0.06, PME 0.06, PLE 0.06, AME-AME/AME 0.60, PME-PME/PME 0.67, AME-ALE/ALE 0.33, PME-PLE/PLE 0.67, coxae IV separated by 0.8 time their width. Length of legs: I 2.68 (0.72, 0.19, 0.70, 0.65, 0.42), II 2.39 (0.65, 0.18, 0.61, 0.55, 0.40), III 1.85 (0.50, 0.15, 0.45, 0.45, 0.30), IV 2.49 (0.69, 0.17, 0.65, 0.60, 0.38). Leg formula: I-IV-II-III. TmI 0.30. Abdomen grey, posterior end slightly darker than the anterior part. Palp: tibia with two retrolateral apophyses and one dorsal apophysis (Figs 77A–B, 78A–B); one tibial retrolateral apophysis sheet-like, slightly curved, the other close to patella, cone-shaped, slightly curved at tip; dorsal tibial apophysis sword-shaped; cymbium with a retrobasal excavation (Figs 77B, 80B); paracymbium ‘V’-shaped, with a pointed process at the turning point, tip broad (Fig. 77B); pit hook short, hooked at tip (Fig. 77D); radix with one small pointed apophysis dorsally (Fig. 77A); anterior terminal apophysis short, flag-like (Fig. 77C); posterior terminal apophysis long, sword-like (Fig. 78C); lamella characteristica with two branches, both long, with pointed apex (Fig. 78B); median membrane with a broad end (Fig. 77C).

Female (one of paratypes). Total length: 1. 38. Carapace 0.58 long, 0.42 wide, brown. Sternum 0.35 long, 0.31 wide. Clypeus 0.06 high. Chelicerae promargin with 3 teeth, retromargin with 4 teeth. Eye sizes and interdistances: AME 0.04, ALE 0.06, PME 0.06, PLE 0.05, AME-AME/AME 1.00, PME-PME/PME 0.50, AME-ALE/ALE 0.33, PME-PLE/PLE 0.40, coxae IV separated by 0.92 their diameters. Length of legs: I 2.40 (0.63, 0.19, 0.63, 0.55, 0.40), II / (0.56, 0.17, /, /, /), III 1.66 (0.45, 0.15, 0.40, 0.40, 0.26), IV 2.24 (0.63, 0.16, 0.60, 0.51, 0.34). Leg formula: I-IV-III. Tm I 0.31, Abdomen light greenish grey, with pale patches. Epigyne: ventral fig oval, wider anteriorly, concave (Fig. 79A); scape ‘S’-shaped, folded, basally broad, with tapering tip (Fig. 79B–D); spermathecae long, narrow, ‘C’-shaped in lateral view (Fig. 79D).

###### Distribution.

Known only from type localities.

##### Parameioneta multifida sp. n.

Taxon classificationAnimaliaAraneaeLinyphiidae

http://zoobank.org/48814403-1A24-4F41-AE7B-3A1A2CFEC754

**Figure 81.**
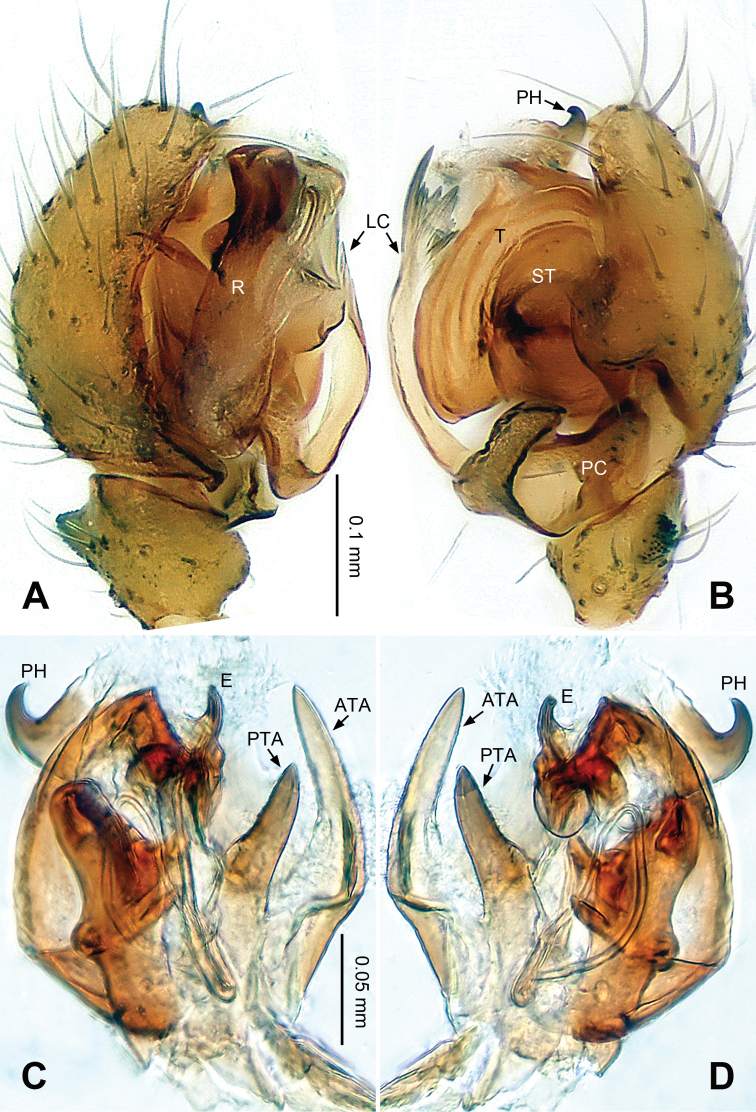
*Parameioneta multifida* sp. n., male holotype. **A** Palp, prolateral view **B** Palp, retrolateral view **C** Embolic division, prolateral view **D** Embolic division, retrolateral view. Scale bars: **B** as **A**, **D** as **C.**

**Figure 82.**
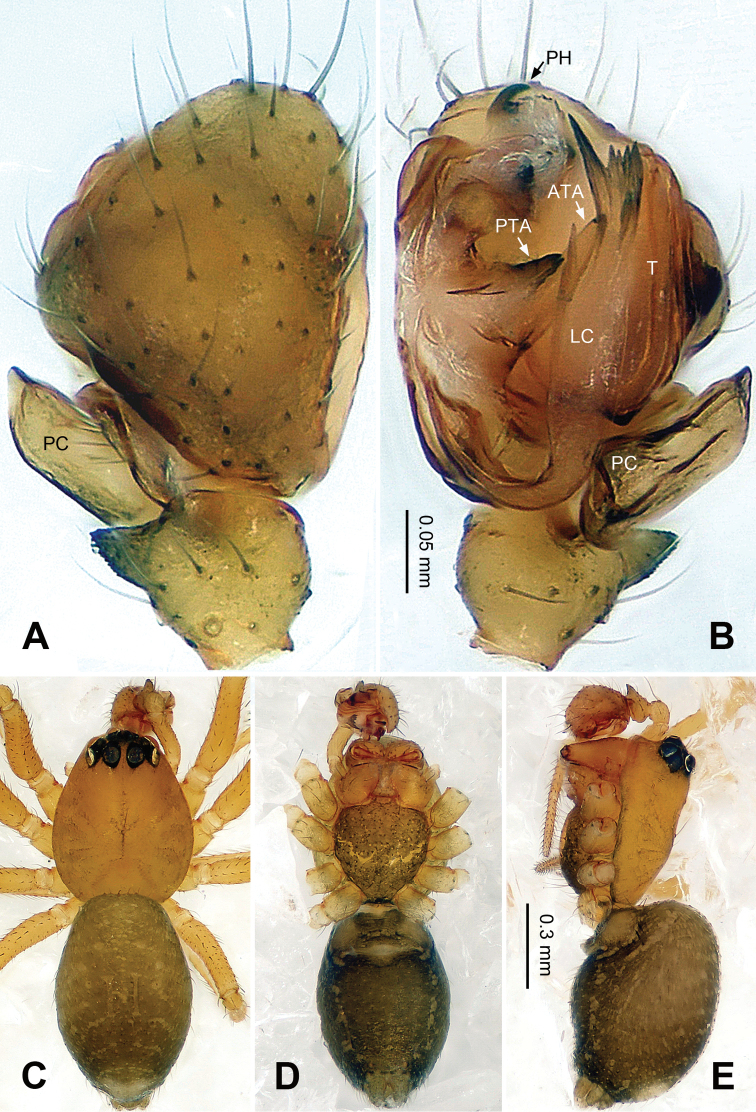
*Parameioneta multifida* sp. n., male holotype. **A** Palp, dorsal view **B** Palp, ventral view **C** Habitus, dorsal view **D** Habitus, ventral view **E** Habitus, lateral view. Scale bars: **A** as **B**, **C** and **D** as **E.**

**Figure 83.**
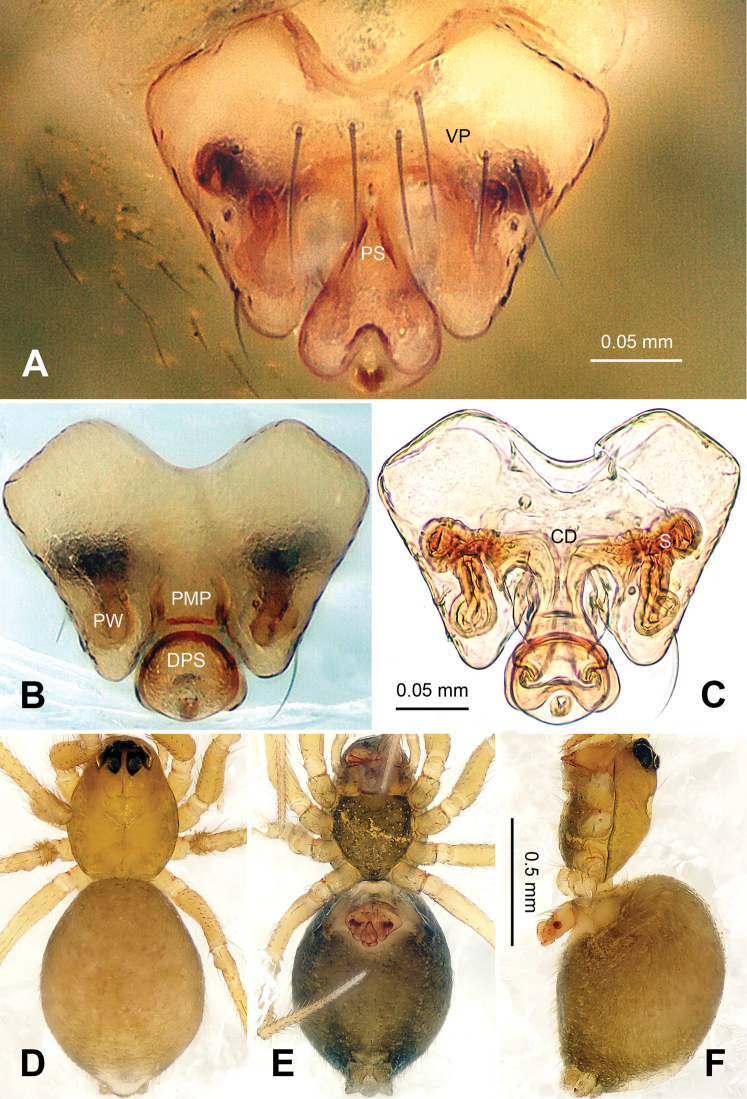
*Parameioneta multifida* sp. n., female paratype. **A** Epigyne, ventral view **B** Epigyne, dorsal view **C** Vulva, dorsal view **D** Habitus, dorsal view **E** Habitus, ventral view **F** Habitus lateral view. Scale bars: **B** as **C**, **D** and **E** as **F.**

**Figure 84.**
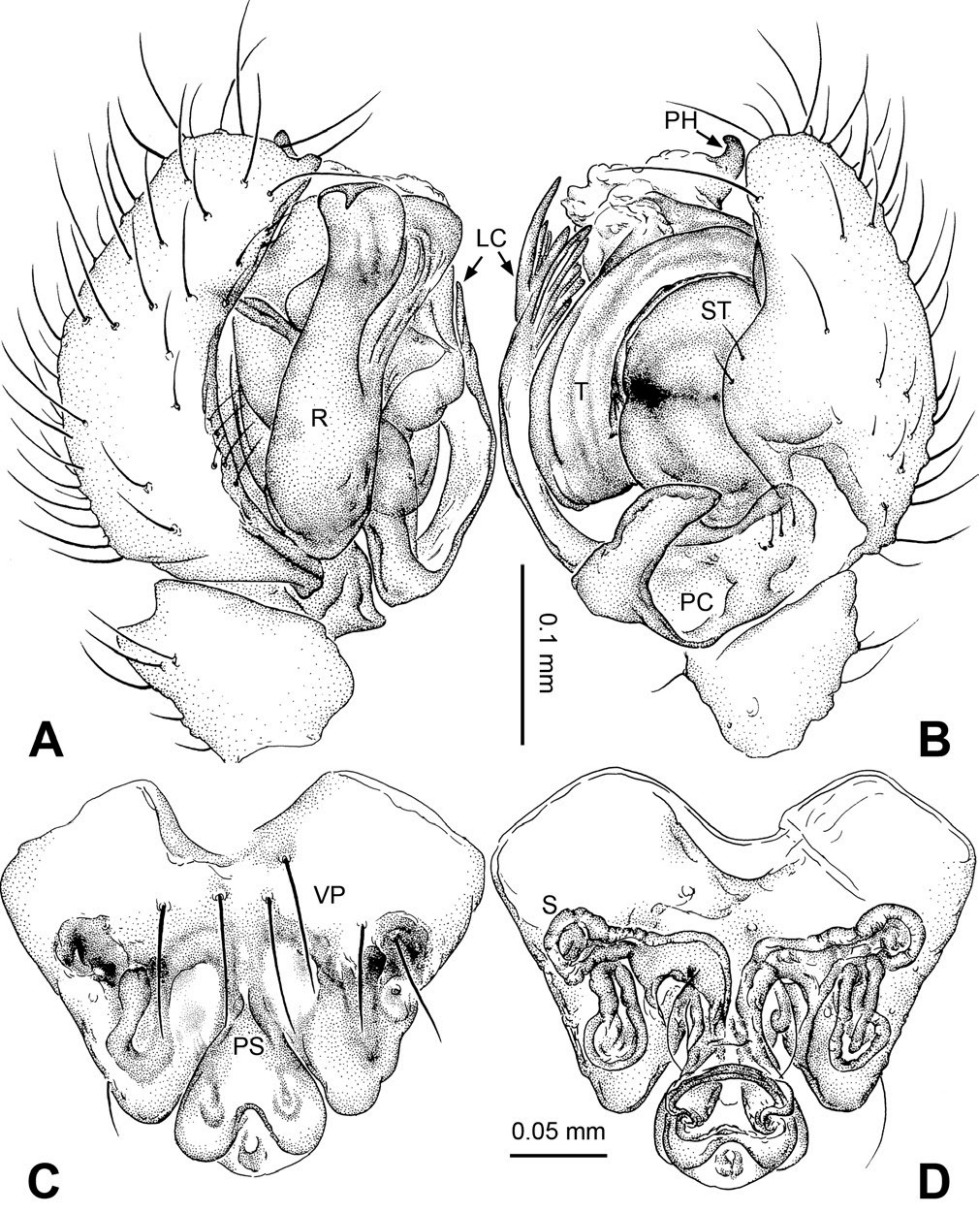
*Parameioneta multifida* sp. n., male holotype (**A–B**) and female paratype (**C–D**). **A** Palp, prolateral view **B** Palp, retrolateral view **C** Epigyne, ventral view **D** Vulva, dorsal view. Scale bars: **A** as **B**, **C** as **D.**

Figs 81 , 82 , 83 , 84

###### Types.

Holotype ♂: Holotype: CHINA, Yunnan: Menglun Town: Xishuangbanna Nature Reserve, 21°53.823'N, 101°17.072'E, elevation ca 613 m, 19.–26.05.2007, *Paramichelia baillonii* plantation, hand-collecting. Paratypes 1♂, same data as holotype; 9♂, 21°53.823'N, 101°17.072'E, elevation ca 613 m, 1.–9.11.2006, *Parameioneta baillonii* plantation, hand-collecting; 1♂, 21°53.823'N, 101°17.072'E, elevation ca 613 m, 1.–15.01.2007, *Parameioneta baillonii* plantation, pitfall traps; 1♀, 21°53.823'N, 101°17.072'E, elevation ca 613 m, 5.–12.01.2007, *Parameioneta baillonii* plantation, hand-collecting; 2♀, 21°53.823'N, 101°17.072'E, elevation ca 613 m, 19.–25.01.2007, *Parameioneta baillonii* plantation, hand-collecting; 1♀, 21°53.823'N, 101°17.072'E, elevation ca 613 m, 5.–12.02.2007, *Parameioneta baillonii* plantation, hand-collecting; 3♀, 21°53.823'N, 101°17.072'E, elevation ca 613 m, 1.–15.04.2007, *Parameioneta baillonii* plantation, pitfall traps; 1♂, 21°53.823'N, 101°17.072'E, elevation ca 613 m, 4.–11.04.2007, *Parameioneta baillonii* plantation, hand-collecting; 17♂, 21°53.823'N, 101°17.072'E, elevation ca 613 m, 1.–15.05.2007, *Parameioneta baillonii* plantation, pitfall traps; 1♀, 21°53.823'N, 101°17.072'E, elevation ca 613 m, 19.–26.05.2007, *Parameioneta baillonii* plantation, hand-collecting.

###### Etymology.

This name originates from the Latin word ‘multi-’ and ‘-fidus’, and the combination means ‘multiply-clefted’, referring to the apex of lamella characteristica; adjective.

###### Diagnosis.

The new species differs from other congeners by the shape of lamella characteristica (Fig. 81A–B) and the tip of embolus (Fig. 81C–D).

###### Description.

Male (holotype). Total length: 1.31. Carapace 0.63 long, 0.55 wide, yellow. Abdomen greenish brown. Sternum 0.38 long, 0.36 wide. Clypeus 0.13 high. Chelicerae promargin with 4 teeth, retromargin with 2 teeth. Eye sizes and interdistances: AME 0.07, ALE 0.07, PME 0.06, PLE 0.07, AME-AME/AME 0.10, PME-PME/PME 0.50, AME-ALE/ALE 0.29, PME-PLE/PLE 0.43, coxae IV separated by their width. Length of legs: I 2.34 (0.60, 0.19, 0.63, 0.53, 0.39), II 2.13 (0.56, 0.18, 0.55, 0.48, 0.36), III 1.70 (0.44, 0.16, 0.40, 0.40, 0.30), IV 2.24 (0.58, 0.18, 0.60, 0.50, 0.38). Leg formula: I-IV-II-III. TmI 0.21. Palp: tibia with one retrolateral trichobothrium and one dorsal trichobothrium; tibial retrolateral apophysis with a patch of small tubercles (Figs 81B, 92A); paracymbium ‘C’-shaped, mesally broad (Fig. 81B); pit hook on distal part of suprategulum, short (Fig. 81C–D); lamella characteristica long, narrow, belt-like, then directed distally (Figs 81A–B, 82B); anterior terminal apophysis long, with tapering tip; posterior terminal apophysis short and erect (Fig. 81C–D); median membrane in ill-defined shape, covering the tip of embolus (Figs 81B, 82B); embolus with a pointed tip (Fig. 81D).

Female. Total length: 1.41. Carapace 0.63 long, 0.44 wide, yellow. Abdomen tanned. Sternum 0.34 long, 0.31 wide. Clypeus 0.08 high. Chelicerae promargin with 4 teeth, retromargin with 3 teeth. Eye sizes and interdistances: AME 0.04, ALE 0.05, PME 0.06, PLE 0.04, AME-AME/AME 0.50, PME-PME/PME 0.50, AME-ALE/ALE 0.40, PME-PLE/PLE 0.50, coxae IV separated by 1.44 their diameters. Length of legs: I 1.96 (0.50, 0.16, 0.52, 0.44, 0.34), II 1.80 (0.47, 0.16, 0.45, 0.42, 0.30), III 1.72 (0.39, 0.16, 0.45, 0.42, 0.30), IV 1.93 (0.53, 0.15, 0.50, 0.45, 0.30). Leg formula: I-IV-II-III. TmI 0.32, TmIV 0.38. Epigyne: trapezoidal; scape long and folded (Fig. 83B–C); in lateral view, proscape bulged; copulatory ducts going along the scape, then turning aside to connect with spermathecae (Figs 83C, 84B); spermathecae long, with two cells (Fig. 83C).

###### Distribution.

Known only from type localities.

##### Parameioneta tricolorata sp. n.

Taxon classificationAnimaliaAraneaeLinyphiidae

http://zoobank.org/16A8893C-B7E1-4A46-A20C-A787C4293D3D

**Figure 85.**
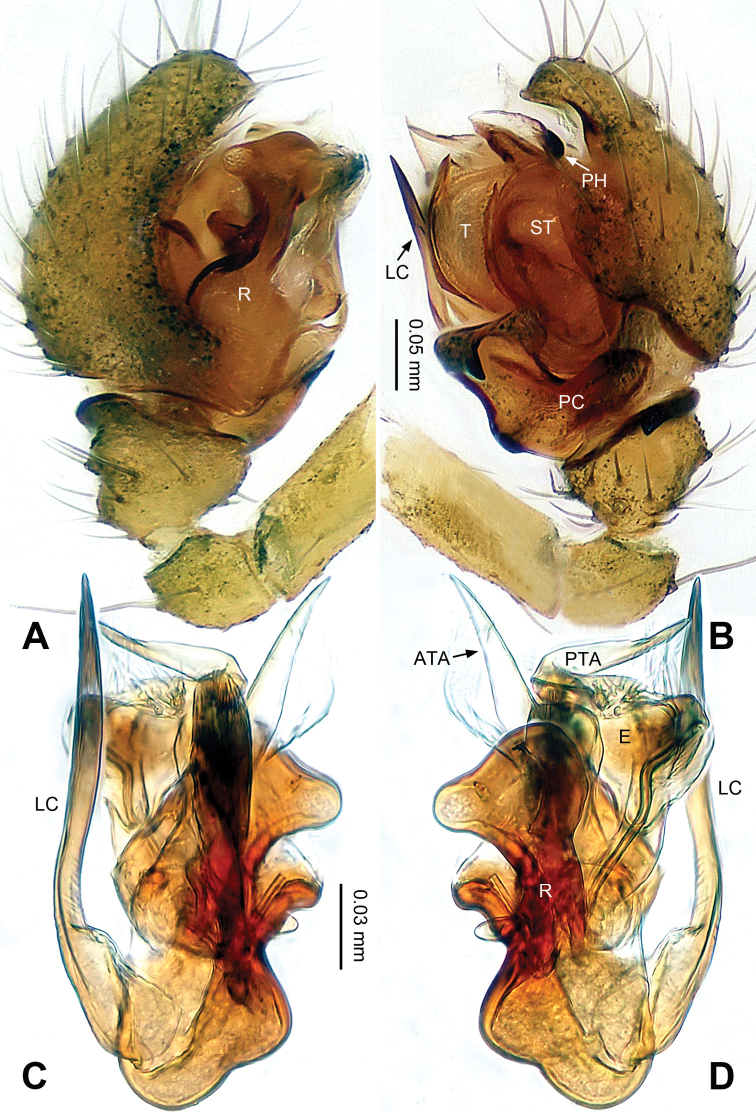
*Parameioneta tricolorata* sp. n., male holotype. **A** Palp, prolateral view **B** Palp, retrolateral view **C** Embolic division, retrolateral view **D** Embolic division, prolateral view. Scale bars: **A** as **B**, **D** as **C.**

**Figure 86.**
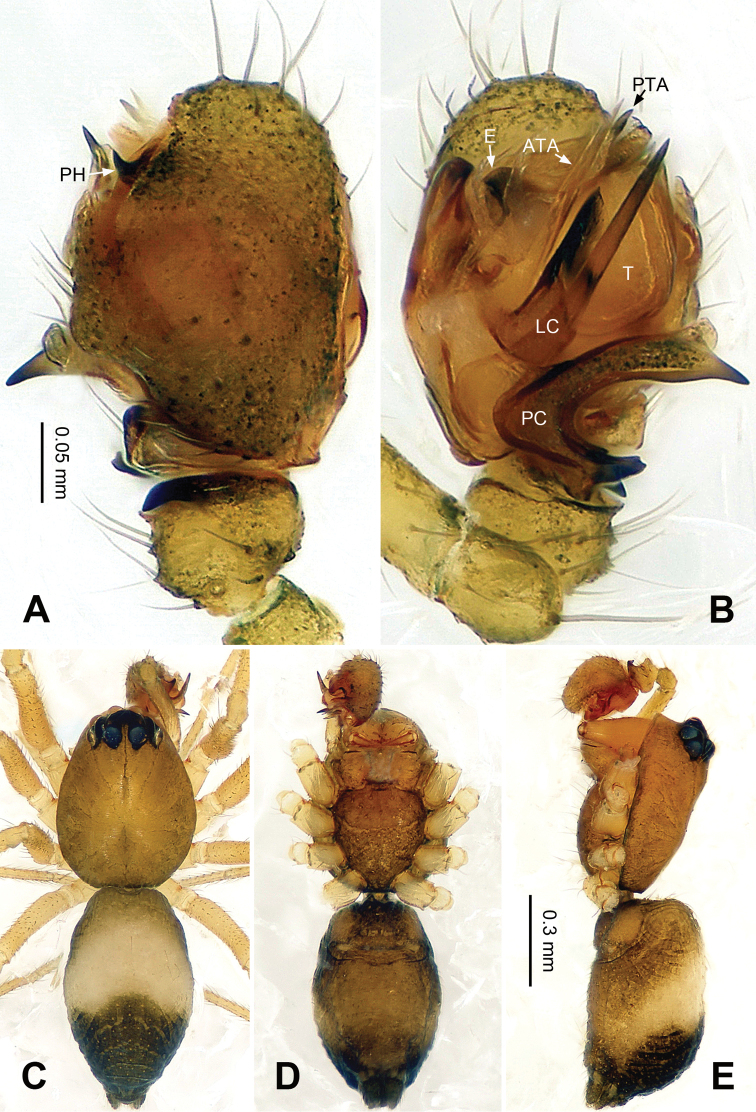
*Parameioneta tricolorata* sp. n., male holotype. **A** Palp, dorsal view **B** Palp, ventral view **C** Habitus, dorsal view **D** Habitus, ventral view **E** Habitus, lateral view. Scale bars: **B** as **A**, **C** and **D** as **E.**

**Figure 87.**
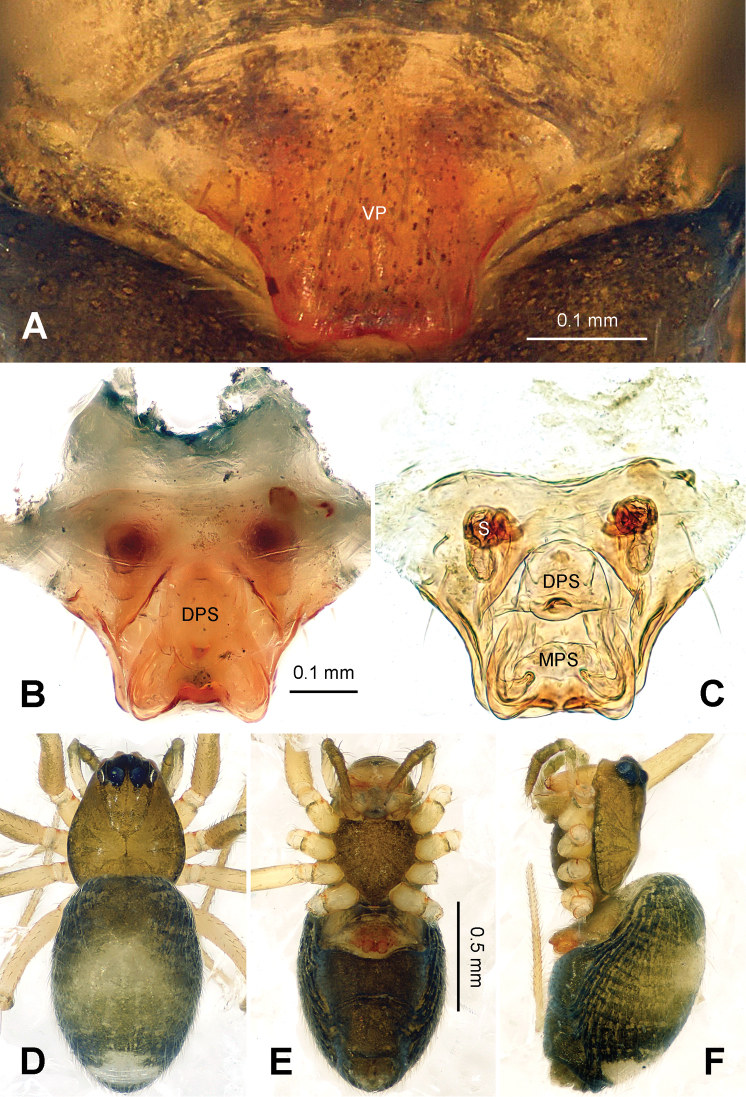
*Parameioneta tricolorata* sp. n., female paratype. **A** Epigyne, ventral view **B** Epigyne, dorsal view **C** Vulva, dorsal view **D** Habitus, dorsal view **E** Habitus, ventral view **F** Habitus lateral view. Scale bars: **C** as **B**, **D** and **F** as **E.**

**Figure 88.**
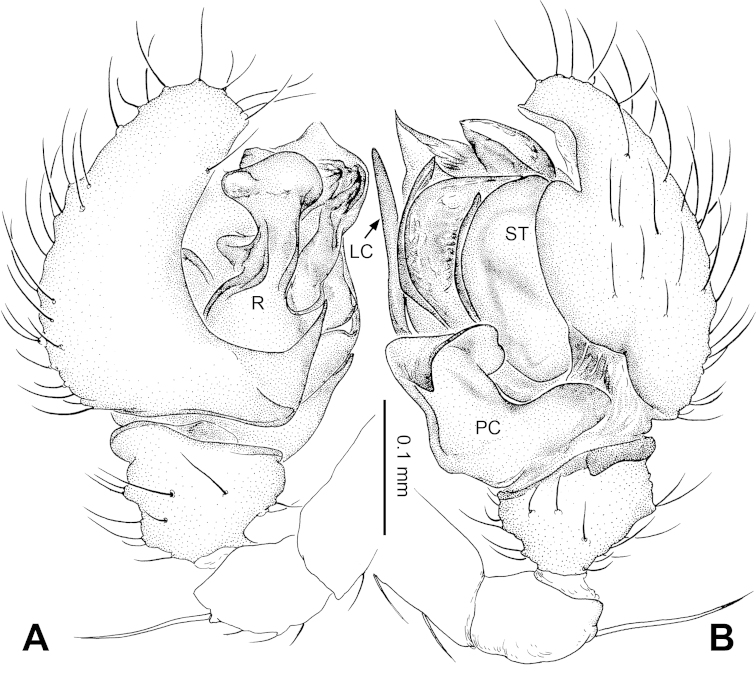
*Parameioneta tricolorata* sp. n., male holotype. **A** Palp, prolateral view **B** Palp, retrolateral view. Scale bar: **A** as **B.**

Figs 85 , 86 , 87 , 88

###### Types.

Holotype ♂: CHINA, Yunnan: Menglun Town: Xishuangbanna Nature Reserve, 21°53.823'N, 101°17.072'E, elevation ca 613 m, 19.–26.05.2007, *Paramichelia baillonii* plantation, hand-collecting. Paratypes 1♂, 21°54.684'N, 101°16.319'E, elevation ca 585 m, 5.–12.10.2006, rubber tree plantation, hand-collecting; 1♀, 21°54.772'N, 101°16.043'E, elevation ca 556 m, 5.–12.12.2006, *Parameioneta baillonii* plantation, hand-collecting; 21°54.035'N, 101°16.500'E, elevation ca 558 m, 5.–12.01.2007, primary tropical seasonal rain forest, hand-collecting; 1♂, 21°54.684'N, 101°16.319'E, elevation ca 585 m, 16.–31.01.2007, rubber tree plantation, hand-collecting; 3♂, 21°54.684'N, 101°16.319'E, elevation ca 585 m, 1.–15.03.2007, rubber tree plantation, pitfall traps; 4♂, 21°54.498'N, 101°16.326'E, elevation ca 586 m, 1.–15.04. 2007, rubber tree plantation, pitfall traps; 1♀, 21°54.498'N, 101°16.326'E, elevation ca 586 m, 1.–15.04.2007, rubber tree plantation, trunk traps; 1♂, 21°53.823'N, 101°17.072'E, elevation ca 613 m, 4.–11.05.2007, *Parameioneta baillonii* plantation, hand-collecting; 1♂, 21°55.551'N, 101°16.923'E, elevation ca 561 m, 10.–20.06.2007, rubber-tea plantation, hand-collecting; 1♂, 21°54.646'N, 101°16.257'E, elevation ca 572 m, 1.–9.10.2007, rubber-tea plantation, pitfall traps; 1♂, 21°54.705'N, 101°16.898'E, elevation ca 656 m, 13.11.2009, Lvshilin tropical rain forest, fogging.

###### Etymology.

This name comes from the Latin ‘tres-’, meaning ‘three’, and the Latin word ‘coloratus’, meaning ‘colored’, and the combination refers to the three transversal bands of different colors on the dorsum of the abdomen; adjective.

###### Diagnosis.

This species is similar to *Parameioneta biobata* Tu & Li, 2006 by the shape of embolus (Tu and Li 2006: fig. 8C; Fig. 85D) and the median membrane (Tu and Li 2006: fig. 8B); differs in the following aspects: *Parameioneta tricolorata* sp. n. has a much smaller tibial dorsal apophysis (Fig. 85B); the shape of radix is different in two species (Tu and Li 2006: fig. 7B; Fig. 85A); the structure of lamella characteristica differs in two species. The color pattern of abdomen in *Parameioneta tricolorata* sp. n. (Fig. 86C) is quite similar to the type species *Parameioneta spicata* Locket, 1982 (Locket 1982: fig. 72). The epigyne of female resembles greatly that of *Parameioneta spicata* (Locket 1982: fig. 78), but differs in the wider distal end of the scape.

###### Description.

Male (holotype). Total length: 1.31. Carapace 0.59 long, 0.47 wide, unmodified, earthy yellow. Sternum 0.38 long, 0.30 wide. Clypeus 0.11 high. Chelicerae promargin with 5 teeth, retromargin with 4 teeth. Eye sizes and interdistances: AME 0.04, ALE 0.07, PME 0.06, PLE 0.06, AME-AME/AME 0.50, PME-PME/PME 0.50, AME-ALE/ALE 0.14, PME-PLE/PLE 0.33, coxae IV separated by their width. Length of legs: I 2.60 (0.68, 0.18, 0.70, 0.60, 0.44), II 2.23 (0.60, 0.16, 0.58, 0.50, 0.39), III 1.71 (0.46, 0.15, 0.40, 0.40, 0.30), IV 2.29 (0.63, 0.15, 0.60, 0.53, 0.38). Leg formula: I-IV-II-III. TmI 0.33. Abdomen with three belts of different colors: from light grey to pale to dark grey with green tone. Palp: tibia with one retrolateral and one dorsal trichobothrium; dorsal tibial apophysis small, hooked (Fig. 85B); paracymbium ‘C’-shaped (Figs 85B, 86B); pit hook short, hooked at tip (Fig. 86A); lamella characteristica with two branches, one long, tapered at tip, the other broad and shorter, with a pointed apex (Fig. 86B); embolus stout, with opening at tip (Fig. 85D).

Female (paratype). Total length: 1.54. Carapace 0.63 long, 0.52 wide, dark yellow. Sternum 0.34 long, 0.36 wide. Clypeus 0.16 high. Chelicerae promargin with 7 teeth, retromargin with 4 teeth. Eye sizes and interdistances: AME 0.03, ALE 0.08, PME 0.08, PLE 0.07, AME-AME/AME 1.00, PME-PME/PME 0.38, AME-ALE/ALE 0.38, PME-PLE/PLE 0.29, coxae IV separated by 0.84 time their width. Length of legs: I 2.22 (0.50, 0.20, 0.63, 0.52, 0.37), II 2.38 (0.64, 0.20, 0.60, 0.56, 0.38), III 1.98 (0.53, 0.19, 0.44, 0.46, 0.36), IV 2.49 (0.70, 0.18, 0.63, 0.60, 0.38). Leg formula: IV-II-I-III. TmI 0.29, TmIV absent. Abdomen greyish green with beige patches on the dorsum. Epigyne: trapezoidal with narrower posterior end (Fig. 87A); scape long, distal part with a broad tip (Fig. 87C); spermathecae with two chambers (Fig. 87C).

###### Distribution.

Known only from type localities.

####### Genus *Prosoponoides* Millidge & Russell-Smith, 1992

*Prosoponoides*: Millidge and Russell–Smith 1992: 1369. Type species *Prosoponoides hamatus* Millidge & Russell-Smith, 1992 from Sumatra.

##### Prosoponoides hamatus

Taxon classificationAnimaliaAraneaeLinyphiidae

Millidge & Russell-Smith, 1992

Prosoponoides hamatus : Millidge and Russell–Smith 1992: 1371, figs 1–4, 8–11. (♂♀).

###### Material examined.

1♂, CHINA, Yunnan: Menglun Town: Xishuangbanna Nature Reserve, 21°57.809'N, 101°12.173'E, elevation ca 888 m, 4.08.2007, secondary tropical montane evergreen broad-leaved forest, fogging; 2♂, 21°57.669'N, 101°11.893'E, elevation ca 790 m, 7.08.2007, primary tropical seasonal rain forest, fogging; 1♂, 21°54.725'N, 101°13.261'E, elevation ca 734 m, 8.08.2007, primary tropical seasonal rain forest, fogging.

###### Distribution.

China, Sumatra.

####### Genus *Saitonia* Eskov, 1992

*Saitonia*: Eskov 1992: 49. Type species *Araeoncus muscus* Saito, 1989 from Japan.

##### Saitonia kawaguchikonis

Taxon classificationAnimaliaAraneaeLinyphiidae

Saito & Ono, 2001

Saitonia kawaguchikonis : Saito and Ono 2001: 39, figs 81–85 (♂♀).

###### Material examined.

1♀, CHINA, Yunnan: Menglun Town: Xishuangbanna Tropical Botanical Garden, 21°54.684'N, 101°16.319'E, elevation ca 585 m, 5.–12.11.2006, rubber tree plantation, hand-collecting; 1♂, 21°54.463'N, 101°15.978'E, elevation ca 569 m, 5.–12.12.2006, rubber tree plantation, hand-collecting; 1♀, 21°54.408'N, 101°16.326'E, elevation ca 586 m, 19.–25.12.2006, rubber tree plantation, hand-collecting; 2♀, 21°54.498'N, 101°16.326'E, elevation ca 586 m, 10.–20.01.2007, rubber tree plantation, hand-collecting; 1♂ 21°54.463'N, 101°15.978'E, elevation ca 569 m, 16.–28.02.2007, rubber tree plantation, pitfall traps; 3♂, 21°54.463'N, 101°15.978'E, elevation ca 569 m, 16.–31.05.2007, rubber tree plantation, pitfall traps.

###### Distribution.

China, Japan.

##### Genus Smerasia gen. n.

Taxon classificationAnimaliaAraneaeLinyphiidae

http://zoobank.org/B33D870C-7F08-4BB0-9D71-93AA7366729D

###### Type species.

*Smerasia obscurus* sp. n.

###### Etymology.

The generic name is a combination of ‘smer’ and ‘Asia’, ‘smer’ is the first a few letters of the genus name *Smermisia*, which is similar to this new genus by the conformation of the male palp. Gender is masculine.

###### Diagnosis.

**Figure 89.**
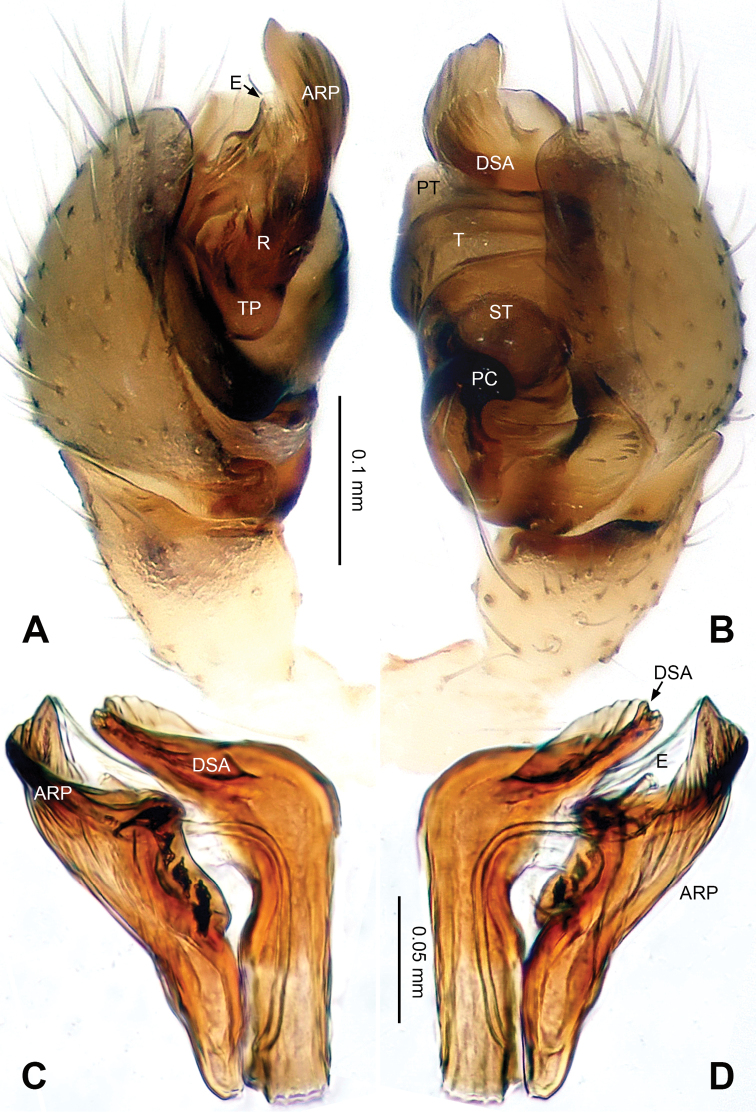
*Smerasia obscurus* sp. n., male holotype. **A** Palp, prolateral view **B** Palp, retrolateral view **C** Embolic division, retrolateral view **D** Embolic division, prolateral view. Scale bars: **B** as **A**, **C** as **D.**

This genus resembles Neotropical genera *Smermisia* Simon, 1894 and *Myrmecomelix* Millidge, 1993 in the general appearence of the male palp and the conformation of embolic division (Miller 2007). They all have a fig-like radix, and an erect anterior radical process. *Smerasia* gen. n. differs from similar genera by having short and weakly sclerotized, less pointed, membranous embolus (Fig. 89C–D) (Miller 2007: figs 63–64, 104), and the paracymbium in *Smerasia* gen. n. is attenuated at apex and much more curved in general (Fig. 89B). The epigyne in *Smerasia* gen. n. is wide and with a convex dorsal fig (Fig. 91A), which is quite different from that in *Smermisia* and *Myrmecomelix*.

###### Description.

Small sized Erigoninae. Carapace uniformly yellow. Male with modified carapace: PME area elevated with sulci behind PME and pit in them. Carapace unmodified in female. Abdomen pale with dark spinnerets area. Chelicerae in male with 5 promarginal and 5 retromarginal teeth, while female with 4 promarginal and 4 retromarginal teeth. Tibial spine formula: 2-2-1-1. TmI ca 0.80, TmIV ca 0.70.

Male palp: tibia with one short and straight dorsal apophysis; paracymbium with black, attenuated apex (Fig. 89B); protegulum small (Figs 89B, 92B); radix simple, fig-like, with short, broad tailpiece (Figs 89A, 92A); anterior radical process straight, curved, with a pointed tip (Figs 89A, 92A); distal suprategular apophysis with a broad tip (Fig. 89C–D); embolus short, inconspicuous (Fig. 89A).

Epigyne: ventral fig wide, with rounded posterior margin (Figs 91A); dorsal fig mesally concave; copulatory ducts straight (Figs 91C); spermathecae with multi-chambers, the bigger one elliptical (Fig. 91C); fertilization ducts rather short (Fig. 91B).

###### Species composition.

Type species only: *Smerasia obscurus* sp. n.

###### Distribution.

China.

##### Smerasia obscurus sp. n.

Taxon classificationAnimaliaAraneaeLinyphiidae

http://zoobank.org/99627A3A-2D72-4986-850A-6E9470BEF914

**Figure 90.**
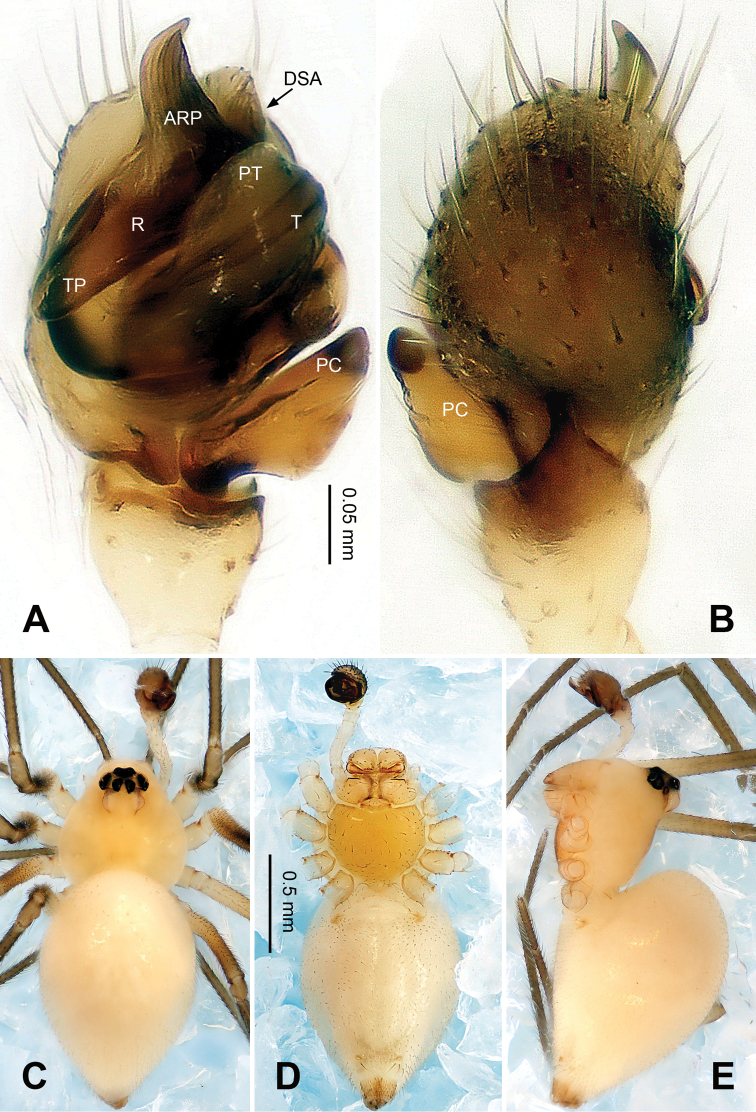
*Smerasia obscurus* sp. n., male holotype. **A** Palp, dorsal view **B** Palp, ventral view **C** Habitus, dorsal view **D** Habitus, ventral view **E** Habitus, lateral view. Scale bars: **B** as **A**, **C** and **E** as **D.**

**Figure 91.**
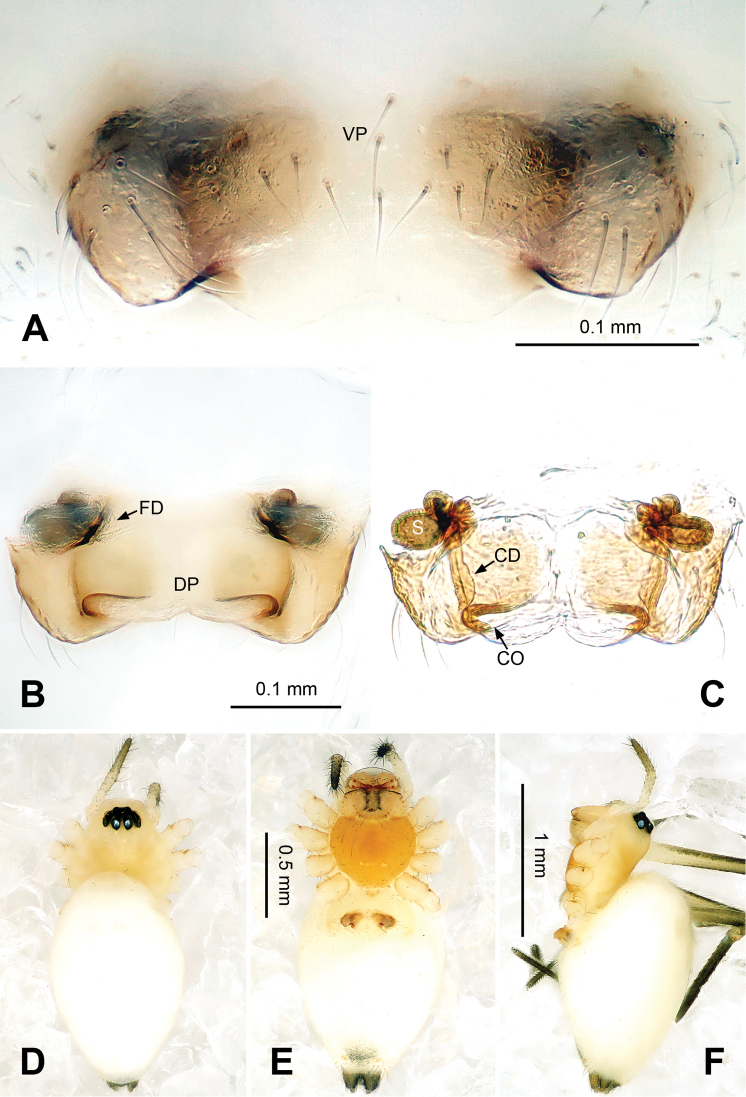
*Smerasia obscurus* sp. n., female paratype. **A** Epigyne, ventral view **B** Epigyne, dorsal view **C** Vulva, dorsal view **D** Habitus, dorsal view **E** Habitus, ventral view **F** Habitus lateral view. Scale bars: **C** as **B**, **D** as **F.**

**Figure 92.**
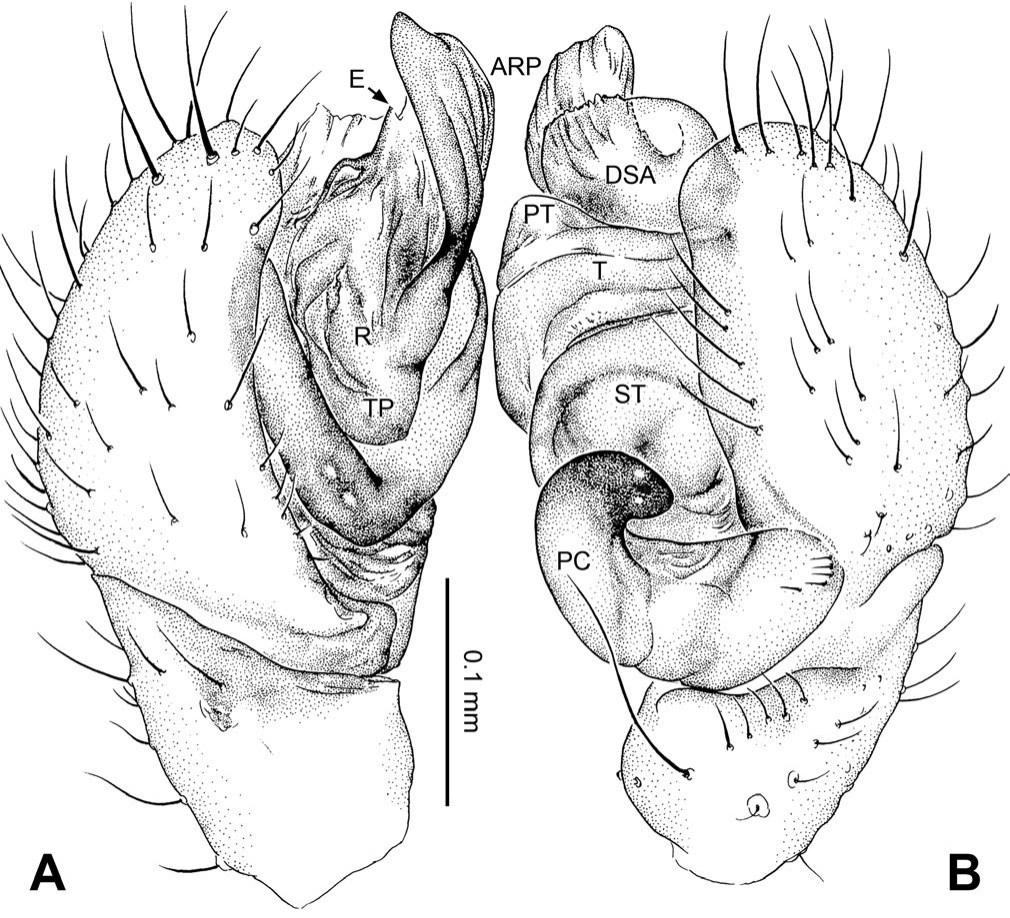
*Smerasia obscurus* sp. n., male holotype. **A** Palp, prolateral view **B** Palp, retrolateral view. Scale bar: **B** as **A.**

Figs 89 , 90 , 91 , 92

###### Types.

Holotype ♂: CHINA, Yunnan: Menglun Town: Xishuangbanna Nature Reserve, 21°57.784'N, 101°11.947'E, elevation ca 895 m, 6.08.2007, secondary tropical montane evergreen broad-leaved forest, fogging. Paratypes 5♂, same data as holotype; 2♀, 21°55.035'N, 101°16.500'E, elevation ca 558 m, 22.07.2007, primary tropical seasonal forest, fogging; 5♂1♀, 21°57.809'N, 101°12.173'E, elevation ca 888 m, 4.08.2007, secondary tropical montane evergreen broad-leaved forest, fogging; 3♂ 1♀, 21°54.813'N, 101°12.634'E, elevation ca 876 m, 5.08.2007, secondary tropical montane evergreen broad-leaved forest, fogging; 6♂1♀, 21°54.767'N, 101°11.431'E, elevation ca 880 m, 6.08.2007, secondary tropical montane evergreen broad-leaved forest, fogging; 2♂, 21°57.669'N, 101°11.893'E, elevation ca 790 m, 7.08.2007, primary tropical seasonal rain forest, fogging; 8♂, 21°54.725'N, 101°13.261'E, elevation ca 734 m, 8.08.2007, primary tropical seasonal rain forest, fogging; 1♂, 21°54.200'N, 101°16.923'E, elevation ca 608 m, 18.08.2007, *Paramechelia baillonii* plantation, fogging.

###### Etymology.

This name is derived from the Latin word ‘obscurus’, which means ‘obscure’, referring to the ambiguous shape of the embolus; adjective.

###### Diagnosis.

See diagnosis of the genus.

###### Description.

Male (holotype). Total length: 1.76. Carapace 0.70 long, 0.64 wide, PME area mildly elevated, post-ocular sulci with pits stretching along the base of lobe laterally, light yellow. Sternum 0.41 long, 0.50 wide. Clypeus 0.23 high. Eye sizes and interdistances: AME 0.05, ALE 0.06, PME 0.05, PLE 0.06, AME-AME/AME 0.40, PME-PME/PME 0.40, AME-ALE/ALE 0.33, PME-PLE/PLE 1.00, coxae IV separated by 1.92 times their width. Length of legs: I 3.52 (0.95, 0.23, 0.88, 0.96, 0.50), II 3.56 (0.94, 0.22, 0.91, 0.94, 0.55), III 2.62 (0.72, 0.20, 0.60, 0.70, 0.40), IV 3.28 (0.90, 0.20, 0.78, 0.90, 0.50). Leg formula: II-I-III-IV. TmI 0.82, TmIV 0.73. Abdomen pale. Palp: see description of the genus.

Female (one of paratypes). Total length: 1.88. Carapace 0.75 long, 0.75 wide, unmodified, yellow. Sternum 0.42 long, 0.50 wide. Clypeus 0.16 high. Chelicerae promargin with 4 teeth, retromargin with 4 teeth. Eye sizes and interdistances: AME 0.05, ALE 0.06, PME 0.06, PLE 0.05, AME-AME/AME 0.63, PME-PME/PME 0.50, AME-ALE/ALE 0.33, PME-PLE/PLE 0.33, coxae IV separated by 2.00 times their width. Length of legs: I 3.84 (1.02, 0.23, 1.02, 1.02, 0.55), II 3.98 (1.09, 0.23, 1.02, 1.08, 0.56), III 2.89 (0.80, 0.20, 0.64, 0.78, 0.47), IV 3.44 (0.98, 0.22, 0.81, 0.94, 0.49). Leg formula: IV-I-II-III. TmI 0.84, TmIV 0.75. Abdomen light yellow, with dark spinnerets. Epigyne: see description of the genus.

###### Distribution.

Known only from type localities.

####### Genus *Tapinopa* Westring, 1851

*Tapinopa*: Westring 1851: 38. Type species *Linyphia longidens* Wider, 1834 from Palearctic.

##### Tapinopa undata sp. n.

Taxon classificationAnimaliaAraneaeLinyphiidae

http://zoobank.org/7A979C10-F4E1-4A67-B517-091C1F130C49

Figs 93 , 94 , 95

###### Types.

Holotype ♂: CHINA, Yunnan: Menglun Town: Xishuangbanna Tropical Botanical Garden, 21°54.200'N, 101°16.923'E, elevation ca 608 m, 5.–12.11.2006, *Paramichelia baillonii* plantation, hand-collecting. Paratypes 1♂, 21°54.200'N, 101°16.923'E, elevation ca 608 m, 1.–15.07.2006, *Parameioneta baillonii* plantation, hand-collecting; 1♂, 21°54.772'N, 101°16.043'E, elevation ca 556 m, 19.–25.11.2006, *Parameioneta baillonii* plantation, hand-collecting; 1♂, 21°54.772'N, 101°16.043'E, elevation ca 556 m, 19.–25.12.2006, *Parameioneta baillonii* plantation, hand-collecting.

###### Etymology.

This name is derived from the Latin word ‘undatus’, which means ‘wavy’, referring to the shape of the proximal edge of the paracymbium; adjective.

###### Diagnosis.

**Figure 93.**
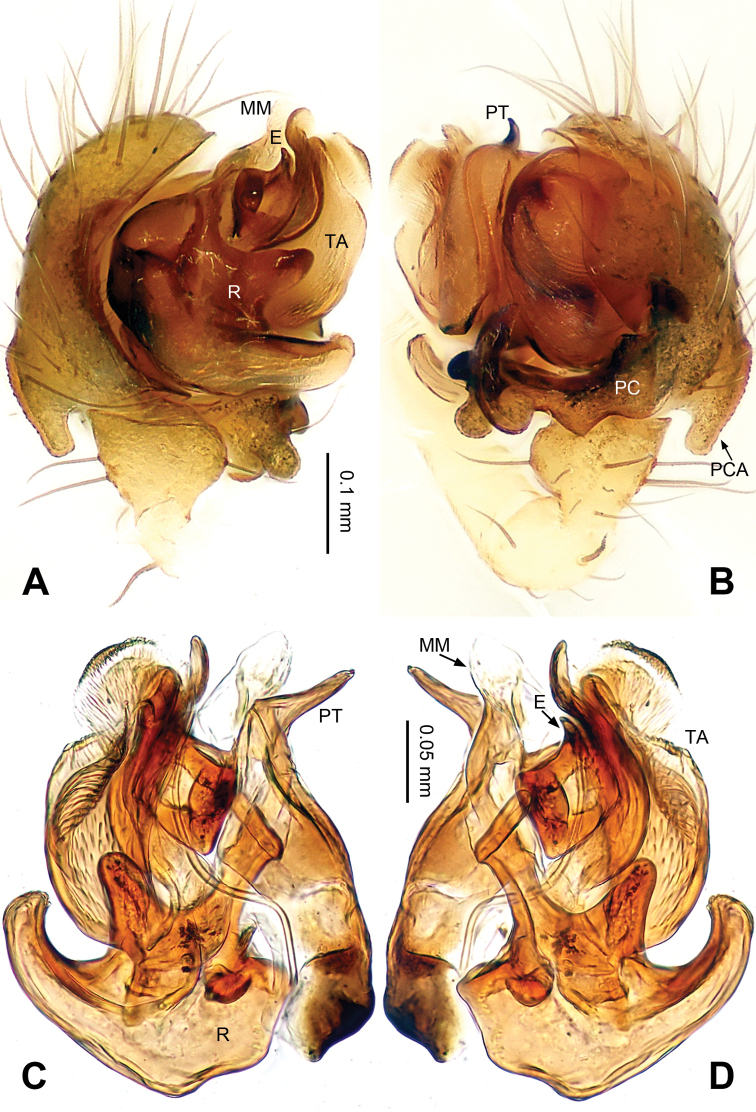
*Tapinopa undata* sp. n., male holotype. **A** Palp, prolateral view **B** Palp, retrolateral view **C** Embolic division, retrolateral view **D** Embolic division, prolateral view. Scale bars: **B** as **A**, **C** as **D.**

This species is related to *Tapinopa vara* Locket, 1982 (from Malaysia), but differs in the shape and size of terminal apophysis (Locket 1982: figs 100–102); the pit hook in *Tapinopa undata* sp. n. is longer and has a more pointed tip compared to the related species (Fig. 93C).

###### Description.

**Figure 95.**
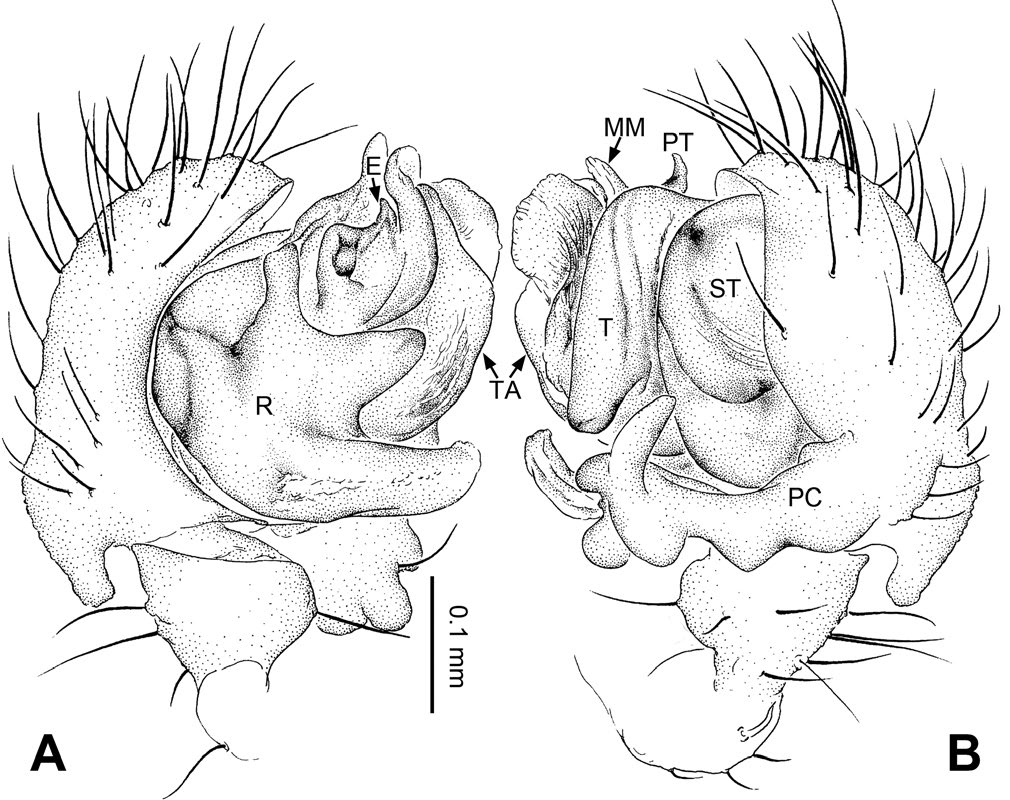
*Tapinopa undata* sp. n., male holotype. **A** Palp, prolateral view **B** Palp, retrolateral view. Scale bar: **B** as **A.**

Male (holotype). Total length: 1.16, carapace 0.75 long, 0.63 wide, orange-yellow, with dark lateral bands. Sternum 0.38 long, 0.38 wide. Clypeus 0.09 high. Chelicerae promargin with 2 teeth, retromargin with 3 teeth. Eye sizes and interdistances: AME 0.06, ALE 0.06, PME 0.06, PLE 0.06, AME-AME/AME 0.33, PME-PME/PME 1.00, AME-ALE/ALE 0.50, PME-PLE/PLE 0.67, Coxae IV separated by their width. Length of legs: I 3.00 (0.78, 0.21, 0.79, 0.73, 0.49), II 2.73 (0.70, 0.19, 0.70, 0.67, 0.47), III 1.92 (0.53, 0.17, 0.40, 0.47, 0.35), IV 2.46 (0.66, 0.16, 0.56, 0.65, 0.43). Leg formula: I-II-IV-III. TmI 0.20. Patellar spination formula: 2-1-1-1. Abdomen pale, with ill-defined dark spots around its circumference, and a large spot at the posterior end. Palp: tibia short, with one retrolateral trichobothrium; paracymbium complex, ‘J’-shaped, with wavelike posterior margin, apex trifurcate (Figs 93B, 95B); proximal cymbial extension turning basally, with a truncated tip (Fig. 93A–B); terminal apophysis of radix with three branches, all with curved silhouette; pit hook long, with an acute apex (Fig. 93C); radix with three projections, the posterior one stretching along paracymbium (Figs 93A, 95A); embolus short and pointed, with opening at tip (Fig. 93D).

Female. Unknown.

###### Distribution.

Known only from type localities.

##### Tapinopa vara

Taxon classificationAnimaliaAraneaeLinyphiidae

Locket, 1982

Tapinopa vara : Locket 1982: 380, figs 96–105 (♂♀).

###### Material examined.

2♀, CHINA, Yunnan: Menglun Town: Xishuangbanna Tropical Botanical Garden, 21°54.718'N, 101°16.940'E, elevation ca 645 m, 19.–25.09. 2006, secondary tropical seasonal moist forest, hand-collecting; 1♂, 21°54.813'N, 101°12.634'E, elevation ca 876 m, 5.–12.10.2006, secondary tropical montane evergreen broad-leaved forest, hand-collecting; 3♀, 21°54.772'N, 101°16.043'E, elevation ca 556 m, 19.–25.11.2006, *Paramechelia baillonii* plantation, hand-collecting; 3♀, 21°54.772'N, 101°16.043'E, elevation ca 556 m, 19.–25.01.2007, *Paramechelia baillonii* plantation, hand-collecting; 1♂, 21°54.813'N, 101°12.634'E, elevation ca 876 m, 5.–12.03.2007, secondary tropical montane evergreen broad-leaved forest, hand-collecting; 1♂, 21°54.463'N, 101°15.978'E, elevation ca 569 m, 19.–26.05.2007, rubber tree plantation, hand-collecting; 1♂, 21°54.684'N, 101°16.319'E, elevation ca 585 m, 10.–20.06.2007, rubber tree plantation, hand-collecting.

###### Distribution.

China, Malaysia.

####### Genus *Theoa* Saaristo, 1995

*Theoa*: Saaristo 1995: 45. Type species *Theonina tricaudata* Locket, 1992 from Malaysia.

##### Theoa bidentata sp. n.

Taxon classificationAnimaliaAraneaeLinyphiidae

http://zoobank.org/8D62CE41-D45D-4B83-A9A2-C948941F3DF7

**Figure 96.**
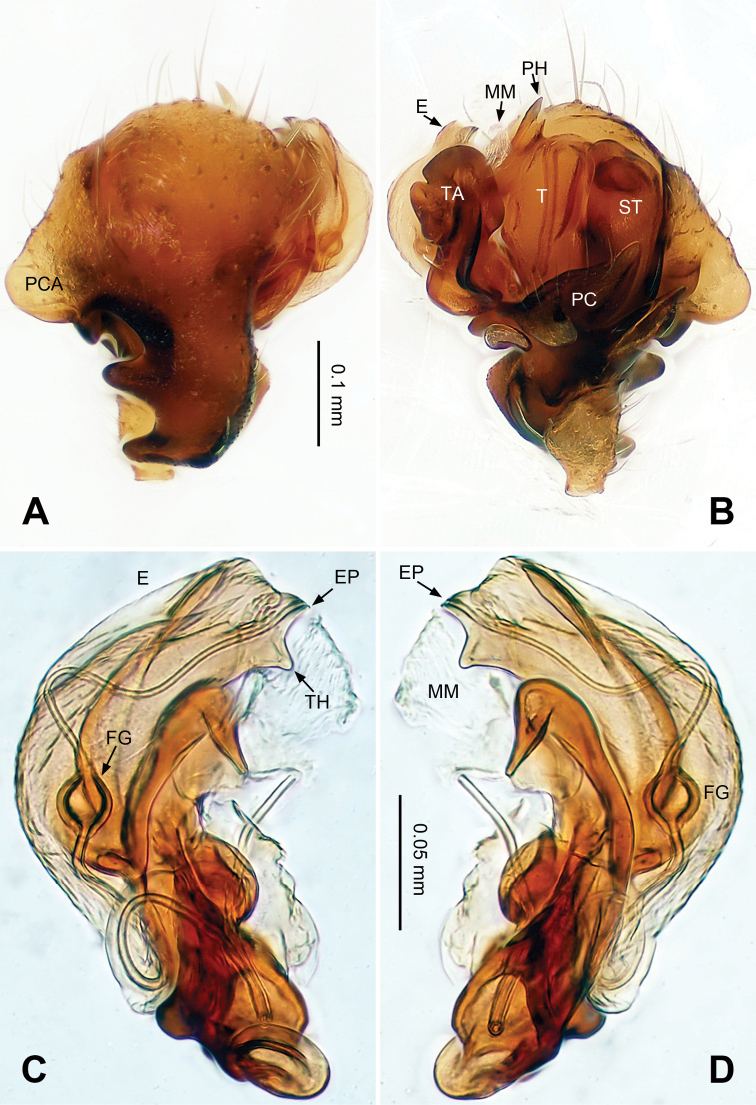
*Theoa bidentata* sp. n., male holotype. **A** Palp, prolateral view **B** Palp, retrolateral view **C** Embolus, retrolateral view **D** Embolus, prolateral view. Scale bars: **B** as **A**, **C** as **D.**

**Figure 97.**
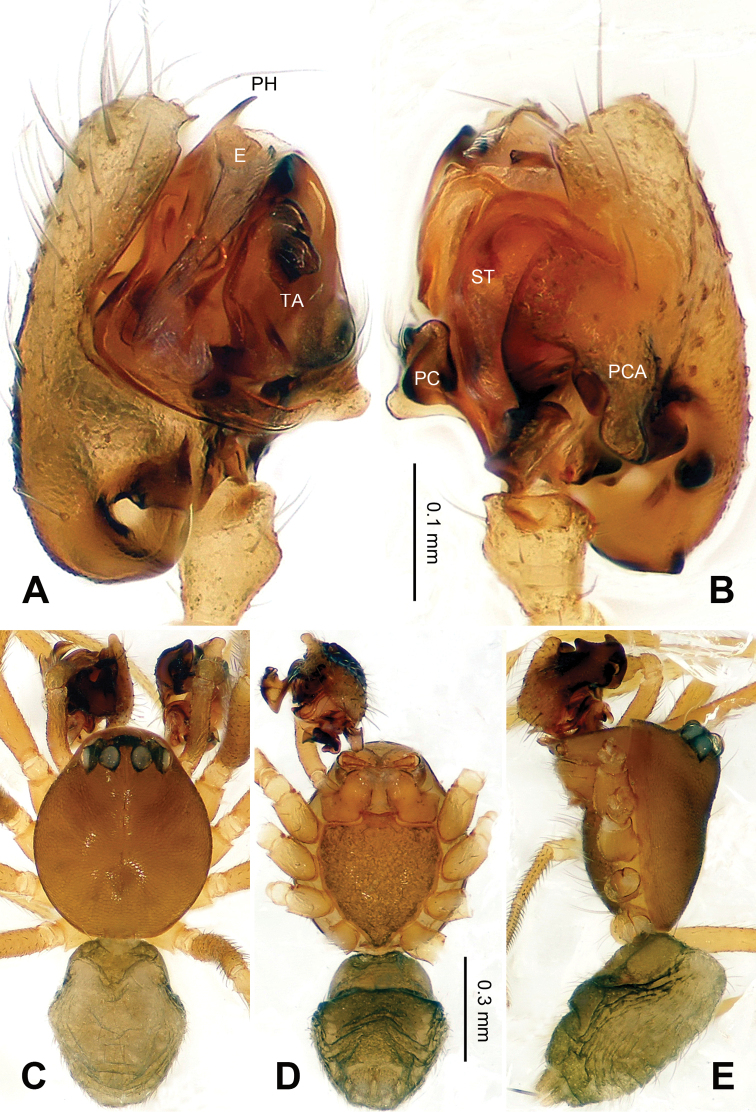
*Theoa bidentata* sp. n., male holotype. **A** Palp, ventral view **B** Palp, dorsal view **C** Habitus, dorsal view **D** Habitus, ventral view **E** Habitus, lateral view. Scale bars: **A** as **B**, **C** and **E** as **D.**

**Figure 98.**
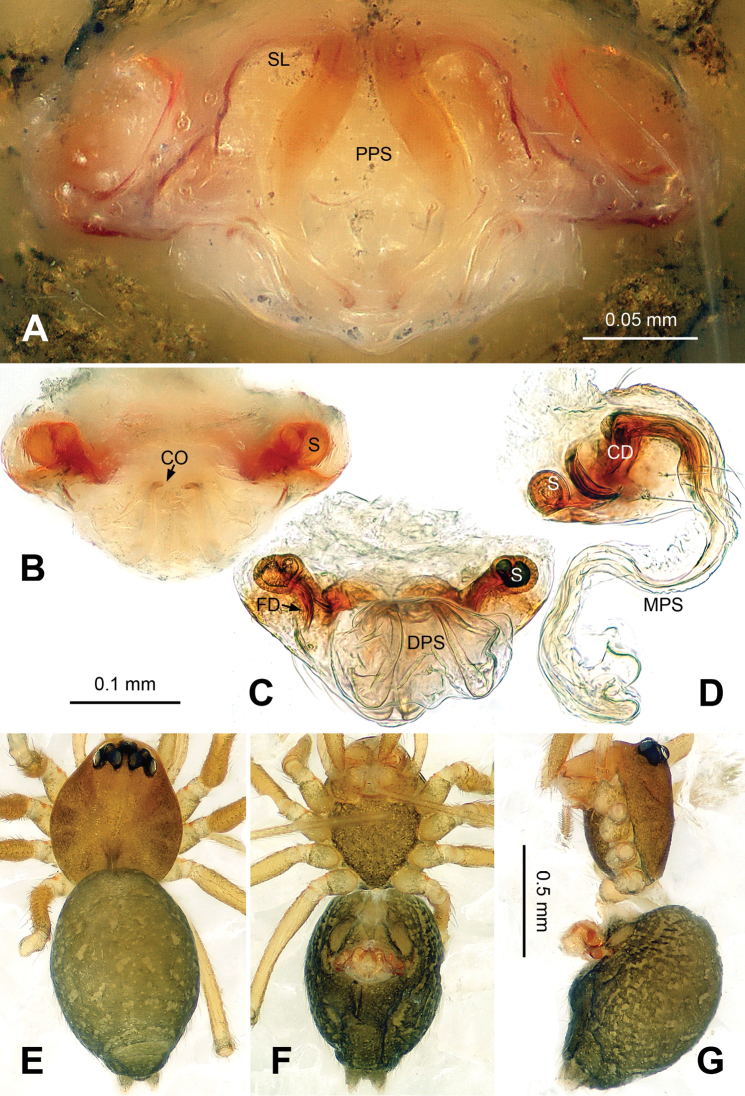
*Theoa bidentata* sp. n., female paratype. **A** Epigyne, ventral view **B** Epigyne, dorsal view **C** Vulva, dorsal view **D** Epigyne, lateral view **E** Habitus, dorsal view **F** Habitus ventral view **G** Habitus lateral view. Scale bars: **C** and **D** as **B**, **E** and **F** as **G.**

**Figure 99.**
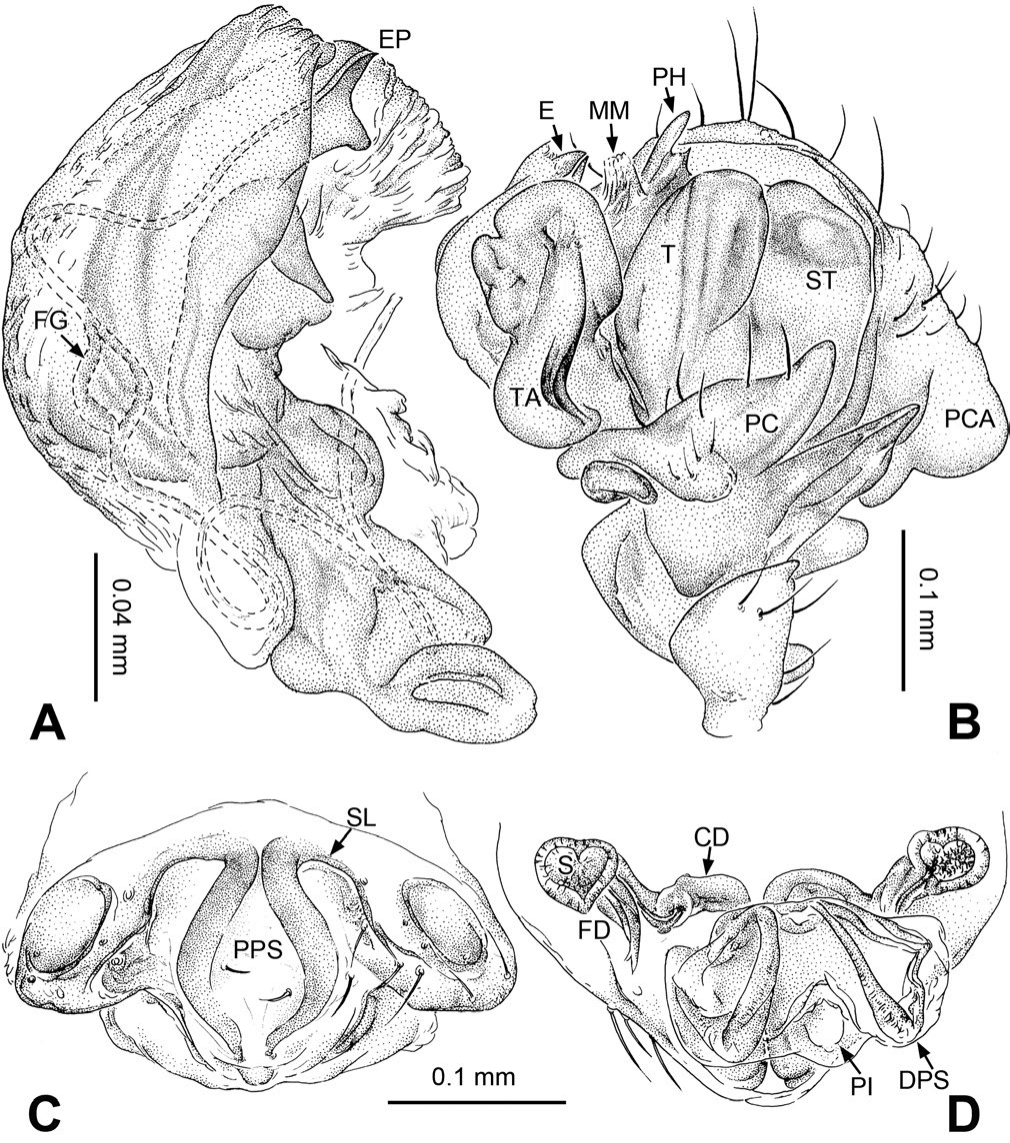
*Theoa bidentata* sp. n., male holotype (**A**–**B**) and female paratype (**C**–**D**). **A** Embolus, retrolateral view **B** Palp, retrolateral view **C** Epigyne, ventral view **D** Vulva, dorsal view. Scale bars: **C** as **D.**

Figs 96 , 97 , 98 , 99

###### Types.

Holotype ♂: CHINA, Yunnan: Menglun Town: Xishuangbanna Tropical Botanical Garden, 21°54.463'N, 101°15.978'E, elevation ca 569 m, 4.–11.05.2007, rubber tree plantation, hand-collecting. Paratypes 5♂, same data as holotype; 1♀, 21°55.551'N, 101°16.923'E, elevation ca 561 m, 1–9.09.2006, rubber-tea plantation, pitfall traps; 4♂, 21°54.463'N, 101°15.978'E, elevation ca 569 m, 1.01.2007, rubber tree plantation, hand-collecting; 4♂1♀, 21°54.684'N, 101°16.319'E, elevation ca 585 m, 16.–31.01.2007, rubber tree plantation, pitfall traps; 2♂, 21°54.684'N, 101°16.319'E, elevation ca 585 m, 4.–11.04.2007, rubber tree plantation, hand-collecting; 4♂, 21°54.684'N, 101°16.319'E, elevation ca 585 m, 1–15.05.2007, rubber tree plantation, pitfall traps; 3♂, 21°54.684'N, 101°16.319'E, elevation ca 585 m, 4.–11.V.2007, rubber tree plantation, hand-collecting; 3♂, 21°54.684'N, 101°16.319'E, elevation ca 585 m, 16–31.05.2007, rubber tree plantation, pitfall traps; 3♂, 21°54.772'N, 101°16.043'E, elevation ca 556 m, 19.–26.05.2007, *Paramichelia baillonii* plantation, hand-collecting; 1♀, 21°54.772'N, 101°16.034'E, elevation ca 556 m, 1.–15.06.2007, *Paramechelia baillonii* plantation, trunk traps; 1♂, 21°57.445'N, 101°12.997'E, elevation ca 774 m, 10.–20.06.2007, primary tropical seasonal rain forest, hand-collecting.

###### Etymology.

This name is combined by ‘bi’, and ‘dentatus’, meaning ‘with two teeth’, which refers to the two projections on the lateral margin of the prolateral cymbial outgrowth; adjective.

###### Diagnosis.

This species is distinguished as a member of *Theoa* by the extension at the dorso-mesal side of cymbium, and the sickle-shaped embolus with Fickert’s gland about half way along (Locket 1982: fig. 87). The large spermathecae on each side of the epigyne also resembles those in *Theoa tricaudata* (Locket 1982: fig. 89). It differs from the type species in the following aspects: the apex of cymbial outgrowth in *Theoa bidentata* sp. n. is bifid (Fig. 96A), not trifid as in *Theoa tricaudata* (Locket 1982: fig. 86); the pit hook in *Theoa bidentata* is not a discrete structure and is pointed, which differs from the bifid one in *Theoa tricaudata*; the terminal apophyses in the two species are of different shapes (Locket 1982: fig. 84; Fig. 96B); the spermathecae are more distantly spaced in *Theoa bidentata* (Fig. 98A). The two species also differ in spination: 1-0-0-0 or 1-1-1-0 in the generotype and 2-2-2-2 in the new taxon.

###### Description.

Male (holotype). Total length: 1.10. Carapace 0.60 long, 0.50 wide, unmodified, dark orange. Sternum 0.35 long, 0.34 wide. Clypeus 0.16 high. Chelicerae promargin with 5 teeth, retromargin with 4 teeth. Eye sizes and interdistances: AME 0.03, ALE 0.06, PME 0.07, PLE 0.06, AME-AME/AME 0.01, PME-PME/PME 0.40, AME-ALE/ALE 0.67, PME-PLE/PLE 0.43, coxae IV separated by 1.33 times their width. Length of legs: I 2.51 (0.66, 0.16, 0.70, 0.55, 0.44), II 2.32 (0.63, 0.16, 0.61, 0.53, 0.39), III 1.78 (0.48, 0.16, 0.42, 0.39, 0.33), IV 2.33 (0.64, 0.16, 0.63, 0.52, 0.38). Leg formula: I-IV-II-III. TmI 0.45. Tibial spine formula 2-2-2-2. Palp: tibia small, with one retrolateral trichobothrium; paracymbium ‘F’-shaped, two branches, both tapered at tip (Figs 96B, 99B); cymbium with a dorsal knob (Figs 96A, 99B); prolateral side of cymbium well-developed, almost fully covering the tegulum and suprategulum (Fig. 96A); cymbial outgrowth with two teeth-like processes at the lateral margin (Fig. 96A); pit hook bifid at tip (Fig. 96B); terminal apophysis in ill-defined shape, broad at ventral view (Fig. 96B); median membrane with a blunt apex, slightly bending forward; embolus sickle-shaped, inwardly curved, with a small, pointed embolus proper (Figs 96C, 99A); thumb of embolus small, located below embolus proper (Fig. 96C); Fickert’s gland present in radix (Figs 96C, 99A).

Female (one of paratypes). Total length: 1.24. Carapace 0.55 long, 0.43 wide, brownish orange. Sternum 0.30 long, 0.34 wide. Clypeus 0.11 high. Chelicerae promargin with 3 teeth, retromargin with 4 teeth. Eye sizes and interdistances: AME 0.03, ALE 0.06, PME 0.06, PLE 0.06, AME-AME/AME 0.33, PME-PME/PME 0.50, AME-ALE/ALE 0.33, PME-PLE/PLE 0.50, coxae IV separated by their width. Length of legs: I 2.37 (0.61, 017, 0.65, 0.50, 0.44), II 2.33 (0.59, 0.19, 0.58, 0.46, 0.41), III 1.76 (0.48, 0.15, 0.41, 0.38, 0.34), IV 2.24 (0.60, 0.16, 0.60, 0.48, 0.40). Leg formula: I-II-IV-III. TmI 0.24. Spine formula as in male. Abdomen greenish grey on dorsum, dark grey on venter. Epigyne: a wide and short ventral fig; scape long, ribbon-like, with copulatory openings at its broad end, (Fig. 98B–D); spermathecae small, separated by about four times their diameter (Fig. 98B).

###### Distribution.

Known only from type localities.

##### Theoa vesica sp. n.

Taxon classificationAnimaliaAraneaeLinyphiidae

http://zoobank.org/B79071B3-3859-412C-859D-137D368DBE0E

**Figure 100.**
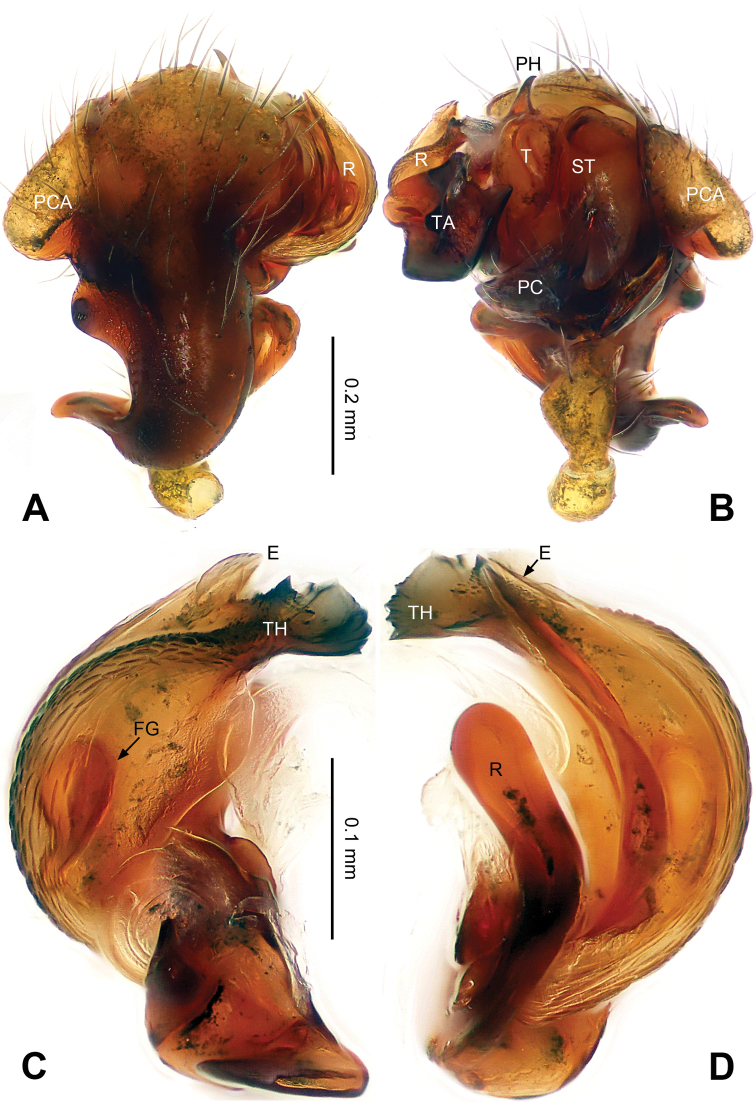
*Theoa vesica* sp. n., male holotype. **A** Palp, prolateral view **B** Palp, retrolateral view **C** Embolus, retrolateral view **D** Embolus, prolateral view. Scale bars: **B** as **A**, **D** as **C.**

**Figure 101.**
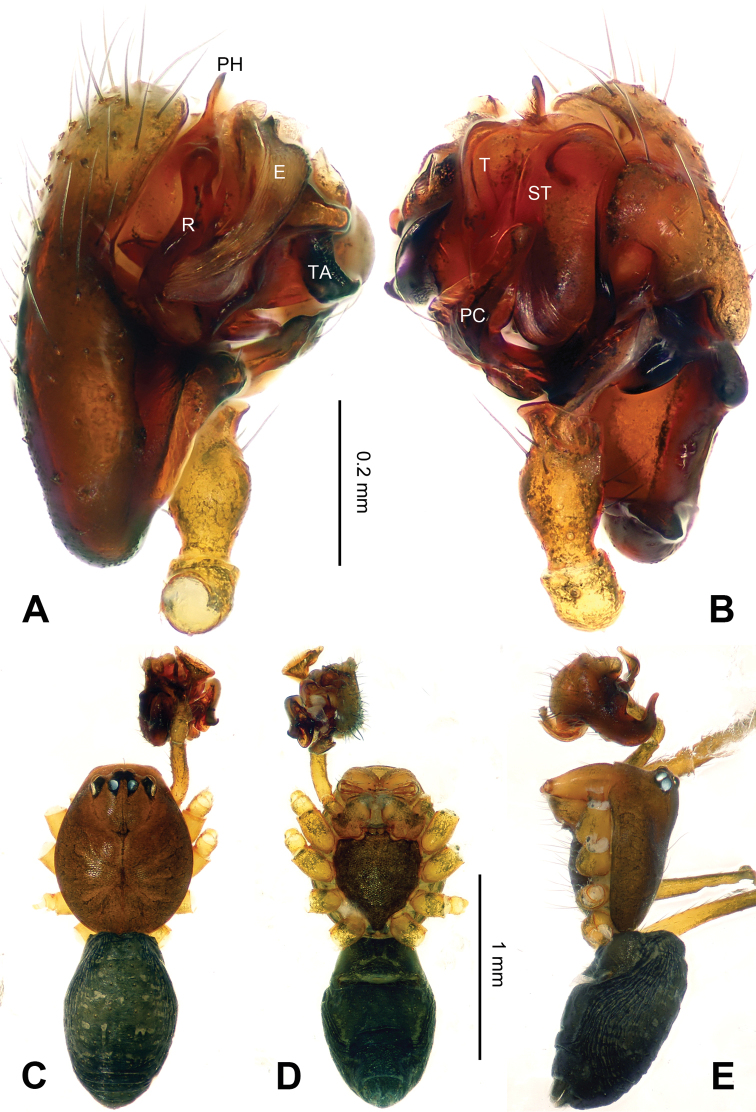
*Theoa vesica* sp. n., male holotype. **A** Palp, ventral view **B** Palp, dorsal view **C** Habitus, dorsal view **D** Habitus, ventral view **E** Habitus, lateral view. Scale bars: **B** as **A**, **C** and **D** as **E.**

**Figure 102.**
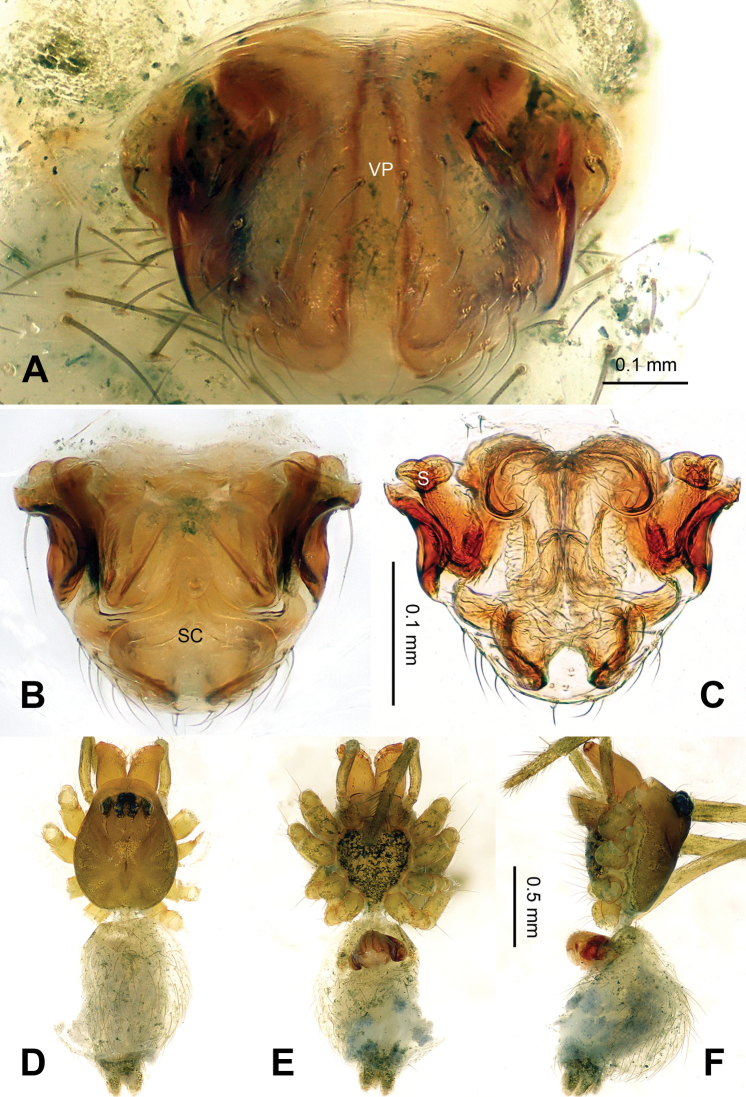
*Theoa vesica* sp. n., female paratype. **A** Epigyne, ventral view **B** Epigyne, dorsal view **C** Vulva, dorsal view **D** Habitus, dorsal view **E** Habitus, ventral view **F** Habitus lateral view. Scale bars: **B** as **C**, **D** and **E** as **F.**

**Figure 103.**
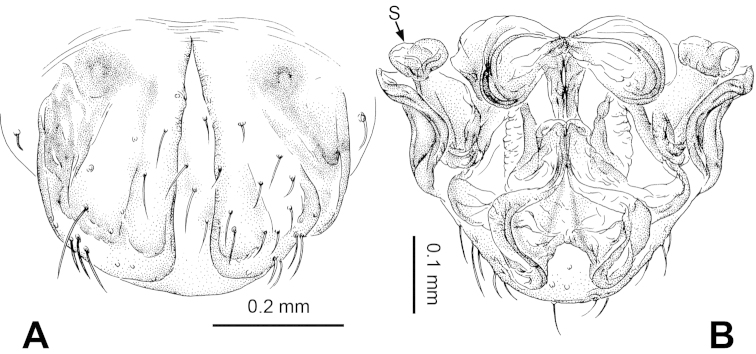
*Theoa vesica* sp. n., female paratype. **A** Epigyne, ventral view **B** Vulva, dorsal view.

Figs 100 , 101 , 102 , 103

###### Types.

Holotype: ♂: CHINA, Yunnan: Menglun Town: Xishuangbanna Nature Reserve, 21°57.010'N, 101°12.058'E, elevation ca 814 m, 18.08.2011, valley rain forest, fogging. Paratypes 1♀, Xishuangbanna Tropical Botanical Garden, 21°55.035'N, 101°16.500'E, elevation ca 558 m, 1.–15.07.2007, primary tropical seasonal rain forest, trunk traps; 1♀, 21°54.813'N, 101°12.634'E, elevation ca 876 m, 4.–11.04.2007, secondary tropical montane evergreen broad-leaved forest, trunk traps.

###### Etymology.

The name is derived from the Latin word ‘vesica’, which means ‘bladder, purse’, referring to the pouch-shaped structure in the vulva; noun in apposition.

###### Diagnosis.

The male is recognized by the huge cymbial outgrowth (Fig. 100B), and the cresent-shaped embolus with large Fickert’s gland about half way along (Fig. 100C). The female has unusually small spermathecae situated laterally (Fig. 102C). It also differs from other congeners by having TmIV.

###### Description.

Male (holotype). Total length: 1.78. Carapace 0.90 long, 0.73 wide, unmodified, brownish red. Sternum 0.45 long, 0.50 wide. Clypeus 0.22 high. Chelicerae promargin with 2 teeth, retromargin with 3 teeth. Eye sizes and interdistances: AME 0.06, ALE 0.06, PME 0.06, PLE 0.06, AME-AME/AME 0.01, PME-PME/PME 0.83, AME-ALE/ALE 0.76, PME-PLE/PLE 1.33, coxae IV separated by their width. Length of legs: I 3.90 (0.94, 0.25, 1.06, 0.98, 0.67), II 3.76 (1.00, 0.22, 1.00, 0.94, 0.60), III 2.98 (0.84, 0.23, 0.72, 0.72, 0.47), IV 3.49 (1.00, 0.24, 0.94, 0.93, 0.38). Leg formula: I-IV-II-III. TmI 0.26, TmIV 0.18. Tibial spine formula: 2-2-2-2. Abdomen dark green. Palp: tibia slightly elongated; paracymbium ‘C’-shaped, with a prominent distal end (Figs 100B, 101B); cymbium with a prominent process, extending proximally then turning dorsally (Fig. 100A); embolus sickle-shaped, with a small, pointed embolus proper (Fig. 100C); thumb of embolus with indented fringe (Fig. 100D); Fickert’s gland very large (Fig. 100C).

Female (one of paratypes). Total length: 1.88. Carapace 0.85 long, 0.68 wide, brownish yellow. Sternum 0.47 long, 0.48 wide. Clypeus 0.16 high. Chelicerae promargin with 3 teeth, retromargin with 5 teeth. Eye sizes and interdistances: AME 0.06, ALE 0.06, PME 0.06, PLE 0.07, AME-AME/AME 0.17, PME-PME/PME 0.83, AME-ALE/ALE 0.67, PME-PLE/PLE 0.86, coxae IV separated by their width. Length of legs: I 4.24 (1.09, 0.25, 1.16, 1.10, 0.64), II 3.80 (1.00, 0.25, 1.00, 0.94, 0.61), III 3.12 (0.80, 0.23, 0.81, 0.78, 0.50), IV 3.84 (1.09, 0.22, 0.98, 1.00, 0.55). Leg formula: I-IV-II-III. TmI 0.24, TmIV 0.14. Spine formula like in male. Abdomen pale. Epigyne: ventral fig bulgy (Figs 102A, 103A); scape short and broad (Fig. 102B); copulatory ducts long, following a complicated route (Fig. 102C); spermathecae small, held by a shield-like structure at each side of the epigyne (Figs 102C, 103B).

###### Distribution.

Known only from type localities.

##### Genus Vittatus gen. n.

Taxon classificationAnimaliaAraneaeLinyphiidae

http://zoobank.org/88D61CBA-E709-4B44-B0B0-301A32924495

###### Type species.

*Vittatus fencha* sp. n.

###### Etymology.

The generic name is an arbitrary combination of letters. Gender is masculine.

###### Diagnosis.

It is easily distinguished from other erigonine genera by its ribbon-like convector, its droplet-shaped cymbium and its prominent anterior radical apophysis (Figs 108A, 111B). The epigyne in this genus has a wide atrium and a scapoid.

###### Description.

Small sized Erigoninae. Carapace brown, modified in male (with small hump around PME and short sulci). Chelicerae with 5 promarginal and 4 retromarginal teeth in male, and with 5 promarginal and 5 retromarginal teeth in female. Chaetotaxy: tibial spine formula: 2-2-1-1. TmI ca 0.61, TmIV ca 0.61.

Male palp: tibia with trichobothria (prolateral and dorsal) and 2 apophyses: prolateral apophyses tongue-shaped, located near tibial base; dorsal apophysis, with a cup-like stem, and a petal-like extension around the upper-rim of the stem, bending downward (Figs 108B, 111B), Cymbium droplet-shaped (subtriangular) with elongate tip. Paracymbium ‘C’-shaped, broad at base (Fig. 108B). Tegulum small, with protegulum protruding ventrally (Fig. 108B). Anterior radical process bifurcate, with sharp tips (Fig. 111B); convector ribbon-like, with a tapering tip (Figs 108B, 111A); embolus long and sinuous, with a small thumb near embolus tip (Fig. 108C–D).

Epigyne: ventral fig mesally concave, with a scapoid (Fig. 110A); copulatory ducts simple (Fig. 110C); spermathecae dewdrop-shaped (Fig. 110C).

###### Species composition.

*Vittatus bian* sp. n, the type species *Vittatus fencha* sp. n, *Vittatus latus* sp. n, and *Vittatus pan* sp. n.

###### Distribution.

China.

##### Vittatus bian sp. n.

Taxon classificationAnimaliaAraneaeLinyphiidae

http://zoobank.org/FECD546B-BC6E-4232-8A30-5FC923805982

**Figure 104.**
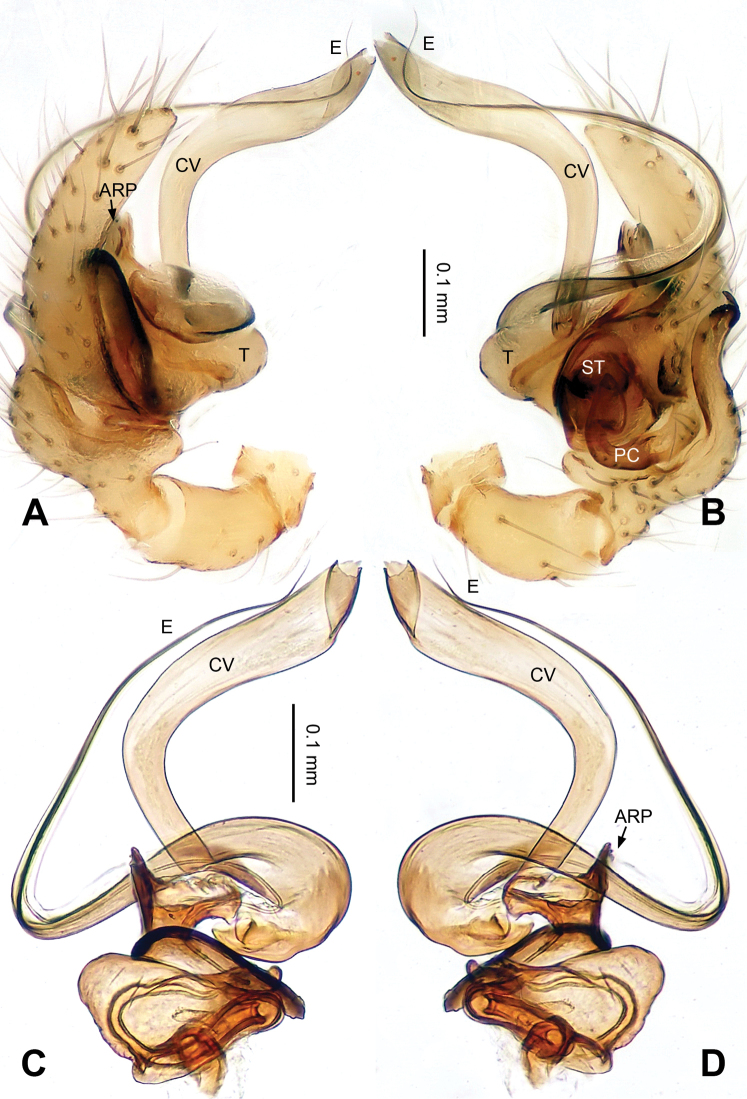
*Vittatus bian* sp. n., male holotype. **A** Right palp, prolateral view **B** Right palp, retrolateral view **C** Embolic division of the right palp, prolateral view **D** Embolic division of the right Palp, retrolateral view. Scale bars: **A** as **B**, **D** as **C.**

**Figure 105.**
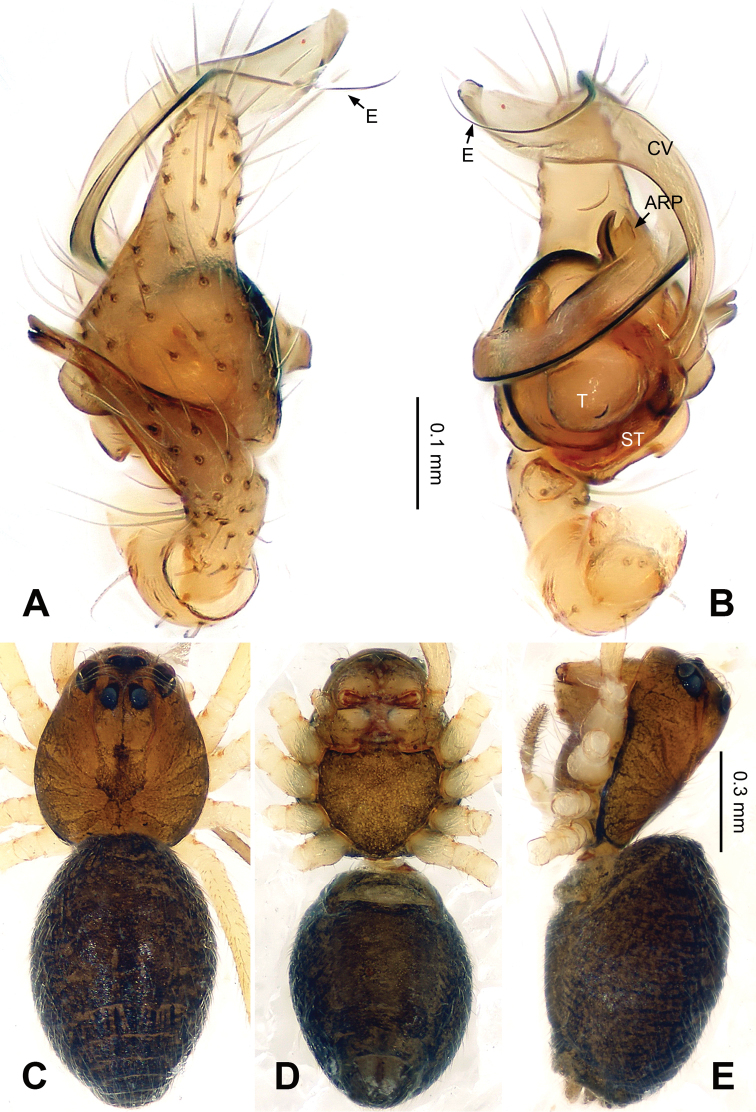
*Vittatus bian* sp. n., male holotype. **A** Right palp, dorsal view **B** Right palp, ventral view **C** Habitus, dorsal view **D** Habitus, ventral view **E** Habitus, lateral view. Scale bars: **A** as **B**, **C** and **D** as **E.**

**Figure 106.**
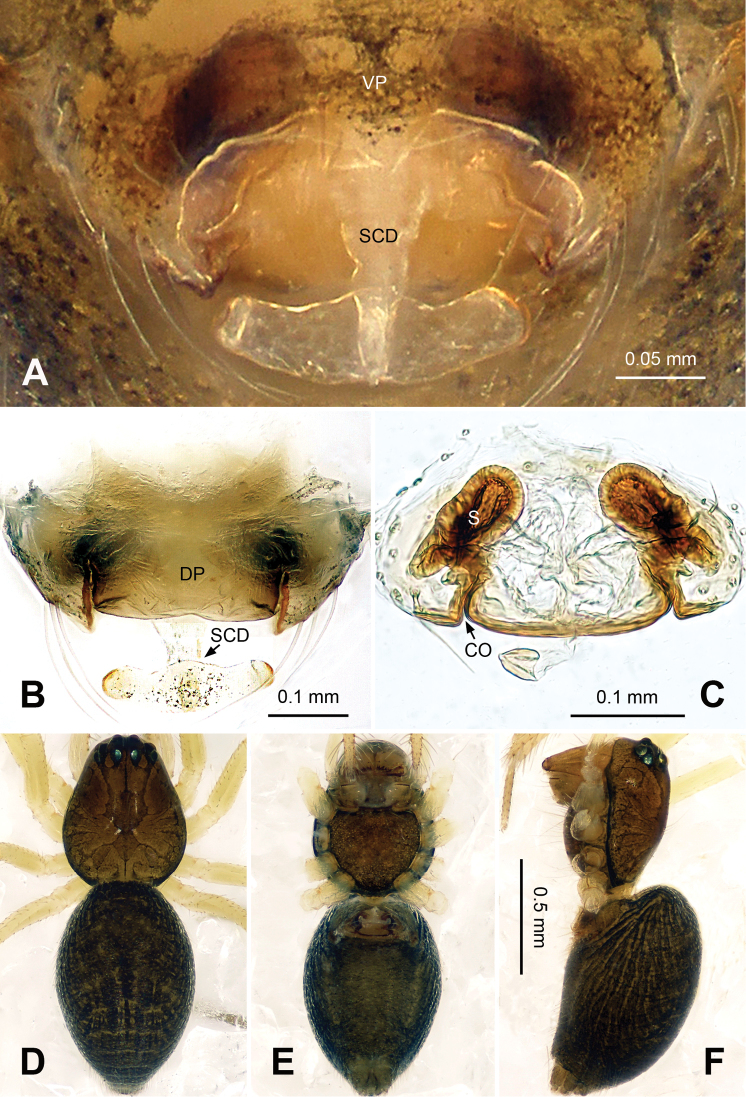
*Vittatus bian* sp. n., female paratype. **A** Epigyne, ventral view **B** Epigyne, dorsal view **C** Vulva, dorsal view **D** Habitus, dorsal view **E** Habitus, ventral view **F** Habitus lateral view. Scale bar: **D** and **E** as **F.**

**Figure 107.**
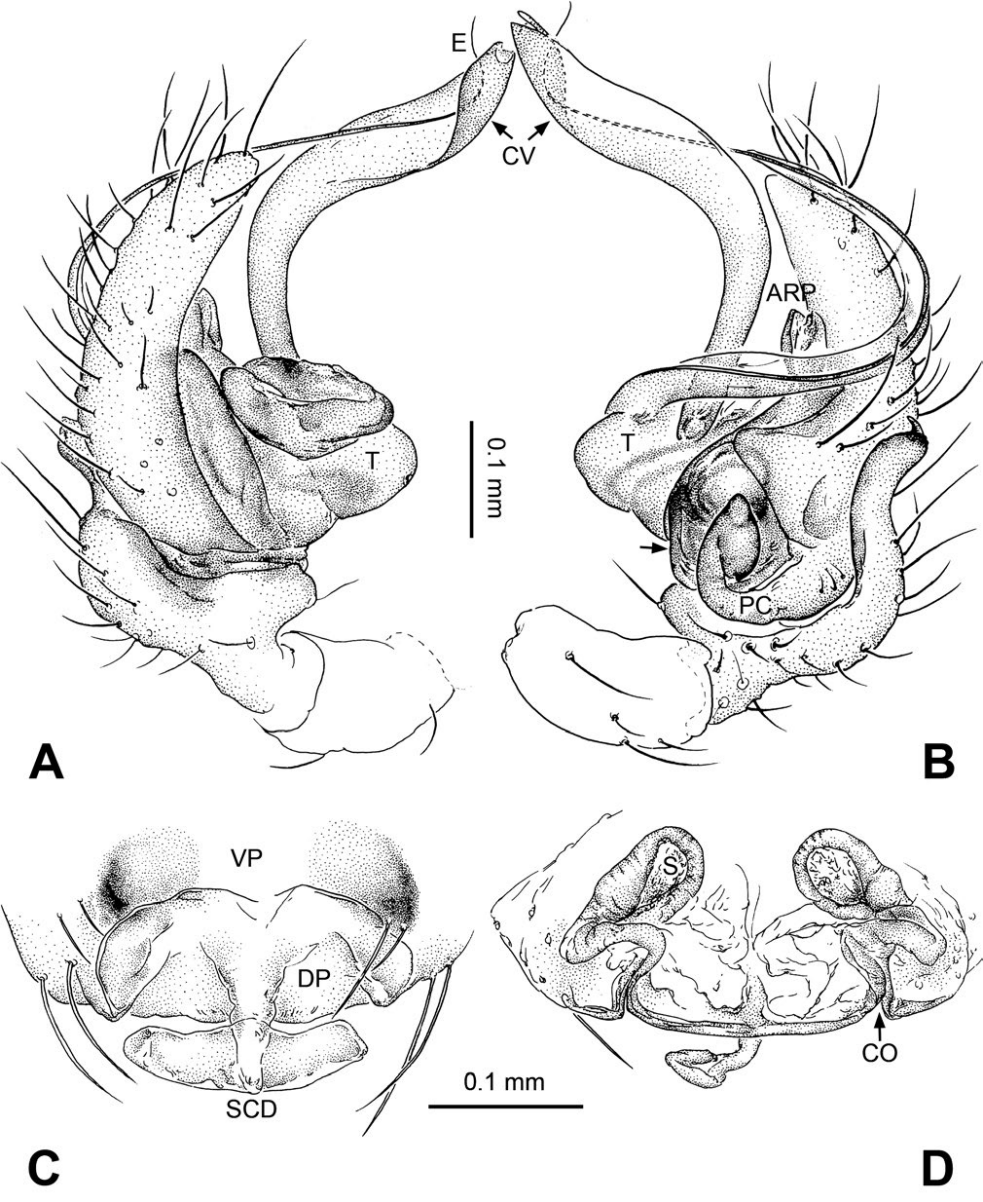
*Vittatus bian* sp. n., male holotype (**A**–**B**) and female paratype (**C**–**D**). **A** Right palp, prolateral view **B** Right palp, retrolateral view **C** Epigyne, ventral view **D** Vulva, dorsal view. Scale bars: **B** as **A**, **C** as **D.**

Figs 104 , 105 , 106 , 107

###### Types.

Holotype ♂: CHINA, Yunnan: Menglun Town: Xishuangbanna Tropical Botanical Garden, 21°57.809'N, 101°12.173'E, elevation ca 888 m, 4.08.2007, secondary tropical montane evergreen broad-leaved forest, fogging. Paratypes 3♀, 21°54.767'N, 101°11.431'E, elevation ca 880 m, 6.08.2007, secondary tropical montane evergreen broad-leaved forest, fogging; 1♀, 21°54.646'N, 101°16.257'E, elevation ca 572 m, 16.07.2007, rubber tree plantation, fogging.

###### Etymology.

This specific name is taken from the Chinese Pinyin ‘biān’, meaning ‘whip’, referring to the long, whip-like embolus; noun in apposition.

###### Diagnosis.

This species is similar to *Vittatus pan* sp. n. in the whip-like embolus and its turning direction (Fig. 104B), but differs by its dark coloration (light in *Vittatus pan* sp. n.) (Fig. 105C), lack of ventral tibial apophysis, straight dorsal tibial apophysis (Fig. 105A) and scape with terminal transversal arms (Fig. 106A).

###### Description.

Male (holotype). Total length: 1.40. Carapace 0.60 long, 0.50 wide, brown, with two rows of long setae between AME and PME. Sternum 0.36 long, 0.34 wide. Clypeus 0.11 high. Eye sizes and interdistances: AME 0.04, ALE 0.06, PME 0.05, PLE 0.05, AME-AME/AME 0.75, PME-PME/PME 0.80, AME-ALE/ALE 0.67, PME-PLE/PLE 1.60, coxae IV separated by 1.60 times their width. Length of legs: I 2.45 (0.64, 0.19, 0.69, 0.56, 0.37), II 2.34 (0.61, 0.16, 0.63, 0.57, 0.37), III 1.55 (0.47, 0.16, 0.31, 0.31, 0.30), IV 2.38 (0.63, 0.18, 0.61, 0.61, 0.35). Leg formula: I-IV-II-III. TmI 0.53, TmIV 0.52. Abdomen greenish brown. Palp: tibia with one dorsal apophysis subdivided on the top (Fig. 105A). Cymbium droplet-shaped (Fig. 105A); paracymbium ‘C’-shaped, with a leaf-like apex (Fig. 104B). Bulb with a flattened subtegulum and a slightly protruding tegulum (Fig. 104A). Embolus long, whip-like; convector long, ribbon-like (Fig. 104B). Embolus and convector of equal length. Radical apophysis straight, bifurcate (Fig. 104B).

Female (one of paratypes). Total length: 1.53. Carapace 0.51 long, 0.52 wide, dark brown, with black pattern. Sternum 0.38 long, 0.36 wide. Clypeus 0.09 high. Chelicerae promargin with 4 teeth, retromargin with 4 teeth. Eye sizes and interdistances: AME 0.04, ALE 0.06, PME 0.04, PLE 0.04, AME-AME/AME 1.00, PME-PME/PME 0.75, AME-ALE/ALE 0.67, PME-PLE/PLE 1.25, coxae IV separated by 1.33 times their width. Length of legs: I 2.27 (0.59, 0.17, 0.61, 0.54, 0.36), II 2.24 (0.61, 0.20, 0.58, 0.50, 0.35), III 1.82 (0.50, 0.15, 0.44, 0.44, 0.29), IV 2.25 (0.62, 0.18, 0.58, 0.55, 0.32). Leg formula: I-IV-II-III. TmI 0.56, TmIV 0.54. Abdomen dark brown. Epigyne: a ventral fig semi-rounded, mesally concave, with a membraneous scapoid, equipped by membraneous arm at each side of the tip (Fig. 106A); dorsal fig wide, with blunt posterior rim; spermathecae somewhat oval (Fig. 106C).

###### Distribution.

Known only from type localities.

##### Vittatus fencha sp. n.

Taxon classificationAnimaliaAraneaeLinyphiidae

http://zoobank.org/5C6D0C4F-5B1E-460A-BD7D-6754010DC295

**Figure 108.**
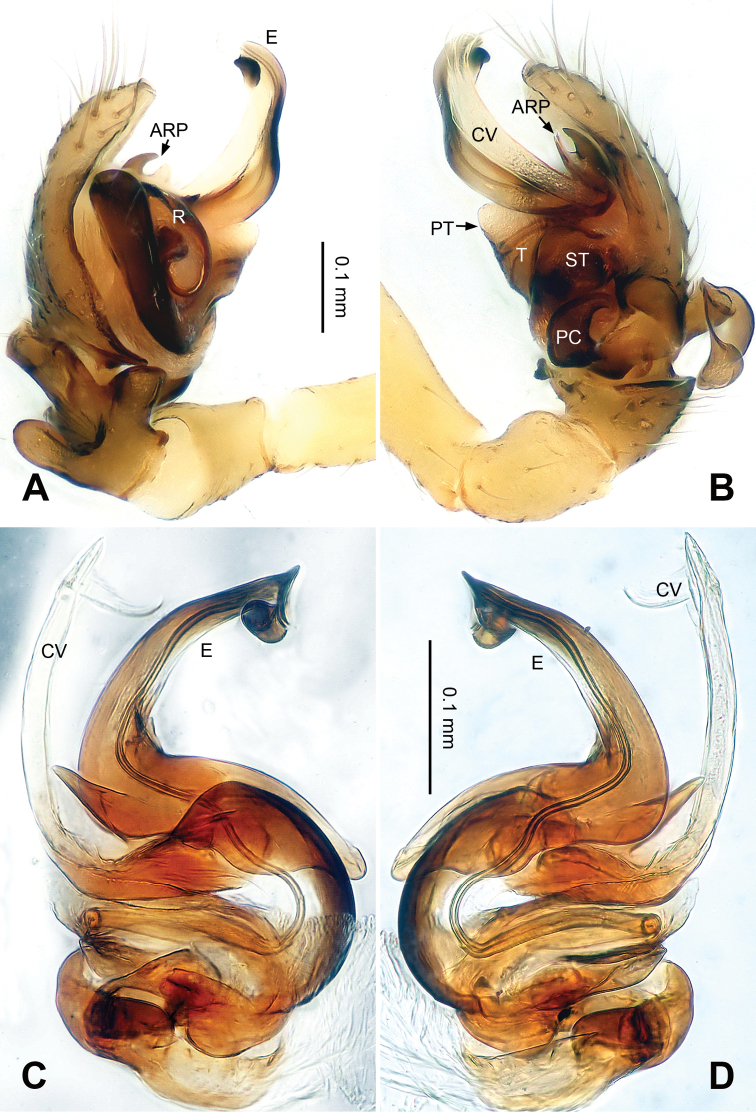
*Vittatus fencha* sp. n., male holotype. **A** Palp, prolateral view **B** Palp, retrolateral view **C** Embolic division, retrolateral view **D** Embolic division, prolateral view. Scale bars: **B** as **A**, **C** as **D.**

**Figure 109.**
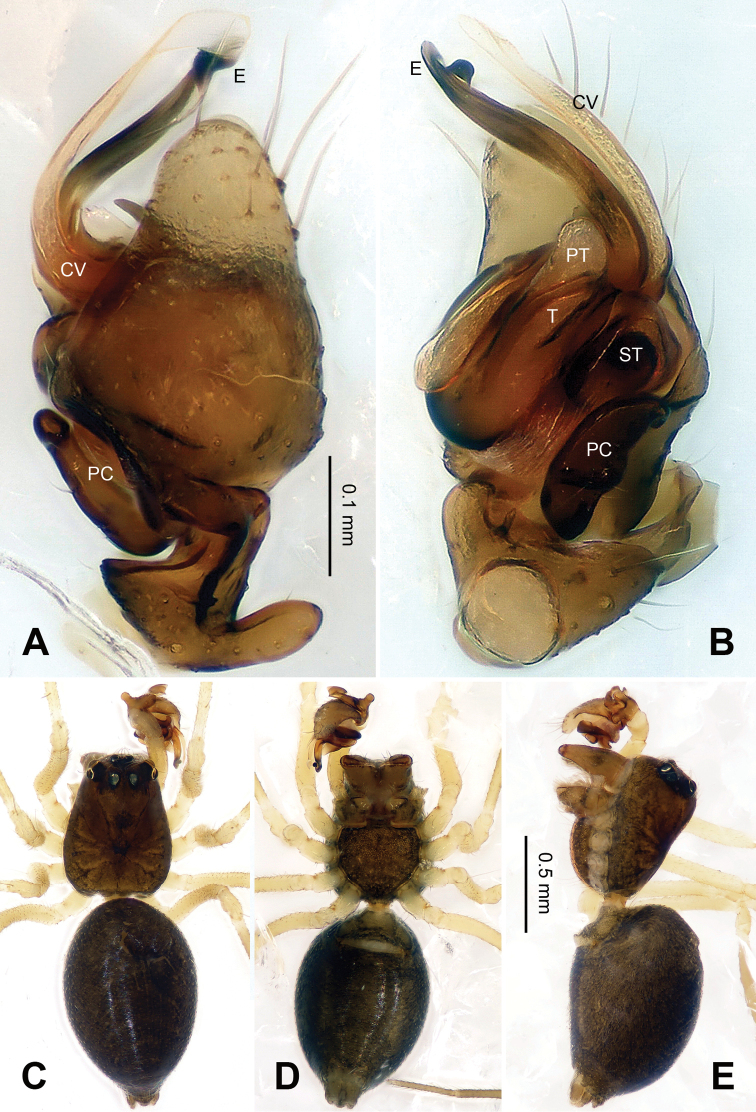
*Vittatus fencha* sp. n., male holotype. **A** Palp, dorsal view **B** Palp, ventral view **C** Habitus, dorsal view **D** Habitus, ventral view **E** Habitus, lateral view. Scale bars: **B** as **A**, **C** and **D** as **E.**

**Figure 110.**
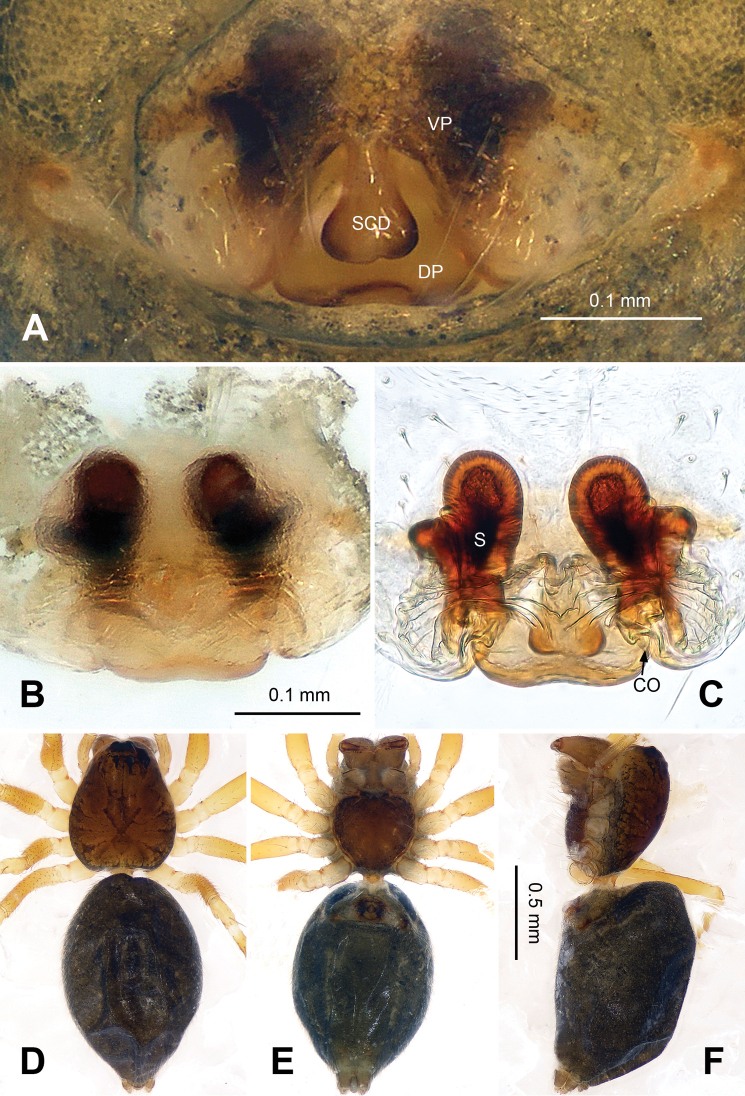
*Vittatus fencha* sp. n., female paratype. **A** Epigyne, ventral view **B** Epigyne, dorsal view **C** Vulva, dorsal view **D** Habitus, dorsal view **E** Habitus, ventral view **F** Habitus lateral view. Scale bars: **C** as **B**, **D** and **E** as **F.**

**Figure 111.**
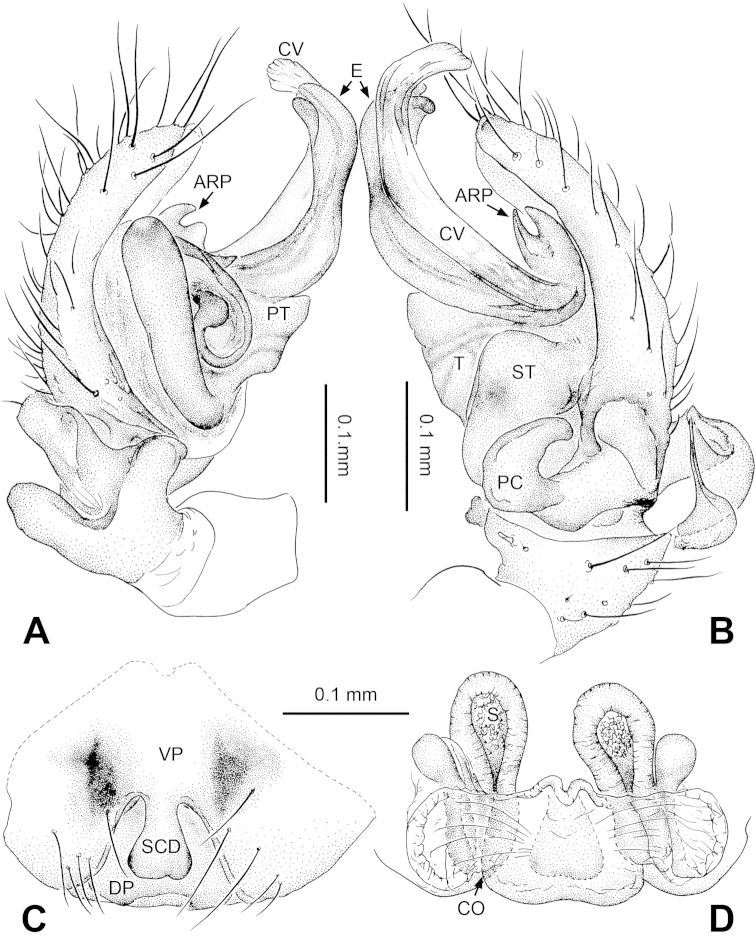
*Vittatus fencha* sp. n., male holotype (**A**–**B**) and female paratype (**C**–**D**). **A** Palp, prolateral view **B** Palp, retrolateral view **C** Epigyne, ventral view **D** Vulva, dorsal view. Scale bar: **D** as **C.**

Figs 108 , 109 , 110 , 111

###### Types.

Holotype ♂: CHINA, Yunnan: Menglun Town: Xishuangbanna Nature Reserve, 21°57.445'N, 101°12.997'E, elevation ca 744 m, 30.07.2007, primary tropical seasonal rain forest, fogging. Paratypes 9♂9♀, same data as holotype; 3♂4♀, 21°57.669'N, 101°11.893'E, elevation ca 790 m, 7.08.2007, primary tropical seasonal rain forest, fogging; 14♂43♀, 21°54.725'N, 101°13.261'E, elevation ca 734 m, 8.08.2007, primary tropical seasonal rain forest, fogging.

###### Etymology.

The name for this species is derived from the Chinese Pinyin ‘fēn chà’, which means ‘forked’, in reference to the fork-shaped anterior radical apophysis in male; term in apposition.

###### Diagnosis.

It differs from other congeners by the stem-cup-shaped dorsal tibial apophysis (Fig. 108B) and the ribbon-like embolus with a small outgrowth near tip in palp (Fig. 109B); female resembles *Vittatus bian* sp. n. in the closely positioned spermathecae (Figs 106C, 110C), but differs in the short and unmodified scapoid (Fig. 110A).

###### Description.

Male (holotype). Total length: 1.80. Carapace 0.60 long, 0.53 wide, ocular area mildly lifted with short setae, brown. Sternum 0.39 long, 0.39 wide. Clypeus 0.13 high. Eye sizes and interdistances: AME 0.05, ALE 0.07, PME 0.06, PLE 0.06, AME-AME/AME 0.50, PME-PME/PME 1.67, AME-ALE/ALE 0.71, PME-PLE/PLE 0.67, coxae IV separated by 1.50 times their width. Length of legs: I 2.87 (0.75, 0.22, 0.78, 0.70, 0.42), II 2.80 (0.76, 0.20, 0.70, 0.66, 0.48), III 2.17 (0.63, 0.18, 0.50, 0.50, 0.36), IV 2.58 (0.63, 0.16, 0.70, 0.70, 0.39). Leg formula: I-II-IV-III. TmI 0.61, TmIV 0.61. Abdomen brown. Palp: see description of the genus.

Female (one of paratypes). Total length: 1.85. Carapace 0.65 long, 0.56 wide, unmodified, same as male in coloration. Sternum 0.41 long, 0.44 wide. Clypeus 0.13 high. Eye sizes and interdistances: AME 0.04, ALE 0.05, PME 0.04, PLE 0.05, AME-AME/AME 0.8, PME-PME/PME 1.5, AME-ALE/ALE 0.9, PME-PLE/PLE 1.1, coxae IV separated by 1.58 times their width. Length of legs: I 2.59 (0.75, 0.19, 0.64, 0.63, 0.38), II 2.49 (0.69, 0.20, 0.60, 0.60, 0.40), III 2.09 (0.63, 0.16, 0.45, 0.50, 0.35), IV 2.50 (0.70, 0.20, 0.60, 0.60, 0.40). Leg formula: I-IV-II-III. TmI 0.60, TmIV 0.63. Abdomen brown. Epigyne: see description of the genus.

###### Distribution.

Known only from type localities.

##### Vittatus latus sp. n.

Taxon classificationAnimaliaAraneaeLinyphiidae

http://zoobank.org/4B60B5E6-1F30-43E0-BEBA-13D3F61C97C2

**Figure 112.**
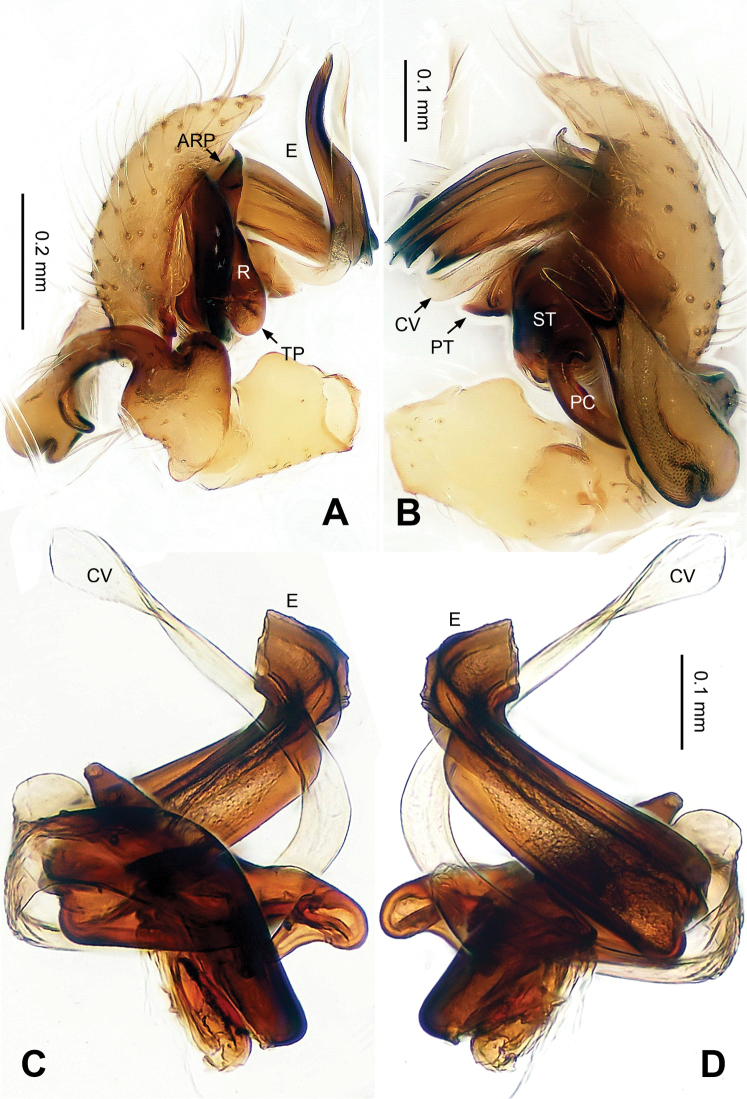
*Vittatus latus* sp. n., male holotype. **A** Right palp, prolateral view **B** Right palp, retrolateral view **C** Embolic division of the right palp, prolateral view **D** Embolic division of the right palp, retrolateral view. Scale bar: **D** as **C.**

**Figure 113.**
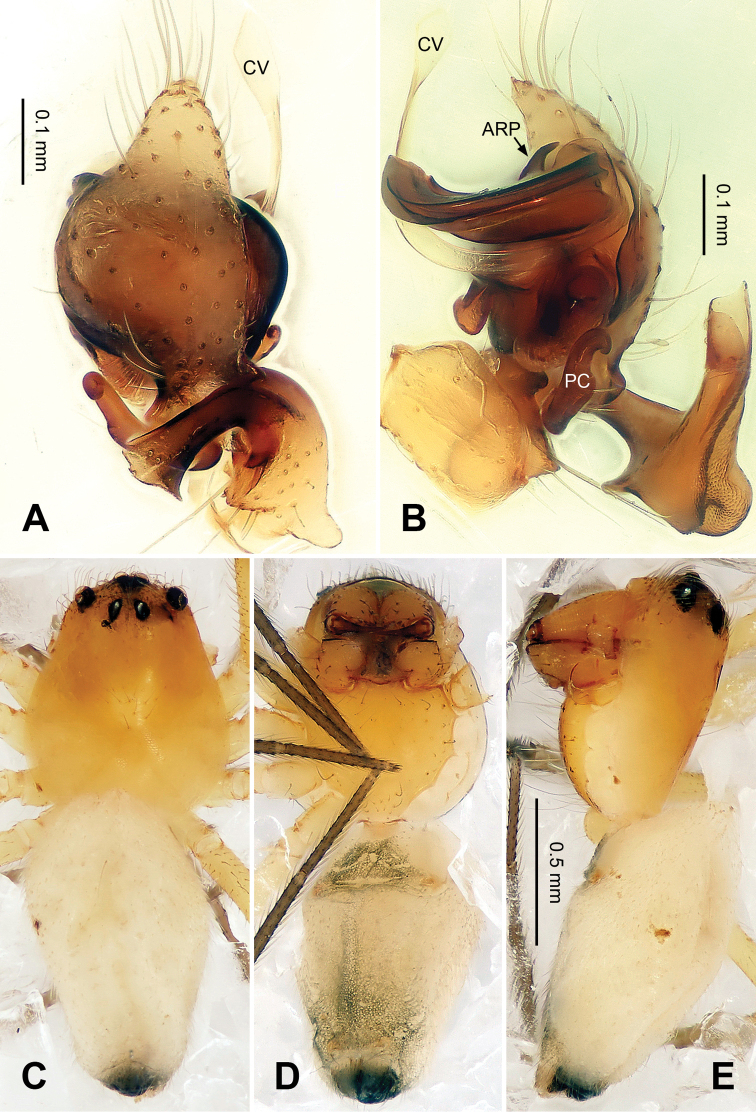
*Vittatus latus* sp. n., male holotype. **A** Right palp, dorsal view **B** Right palp, ventral view **C** Habitus, dorsal view **D** Habitus, ventral view **E** Habitus, lateral view. Scale bar: **C** and **D** as **E.**

**Figure 114.**
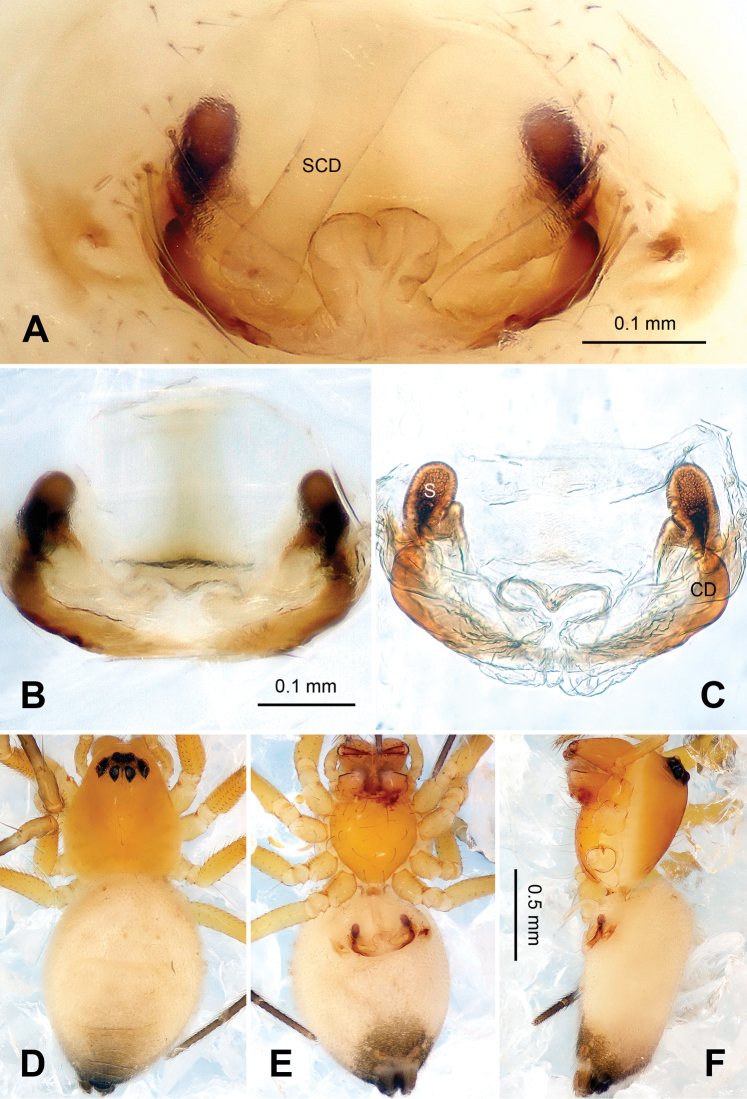
*Vittatus latus* sp. n., female paratype. **A** Epigyne, ventral view **B** Epigyne, dorsal view **C** Vulva, dorsal view **D** Habitus, dorsal view **E** Habitus, ventral view **F** Habitus lateral view. Scale bars: **C** as **B**, **D** and **E** as **F.**

**Figure 115.**
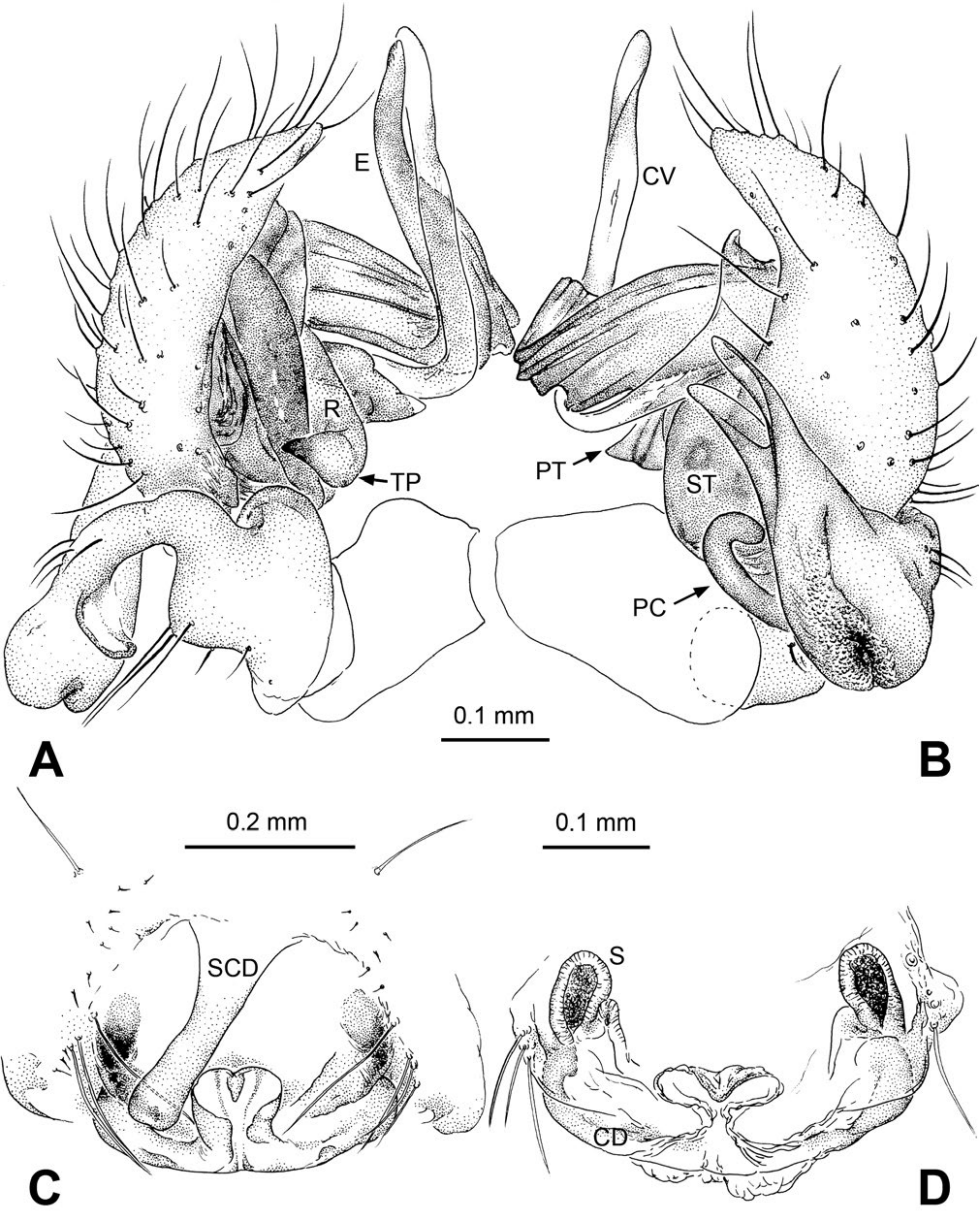
*Vittatus latus* sp. n., male holotype (**A**–**B**) and female paratype (**C**–**D**). **A** Right palp, prolateral view **B** Right palp, retrolateral view **C** Epigyne, ventral view **D** Vulva, dorsal view. Scale bar: **A** as **B.**

Figs 112 , 113 , 114 , 115

###### Types.

Holotype ♂: CHINA, Yunnan: Menglun Town: Xishuangbanna Nature Reserve, 21°57.809'N, 101°12.173'E, elevation ca 888 m, 4.08.2007, secondary tropical montane evergreen broad-leaved forest, fogging. Paratypes 12♀, same data as holotype; 3♀, 21°57.784'N, 101°11.947'E, elevation ca 895 m, 6.08.2007, secondary tropical montane evergreen broad-leaved forest, fogging; 3♀, 21°54.767'N, 101°11.431'E, elevation ca 880 m, 6.08.2007, secondary tropical montane evergreen broad-leaved forest, fogging.

###### Etymology.

This specific name is taken from the Latin word ‘latus’, meaning ‘broad’, in reference to the wide base of the embolus; adjective.

###### Diagnosis.

This species greatly resembles *Vittatus fencha* sp. n., but differs in the following aspects: the anterior radical process in *Vittatus latus* sp. n. is larger and has a hooked tip (Fig. 112A); the embolus is wider at the base, without thumb near tip (Fig. 112B); the scapoid of *Vittatus latus* sp. n. is much longer and the spermathecae are distantly separated (Fig. 114A, C). Palpal patella is modified with a hump in *Vittatus latus* sp. n.

###### Description.

Male (holotype). Total length: 1.75. Carapace 0.75 long, 0.68 wide, unmodified, orange. Abdomen pale, with greenish grey spinnerets. Sternum 0.38 long, 0.44 wide. Clypeus 0.18 high. Chelicerae promargin with 5 teeth, retromargin with 3 teeth. Eye sizes and interdistances: AME 0.04, ALE 0.05, PME 0.03, PLE 0.04, AME-AME/AME 0.75, PME-PME/PME 1.67, AME-ALE/ALE 1.80, PME-PLE/PLE 2.50, coxae IV separated by 1.73 of their diameters. Length of legs: I 3.03 (0.80, 0.23, 0.81, 0.75, 0.44), II 2.90 (0.80, 0.22, 0.78, 0.70, 0.40), III 2.38 (0.64, 0.20, 0.56, 0.60, 0.38), IV 2.89 (0.80, 0.20, 0.75, 0.75, 0.39). Leg formula: I-II-IV-III. TmI 0.67, TmIV 0.63. Palp: tibial prolateral apophysis modified, a tube-like process stretching from the distal margin of tibia, slightly curved (Fig. 112B), developed into a broader, hollow structure, with sulci along the bottom (Fig. 112A); a belt of conspicuous papillae stretching from the inner surface of the sulci to the bifid apex (Fig. 112A); cymbium lunar-shaped (Figs 112B, 115B); paracymbium ‘J’-shaped, narrow (Fig. 112A); distal suprategular apophysis triangular, slightly hooked at tip (Fig. 112B); embolus long and wide, accompanied by convector, following the same route (Figs 112A–B, 115A–B).

Female (one of paratypes). Total length: 1.88. Carapace 0.80 long, 0.63 wide, unmodified, orange. Abdomen pale, with greenish-grey spinnerets and dark patches around them. Sternum 0.47 long, 0.47 wide. Clypeus 0.15 high. Chelicerae promargin and retromargin with 5 teeth. Eye sizes and interdistances: AME 0.04, ALE 0.06, PME 0.06, PLE 0.05, AME-AME/AME 0.75, PME-PME/PME 0.60, AME-ALE/ALE 0.50, PME-PLE/PLE 0.67, Coxae IV separated by 1.54 times their width. Lengths of legs: I 2.94 (0.75, 0.25, 0.81, 0.69, 0.44), II 2.82 (0.75, 0.23, 0.70, 0.70, 0.44), III 2.36 (0.63, 0.23, 0.55, 0.56, 0.39), IV 2.96 (0.80, 0.20, 0.74, 0.74, 0.48). Leg formula: IV-I -II-III. TmI 0.69, TmIV 0.68. Patellar spine formula: 2-2-2-2. Epigyne: scapoid ribbon-like, with a slightly concave tip (Fig. 114A); dorsal fig with an opening at the posterior rim (Fig. 114A–C); copulatory ducts follow a path from the middle to each side, then up to the spermathecae (Fig. 114D); spermathecae elliptical (Fig. 114A).

###### Distribution.

Known only from type localities.

##### Vittatus pan sp. n.

Taxon classificationAnimaliaAraneaeLinyphiidae

http://zoobank.org/74E52D87-E9E5-466C-A54B-18F9127707A8

**Figure 116.**
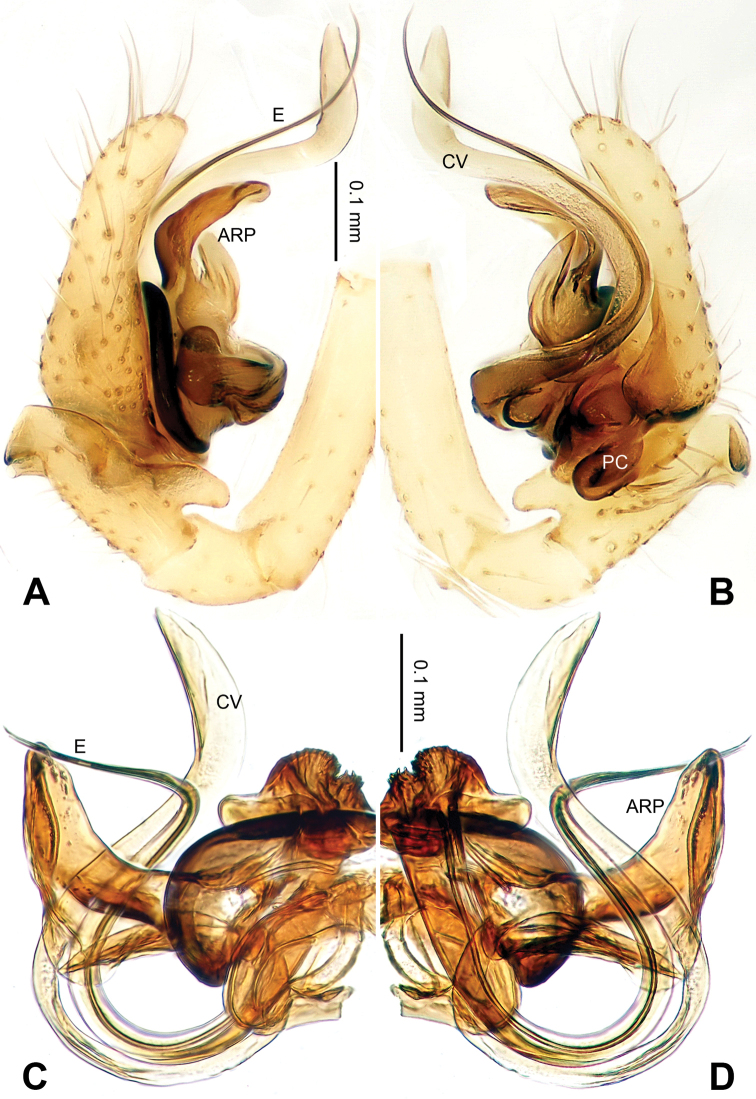
*Vittatus pan* sp. n., male holotype. **A** Palp, prolateral view **B** Palp, retrolateral view **C** Embolic division, retrolateral view **D** Embolic division, prolateral view. Scale bars: **B** as **A**, **C** as **D.**

**Figure 117.**
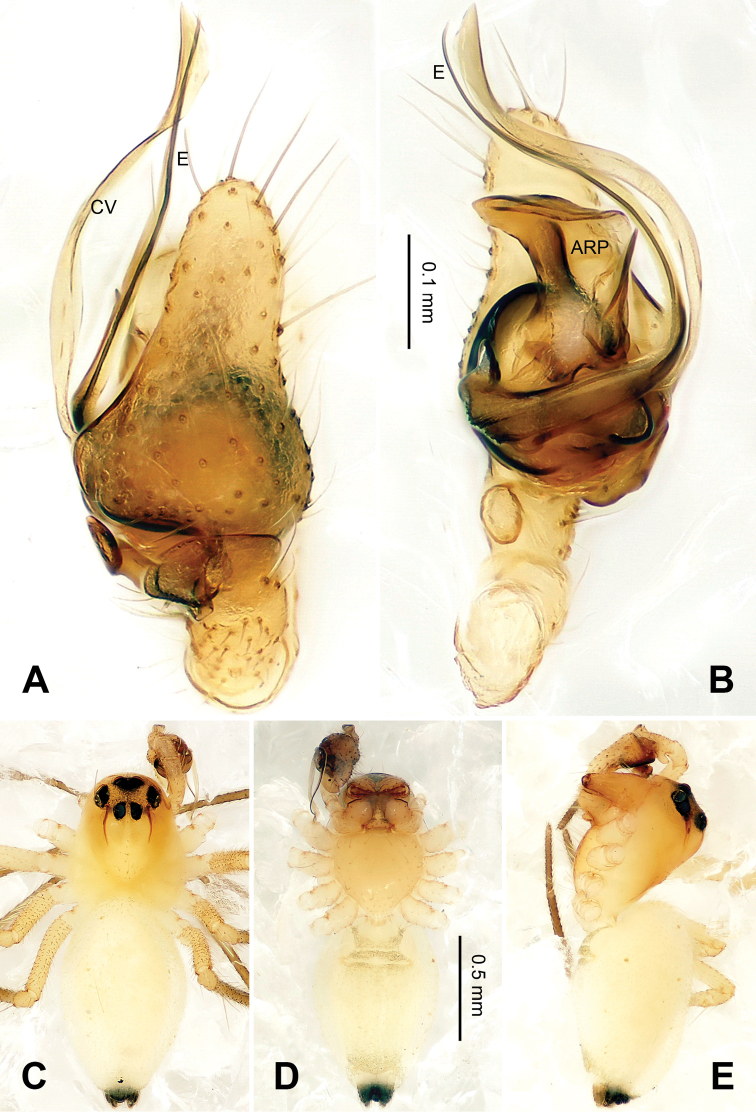
*Vittatus pan* sp. n., male holotype. **A** Palp, dorsal view **B** Palp, ventral view **C** Habitus, dorsal view **D** Habitus, ventral view **E** Habitus, lateral view. Scale bars: **A** as **B**, **C** and **E** as **D.**

**Figure 118.**
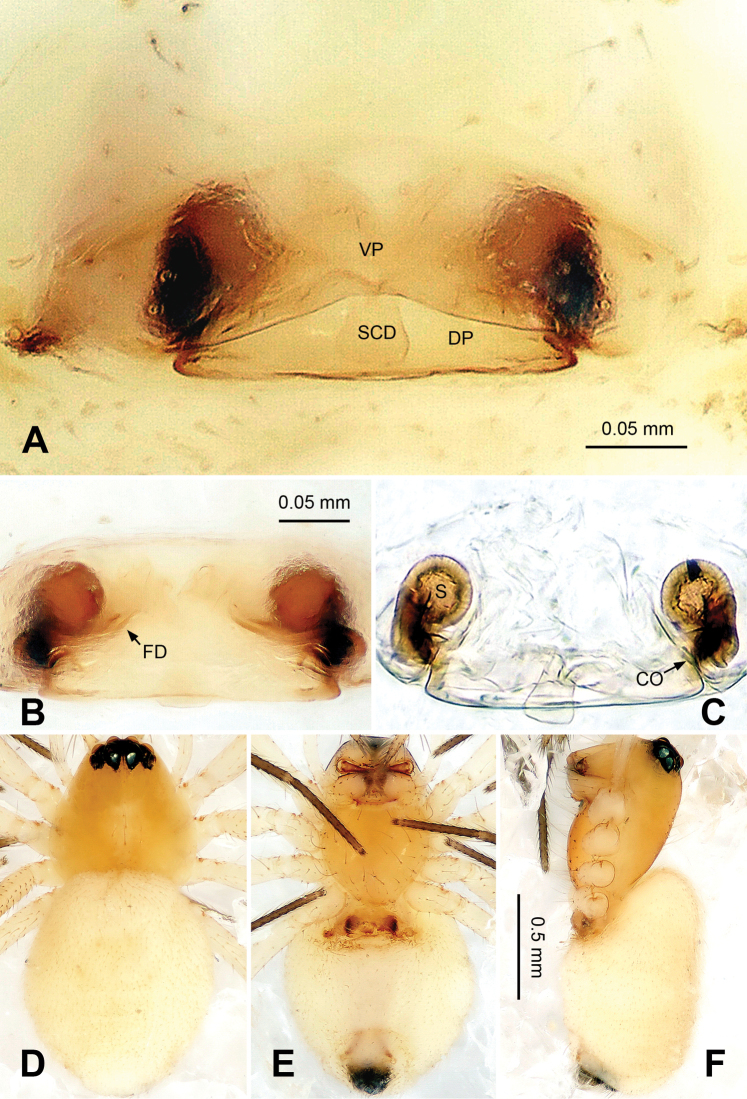
*Vittatus pan* sp. n., female paratype. **A** Epigyne, ventral view **B** Epigyne, dorsal view **C** Vulva, dorsal view **D** Habitus, dorsal view **E** Habitus, ventral view **F** Habitus lateral view. Scale bars: **C** as **B**, **D** and **E** as **F.**

**Figure 119.**
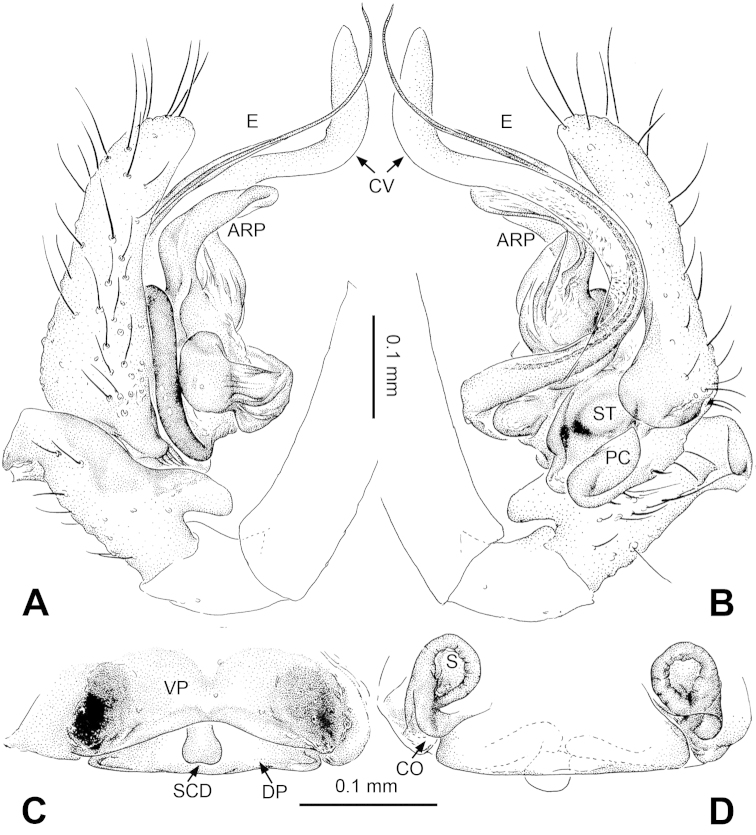
*Vittatus pan* sp. n., male holotype (**A**–**B**) and female paratype (**C**–**D**). **A** Palp, prolateral view **B** Palp, retrolateral view **C** Epigyne, ventral view **D** Vulva, dorsal view. Scale bar: **A** as **B**, **C** as **D.**

**Figure 120.**
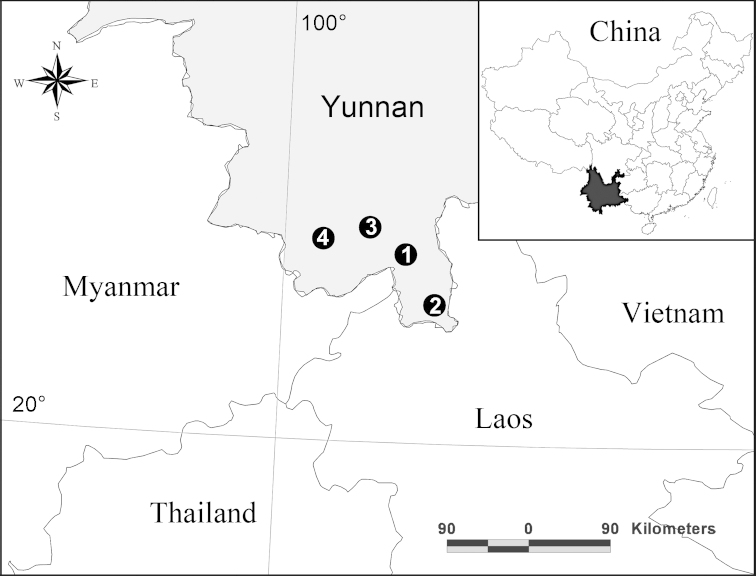
Four main collection localities in Xishuangbanna, Yunnan China. **1** Menglun Town **2** Mengla Town **3** Mengyang Town **4** Menghai Town.

Figs 116 , 117 , 118 , 119

###### Types.

Holotype ♂: CHINA, Yunnan: Menglun Town: Xishuangbanna Tropical Botanical Garden, 21°54.772'N, 101°16.043'E, elevation ca 556 m, 18.07.2007, *Paramichelia baillonii* plantation, fogging. Paratypes 2♀, 21°55.035'N, 101°16.500'E, elevation ca 558 m, 22.07.2007, primary tropical seasonal forest, fogging; 5♂1♀, 21°57.809'N, 101°12.173'E, elevation ca 888 m, 4.08.2007, secondary tropical montane evergreen broad-leaved forest, fogging.

###### Etymology.

This name is derived from the Chinese Pinyin ‘pán’ meaning ‘fig’, for the broad, fig-like terminal apophysis in this species; noun in apposition.

###### Diagnosis.

It differs from the congener *Vittatus bian* sp. n. by light coloration, much shorter dorsal tibial apophysis (Fig. 117A), prominent and broad branch of radical apophysis (Fig. 117B) and short scape (as long as wide).

###### Description.

Male (holotype). Total length: 1.56. Carapace 0.69 long, 0.59 wide, yellow, PME area elevated to form a lobe, post-ocular sulci stretching along base of lobe laterally (Fig. 53E). Sternum 0.44 long, 0.44 wide. Clypeus 0.15 high. Eye sizes and interdistances: AME 0.06, ALE 0.08, PME 0.06, PLE 0.08, AME-AME/AME 0.50, PME-PME/PME 0.50, AME-ALE/ALE 0.50, PME-PLE/PLE 1.13, coxae IV separated by 2.00 times their width. Length of legs: I 2.83 (0.75, 0.19, 0.75, 0.71, 0.43), II 2.88 (0.75, 0.23, 0.75, 0.70, 0.45), III 2.22 (0.61, 0.17, 0.55, 0.55, 0.34), IV 2.67 (0.73, 0.17, 0.67, 0.69, 0.41). Leg formula: II-I-IV-III. TmI 0.54, TmIV 0.51. Abdomen pale, with a small black patch posteriorly (Fig. 117C). Palp: tibia with two retrolateral one dorsal trichobothria, a row of setae retrolaterally (Fig. 116B) and two tibial apophyses, ventral and dorsal; dorsal apophysis short, and scale-like, with a bent tip (Figs 116B, 117A); ventral apophysis short and stout with a rounded tip (Fig. 116A); paracymbium ‘J’-shaped, with a leaf-shaped apex (Fig. 116B); radical apophysis with two prominent branches, one stretching along the cymbium, broad and slightly bent distally (Fig. 116A), the other smaller with an attenuated tip (Fig. 116B); convector long, sigmoid (Fig. 116B).

Female (one of paratypes). Total length: 1.50. Carapace 0.63 long, 0.55 wide, unmodified, yellow. Sternum 0.41 long, 0.45 wide. Clypeus 0.09 high. Chelicerae promargin with 4 teeth, retromargin with 4 teeth. Eye sizes and interdistances: AME 0.06, ALE 0.07, PME 0.06, PLE 0.07, AME-AME/AME 0.33, PME-PME/PME 0.17, AME-ALE/ALE 0.23, PME-PLE/PLE 0.33, coxae IV separated by 1.92 times their width. Length of legs: I 2.67 (0.72, 0.22, 0.68, 0.65, 0.40), II 2.64 (0.70, 0.20, 0.64, 0.68, 0.42), III 2.26 (0.63, 0.19, 0.55, 0.55, 0.34), IV 2.81 (0.78, 0.22, 0.70, 0.70, 0.41). Leg formula: IV-I-II-III. TmI 0.80, TmIV 0.70. Abdomen light yellow, with dark spinnerets. Epigyne: ventral fig wide, with arc-like posterior margin (Figs 118A, 119C); scapoid short, tongue-shaped, as long as wide; dorsal fig wide; copulatory ducts short (Figs 118C, 119D); spermathecae round, separated by 2.5 diameters (Fig. 118B); fertilization ducts relatively long (Fig. 118B).

###### Distribution.

Known only from type localities.

## Discussion

**Figure 121.**
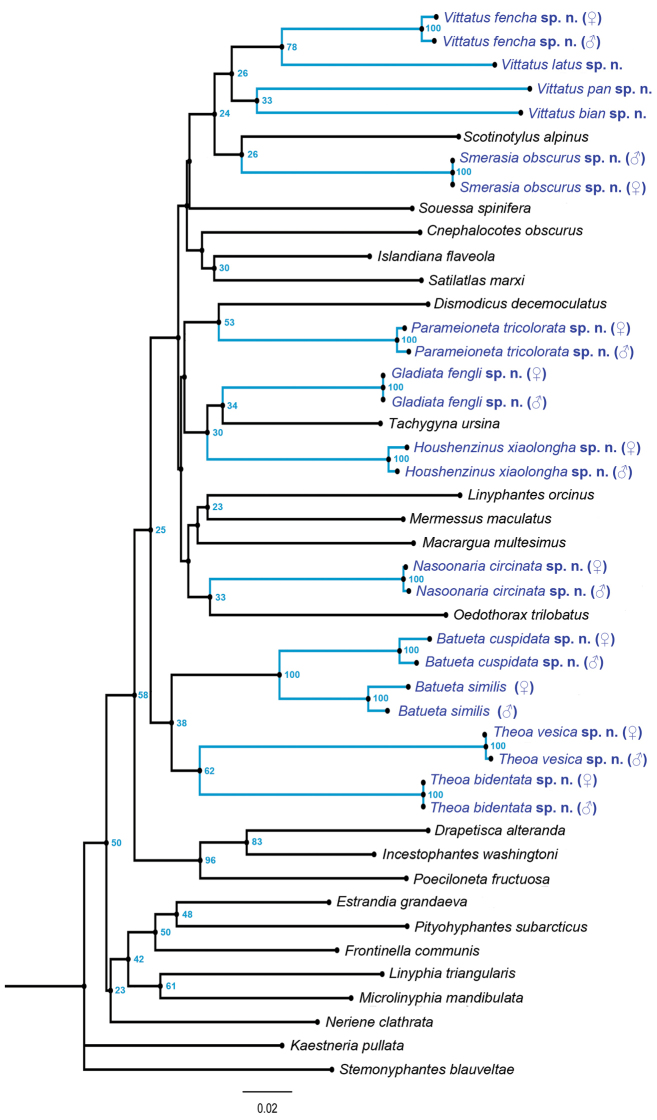
Neighor-joining tree based on DNA barcode of the mitochondrial COI sequences from paired Xishaungbanna linyphiids (marked in blue) and sequences deposited in BOLD systems (marked in black). (K2P model, bootstrap=1000, bootstrap values less than 20% are not shown).

The process of matching the sexes of the Xishuangbanna linyphiids collected from the forest canopy is arduous, especially with the occurrence of similar habitus and conformation of epigynes (Figs 46, 62, 99). In order to solve this problem, we conducted molecular work to obtain DNA sequence barcodes of COI from freshly collected linyphiid spiders (mostly from canopies), and constructed a Neighbor-joining tree. The pairing results are shown in Figure 121.

We have described some new species based on only one sex, which is an unconventional act. However, we feel reasonably certain that our conclusions are generally valid and taxonomically informative. While there may be errors in the identification of some species, finding the other sex of monotypic species will take much time and effort, and we believe it is best to share our information as soon as possible. Further investigations should be conducted in Xishuangbanna and neighboring places. More new discoveries will definitely benefit our understanding of the systematics of the linyphiids from Southeast Asia.

## Supplementary Material

XML Treatment for Agyneta nigra

XML Treatment for Asiagone perforata

XML Treatment for Atypena cirrifrons

XML Treatment for Bathyphantes paracymbialis

XML Treatment for Batueta cuspidata

XML Treatment for Batueta similis

XML Treatment for Capsulia laciniosa

XML Treatment for Cirrosus

XML Treatment for Cirrosus atrocaudatus

XML Treatment for Conglin

XML Treatment for Conglin personatus

XML Treatment for Curtimeticus

XML Treatment for Curtimeticus nebulosus

XML Treatment for Dactylopisthes separatus

XML Treatment for Erigone grandidens

XML Treatment for Gladiata

XML Treatment for Gladiata fengli

XML Treatment for Glebala

XML Treatment for Glebala aspera

XML Treatment for Glomerosus

XML Treatment for Glomerosus lateralis

XML Treatment for Gongylidiellum bracteatum

XML Treatment for Houshenzinus xiaolongha

XML Treatment for Hylyphantes graminicola

XML Treatment for Kaestneria bicultrata

XML Treatment for Laogone bai

XML Treatment for Laogone lunata

XML Treatment for Maro bulbosus

XML Treatment for Nasoona asocialis

XML Treatment for Nasoona crucifera

XML Treatment for Nasoonaria circinata

XML Treatment for Nasoonaria sinensis

XML Treatment for Nematogmus sanguinolentus

XML Treatment for Neriene circifolia

XML Treatment for Neriene macella

XML Treatment for Neriene nitens

XML Treatment for Neriene strandia

XML Treatment for Oedothorax biantu

XML Treatment for Oilinyphia hengji

XML Treatment for Paikiniana furcata

XML Treatment for Parameioneta bishou

XML Treatment for Parameioneta multifida

XML Treatment for Parameioneta tricolorata

XML Treatment for Prosoponoides hamatus

XML Treatment for Saitonia kawaguchikonis

XML Treatment for Smerasia

XML Treatment for Smerasia obscurus

XML Treatment for Tapinopa undata

XML Treatment for Tapinopa vara

XML Treatment for Theoa bidentata

XML Treatment for Theoa vesica

XML Treatment for Vittatus

XML Treatment for Vittatus bian

XML Treatment for Vittatus fencha

XML Treatment for Vittatus latus

XML Treatment for Vittatus pan

## Figures and Tables

**Figure 6.**
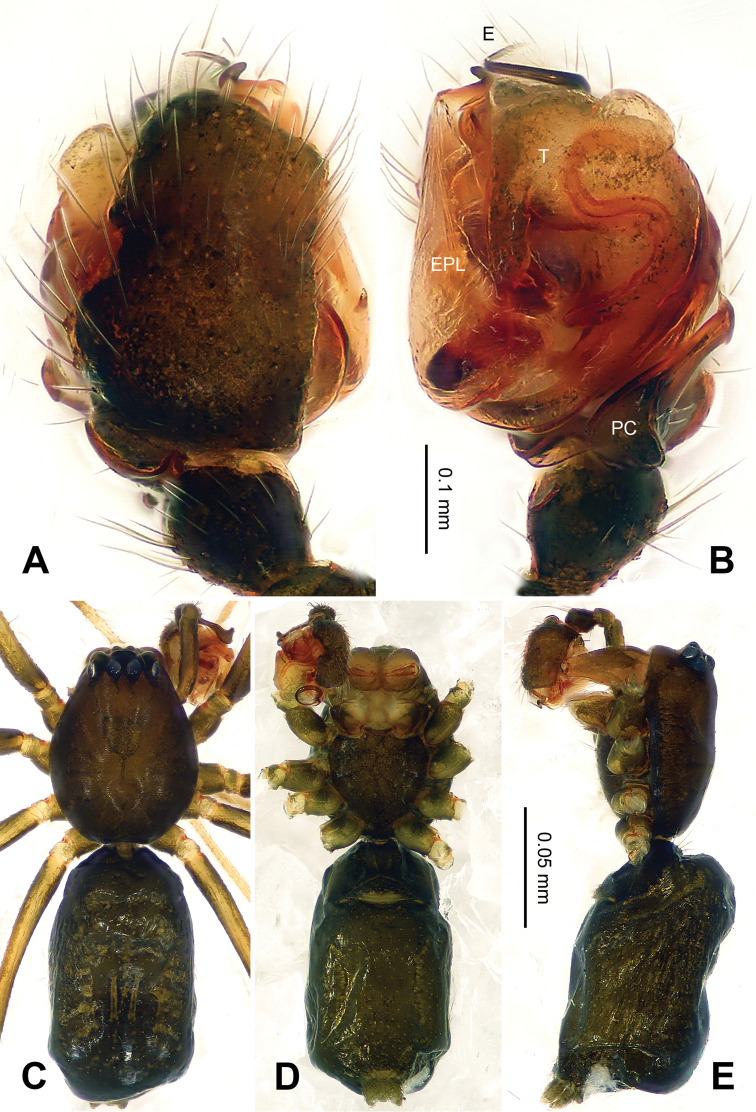
*Bathyphantes paracymbialis.* **A** Palp, dorsal view **B** Palp, ventral view **C** Habitus, dorsal view **D** Habitus, ventral view **E** Habitus, lateral view. Scale bars: **A** as **B**, **C** and **D** as **E.**

**Figure 8.**
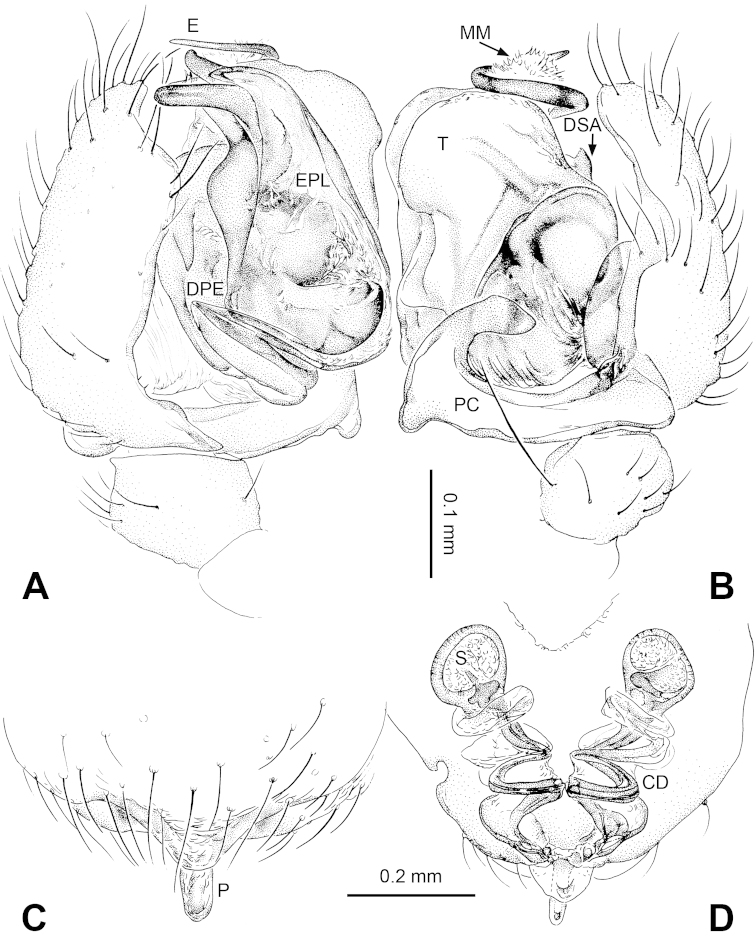
*Bathyphantes paracymbialis.* **A** palp, prolateral view **b** palp, retrolateral view **c** epigyne, ventral view **d** vulva, dorsal view. Scale bars: **A** as **B**, **C** as **D.**

**Figure 25.**
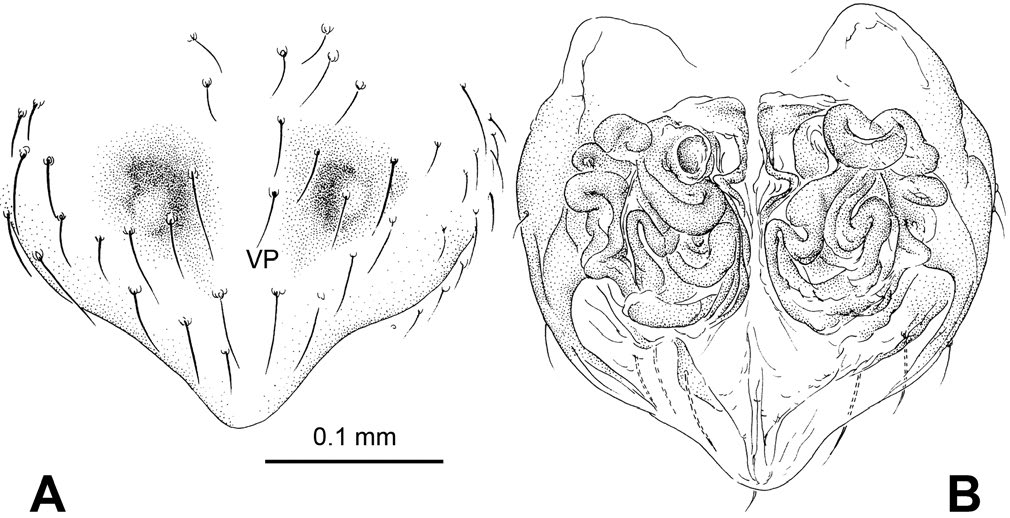
*Conglin personatus* sp. n., female holotype. **A** Epigyne, ventral view **B** Vulva, dorsal view Scale bar: **B** as **A.**

**Figure 34.** *Gladiata fengli* sp. n., female paratype. **A** Epigyne, ventral view **B** Epigyne, dorsal view **C** Vulva, dorsal view **D** Habitus, dorsal view **E** Habitus, ventral view **F** Habitus lateral view. Scale bars: **B** as **C**, **D** and **F** as **E.**

**Figure 38.**
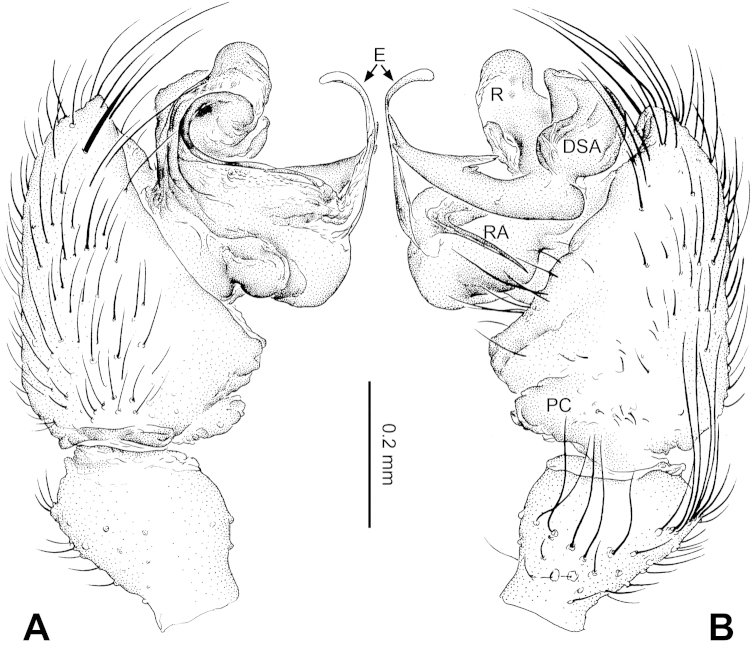
*Glebala aspera* sp. n., male holotype. **A** Right palp, prolateral view **B** Right palp, retrolateral view. Scale bar: **A** as **B.**

**Figure 94.**
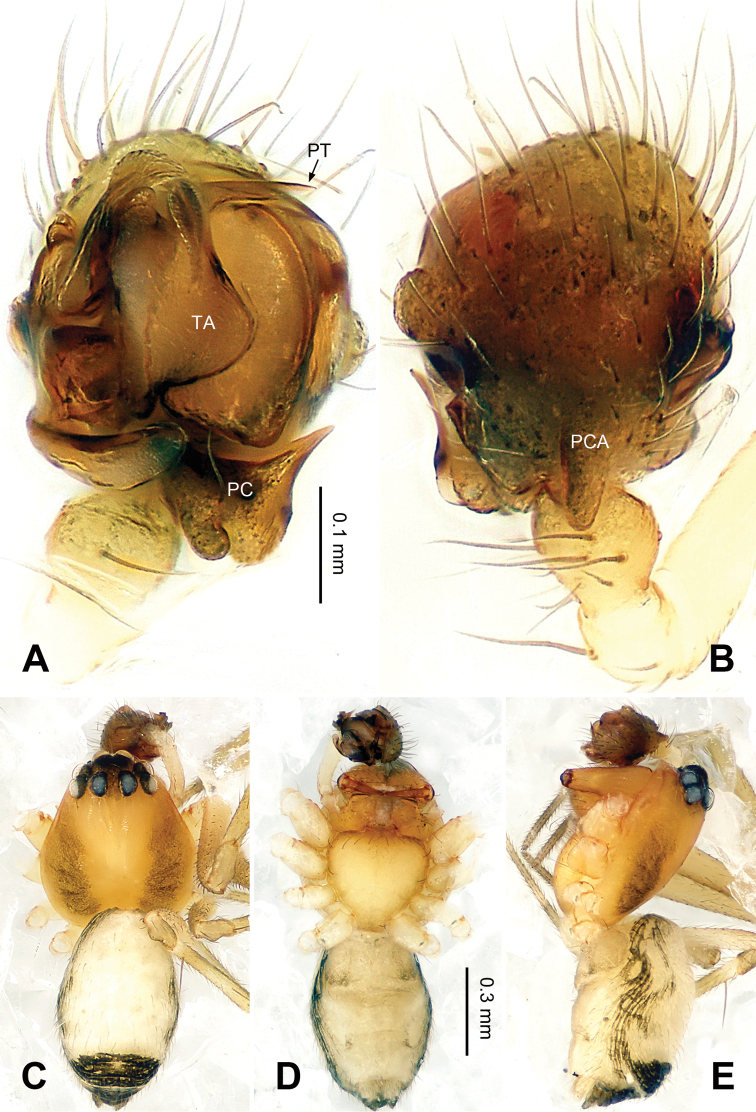
*Tapinopa undata* sp. n., male holotype. **A** Palp, ventral view **B** Palp, dorsal view **C** Habitus, dorsal view **D** Habitus, ventral view **E** Habitus, lateral view. Scale bars: **B** as **A**, **C** and **E** as **D.**
